# Causes and Effects of Loss of Classical Nonhomologous End Joining Pathway in Parasitic Eukaryotes

**DOI:** 10.1128/mBio.01541-19

**Published:** 2019-07-16

**Authors:** Anna Nenarokova, Kristína Záhonová, Marija Krasilnikova, Ondřej Gahura, Richard McCulloch, Alena Zíková, Vyacheslav Yurchenko, Julius Lukeš

**Affiliations:** a Institute of Parasitology, Biology Centre, Czech Academy of Sciences, České Budějovice, Czech Republic; b Faculty of Science, University of South Bohemia, České Budějovice, Czech Republic; c Department of Parasitology, Faculty of Science, Charles University, BIOCEV, Prague, Czech Republic; d Wellcome Centre for Molecular Parasitology, College of Medical, Veterinary and Life Sciences, University of Glasgow, Glasgow, Scotland; e Martsinovsky Institute of Medical Parasitology, Sechenov University, Moscow, Russia; f Life Science Research Centre and Institute of Environmental Technologies, Faculty of Science, University of Ostrava, Ostrava, Czech Republic; Duke University; McGill University; Centre of Research in Infectious Disease, Laval University

**Keywords:** DNA repair, genome size, parasite

## Abstract

We report frequent losses of components of the classical nonhomologous end joining pathway (C-NHEJ), one of the main eukaryotic tools for end joining repair of DNA double-strand breaks, in several lineages of parasitic protists. Moreover, we have identified a single lineage among trypanosomatid flagellates that has lost Ku70 and Ku80, the core C-NHEJ components, and accumulated numerous insertions in many protein-coding genes. We propose a correlation between these two phenomena and discuss the possible impact of the C-NHEJ loss on genome evolution and transition to the parasitic lifestyle.

## Abstract (precis)

Parasites tend to evolve small and compact genomes, generally endowed with a high mutation rate, compared with those of their free-living relatives. However, the mechanisms by which they achieve these features, independently in unrelated lineages, remain largely unknown. We argue that the loss of the classical nonhomologous end joining pathway components may be one of the crucial steps responsible for characteristic features of parasite genomes.

## OBSERVATION

While DNA integrity and genome stability are crucial for all living organisms, they are permanently challenged by various factors causing DNA damage. The most deleterious DNA lesions are double-strand breaks (DSBs), since accurate repair of one strand using the other one as a template, as occurs in other types of DNA damage, is not possible in this case. To fix such an extreme type of damage, cells have evolved repair mechanisms known as homologous recombination (HR) and nonhomologous end joining (NHEJ).

HR, which relies on the presence of a homologous intact template, starts with 5′-to-3′ resection at the DSB, producing 3′ overhangs usually longer than 100 nucleotides. At least one of the single strand ends invades the homologous region of an intact chromosome, preferentially the sister chromatid (1). This strand invasion of single-stranded DNA into a template sequence produces a displacement loop (D-loop) and is mediated by recombinases of the RecA/Rad51/RadA family, found in all three domains of life (2). Upon invasion, the free 3′ end of the strand is then extended by DNA polymerase(s). Subsequent steps diverge into one of the three pathways with various mutagenic potentials: (i) the double Holliday junction (dHJ) pathway engages both ends of the DSB and can lead to sequence crossover between the broken and intact molecules, (ii) synthesis-dependent strand annealing initially involves only one DSB end, and (iii) break-induced replication employs only one end of the break and can copy many kilobases from the donor sequence (3–5).

In contrast to HR, NHEJ repairs a DSB by religating the broken ends without engaging an unbroken homologous template. It is divided into two main types, classical (C-NHEJ) and alternative (A-NHEJ) NHEJ. Unlike A-NHEJ, C-NHEJ has no enzymatic overlap with HR and in mammals is directed by five core components: Ku70/Ku80 heterodimer (Ku), DNA-dependent protein kinase catalytic subunit (DNA-PKcs), DNA ligase IV (Lig4), and the XRCC4 and XLF proteins (6–8). The Ku heterodimer first recognizes and binds a DSB in a sequence-independent manner, preventing extensive DSB end resection and serving as a scaffold on which other components of the C-NHEJ machinery are subsequently assembled.

Ku recruits DNA-PKcs, with which it forms a stable complex, tethers the broken DNA ends, and blocks access of other proteins. The lesion is processed, and DNA ends are sealed by the Lig4-XRCC4-XLF complex. Depending on the type of DNA end (overhang or blunt end), other factors (such as the endonuclease Artemis and DNA polymerases) and processes (end resection and DNA synthesis) may also be involved in this repair mechanism (6, 7).

The C-NHEJ machinery is conserved from bacteria to higher eukaryotes, although the levels of conservation of its components differ. In eukaryotes, the Ku heterodimer and Lig4 represent its core. Other components are less conserved and may even be absent. While retained in animals (9, 10), DNA-PKcs is absent in the yeast Saccharomyces cerevisiae, in which its roles are carried out by the MRX complex (11). Whether the absence of DNA-PKcs results in a reduced use of C-NHEJ is unclear, though yeasts certainly use HR as the main mechanism for DSB repair (12). Bacterial C-NHEJ employs a reduced enzymatic machinery, which comprises a Ku homodimer, homologous to eukaryotic Ku70 and Ku80, and a DNA ligase often fused to other functional domains (13–16). C-NHEJ in Archaea also utilizes a Ku homodimer, but with a different DNA ligase, DNA polymerase, and phosphodiesterase, all of which nonetheless appear closely related to their bacterial homologues (17).

Although the C-NHEJ pathway is often considered more error-prone than the HR pathway, this view has been challenged recently by emerging evidence that the latter can often be erroneous as well, especially in large and repetitive genomes (3, 18), whereas the C-NHEJ is often robust and accurate (19). However, such fidelity does not apply to the A-NHEJ pathways, named microhomology-mediated end joining (MMEJ) and single-strand annealing (SSA). Both are frequently associated with deletions, since they rely on short regions of homology around a DSB, revealed by more extensive DSB processing than in the case of C-NHEJ. The SSA pathway is independent of Rad51 but operates by annealing 25- to 400-bp-long stretches of high sequence homology in a Rad52-dependent reaction, suggesting at least some functional overlap with the HR machinery (3–5). Since such long stretches of homology are relatively rare, SSA normally generates large deletions around the DSB and is often associated with tandem repeats. MMEJ also results in deletions (20), but the shorter lengths of homology needed for strand annealing, allied to the reaction’s tolerance of mismatches, ensure that deletions are normally less extensive. However, the same substrate requirements also imply that MMEJ can cause translocations, as well as complex deletions/insertions, where insertions are usually 2- to 30-bp-long, reiterating either adjacent or distant sequences (21, 22).

In metazoans, MMEJ is facilitated by poly(ADP-ribose) polymerase 1 (23), while DSB recognition requires additional proteins. Six- to 20-bp-long microhomologies are used to allow annealing around the processed DSB (24, 25), the overhangs are cleaved off, and single-stranded gaps are filled in and ligated by DNA ligases I and/or III (26, 27). Another key component of metazoan MMEJ is DNA polymerase theta (Pol θ), which possesses both polymerase and helicase domains, tethers DSB ends, anneals the broken ends at microhomology sites, and synthesizes DNA in template-dependent and -independent manners to allow DSB religation (21, 28–31). Despite this central role in MMEJ, Pol θ is not present in all organisms. For example, yeasts employ other polymerases for this purpose (32).

The HR pathway predominates in the S and G_2_ phases of the cell cycle, when newly replicated, homologous sister chromatids are present. In contrast, Ku-dependent C-NHEJ operates during the whole cell cycle, being the major DSB repair mechanism in multicellular eukaryotes (12, 33, 34). Whether MMEJ or SSA is limited to specific parts of the cell cycle is unclear.

## 

### Parasites tend to lose C-NHEJ.

Perhaps because C-NHEJ is not the sole mechanism of end joining in eukaryotes, the pathway has been lost in several lineages (32, 35, 36). Prominent among the organisms lacking C-NHEJ are parasites. The absence of C-NHEJ components has been documented for the human parasitic protists Trypanosoma spp. (37), *Plasmodium* spp. (38), and Encephalitozoon cuniculi (39). Experimental analysis of DSB repair has shown that only A-NHEJ and not C-NHEJ is used in at least two of these genera (40–45).

**FIG 1:**
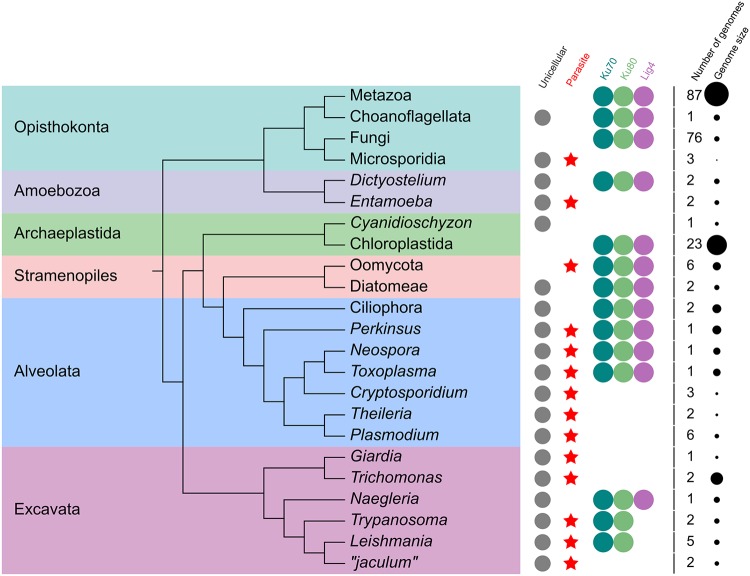
Distribution of main C-NHEJ components across eukaryotes. Median genome size is represented as black circles of corresponding size.

To understand the phylogenetic distribution of C-NHEJ across eukaryotes, we searched for the orthologues of Ku70, Ku80, and Lig4, since these are the main widely conserved factors (Fig. 1).

From 230 eukaryotic genomes present in the EggNOG database (the genome of Aspergillus oryzae, in which Ku70 and Ku80 were artificially deleted to make HR more effective, was not included), 181, 26, and 3 genomes encoded all three, two, and one component, respectively, and in 20 genomes, all three components were missing (Table S1). The analysis revealed an overall trend of parasitic protists to lack the C-NHEJ pathway. For example, C-NHEJ is lost in microsporidia and *Entamoeba* spp., yet it is retained in free-living fungi (46) and *Dictyostelium* spp. that form their sister clades, respectively. Nonetheless, this rule is not without exceptions. Among apicomplexan parasites, all C-NHEJ components were retained in the genera *Toxoplasma* and *Neospora* yet lost in *Plasmodium*, *Cryptosporidium*, and *Theileria*. Moreover, C-NHEJ is absent in the red alga Cyanidioschyzon merolae, the only known free-living protist lacking it (Fig. 1). Such a sporadic absence of C-NHEJ is most readily explained by multiple independent losses during eukaryotic evolution.

10.1128/mBio.01541-19.2 TABLE S1 Presence of proteins of repair pathways in studied organisms from the EggNOG database. Download Table S1, XLSX file, 0.6 MB. Copyright © 2019 Nenarokova et al. 2019 Nenarokova et al. This content is distributed under the terms of the Creative Commons Attribution 4.0 International license.

### Why parasites?

Two important questions arise from the observation that multiple eukaryotic lineages have discarded C-NHEJ. What processes and forces triggered the loss of such an important DNA repair pathway? What consequences might it have for genome stability and structure?

It has been suggested that the distribution of C-NHEJ in bacteria is connected with their life cycle, with the pathway present in species with a prolonged stationary phase (47, 48), during which there is no available sister chromatid to perform HR. This is also consistent with the observed predominance of C-NHEJ in the haploid cells of eukaryotes, as well as in the G_1_ or G_0_ phase of the cell cycle, when HR cannot be implemented and the cell has to rely on the nonhomologous DSB repair pathways (49, 50). Vice versa, the organisms that divide often and spend long time in the diploid state tend to rely on HR and lose C-NHEJ.

Alternatively, the loss of C-NHEJ may be triggered by an attempt to limit or even eradicate transposons that rely on it for their movement (51). Finally, the patchy distribution of different DSB repair pathways may reflect their relative impact on genome changes. For example, C-NHEJ can be mutagenic, contributing to sequence diversity during maturation of vertebrate immune genes (52). Consequently, the balance between the beneficial and detrimental aspects of C-NHEJ-associated mutagenesis (53) may dictate the need for its loss, facilitating use of the more faithful HR. However, the absence of C-NHEJ also results in a higher dependence on the A-NHEJ pathway, as appears to be the case during DSB repair in trypanosomatids and other organisms without C-NHEJ (40–45, 54, 55). Such prominence of A-NHEJ may become useful because of additional functions that C-NHEJ cannot perform, such as enhanced genome rearrangement, due to the reliance of A-NHEJ on annealing short, imperfect regions of homology. However, at least in the case of trypanosomatids, the extensive synteny of the Trypanosoma brucei, Trypanosoma cruzi, and *Leishmania* genomes (56) argues against the function of A-NHEJ in genome rearrangements, although we cannot exclude its reclusive role in localized genome variation, such as in multigene families (57–59).

Instead, loss of C-NHEJ can be better correlated with reduced genome size. For instance, the chordate *Oikopleura* (54), the red alga *Cyanidioschyzon* (60), and the prokaryote Mycobacterium leprae (61) have undergone a process of genome compaction and, unlike their relatives, notably lack C-NHEJ. Similarly, the size range from 8 to 23 Mb of the C-NHEJ-lacking genomes of the apicomplexans Theileria parva (62), *Cryptosporidium* spp. (63), and *Plasmodium* spp. (64) is significantly smaller than the 80-Mb genome of the related Toxoplasma gondii (65) (Fig. 1). The loss of C-NHEJ and subsequent gradual compaction of the genome were also observed in the evolution of microsporidians (46, 66). Importantly, Deng and colleagues associated the genome compaction in *Oikopleura* with the loss of C-NHEJ machinery (54). Consistent with this suggestion, our comparative analysis of eukaryotic genomes lacking and containing C-NHEJ machinery revealed a mean size of 29.2 Mb for the former and 667.9 Mb for the latter, a remarkable difference of >20 times (*P* = 1.0 ×10^−8^). While this cannot be the sole explanation of size differences, since the ∼165-Mb genome of Trichomonas vaginalis (67) also lacks C-NHEJ machinery (although its close relative Trichomonas tenax has a genome of only 46 Mb [68]), it is highly plausible that when genome streamlining is advantageous, C-NHEJ tends to be discarded, either due to its dispensability or because this step further accelerates sequence loss.

Selective pressure makes parasites fast, concise, and economic, preferably exceeding their hosts in these parameters. Moreover, compared with their free-living relatives, parasites typically have smaller and streamlined genomes and are more susceptible to gene loss. All this is beneficial, since smaller genomes allow parasites to multiply faster and with lower metabolic costs (69, 70). In this context, we posit that the observed multiple independent losses of the C-NHEJ components in parasitic lineages provide evidence that loss of this DSB repair mechanism leads to genome compaction and, in turn, provides parasites with a number of selective advantages detailed below.

At a DSB, the Ku heterodimer binds promptly to the broken DNA ends (71), protecting them from further degradation and resection by nucleases, which would lead to deleterious deletions (72). In the absence of C-NHEJ, the organism uses A-NHEJ pathways, such as MMEJ and SSA, which inevitably triggers sequence deletions (20). Moreover, the HR-based break-induced replication and SSA pathways can also produce deletions at the breakpoint flanks (73, 74). Thus, following the loss of C-NHEJ, a eukaryotic genome undergoes chromosome aberrations, including deletions and translocations, leading to loss of genetic material and consequent genome shrinkage (75–77). For instance, it has been experimentally demonstrated that A-NHEJ causes novel indel mutations in *Oikopleura*, and this process was implicated in the mechanism of genome shrinkage (54).

We may speculate about the potential mechanisms behind the genome shrinkage. Keeling and Slamovits considered two principal ways leading to the shrinkage of a genome, which are not mutually exclusive: reduction and compaction (78). Reduction is a process of elimination of some functional elements, such as protein-coding genes, whereas compaction is a process of rearranging the existing functional elements in a denser way, for instance, by removing the parts of the noncoding sequences. Both processes operate in the eukaryote genomes: they can occur together or separately. The smallest known nuclear genomes are those of parasitic microsporidia (2.5 Mb) and nucleomorphs (0.373 Mb). They represent extreme cases of both processes, having the highest gene density and the smallest number of genes among eukaryotes (78).

The physical mechanism of genome shrinkage is the loss of whole chromosomes (aneuploidy) or their parts (deletion mutations). Aneuploidy occurs due to the erroneous cell division when the chromosomes do not distribute correctly between the daughter cells. Large deletions originate as a result of DSB without rejoining, translocation of mobile elements, or erroneous, unequal, and ectopic recombination, such as between repeated regions. It is probable that this recombination is more likely to occur in the noncoding parts of genome, which have more repeated elements than protein-coding sequences, causing genome compaction (79). Small deletions occur as a result of DNA polymerase errors, such as slippering on repeats (80). Comparative studies of various animal genomes showed that on the level of small (<400-bp) indels, deletions prevail over insertions both in the protein-coding genes (81) and in the noncoding sequences (82), which may also lead to gradual loss of genetic material.

Still, we cannot exclude the possibility that loss of the C-NHEJ pathway is not the cause but rather the consequence of genome shrinkage. Even though HR occurs in mammals, C-NHEJ acts as their main DSB repair pathway (12, 33, 34). However, eukaryotes with smaller genomes and functional C-NHEJ, such as S. cerevisiae, preferably employ HR for DSB repair (12). There is at least one reason for C-NHEJ being the main DSB repair pathway in large eukaryotic genomes. The search for a homologous sequence during HR occurs across the entire genome, raising the risk of invading homologous ectopic sequences, which is especially high given the abundance of almost identical retrotransposon repeats in such genomes (3, 18, 83). In contrast, HR may be the mechanism of choice in small, nonrepetitive genomes, such as those of most bacteria and some unicellular eukaryotes, including parasites. The dependence of HR on the presence of homologous chromatids implies that during haploid cell cycle stages, organisms without C-NHEJ must rely on other repair pathways, such as MMEJ and/or SSA. However, as mentioned above, these pathways are highly error-prone, with a tendency to generate indel mutations (20, 75, 84–86). While deleterious for free-living eukaryotes, this sloppiness in repair mechanisms may be beneficial for parasites. By depending on these mutagenic pathways, they increase their mutation rate, thus benefiting in the arms race with the host’s immune system (69, 70).

The nonrandom loss of the Ku proteins in parasitic lineages might be also associated with function(s) of the heterodimer in telomere maintenance. Ku is known to protect telomeres from abnormal fusions and has an inhibitory effect on the recombination of normal telomeres. The Ku heterodimer also controls telomere length by recruiting telomerase and is involved in the telomere silencing effect (87–89). Furthermore, chromosomal ends and adjacent subtelomeric regions are of particular importance for parasites, as this is where factors involved in host cell interaction and immune escape mechanisms are frequently located (90, 91). Genes specifying variant surface antigens that allow parasites to evade the hosts’ immune response are often found in the (sub)telomeric regions. Such surface variation systems are known for *Plasmodium* and *Babesia* spp. (64, 92), T. brucei (93, 94), and the fungus Pneumocystis carinii (95). Similar strategies have also been described for several prokaryotic pathogens, such as *Neisseria* spp. (96), Haemophilus influenzae (97), and *Borrelia* spp. (98). Importantly, variation of these polymorphic and fast-evolving surface proteins is promoted by DSBs, at least in the case of T. brucei (99). In the (sub)telomeric regions of P. falciparum, antigenic variation occurs via homologous and ectopic recombination (100–102), which is inhibited by Ku in the organisms that have it (10, 103). In this regard, the retention of Ku in T. brucei and other trypanosomatids, in the absence of other C-NHEJ components, is a notable anomaly.

### Why is Ku retained in trypanosomatids?

**FIG 2:**
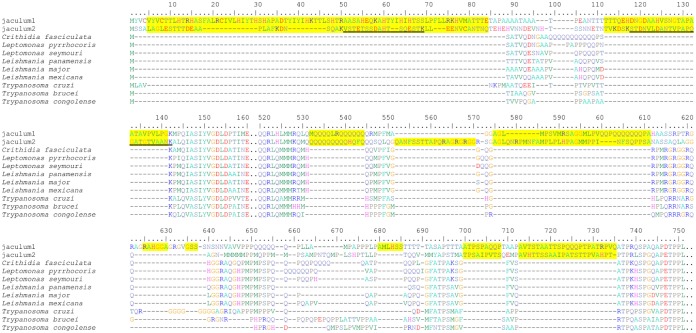
Multiple insertions are present in “*jaculum*” proteins. The N-terminal part of the poly(A)-binding protein alignment of chosen trypanosomatids is shown (full-length alignment is available in Fig. S1). Insertions present in “*jaculum*” proteins are highlighted by yellow background. Peptides identified by mass spectrometry are underlined in black. Two dots represent regions of the sequence alignment that are conserved among the species and were omitted for simplicity.

The human parasites *Trypanosoma* and *Leishmania* (Trypanosomatida, Kinetoplastida) retain Ku70 and Ku80 (104, 105) but have lost Lig4. This is an unusual combination, since other organisms lacking Lig4 usually also do not possess the Ku proteins (Fig. 1). Recently, we have sequenced and annotated the genomes of two unnamed insect flagellates belonging to the “*jaculum*” clade, a novel trypanosomatid lineage (106, 107); the raw sequencing data and the draft assembly are available at NCBI (www.ncbi.nlm.nih.gov) under BioProject PRJNA543408. Their genome sizes are 19.8 Mb and 24.9 Mb in the draft genome assemblies, and the numbers of predicted proteins are 6,163 and 7,571, correspondingly. Unexpectedly, unlike for other trypanosomatids, both Ku genes were ablated from these genomes, proving that the Ku heterodimer is not indispensable for these organisms. Interestingly, a detailed inspection of the genomes of both “*jaculum*” species revealed a high frequency of specific insertions in protein-coding genes, while deletions were rare (Fig. 2; see also Fig. S1 in the supplemental material). Since “*jaculum*” is not a basal trypanosomatid clade, but rather one from the crown (106, 107), and the insertions are specific for this group, the most parsimonious scenario is that the insertions appeared *de novo* in the common ancestor of “*jaculum*.”

10.1128/mBio.01541-19.1 FIG S1 Multiple insertions are present in “*jaculum*” proteins. Full-length protein alignments of chosen trypanosomatids are shown. Insertions present in “*jaculum*” proteins are highlighted by yellow background. Peptides identified by mass spectrometry are underlined in black. To display borders between neighboring identified peptides, black and red underlining is used. Download FIG S1, DOCX file, 0.4 MB. Copyright © 2019 Nenarokova et al. 2019 Nenarokova et al. This content is distributed under the terms of the Creative Commons Attribution 4.0 International license.

Insertions were present in the majority of examined coding sequences, although they were underrepresented or completely absent from the most conserved genes, such as ribosomal proteins and glycolytic enzymes (Table S2). In 247 analyzed alignments in the two “*jaculum*” species, inserted sequences constituted 14.9% and 17.4% of the alignments, whereas in T. brucei only 8.9% of the alignment were represented by insertions (*P*_1_=4.3 × 10^−11^; *P*_2_=1.4 × 10^−13^) (Table S2). We compared the amino acid compositions of insertions and sequences without insertions, and we found that some amino acids were overrepresented or underrepresented in the inserted sequences; however, this pattern was similar in all the analyzed species (Table S3). Mass spectrometry confirmed that the insertions were indeed retained in mature proteins (Fig. 2 and Fig. S1).

10.1128/mBio.01541-19.3 TABLE S2 Comparative analysis of the distribution of insertions. Download Table S2, XLSX file, 0.1 MB. Copyright © 2019 Nenarokova et al. 2019 Nenarokova et al. This content is distributed under the terms of the Creative Commons Attribution 4.0 International license.

10.1128/mBio.01541-19.4 TABLE S3 Amino acid composition of insertions and non-insertion-bearing parts of the proteins. Download Table S3, XLSX file, 0.03 MB. Copyright © 2019 Nenarokova et al. 2019 Nenarokova et al. This content is distributed under the terms of the Creative Commons Attribution 4.0 International license.

**FIG 3:**
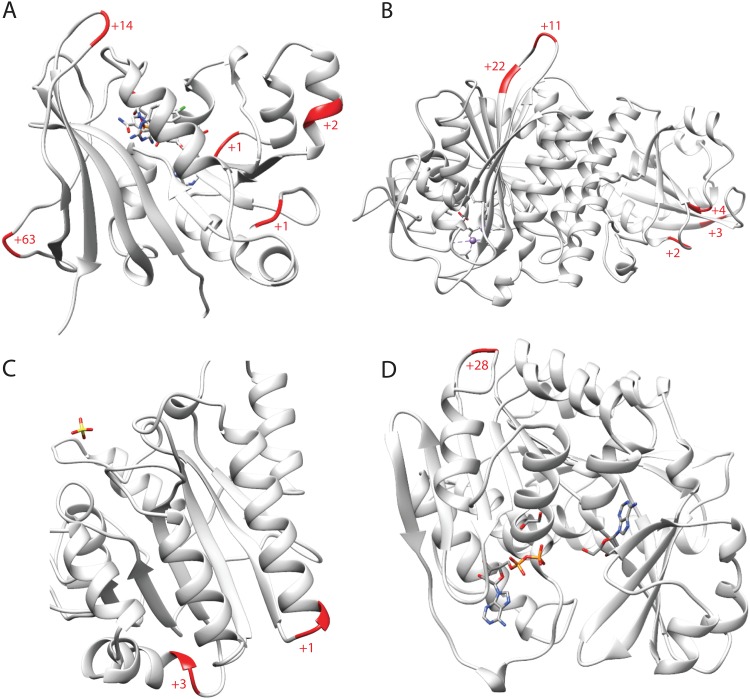
Mapping of insertions in the *“jaculum”* proteins onto structures of dihydrofolate reductase in complex with pyrimethamine (118) (A), leucyl aminopeptidase (119) (B), the phosphatase domain of phosphoglycerate mutase (120) (C), and adenosine kinase in complex with adenosine and AMPPNP (121) (D) from T. brucei. The positions and lengths of insertions in the “*jaculum*” proteins are shown in red.

Next, we investigated whether the observed insertions are neutral with respect to the function of the affected proteins. For that purpose, we mapped the insertions in selected conserved “*jaculum*” proteins on experimentally determined structures of their orthologues in T. brucei (Fig. 3). The inspected insertions either formed terminal extensions or were located to the external loops, but they never occurred in regions involved in ligand binding, ion coordination, or interaction with other molecules. This observation is fully consistent with the hypothesis that all insertions are functionally neutral.

We propose that the observed features are a consequence of the loss of the Ku heterodimer. Moreover, our data suggest an additional, so far unexplored, role(s) of Ku in trypanosomatid parasites. In all examined species, with the sole exception of the “*jaculum*” lineage, Lig4 is absent but both Ku70 and Ku80 are retained (Fig. 1). Data available from Trypanosoma cruzi, T. brucei, and *Leishmania* spp. indicate that the Ku heterodimer does not participate in C-NHEJ and that in the corresponding genomes DSBs are predominantly repaired via HR and MMEJ (37, 43–45, 108). However, it is possible that the Ku70/80 complex plays a role in DSB repair even without its partner Lig4, because it may act as “first aid,” binding within seconds to the disrupted DNA ends (71), holding them together and protecting them from further damage until the slower HR or A-NHEJ proteins come to serve. Such a role may be important in *Leishmania* spp. and T. brucei, in which pronounced levels of genome rearrangements are observed, either genome-wide or in the subtelomeric region for immune evasion, and might involve DNA DSBs (109, 110). Alternatively, Ku70 and Ku80 are involved in other DNA repair pathways, such as base excision and DNA alkylation repair (111), although a role for Ku in these processes has so far not been examined in trypanosomatids. Moreover, together with the MRN complex, the Ku heterodimer may serve as a signaling molecule, modulating activity of the ATM kinase, which phosphorylates other factors and initiates a signaling cascade in the DNA damage response pathway (10). Again, the function of the ATM kinase has not yet been scrutinized in trypanosomatids. Finally, the Ku proteins play an important role in telomere maintenance (104, 105, 112). Data obtained from the analysis of the “*jaculum*”genomes may shed light on the genome-wide roles of these conserved and multifunctional proteins not only in trypanosomatids but also in other eukaryotes.

Taking the alternative end joining pathways into consideration may give us a hint regarding the origin of the insertions that are prominent in “*jaculum*.” In metazoan MMEJ, DNA polymerase θ uses only one to four complementary nucleotides to initiate polymerization, frequently producing short templated and nontemplated insertions (113, 114), reminiscent of those pervading the “*jaculum*” genome. We consider as highly plausible a hypothesis that in the “*jaculum*” trypanosomatids, the insertions may result from the erroneous A-NHEJ and HR repair processes, unconstrained by the Ku proteins. Similarly, in tunicate Oikopleura dioica, which lacks Ku70/80 and other components of C-NHEJ, DSB repair by A-NHEJ results in acquisition of multiple novel insertions (54).

An interesting question is why the observed insertions in “*jaculum*” and other trypanosomatids were significantly prevalent over deletions (Fig. 2 and Fig. S1). It is known that insertions in protein-coding sequences are usually several times more frequent than deletions, apparently because the latter are generally more deleterious and more susceptible to purifying selection (115). We also noticed that amino acids are predominantly altered in the flanking regions of the insertions and may represent remnants of the deletions, rendering these parts of the alignment to be inaccurately aligned. Moreover, the lengths of the inserted region are often variable in different species, which may be explained by consequent insertions and deletions (Fig. 2 and Fig. S1).

A comparably high incidence of indel mutations, accompanied by loss of all main C-NHEJ components, has been reported for the causative agent of human malaria, Plasmodium falciparum (42) (Fig. 1). In this protist, the occurrence of indels is over 10-fold higher than that of base substitutions (116). It is therefore worth pointing out that in most other organisms, base substitutions are much more frequent than indels. For example, the substitution-to-indel ratios are approximately 10:1 in primates and 20:1 in bacteria (117). While P. falciparum is known to be a highly polymorphic and fast-evolving parasite (116), these features are so far not associated with the absence of C-NHEJ. The above-described circumstantial evidence makes the putative connection between the DNA repair pathways and the unique features of the *Plasmodium* genomes worth exploring.

### Concluding remarks.

We have found that the C-NHEJ pathway, which is a highly conserved key eukaryotic DNA repair pathway, has been independently lost multiple times in several parasitic protist lineages. We provide several alternative explanations for these seemingly nonrandom losses. Moreover, we raise the question of whether parasites benefit from this repair mechanism or, unlike their free-living kin, try to free themselves from its constraints.
