# Supplementary material for: Causes and Effects of Loss of Classical Nonhomologous End Joining Pathway in Parasitic Eukaryotes
Source: mBio. 2019 Jul 16;10(4):e01541-19. doi: 10.1128/mBio.01541-19 (PMC6635534; doi:10.1128/mBio.01541-19)
Supplement: FIG S1 [file mBio.01541-19-sf001.docx]

**A) polyA-binding protein**

10 20 30 40 50 60 70 80 90 100

....|....|....|....|....|....|....|....|....|....|....|....|....|....|....|....|....|....|....|....|

jaculum1 MYVCVYVCTTLHTRHASFALRCIVLHIYTHSHAPADTYIYIHKTTLSHTRAASAHEQKAHTYIHIHTSSLPFLLRKHVMATTTETAPAAAATAAA---T-

jaculum2 MSSALAGLESTTTDEAA-------------PLAFKDN---------SQAKVSTETSSDAHT--SQESTKLL---EENVCANTNQTEHEHVNNDEVNH-T-

*C. fasciculata* M---------------------------------------------------------------------------------------SATVQDNGAAQQ

*L. pyrrhocoris*  M---------------------------------------------------------------------------------------SATVQDNGAAP-

*L. seymouri*  M---------------------------------------------------------------------------------------SATVQESNAVP-

*L. arabica*  M---------------------------------------------------------------------------------------AAAVQEAAAPV-

*L. aethiopica*  M---------------------------------------------------------------------------------------AAAVQEAAALV-

*L. donovani*  M---------------------------------------------------------------------------------------AAAVQEAAAPV-

*L. gerbilli*  M---------------------------------------------------------------------------------------AAAVQEAAAPV-

*L. enriettii*  M---------------------------------------------------------------------------------------TAIVQETVAPV-

*L. infantum*  M---------------------------------------------------------------------------------------AAAVQEAAAPV-

*L. panamensis*  M---------------------------------------------------------------------------------------TTTVQETATPV-

*L. turanica*  M---------------------------------------------------------------------------------------AAAVQEAAAPV-

*L. tropica*  M---------------------------------------------------------------------------------------AAAVQAAAAPV-

*L. major*  M---------------------------------------------------------------------------------------AAAVQEAAAPV-

*L. mexicana*  M---------------------------------------------------------------------------------------TAVVQQAAAPV-

*T. cruzi*  MLAV--------------------------------------------------------------------------------NKPMAATQEEI---T-

*T. brucei brucei*  M---------------------------------------------------------------------------------------TIAAQGV-----

*T. brucei gambiense* M---------------------------------------------------------------------------------------TIAAQGV-----

*T. evansi*  M---------------------------------------------------------------------------------------TIAAQGV-----

*T. congolense*  M---------------------------------------------------------------------------------------TVVPQGA-----

110 120 130 140 150 160 170 180 190 200

....|....|....|....|....|....|....|....|....|....|....|....|....|....|....|....|....|....|....|....|

jaculum1 ----PEANTTTTTTQEHDNGDAAHVSNGTAPAATAVPVLPGKMPQIASIYVGDLDPTIMEPQLVELFKPFGTILNVRVCRDIITQRSLGYGYVNFDTHDA

jaculum2 ----SSNNETNTVKDSKSTDNVLDANTVPAPQGATGTVAANKALQIASIYVGDLDPTITEPQLVEVFKTFGTILNVRVCRDIITQRSLGYGYVNYDNHSD

*C. fasciculata* QQQQQQQQQPS------------------------------KAMQIASIYVGDLDATINEPQLVELFKSFGTILNVRVCRDIITQRSLGYGYVNFDNHDD

*L. pyrrhocoris*  --PAPPPQQPN------------------------------KPIQIASIYVGDLDATINEPQLVELFKTFGTILNVRVCRDIITQRSLGYGYVNFDNHND

*L. seymouri*  ----QPQQQPT------------------------------KPMQIASIYVGDLDATINEPQLVELFKSFGTILNVRVCRDIITQRSLGYGYVNFDSHED

*L. arabica*  ----AHQPQMD------------------------------KPMQIASIYVGDLDATINEPQLVELFKPFGTILNVRVCRDIITQRSLGYGYVNFDNHDS

*L. aethiopica*  ----AHQPQMD------------------------------KPMQIASIYVGDLDATINEPQLVELFKPFGTILNVRVCRDIITQRSLGYGYVNFDSHDS

*L. donovani*  ----AHQPQMD------------------------------KPMQIASIYVGDLDAAINEPQLVELFKPFGTILNVRVCRDIITQRSLGYGYVNFDNHDS

*L. gerbilli*  ----AHQPQMD------------------------------KPMQIASIYVGDLDATINEPQLVELFKPFGTILNVRVCRDIITQRSLGYGYVNFDNHDS

*L. enriettii*  ----APSQLVG------------------------------KSMHIASIYVGDLDAAINEPQLVEMFKPFGTILNVRVCRDIITQRSLGYGYVNFDNHDS

*L. infantum*  ----AHQPQMD------------------------------KPMQIASIYVGDLDAAINEPQLVELFKPFGTILNVRVCRDIITQRSLGYGYVNFDNHDS

*L. panamensis*  ----AQQPQVS------------------------------KPMQIASIYVGDLDAAINEPQLVELFKPFGTILNVRVCRDIITQRSLGYGYVNFDNHHS

*L. turanica*  ----AHQPQMD------------------------------KPMQIASIYVGDLDATINEPQLVELFKPFGTILNVRVCRDIITQRSLGYGYVNFDNHDS

*L. tropica*  ----AHQPQMD------------------------------KPMQIASIYVGDLDATINEPQLVELFKPFGTILNVRVCRDIITQRSLGYGYVNFDSHDS

*L. major*  ----AHQPQMD------------------------------KPMQIASIYVGDLDATINEPQLVELFKPFGTILNVRVCRDIITQRSLGYGYVNFDNHDS

*L. mexicana*  ----AHQPQVD------------------------------KPMQIASIYVGDLDAAINEPQLVELFKPFGTILNVRVCRDIITQRSLGYGYVNFDNHES

*T. cruzi*  ----PTVPVTT------------------------------KALQVASLYVGDLDPVVTEPHLVELFKPFGTILNVRVCRDIITQRSLGYGYVNFNSHDS

*T. brucei brucei*  ----PSGPSAT------------------------------KPLQVASLYVGDLDPAINEPQLVDIFKPYGTILNVRVCRDIITQRSLGYGYVNYDDVNS

*T. brucei gambiense* ----PSGPSAT------------------------------KPLQVASLYVGDLDPAINEPQLVDIFKPYGTILNVRVCRDIITQRSLGYGYVNYDDVNS

*T. evansi*  ----PSGPSAT------------------------------KPLQVASLYVGDLDPAINEPQLVDIFKPYGTILNVRVCRDIITQRSLGYGYVNYDDVNS

*T. congolense*  ----PPAAPAA------------------------------KQLQVASLYVGDLDPAISEPQLVEIFRPYGTILNVRVCRDIITQRSLGYGYVNYDNANS

210 220 230 240 250 260 270 280 290 300

....|....|....|....|....|....|....|....|....|....|....|....|....|....|....|....|....|....|....|....|

jaculum1 AARAIDTLNFTRVGDKCVRLMWQQRDPALRYSGSGNIFVKNLEANVDSKGLHDLFHKFGAILSCKIMEDEQGKSRGYGFVHFKDEQAAQQAIAKMNGAND

jaculum2 AEKAIETLNFKRIGDKCVRLMWQQRDPALRYSGNGNIFVKNLDQGVDSKGLHDLFEKFGFILSCKIMEDEEGKSRGYGFVHFKDEQAAKKAIEKMNGANE

*C. fasciculata* AEKAIEAMNFKRVGDKCMRLMWQQRDPALRYSGNGNIFVKNLETEVDSKSLHDIFNKFGLILSCKVMEDEEGKSRGYGFVHFKDETSAKDAIVKMNGAGD

*L. pyrrhocoris*  AEKAIEAMNFKRVGDKCMRLMWQQRDPALRYSGNGNIFVKNLETEVDSKSLHDLFTKFGAILSCKVMEDEEGKSRGYGFVHFKEETSATDAIVKMNGSTD

*L. seymouri*  AEKAIEAMNFKRVGDKCMRLMWQQRDPALRYSGNGNIFVKNLETQVDSKSLHDIFTKFGSILSCKVMEDEEGKSRGYGFVHFKDEAAASDAIVKMNGSAD

*L. arabica*  AEKAIESMNFKRVGDKCVRLMWQQRDPALRYSGNGNVFVKNLEKDVDSRSLHDIFTKFGSILSCKVMQDEEGKSRGYGFVHFKDETSAKDAIVQMNGAAD

*L. aethiopica*  AEKAIESMNFKRVGDKCVRLMWQQRDPALRYSGNGNVFVKNLERDVDSKSLHDIFTKFGSILSCKVMQDEEGKSRGYGFVHFKDETSAKDAIVKMNGAAD

*L. donovani*  AEKAIESMNFKRVGDKCVRLMWQQRDPALRYSGNGNVFVKNLEKDVDSKSLHDIFTKFGSILSCKVMQDEEGKSRGYGFVHFKDETSAKDAIVKMNGAAD

*L. gerbilli*  AEKAIESMNFKRVGDKCVRLMWQQRDPALRYSGNGNVFVKNLEKDVDSKSLHDIFTKFGSILSCKVMQDEEGKSRGYGFVHFKDETSAKDAIVKMNGAAD

*L. enriettii*  AEKAIESMNFKRVGDKCVRLMWQQRDPALRYSGNGNVFVKNLEKDVDSKSLHDIFTKFGSILSCKVMQDEEGKSRGYGFVHFKDEASAKDAIVKMNGVPD

*L. infantum*  AEKAIESMNFKRVGDKCVRLMWQQRDPALRYSGNGNVFVKNLEKDVDSKSLHDIFTKFGSILSCKVMQDEEGKSRGYGFVHFKDETSAKDAIVKMNGAAD

*L. panamensis*  AERAIESMNFRRVGDKCVRLMWQQRDPSLRYSGNGNVFVKNLEKDVDSKSLHDIFTKFGSILSCKVMEDEEGKSRGYGFVHFKDEISAKDAIVKMNGAAV

*L. turanica*  AEKAIESMNFKRVGDKCVRLMWQQRDPALRYSGNGNVFVKNLEKDVDSKSLHDIFTKFGSILSCKVMQDEEGKSRGYGFVHFKDETSAKDAIVKMNGAAD

*L. tropica*  AEKAIESMNFKRVGDKCVRLMWQQRDPALRYSGNGNVFVKNLERDVDSKSLHDIFTKFGSILSCKVMQDEEGKSRGYGFVHFKDETSAKDAIVKMNGAAD

*L. major*  AEKAIESMNFKRVGDKCVRLMWQQRDPALRYSGNGNVFVKNLEKDVDSKSLHDIFTKFGSILSCKVMQDEEGKSRGYGFVHFKDETSAKDAIVKMNGAAD

*L. mexicana*  AEKAIESMNFKRVGDKCVRLMWQQRDPALRYSGNGNVFVKNLEKDVDSKSLHDIFTKFGSILSCKVMQDEEGKSRGYGFVHFKDETSAKDAIVKMNGAAD

*T. cruzi*  AAKAMEALNFKQVGDKCMRIMWQQRDPALRYSGNGNIFVKNLKNEVDSRELSVIFKKFGDILSCKVMEDEEGKSRGYGFVHFKNDNDAKEAIEKMNGEKD

*T. brucei brucei*  ASKAMEELNFKRVGEKCIRIMWQQRDPALRYSGSGNIFVKNLKEEVDSRELSLIFKKFGEILSCKVMDDESGKSRGYGFVHFKDDDAAKAAIEKMNGDKE

*T. brucei gambiense* ASKAMEELNFKRVGEKCIRIMWQQRDPALRYSGSGNIFVKNLKEEVDSRELSLIFKKFGEILSCKVMDDESGKSRGYGFVHFKDDDAAKAAIEKMNGDKE

*T. evansi*  ASKAMEELNFKRVGEKCIRIMWQQRDPALRYSGSGNIFVKNLKEEVDSRELSLIFKKFGEILSCKVMDDESGKSRGYGFVHFKDDDAAKAAIEKMNGDKE

*T. congolense*  ATKAMEEMNFKRVGEKCIRIMWQQRDPALRYSGNGNVFVKNLKGEVDSRELSLIFKKFGEILSCKVMDDESGNSRGYGFVHFKDDNAAKSAIESMNGVTE

310 320 330 340 350 360 370 380 390 400

....|....|....|....|....|....|....|....|....|....|....|....|....|....|....|....|....|....|....|....|

jaculum1 HAN-AEKQALYVANFIRRNARLAALVANFTNVYIKQVLPTVDKAAIEAFFAKFGGITSAAACKDKNGRVFAFCNFANHDDAVKAIEAVHDHVIDGITAPD

jaculum2 HAS-ADKHALYVANFIRRNARLAALVANFTNIYIKQVLPTVNKEVIEKFFAKFGGITSAAACKDKSGRVFAFCNFEKHDDAVKAIEAMHDKHIDGITAPE

*C. fasciculata* HAC-EEKKALYVANFIRRNARLAALVANFTNVYIKQVLPTVNKDVIEKFFAKFGGITSAAACKDKSGRVFAFCNFEKHDDAVKAIEAMHDHHIDGITAAG

*L. pyrrhocoris*  HAN-EEKKALYVANFIRRNARLAALVANFTNVYIKQVLPSVNKDVIEKFFAKFGGITSAAACKDKSGRVFAFCNFEKHDDAVKAIEAMHDHHIDGITAPG

*L. seymouri*  HAN-DEKKALYVANFIRRNARLAALVANFTNVYIKQVLPSVNKDVIEKFFAKFGGITSAAACKDKSGRVFAFCNFEKHDDAVKAIEAMHDHHIDGITAPG

*L. arabica*  HAS-EDKKALYVANFIRRNARLAALVANFTNVYIKQVLPTVNKDVIEKFFAKFGGITSAAACKDKSGRVFAFCNFKKHDDAVKAVEAMHDHHIDGITAPG

*L. aethiopica*  HAS-EDKKALYVANFIRRNARLAALVANFTNVYIKQVLPTVNKEVIEKFFAKFGGITSAAACKDKSGRVFAFCNFEKHDDAVKAVEAMHDHHIDGITAPG

*L. donovani*  HAS-EDKKALYVANFIRRNARLAALVANFTNVYIKQVLPTVNKEVIEKFFAKFGGITSAAACKDKSGRVFAFCNFEKHDDAVKAVEAMHDHHIDGITAPG

*L. gerbilli*  HAS-EDKKALYVANFIRRNARLAALVANFTNVYIKQVLPTVNKDVIEKFFAKFGGITSAAACKDKSGRVFAFCNFEKHDDAVKAVEAMHDHHIDGITAPG

*L. enriettii*  HAS-EDKKALYVANFIRRNARLAALVANFTNVYIKQVLPTVNKEVIEKFFAKFGGITSAAACKDKNGRVFAFCNFEKHDDAVKAVEAMHDHHIDGITAPG

*L. infantum*  HAS-EDKKALYVANFIRRNARLAALVANFTNVYIKQVLPTVNKEVIEKFFAKFGGITSAAACKDKSGRVFAFCNFEKHDDAVKAVEAMHDHHIDGITAPG

*L. panamensis*  HAS-EDKKALYVANFIRRNARLAALVANFTNVYIKQVLPTVNKEVIENFFAKFGGITSAAACKDKSGRVFAFCNFEKHDDAVKAVEAMHDHHIDGITAPG

*L. turanica*  HAS-EDKKALYVANFIRRNARLAALVANFTNVYIKQVLPTVNKDVIEKFFAKFGGITSAAACKDKSGRVFAFCNFEKHDDAVKAVEAMHDHHIDGITAPG

*L. tropica*  HAS-EDKKALYVANFIRRNARLAALVANFTNVYIKQVLPTVNKEVIEKFFAKFGGITSAAACKDKSGRVFAFCNFEKHDDAVKAVEAMHDHHIDGITAPG

*L. major*  HAS-EDKKALYVANFIRRNARLAALVANFTNVYIKQVLPTVNKDVIEKFFAKFGGITSAAACKDKSGRVFAFCNFEKHDDAVKAVEAMHDHHIDGITAPG

*L. mexicana*  HAS-EDKKALYVANFIRRNARLAALVANFTNVYIKQVLPTVSKEVIEKFFAKFGGITSAAACKDKSGRVFAFCNFEKHDDAVKAVEAMHDHHIDGITAPG

*T. cruzi*  HADEEKKMGLYVANFIRRNARLATLVANFTNVYIKQVLPTVDKEVIERFFSKFGGITSSATCKDKSGRVFAFCNFEKHEDAVKAIEASHEQFVDGVVPPG

*T. brucei brucei*  HAD-ADKAALYVANFIRRNARLAALVANFTNVYIKQLLPTVDKDVIEKFFSKFGGITSAAICKDKNGRAFAFCNFEKHDDAVKAIEASHDQEVEGVTQPG

*T. brucei gambiense* HAD-ADKAALYVANFIRRNARLAALVANFTNVYIKQLLPTVDKDVIEKFFSKFGGITSAAICKDKNGRAFAFCNFEKHDDAVKAIEASHDQEVEGVTQPG

*T. evansi*  HAD-ADKAALYVANFIRRNARLAALVANFTNVYIKQLLPTVDKDVIEKFFSKFGGITSAAICKDKNGRAFAFCNFEKHDDAVKAIEASHDQEVEGVTQPG

*T. congolense*  YAD-EKKTALYVANFIRRNARLAALVANFTNVYIKQILPTVDKAIIEKFFSKFGGITSAAICKDKNGRAFAFCNFEKHDDAVKAIEEFHDHEVEGVTQPG

410 420 430 440 450 460 470 480 490 500

....|....|....|....|....|....|....|....|....|....|....|....|....|....|....|....|....|....|....|....|

jaculum1 EKIYVQRAQPRSERLIALRQKYMQSQSLGNNLYVRNFDPSFTDDDLRELFKEYGDIKSCRVMTDPKGVSRGFGFVSFANAEEANAALREMNGRMLNNKPL

jaculum2 EKLYVQRAQPRSERLIALRQKYLQSQSLGNNLYVRNFDLSFTDDNLLELFKEYGEVKSCRVMTDANGVSRGFGFVSFANAEQANAALREMNGRMLNNKPL

*C. fasciculata* EKLYVQRAQPRSERLIGLRQKYMQHQSLGNNLYVRNFDPEFTDADLLELFKEYGNVKSCRVMMSESGASRGFGFVSFSDADEANAALREMNGRMLNGKPL

*L. pyrrhocoris*  EKLYVQRAQPRSERLIALRQKYMQHQSLGNNLYVRNFDPEFTDADLLELFKEYGNVKSCRVMVSENGASRGFGFVSFTDADEANAALREMNGRMLNGKPL

*L. seymouri*  EKLYVQRAQPRSERLIALRQKYMQHQSLGNNLYVRNFDAEFTDADLLELFKEYGNVKSCRVMVSENGASRGFGFVSFSDADEANAALREMNGRMLNGKPL

*L. arabica*  EKLYVQRAQPRSERLIALRQKYMQHQALGNNLYVRNFDPEFTGADLLELFKEYGEVKSCRVMVSESGVSRGFGFVSFSNADEANAALREMNGRMLNGKPL

*L. aethiopica*  EKLYVQRAQPRSERLIALRQKYMQHQALGNNLYVRNFDPEFTGADLLELFKEYGEVKSCRVMVSESGASRGFGFVSFSNADEANAALREMNGRMLNGKPL

*L. donovani*  EKLYVQRAQPRSERLIALRQKYMQHQSLGNNLYVRNFDPEFTGADLLELFKEYGEVKSCRVMVSESGASRGFGFVSFSNADEANAALREMNGRMLNGKPL

*L. gerbilli*  EKLYVQRAQPRSERLIALRQKYMQHQALGNNLYVRNFDPEFTGADLLELFKEYGEVKSCRVMVSESGVSRGFGFVSFSNADEANAALREMNGRMLNGKPL

*L. enriettii*  EKLYVQRAQPRSERLIALRQKYMQHQSLGNNLYVRNFDAEFTSADLLELFKEYGEIKSCRVMMSESGASRGFGFVSFSNADEANAALREMNGRMLNGKPL

*L. infantum*  EKLYVQRAQPRSERLIALRQKYMQHQSLGNNLYVRNFDPEFTGADLLELFKEYGEVKSCRVMVSESGASRGFGFVSFSNADEANAALREMNGRMLNGKPL

*L. panamensis*  EKLYVQRAQPRSERLIALRQKYMQHQSLGNNLYVRNFDPEFTGADLLELFKEYGDVKSCRVMMSESGASRGFGFVSFSNADEANAALREMNGRMLNGKPL

*L. turanica*  EKLYVQRAQPRSERLIALRQKYMQHQALGNNLYVRNFDPEFTGADLLELFKEYGEVKSCRVMVSESGVSRGFGFVSFSNADEANAALREMNGRMLNGKPL

*L. tropica*  EKLYVQRAQPRSERLIALRQKYMQHQALGNNLYVRNFDPEFTGADLLELFKEYGEVKSCRVMVSESGASRGFGFVSFSNADEANAALREMNGRMLNGKPL

*L. major*  EKLYVQRAQPRSERLIALRQKYMQHQALGNNLYVRNFDPEFTGADLLELFKEYGEVKSCRVMVSESGVSRGFGFVSFSNADEANAALREMNGRMLNGKPL

*L. mexicana*  EKLYVQRAQPRSERLIALRQKYMQHQSLGNNLYVRNFDPEFTGADLLELFKEYGDVKSCRVMVSESGASRGFGFVSFSNADEANAALREMNGRMLNGKPL

*T. cruzi*  EKLYVQRAQPRSERLIALRQKYMQCQTLGNNLYVRNFDPEFTEENLHELFKEYGVIRSCRVMTDANGNSRGFGFVSFENADQANAALREMNGRMLNGKPL

*T. brucei brucei*  EKLYVQRAQPRSERLIALRQKYMQCQSLGNNLYVRNFDAEFTEKDLNELFKEYGVIRSCRVMTDANGISRGFGFVSFENADQANAALREMNGRMLNGKPL

*T. brucei gambiense* EKLYVQRAQPRSERLIALRQKYMQCQSLGNNLYVRNFDAEFTEKDLNELFKEYGVIRSCRVMTDANGISRGFGFVSFENADQANAALREMNGRMLNGKPL

*T. evansi*  EKLYVQRAQPRSERLIALRQKYMQCQSLGNNLYVRNFDAEFTEKDLNELFKEYGVIRSCRVMTDANGISRGFGFVSFENADQANAALREMNGRMLNGKPL

*T. congolense*  EKLYVQRAQPRSERLIALRQKYMQCQSLGNNLYVRNFDPEFTEKDLNELFKEYGVIRSCRVMTDANGVSRGFGFVSFENADQANAALREMSGRMLNGKPL

510 520 530 540 550 560 570 580 590 600

....|....|....|....|....|....|....|....|....|....|....|....|....|....|....|....|....|....|....|....|

jaculum1 IVNIAQRRDQRYTMLRMQFQQRLHLMMRQLQMQQQQLRQQQQQQQRMPFMA---------------------GGAGL--------MPSVMRSAGGMLPVQ

jaculum2 IVNIAQRRDQRYTMLRMQFQQRLHLMMKQMQQQQQQQQQQHQFQQQSQLQGQANFSSTTAPQRAGRGRGGR-SGAGLQNRPMNFAMPLPLHPAGMMPPI-

*C. fasciculata* IVNIAQRRDQRYTMLRMQFQQRLQMMMRQMH-------------QQVPFIG-------------------G-QG--------------------------

*L. pyrrhocoris*  IVNIAQRRDQRYTMLRMQFQQRLQMMMRQMH-------------QQMPFVG-------------------GQQG--------------------------

*L. seymouri*  IVNIAQRRDQRYTMLRMQFQQRLQMMMRQMH-------------QQMPFVG-------------------DQQG--------------------------

*L. arabica*  IVNIAQRRDQRYTMLRLQFQQRLQMMMRQMH-------------QPMPFVG-------------------G-QG--------------------------

*L. aethiopica*  IVNIAQRRDQRYTMLRLQFQQRLQMMMRQMH-------------QPMPFVG-------------------G-QG--------------------------

*L. donovani*  IVNIAQRRDQRYTMLRLQFQQRLQMMMRQMH-------------QPMPFVG-------------------G-QG--------------------------

*L. gerbilli*  IVNIAQRRDQRYTMLRLQFQQRLQMMMRQMH-------------QPMPFVG-------------------G-QG--------------------------

*L. enriettii*  IVNIAQRRDQRYTMLRLQFQQRLQMMMRQMH-------------QPMPFVG-------------------G-QV--------------------------

*L. infantum*  IVNIAQRRDQRYTMLRLQFQQRLQMMMRQMH-------------QPMPFVG-------------------G-QG--------------------------

*L. panamensis*  IVNIAQRRDQRYTMLRLQFQQRLQMMMRQMH-------------QPMPFVG-------------------G-QG--------------------------

*L. turanica*  IVNIAQRRDQRYTMLRLQFQQRLQMMMRQMH-------------QPMPFVG-------------------G-QG--------------------------

*L. tropica*  IVNIAQRRDQRYTMLRLQFQQRLQMMMRQMH-------------QPMPFVG-------------------G-QG--------------------------

*L. major*  IVNIAQRRDQRYTMLRLQFQQRLQMMMRQMH-------------QPMPFVG-------------------S-QG--------------------------

*L. mexicana*  IVNIAQRRDQRYTMLRLQFQQRLQMMMRTMH-------------QPMPFVG-------------------G-QG--------------------------

*T. cruzi*  IVNIAQRRDQRFMMLRLQFQQRLQAMMRRM--------------HSMPFAS---------------------HG--------------------------

*T. brucei brucei*  VVNIAQRRDQRLTMLKLQLQQRLQMMMHHM--------------HPPPFGM---------------------PG--------------------------

*T. brucei gambiense* VVNIAQRRDQRLTMLKLQLQQRLQMMMHHM--------------HPPPFGM---------------------PG--------------------------

*T. evansi*  VVNIAQRRDQRLTMLKLQLQQRLQMMMHHM--------------HPPPFGM---------------------PG--------------------------

*T. congolense*  VVNIAQRRDQRFTMLRLQLQQRLQMMMRQI--------------HPPPFGM---------------------PR--------------------------

610 620 630 640 650 660 670 680 690 700

....|....|....|....|....|....|....|....|....|....|....|....|....|....|....|....|....|....|....|....|

jaculum1 QPQQQQQQQPAHAASSRPTRGRAGRAHGGAGRGVGSS-SNSNNVAVVPPPQQQQQ--Q--PLLA-------MPAPPPLPAMLHSSTTTT-TASAPTTTAA

jaculum2 ---NFSQPPSANASSAQLAGGQ-----------------AGN-MMMMMPPMQPPM--M--PSAMPNTQMP-LSHPTLLP------TQQV-MMYAPSTMAT

*C. fasciculata* -----------RPMRGRGGRQQ-----------------HGGRAQGQPMPMPSPQ--Q--PQQPQQQQ-------QATP------QQPL-GFATPAKSG-

*L. pyrrhocoris*  -----------RPMRGRGGRQQ-----------------HGGRAQGHPMPMPSPQ--QQQQQQPQ-----------VAP------QQQQIGFATPAKSG-

*L. seymouri*  -----------RPMRGRGGRQQ-----------------HGGRAQGHPMPMPSPQ--Q--PQQPH-----------ASP------PQQP-GFATPAKSG-

*L. arabica*  -----------RPMRGRGGRQQ-----------------LGGRAQGHPMPMPSPQQPQ--P---------------QAP------AQPQ-GFATPSAVG-

*L. aethiopica*  -----------RPMRGRGGRQQ-----------------LGGRAQGHPMPMPSPQ--Q--P---------------QAP------AQPQ-GFATPSAVG-

*L. donovani*  -----------RPMRGRGGRQQ-----------------LGGRAQGHPMPMPSPQ--Q--P---------------QAP------AQPQ-GFATPSAVG-

*L. gerbilli*  -----------RPMRGRGGRQQ-----------------LGGRAQGHPMPMPSPQQPQ--P---------------QAP------AQPQ-GFATPSAVG-

*L. enriettii*  -----------RPMRGRGGRQQ-----------------QGGRAQGQPMPMSSPQ--Q--P---------------LAP------AQPQ-GFATPSAVG-

*L. infantum*  -----------RPMRGRGGRQQ-----------------LGGRAQGHPMPMPSPQ--Q--P---------------QAP------AQPQ-GFATPSAVG-

*L. panamensis*  -----------RPMRGRGGRQQ-----------------QGGRAQGHPMPMPSPQ-------------------------------QSQ-GFATPSAVG-

*L. turanica*  -----------RPMRGRGGRQQ-----------------LGGRAQGHPMPMPSPQQPQ--P---------------QAP------AQPQ-GFATPSAVG-

*L. tropica*  -----------RPMRGRGGRQQ-----------------LGGRAQGHPMPMPSPQ--Q--P---------------QAP------AQPQ-GFATPSAVG-

*L. major*  -----------RPMRGRGGRQQ-----------------LGGRAQGHPMPMPSPQ--Q--P---------------QAP------AQPQ-GFATPSAVG-

*L. mexicana*  -----------RPMRGRGGRQQ-----------------LGGRAQGHPMPMPSPQ--Q--PQAPV-----------QAP------VQPQ-GFATPSAVG-

*T. cruzi*  -----------HLPQRRNARGTQR------GGGG---GGGGAGRIQAPPPMPPPV--Q-----------------------------QD-MFATPSMAF-

*T. brucei brucei*  -----------HPLQRRNARSG-----------------GRGNR-----PHPRQQ--Q--PQPQPEPQPPLATTVPPAA------AHSV-MFTAPSMGF-

*T. brucei gambiense* -----------HPLQRRNARSG-----------------GRGNR-----PHPRQQ--Q--PQPQPEPQPPLATTVPPAA------AHSV-MFTAPSMGF-

*T. evansi*  -----------HPLQRRNARSG-----------------GRGNR-----PHPRQQ--Q--PQPQPEPQPPLATTVPPAA------AHSV-MFTAPSMGF-

*T. congolense*  -----------QMPQRRRGRQ-----------------------------HPRGH--D---------QMPSLPVMPPVI------PRND-MFNTPSMGV-

710 720 730 740 750 760 770 780 790 800

....|....|....|....|....|....|....|....|....|....|....|....|....|....|....|....|....|....|....|....|

jaculum1 TPSPAQQPTAAPAVTSTAATTSPQQQPTPATRPVQATPRQSPAQAPDTPPLPPISLRELDGMSHDEQRAALGDRLFIKVCEVVPEYAPKITGMFLEMDYK

jaculum2 PSAIPVTSQEMPAVHTTSSAAIPATSTTPVAHPT-PTPRLSPGQAPETPPLPPITMQELDTMSRDEQRSALGDRLFIKICEIAPEYAPKITGMILEMDYK

*C. fasciculata* ---------FVS-----------------------ATPKQSPGQAPDTPPLPPITPQELDSMSPQEQRAALGDRLFLKVYDIAPELAPKITGMFLEMELM

*L. pyrrhocoris*  ---------FVS-----------------------ATPKHSPGQAPETPPLPPITPQELESMSPQEQRAALGDRLFLKVYDIAPELAPKITGMFLEMKFK

*L. seymouri*  ---------FVS-----------------------ATPKHSPGQAPETPPLPPITPQELDSMSPQEQRAALGDRLFLKVYDIVPELAPKITGMFLEMKLK

*L. arabica*  ---------FVQ-----------------------ATPKHSPGDVPETPPLPPITPQELESMSPQEQRAALGDRLFLKVYEIAPELAPKITGMFLEMKPK

*L. aethiopica*  ---------FVQ-----------------------ATPKHSPGDVPETPPLPPITPQELESMSPQEQRAALGDRLFLKVYEIAPELAPKITGMFLEMKPK

*L. donovani*  ---------FVQ-----------------------ATPKHSPGDVPETPPLPPITPQELESMSPQEQRAALGDRLFLKVYEIAPELAPKITGMFLEMKPK

*L. gerbilli*  ---------FVQ-----------------------ATPKHSPGDVPETPPLPPITPQELESMSPQEQRAALGDRLFLKVYEIAPELAPKITGMFLEMKPK

*L. enriettii*  ---------FVQ-----------------------ATPKHSPGQAPETPPLPPITPQELESMSPQEQRAALGDRLFLKVYDIAPELAPKITGMFLEMNPK

*L. infantum*  ---------FVQ-----------------------ATPKHSPGDVPETPPLPPITPQELESMSPQEQRAALGDRLFLKVYEIAPELAPKITGMFLEMKPK

*L. panamensis*  ---------FVQ-----------------------ATPKHSPGQVPETPPLPPITPQELESMSPQEQRAALGDRLFLKVYEIAPELAPKITGMFLEMNPK

*L. turanica*  ---------FVQ-----------------------ATPKHSPGDVPETPPLPPITPQELESMSPQEQRAALGDRLFLKVYEIAPELAPKITGMFLEMKPK

*L. tropica*  ---------FVQ-----------------------ATPKHSPGDVPETPPLPPITPQELESMSPQEQRAALGDRLFLKVYEIAPELAPKITGMFLEMKPK

*L. major*  ---------FVQ-----------------------ATPKHSPGDVPETPPLPPITPQELESMSPQEQRAALGDRLFLKVYEIAPELAPKITGMFLEMKPK

*L. mexicana*  ---------FVQ-----------------------ATPKHSPGDAPETPPLPPITPQELESMSPQEQRAALGDRLFLKVYEIAPELAPKITGMFLEMKPK

*T. cruzi*  ---------APP-----------------------RTPQPSPGVAPDTPPLPPITAEDLRSMSVDEQRAALGDRLYIKVFEIAPDHAPKITGMFLEMDLK

*T. brucei brucei*  --------SAVP-----------------------RTPQASPAIAPDTPPLPPISAEDLQQMSVDEQRAALGDRLYIKVHELAPDHAPKITGMFLEMNPK

*T. brucei gambiense* --------SAVP-----------------------RTPQASPAIAPDTPPLPPISAEDLQQMSVDEQRAALGDRLYIKVHELAPDHAPKITGMFLEMNPK

*T. evansi*  --------SAVP-----------------------RTPQASPAIAPDTPPLPPISAEDLQQMSVDEQRAALGDRLYIKVHELAPDHAPKITGMFLEMNPK

*T. congolense*  --------SAAP-----------------------RTPQASPATAPDTPPLPPITAEDLQNMTMEEQRAALGDRLYIKVYELAPDHAPKITGMFLEMNPK

810 820 830

....|....|....|....|....|....|.

jaculum1 DAYELLGNQKLLMDRITEAMCVLKAHDRGAQ

jaculum2 DAFDLLNDNKELLARVTEALCVLKAHDRPK-

*C. fasciculata* DAYELLNDQKRLEDRVTEALCVLKAHQTA--

*L. pyrrhocoris*  DAYELLNDQKRLEDRVTEALCVLQAHQTA--

*L. seymouri*  DAYDLLNDQKRLEDRVTEALCVLKAHQTS--

*L. arabica*  EAYELLNDQKRLEERVTEALCVLKAHQTA--

*L. aethiopica*  EAYELLNDQKRLEERVTEALCVLKAHQTA--

*L. donovani*  EAYELLNDQKRLEERVTEALCVLKAHQTA--

*L. gerbilli*  EAYELLNDQKRLEERVTEALCVLKAHQTA--

*L. enriettii*  EAYELLNDQKRLEDRVTEALCVLKAHQTA--

*L. infantum*  EAYELLNDQKRLEERVTEALCVLKAHQTA--

*L. panamensis*  EAYELLNDQKRLEERVTEALCVLKAHQTV--

*L. turanica*  EAYELLNDQKRLEERVTEALCVLKAHQTA--

*L. tropica*  EAYELLNDQKRLEERVTEALCVLKAHQTA--

*L. major*  EAYELLNDQKRLEERVTEALCVLKAHQTA--

*L. mexicana*  EAYELLNDQKRLEDRVTEALCVLKAHQTT--

*T. cruzi*  EAFTLLTNQRLLQEKVIEALCVLKAHESSA-

*T. brucei brucei*  EALALLSNPKLMHEKVTEALCVLKVHASSA-

*T. brucei gambiense* EALALLSNPKLMHEKVTEALCVLKVHASSA-

*T. evansi*  EALALLSNPKLMHEKVTEALCVLKVHASSA-

*T. congolense*  EAHTLLTNQRLLQDKVTEALCVLKVHASNS-

**B) DNA polymerase delta subunit 2**

10 20 30 40 50 60 70 80 90 100

....|....|....|....|....|....|....|....|....|....|....|....|....|....|....|....|....|....|....|....|

jaculum1 MT---------ESASDAQTRSACDIVRTHQRFLLRRLEFAQQYAPMYRNRIEAQHARALHAIREAVA-----SSSSSS-VQQQQQL--------------

jaculum2 MTN-------NNINNEKVVRASCEITRTHQRFLLRRLEFSQQYAPMYRNRIEAQFDAVLEAIQSCIAFDKELNKNSNN-EHVKSKLTEETEECYNCYDNE

*C. fasciculata*  M------------SSSVEARDACPIVRTHQRFLLRSLEFTQQYAPMYRCRLEAQHSSALRAIQRVAG-----EYEASSGSRLKS----------------

*L. pyrrhocoris*  M------------PSAVETRDACPIVRTHQRFLLRSLEFTQQYAPMYRCRLEAQYTPAIRAIQHIVT-----QHAANGVSSAGL----------------

*L. seymouri*  M------------PCGAEARDACPIVRTHQRFLLRSLEFTQQYAPMYRCRLEAQYTPALQAIQRIAA-----EHASSSVRGAGS----------------

*L. arabica*  MSNNGS------AAAAVETRSACPITRTHQRFLLRSLEFTQQYAPMYRCRMEAQYTSALHAIQRIAR-----EDPMYG-PEAGA----------------

*L. aethiopica*  MINNGS------AAAAVETRSACPILRTHQRFLLRSLEFTQQYAPMYRCRMGAQYTSALHAIQRILR-----EDPMYG-PEAGA----------------

*L. donovani*  MISNGS------AAAAVETRSACPITRTHQRFLLRSLEFTQQYAPMYRCRMGAQYTSALHAIQRIVR-----EDPMYG-PEAGA----------------

*L. gerbilli*  MSNNGS------AAAAVETRSACPITRTHQRFLLRSLEFTQQYAPMYRCRMGAQYTSALHAIQRIVR-----EDPMYG-PEAGA----------------

*L. enriettii*  MASNG-------SGAAVEARSACPITRTHQRFLLRSLEFTQQYAPMYRCRMEAQYPSALRAVQHVVG-----KDPRYS-PHACA----------------

*L. infantum*  MISNGS------AAAAVETRSACPITRTHQRFLLRSLEFTQQYAPMYRCRMGAQYTSALHAIQRIVR-----EDPMYG-PEAGA----------------

*L. panamensis*  MISNGSGTTAAAAAAGAETRSACPITRTHQRFLLRSLEFTQQYAPMYRCRMEAQYASALRAIQRIVR-----EDPRYG-SQAYV----------------

*L. turanica*  MSNNGS------AAVAVETRSACPITRTHQRFLLRSLEFTQQYAPMYRCRMGAQYKSALHAIQRIVR-----EDPMYG-PEAGA----------------

*L. tropica*  MINNGS------AAAAVETRSACPIIRTHQRFLLRSLEFTQQYAPMYRCRMGAQYTSALHAIQRIVR-----EDPMYG-PEAGA----------------

*L. major*  MSHSGS------AAAAVETRSACPITRTHQRFLLRSLEFTQQYAPMYRCRMGAQYTSALQAIQRLVR-----ADPMYG-PEAGA----------------

*L. mexicana*  MTSNGS------SAVAVETRSACPIVRTHQRFLLCSLEFTQQYAPMYRCRMGAQYASALHAIQRIVR-----EDPMYG-PEAGV----------------

*T. cruzi*  ---------------------------------------------MYQCRERALQPRALRAIRTKID-----VHLSTG-SSA------------------

*T. brucei brucei*  MSEALID---EENFVPPTARVGLSIVRTHRRFLLRHRHFVHQYAAMYRARLEALERRVLKAVSAKVA------AGNVV-GTF------------------

*T. brucei gambiense* MSEALID---EENFVPPTARVGLPIVRTHHRFLLRHRHFVHQYAAMYRARLEALERRVLKAVSAKVA------AGNVV-GTF------------------

*T. evansi*  MSEALID---EENFVPPTARVGLPIVRTHHRFLLRHRHFVHQYAAMYRARLEALERRVLKAVSAKVA------AGNVV-GTF------------------

*T. congolense*  MTKTLAG---TGSVDQPQKRAELPIRPTHQRFLLRQRHFTHQYAAMYSARLQALEERAFQAIRAKVL------DATGD-GSL------------------

110 120 130 140 150 160 170 180 190 200

....|....|....|....|....|....|....|....|....|....|....|....|....|....|....|....|....|....|....|....|

jaculum1 --PGLLRVLELVPGVASICVGVVYKHMKLLPQFLDEYQRELVRVDAGGDDDDDE-DGDGGGGGDDGGGA-AAVDATVSTSPGDATAA---EAAQDMSMAA

jaculum2 LSPSLSRVLELVPGHPAICVGVLYKHMKLLPKFLDEYQRELIRINAGGDDDENE-ENDNTAAGIEMTEIIATEREEKLAAKEDATGLELDSQEAGLELAA

*C. fasciculata*  --LEPRRVLELQPGVPALCVGVVYKNMKMLPRFLDEYQRELVRIDAGDDDNDDDGGGAVDSAPMD-TE--DVAVGLTN----EEGDL----PNGGQTLAA

*L. pyrrhocoris*  --LDPRRVLELQPGVPAICVGVVYKNMKMLPRFLDEYQRELVRIDAGDEDNDDDGGGAVESVPLD-AK--DAVSGMMN----EEGEL----SNGGQALAA

*L. seymouri*  --LEPRRVLELQRDVPAVCVGVVYKSMKMLPRFLDEYQRELVRIDAGDEDNDDDGGGAPEPVPLD-TQ--GAVVGMMD----EEGEA----SNGNQALAA

*L. arabica*  --MKPRRVLELQPGVPAVCVGIVYKNMKLLPRFLDEYQSELVRIDAGDDDNDEE-SGVVEAAPLDRSE--EAAAAAMHRGNDAEEDV----TNDGQTLAA

*L. aethiopica*  --MKPRRVLELQPGVPAVCVGIVYKNMKLLPRFLDEYQSELVRIDAGDDDNDEE-SGVVEAAPLDRPE--DAAAAAMHCGNEAEEDV----SNDGQTLAA

*L. donovani*  --MKPRRVLELQPGMPAVCVGIVYKNMKLLPRFLDEYQSELVRIDAG-DDNDEE-SGVVEAAPPERSE--DAAAAAMRLGNEAEEDA----SNDGQTLAA

*L. gerbilli*  --MKPRRVLELQPGVPAVCVGIVYKNMKLLPRFLDEYQSELVRIDAGDDDNGEE-SGVVEAAPLDRSE--EAAAAAMHRGNDAEEDV----SNDGQTLAA

*L. enriettii*  --TTPRRVLELQPGVPAMCVGILYKNMKLLPRFLDEYQSELVRIDAGDDDNDEE-SGAVEAAPLGRSA--GAAASTVRRNNEEEEDM----SNDGQALAA

*L. infantum*  --MKPRRVLELQPGMPAVCVGIVYKNMKLLPRFLDEYQSELVRIDAG-DDNDEE-SGVVEAAPPERSE--DAAAAAMRLGNEAEEDA----SNDGQTLAA

*L. panamensis*  --MNPRRVLELQPGVPAVCVGIVYKNMKLLPRFLDEYQSELVRIDAGDDDNDDE-SGVVEAVPLDRSA--ETAAATMHRSNEAEEDA----NNDGQALAA

*L. turanica*  --MKPRRVLELQPGVPAVCVGIVYKNMKLLPRFLDEYQSELVRIDAGDDDNDGE-SGVVEAAPLDRSE--EAAAAAMHRGNDAEEDV----SNDGQTLAA

*L. tropica*  --MKPRRVLELQPGVPAVCVGIVYKNMKLLPRFLDEYQSELVRIDAGDDDNDEE-SGVVEAAPLDRPE--DAAAAAMHRGNEAEEDV----SNDGQTLAA

*L. major*  --MKPRRVLELQPGVPAVCVGIVYKNMKLLPRFLDEYQSELVRIDAGDDDNDEE-SGVVEAAPLDRSE--EAAAAAMHRGNDAEEDV----SNDGQTLAA

*L. mexicana*  --MKPWRVLELQPGVPAVCVGIVYKNMKLLPRFLDEYQSELVRIDAGDDDNDEV-SGVVETAPLDRSG--DAAAAVMHRGNEAEEDV----SNDGQTLAA

*T. cruzi*  --PPFARVLDLVPGQRALCVGVIYKQMRLLPRFLDEYQKELIRIDAGEDDDEES-GAADALCDTTPPL--ASERVATINGDGEIGGG-RHAEWQGGRGES

*T. brucei brucei*  --PQRSRVLELGIGERALCVGVAYKEARLLPRFLDEYQKELVRIDAGEEEDSYG-GSEAVSCSVPLRA--NSRGVNPTPGNCCEGDA-----------RN

*T. brucei gambiense* --PQRSRVLELGIGERALCVGVAYKEARLLPRFLDEYQKELVRIDAGEEEDSYG-GSEAVSCSVPLRA--NSRGINPTPGTCCEGDA-----------RN

*T. evansi*  --PQRSRVLELGIGERALCVGVAYKEARLLPRFLDEYQKELVRIDAGEEEDSYG-GSEAVSCSVPLRA--NSRGINPTPGTCCEGDA-----------RN

*T. congolense*  --PPHTRVLELVPHQRAICTGIIYKQSRLLPRFLDEYQKELVRIDAGGEEDGEA-MVEGPLCPDPFIV--RESAVNLTFANGSDGDL-----------RS

210 220 230 240 250 260 270 280 290 300

....|....|....|....|....|....|....|....|....|....|....|....|....|....|....|....|....|....|....|....|

jaculum1 VHEHYTLCNAADELTLEDSSGRILLHGLDTSRFCTGVVLGVYGTLRDNGSLLVHQYAFS-GLDELH------VPRRLLS---------------------

jaculum2 SNEHYTLCHSNDELLLEDSSGRIVLVGLDPTRFCTGLVIGLCGTLETNGRLRVHRYAFA-HSLPVHYNNSNTIPRLLHTPQAQIRIHDGNDNNNNNNNAS

*C. fasciculata*  ANEHYSICNAADELTLEDSSGRVLLHGLHADRFCTGVVLGIYGVLHANGSVEVIRYVFAGDLRGSY------VPRSL-----------------------

*L. pyrrhocoris*  ENEQYSVCNSADELMLEDSSGRVLLQGLNPERFCTGVVLGIYGNLLANGSIAVIRYAFAGDLRSSY------VPRSL-----------------------

*L. seymouri*  ADEHYSLSNSADELMLEDSSGRVLLQGLNPERFCTGVVLGIYGVLHANGSIEVIRYAFAGDLRASY------VPRPV-----------------------

*L. arabica*  ADEHYSVCNSADELMLEDSSGRVLLQGLDAERFCTGVVLGVYGTLLPNGSIRVLRYAFSGDLRFMF------VPRPL-----------------------

*L. aethiopica*  ADEHYSVCDSADELMLEDSSGRVLLQGLDSERFCTGVVLGVYGTLLPNGSIKVLRYAFSGDLRLMF------VPRPL-----------------------

*L. donovani*  ADEHYSVCNSADELMLEDSSGRVLLQGLDAERFCTGVVLGVYGTLLPNGSIKVLRYAFSGDLRFTF------VPRPL-----------------------

*L. gerbilli*  ADEHYSVCNSADELMLEDSSGRVLLQGLDAERFCTGVVLGVYGTLLPNGSIKVLRYAFSGDLRFMF------VPRPL-----------------------

*L. enriettii*  ADEHYTVCSSADELMLEDSSGRVLLQGLDAERFCTGIVLGIYGTLLPNGSVNVLCYAFSGGSRSTF------VPRPL-----------------------

*L. infantum*  ADEHYSVCNSADELMLEDSSGRVLLQGLDAERFCTGVVLGVYGTLLPNGSIKVLRYAFSGDLRFTF------VPRPL-----------------------

*L. panamensis*  ADEHYSVCDSADELMLEDSSGRALLQGLDAERFCTGIVLGVYGALLPNGSMKVLRYAFSGDLGSAF------VPRPV-----------------------

*L. turanica*  ADEHYSVCNSADELMLEDSSGRVLLQGLDAERFCTGVVLGVYGTLLPNGSIKVLRYAFSGDLRFMF------VPRPL-----------------------

*L. tropica*  ADEHYSVCDSADELMLEDSSGRVLLQGLDAERFCTGVVLGVYGTLLPNGSIKVLRYAFSGDLRFMF------VPRPL-----------------------

*L. major*  ADEHYNVCNSADELMLEDSSGRVLLQGLDAERFCTGVVLGVYGTLLSNGSIKVLRYAFSGDLSFMV------VPRPL-----------------------

*L. mexicana*  TDEHYSVCNSADELMLEDSSGRVLLQGLDAERFCTGIVLGVYGTLLPNGCIKVLRYAFSGDLGFTF------VPRPL-----------------------

*T. cruzi*  REQHVPLCHSADEVFMEDDSGRVLLKEVPVDLLCTGLVIGVDGTLRDNGCLFVHSYALT-DLREIY------VPRLL-----------------------

*T. brucei brucei*  QSHLFNICGEDDEVFMEDDSGRVRLEGIPAAVVCTGLVLGVDGTLLENGCLSVNCYAIG-DLREVY------VPRTL-----------------------

*T. brucei gambiense* QSHLFNICGEDDEVFMEDDSGRVRLEGIPAAVVCTGLVLGVDGTLLENGCLSVNCYAIG-DLREVY------VPRTL-----------------------

*T. evansi*  QSHLFNICGEDDEVFMEDDSGRVRLEGIPAAVVCTGLVLGVDGTLLENGCLSVNCYAIG-DLREVY------VPRTL-----------------------

*T. congolense*  QQCFPNICAPDDELYMEDDSGRVRLENIPADMMCTGVVLGVDGTLLESGRLSVHRYALG-DLRKVY------VPRLI-----------------------

310 320 330 340 350 360 370 380 390 400

....|....|....|....|....|....|....|....|....|....|....|....|....|....|....|....|....|....|....|....|

jaculum1 --RHTPPHA--EPCYVAFVCGLQL----GGGGSSSSGG----DETVVD------AQRGRVMLELLLDFVSGNVVDEALFAQARRVARVVVGGNSIAATEE

jaculum2 ERRNSNESH--VPCYIAFVCGLNL----QPWARDRNASSNNEKQNVTNDTHSAHRHRSFMMLRLLVDFICGSVSDDHLLYMARHIARLVIGGNSIAPTDE

*C. fasciculata*  ---PRAASATVAPCYIAFVSGLNINLPRDGSKEEHVA-----------------TAKARASLELLVDFLCGNTSNASLRTKAKHVTRLVIGGDSIAPTDE

*L. pyrrhocoris*  ---AHITDSAIEPCYIAFVSGLSINLPRDGSREEHAA-----------------VAKARASLELLVDFLCGGTSNAALRTKAKHVTRLVIGGDSIAPTDE

*L. seymouri*  ---ARAAGGAIEPCYIAFVSGLNVNLPRDDSKEEHAA-----------------VAKARASLELLVDFLCGNTNNAALHTKAKRISRLVIGGDSIAPTDE

*L. arabica*  ---IRAA----SPCYIAFVSGLNINIPRGSSKEEQAA-----------------AARARASLELLVEFLCGNTGNASLRAKARCVSRLVIGGDSIAPTDE

*L. aethiopica*  ---IRAA----SPCYIAFVSGLNINIPRGSSKEEQAA-----------------AARARASLELLVEFLCGNTVNASLRAKAKCVSRLVIGGDSIAPTDE

*L. donovani*  ---IRAA----SPCYIAFVSGLSINIPRGSSKEEQAA-----------------ATRARASLELLVEFLCGNTGNASLRAKAKCVSRLVIGGDSIAPTDE

*L. gerbilli*  ---IRAA----SPCYIAFVSGLNINIPRGSSKEEQAA-----------------AARARASLELLVEFLCGNTGNTSLRAKAKCVSRLVIGGDSIAPTDE

*L. enriettii*  ---IISA----SPCYIAFVGGLNVNLPRDSSKEAQAA-----------------ASRARASLELLVEFLCGNTGNASLRAKAKCVSRLVIGGDSIAPTDE

*L. infantum*  ---IRAA----SPCYIAFVSGLSINIPRGSSKEEQAA-----------------ATRARASLELLVEFLCGNTGNASLRAKAKCVSRLVIGGDSIAPTDE

*L. panamensis*  ---IRAT----GPCYIAFVSGLSLNVPRDNGKEEQAA-----------------ASRARASLELLVEFLCGNTGNAALRGKAKCVSRLVIGGDSIAPTDE

*L. turanica*  ---IRAA----SPCYIAFVSGLNINIPRGSSKEEQAA-----------------AARARASLELLVEFLCGNTGNASLRAKAKCVSRLVIGGDSIAPTDE

*L. tropica*  ---IRAA----SPCYIAFVSGLNINIPRGSSKEEQAA-----------------AARARASLELLVEFLCGNTVNASLRAKAKCVSRLVIGGDSIAPTDE

*L. major*  ---IRAA----SPCYIAFVSGLNINIPRGSSKEEQAA-----------------AARARASLELLVEFLCGNTGNASLRAKAKCVSRLVIGGDSIAPTDE

*L. mexicana*  ---IRTA----SPCYIAFVSGLNINIPRGSGKEEQAA-----------------GARARASLELLMEFLCGNTGNASLRAKAKCVSRLVIGGDSIAPTDE

*T. cruzi*  ---PGKTRS--LPCYVGIVCGLGL----GSPQMQEAE-----------------VTRSRTMVELLVDYLSGHVGDDAMITQASRIAHLVIAGNSIAPTDE

*T. brucei brucei*  ---PTVSSP--TPCYVGFVCGLEL----CSAQTQTDA-----------------ATNGRTMIELLVDYLSGTLGSTGKVVQPSYISRLVIGGNSIAPTEE

*T. brucei gambiense* ---PTVSSP--MPCYVGFVCGLEL----CSAQTQTDA-----------------ATNGRAMIELLVDYLSGALGSTGKVVQPSYISRLVIGGNSIAPTDE

*T. evansi*  ---PTVSSP--MPCYVGFVCGLEL----CSAQTQTDA-----------------ATNGRAMIELLVDYLSGALGSTGKVVQPSYISRLVIGGNSIAPTDE

*T. congolense*  ---PNVPPR--TPRYIAFVCGLEL----GSPQMQNSAS----------------TACARTMVELLVDYLSGAVGDSAMVAQASCITRLVIGGNSIAPTEE

410 420 430 440 450 460 470 480 490 500

....|....|....|....|....|....|....|....|....|....|....|....|....|....|....|....|....|....|....|....|

jaculum1 QQLKKKVRLEPSDHARLGDDNKTTMSTAAAAAAAASTRNVAAGTAMTGAAGVTHGAVSSSSAAALMRELDAVLARLCATVEVELMPGDTDLSDAFLPQQP

jaculum2 LRLKKKVRLEPADHAKLNEDKLQSSSTSSSPLTGV-----------------------TVTSAELMREFDQVLSTLCQSVEVELMPGDNDMSDAFFPQQP

*C. fasciculata*  LKLKKKVKLDPSDHLRLNDDKAQAGI---------------------------------VTSSALMRELDAVLERLVSAVEVEVMPGDNDMSDAFQPQQP

*L. pyrrhocoris*  LKLKKKVKLDPSDHVRMNDDKAQAGI---------------------------------VTSAALMRELDVVLERVVSSVEVEMMPGDNDMSDAFQPQQP

*L. seymouri*  LKLKKKVKLDPSDHVRLNDDKAQAGI---------------------------------VTSAALMRELDALLERVVSSVEVEMMPGDNDMSDAFQPQQP

*L. arabica*  LKLKKKVKLDPSDHVRLNDDKAQAST---------------------------------VTSAALMRQLDTLLERVVRTVEVELMPGANDMSDAFQPQQP

*L. aethiopica*  LKLKKKVKLDPSDHVRLNDDKAQAST---------------------------------VTSAALMRQLDTLLERVVRTVEVELMPGSNDMSDAFQPQQP

*L. donovani*  LKLKKKVKLDPSDHVRLNDDKAQAST---------------------------------VTSAALMRQLDTLLERVVRTVEVELMPGANDMSDAFQPQQP

*L. gerbilli*  LKLKKKVKLDPSDHVRLNDDKAQAST---------------------------------VTSAALMRQLDTLLERVVRTVEVELMPGANDMSDAFQPQQP

*L. enriettii*  LKLKKKVKLDPSDHVRLNDDKAQVST---------------------------------VTSAALMRQLDAFLERVVRSVEVELMPGANDMSDAFQPQQP

*L. infantum*  LKLKKKVKLDPSDHVRLNDDKAQAST---------------------------------VTSAALMRQLDTLLERVVRTVEVELMPGANDMSDAFQPQQP

*L. panamensis*  LKLKKKVKLDPSDHVRLNDDKAQGNT---------------------------------VTSAALMRQLDTLLERVVRTVEVELMPGANDMSDAFQPQQP

*L. turanica*  LKLKKKVKLDPSDHVRLNDDKAQAST---------------------------------VTSAALMRQLDTLLERVVRTVEVELMPGANDMSDAFQPQQP

*L. tropica*  LKLKKKVKLDPSDHVRLNDDKAQAST---------------------------------VTSAALMRQLDTLLERVVRTVEVELMPGANDMSDAFQPQQP

*L. major*  LKLKKKVKLDPSDHVRLNDDKAQAST---------------------------------VTSAALMRQLDTLLERVVRTVEVELMPGANDMSDAFQPQQP

*L. mexicana*  LKLKKKVKLDPSDHVRLNDDKAQAST---------------------------------VTSAALMRQLDTLLERVVRTVEVELMPGANDMSNAFQPQQP

*T. cruzi*  LRLKKKVKLDPSDHTRLSDEKQEGGA---------------------------------ITSAMLMTEFDAVLTRITETIEVDVMPGDQDVTNAFQPQQP

*T. brucei brucei*  LRLKRKVKLDPSDHVKLSDDKQNNGT---------------------------------VTSASLMRELDAILARLADSIEVELMPGDNDMTNAFHPQQP

*T. brucei gambiense* LRLKRKVKLDPSDHVKLSDDKQNNGT---------------------------------VTSASLMRELDAILARLADSIEVELMPGDNDMTNAFHPQQP

*T. evansi*  LRLKRKVKLDPSDHVKLSDDKQNNGT---------------------------------VTSASLMRELDAILARLADSIEVELMPGDNDMTNAFHPQQP

*T. congolense*  LRLKKKVKLDPSDHVKFGDDKGGGGA---------------------------------VTSAQLMRELDKVLKRIVCSVEVELMPGDNDMTNAFHPQQP

510 520 530 540 550 560 570 580 590 600

....|....|....|....|....|....|....|....|....|....|....|....|....|....|....|....|....|....|....|....|

jaculum1 LHPVLLPRAARLSTLRLVTNPYEFVATPLSRG------GGGGRRDDGSRRSRGGGADDSDDGTSNERKRSDHAAGDEAKDGDDGGG--------------

jaculum2 LHPVLLPHAARMSSLRLVTNPFEFTAHVSKST------PSQAHNITSTDSVDHNNNNKKAETTSISKKVKENNMLDEVKRQHQENEEEEEEEEHLYKEEL

*C. fasciculata*  LHPLLLPKSARHSTLRLVPNPFCFTALPPTASALK---PEDGED------------------------------EESDKKKRKMEA--------------

*L. pyrrhocoris*  LHPVLLPKAARHSTLRLVSNPFCFTALPPACA------WKSGAE------------------------------EGSDKKKHRSEV--------------

*L. seymouri*  LHPVLLPKAARHSTLRLVPNPFCFTALPPAYA------LKGEAG------------------------------DESGRKKHKSEV--------------

*L. arabica*  LHPLLLPKAGKHSTLRLVSNPFCFTAQPPAAATAELE-VRGEER------------------------------VESEAKKHKVEV--------------

*L. aethiopica*  LHPLLLPKAGRHSTLRLVSNPFCFTAQPPAAATAELE-VRSEER------------------------------VESEAKKHKVEV--------------

*L. donovani*  LHPLLLPKAGKHSTLRLVSNPFCFTAQPPAAATAELE-VRSEER------------------------------VGSEAKKHKVEV--------------

*L. gerbilli*  LHPLLLPKAGKHSTLRLVSNPFCFTAQPPAAATAESE-VRGEER------------------------------VESEAKKHKVEV--------------

*L. enriettii*  LHPLLLPRAGKHSTLRLVSNPFCFTAQPPAAATTAFE-VRSEEQ------------------------------VESEAKKHKAEV--------------

*L. infantum*  LHPLLLPKAGKHSTLRLVSNPFCFTAQPPAAATAELE-VRSEER------------------------------VGSEAKKHKVEV--------------

*L. panamensis*  LHPLLLPKAGKHSTLRLVSNPFCFTAQPGAATVATTELLKSEEC------------------------------VESEAKKHKAEV--------------

*L. turanica*  LHPLLLPKAGKHSTLRLVSNPFCFTAQPPAAATAELE-VRGEER------------------------------VESEAKKHKVEV--------------

*L. tropica*  LHPLLLPKAGKHSTLRLVSNPFCFTAQPPAAATAELE-VRSEER------------------------------VESEAKKHKVEV--------------

*L. major*  LHPLLLPKAGKHSTLRLVSNPFCFTAQPPAAASAESE-VRGEER------------------------------VESEAKKHKVEV--------------

*L. mexicana*  LHPLLLPKAGKHSTLRLVSNPFCFTAQPPAAVTAEVE-VRSEEC------------------------------VESEAKKHKAEV--------------

*T. cruzi*  LHPLLLPEAARRSSLKLVTNPYEFHAFPGKKP------TATATA------------------------------ERTATTTTTTTA--------------

*T. brucei brucei*  IHPLLLPEAARRSSIGFVTNPYEFVAFPSDDE------------------------------------------ENHVERSGADAT--------------

*T. brucei gambiense* IHPLLLPEAARRSSIGFVTNPYEFVAFPSDDE------------------------------------------ENHVERSGADAT--------------

*T. evansi*  IHPLLLPEAARRSSIGFVTNPYEFVAFPSDDE------------------------------------------ENHVERSGADAT--------------

*T. congolense*  MHPLLLSESARCSSMRLVTNPYEFVALSSDES------------------------------------------GVDASDGTNTTS--------------

610 620 630 640 650 660 670 680 690 700

....|....|....|....|....|....|....|....|....|....|....|....|....|....|....|....|....|....|....|....|

jaculum1 ------------------SGGVCFFASAGQNVNDVARETRFASRLDAMAMMLQSGCACPTAPNTLFSYPFLHDDPFLFRRAPACFVACDQPRFETCYCTH

jaculum2 EEEEEKGKVKKHKTRRLHKQDVQFMVTSGQNVNDVARETQYATRLETMATMVAAGCMCPTAPNTLFSYPFRDVDPFVMSHLPNCFVACDQPQFETAHYSL

*C. fasciculata*  ------------------VGGVNIFVTSGQNINDVARESRFPTRLDAMAMVVESGCACPTAPNSLFSYPFCKSDPFLFQGTPHCVVACDQPRFETRFATL

*L. pyrrhocoris*  ------------------SDGVNVFVTSGQNINDVARESRFPTRLDTMSLVIESGCACPTAPNTLFSYPFCKRDPFLFERTPHCMVACDQPRFETRFATL

*L. seymouri*  ------------------SDGVSVFVTSGQNINDVARESRFPTRLDTMSLVLESGCACPTAPNTLFSYPFCHDDPFLFQRTPHCVVACDQPRFETRFVTL

*L. arabica*  ------------------ADGVNFFVTSGQNINDVARESRFPTRLDTMCMVVVSGCACPTAPNTLFSYPFCNHDPFLFQHTPHCVVACDQPQFETRYATL

*L. aethiopica*  ------------------ADGVNFFVTSGQNINDVARESRFPTRLDTMCMVVVSGCACPTAPNTLFSYPFCNHDPFLFQHTPHCVVACDQPQFETRYATL

*L. donovani*  ------------------ADGVNFFVTSGQNINDVARESRFPTRLDTMCMVVVSGCACPTAPNTLFSYPFCNHDPFLFQHTPHCVVACDQPQFETRYATL

*L. gerbilli*  ------------------ADGVNFFVTSGQNINDVARESRFPTRLDTMCMVVVSGCACPTAPNTLFSYPFCNHDPFLFQHTPHCVVACDQPQFETRYATL

*L. enriettii*  ------------------AAGVDFFVTSGQNINDVARESRFPTRLDAMCMVVVSGCACPTAPNTLFSYPFRNHDPFLFQNTPHCVVACDQPQFETRYATL

*L. infantum*  ------------------ADGVNFFVTSGQNINDVARESRFPTRLDTMCMVVVSGCACPTAPNTLFSYPFCNHDPFLFQHTPHCVVACDQPQFETRYATL

*L. panamensis*  ------------------AEGVNFFVTSGQNINDVARESRFPTRLDTMCMVVVSGCACPTAPNTLFSYPFCNHDPFLFQNTPHCVVACDQPQFETRYATL

*L. turanica*  ------------------ADGVNFFVTSGQNINDVARESRFPTRLDTMCMVVVSGCACPTAPNTLFSYPFCNHDPFLFQHTPHCVVACDQPKFETRYATL

*L. tropica*  ------------------ADGVNFFVTSGQNINDVARESRFPTRLDTMCMVVVSGCACPTAPNTLFSYPFCNHDPFLFQHTPHCVVACDQPQFETRYATL

*L. major*  ------------------ADGVNFFVTSGQNINDVARESRFPTRLDTMCMVVVSGCACPTAPNTLFSYPFCNHDPFLFQHTPHCVVACDQPQFETRYATL

*L. mexicana*  ------------------ADGVNFFVTSGQNINDVARESRFPTRLDTMCMVVVSGCACPTAPNTLFSYPFCNQDPFLFHHTPHCVVACDQPQFETRYATL

*T. cruzi*  ------------------EGGTIFFISSGSNLNDVSRETRYRTRLDAMSMLLRCGCACPTAPNTLFSYPFKILDPFVFPNAPHCFVCCDQPEMQTRWVPL

*T. brucei brucei*  ------------------EGGTIFFVSSGSNLNCVNRETRFDSRLDAMSLILQSGCACPTAPNTVFSYPFTDTDPFVFPKAPHCFVCCDQPEWETRWEPL

*T. brucei gambiense* ------------------EGGTIFFVSSGSNLNCVNRETRFDSRLDAMSLILQSGCACPTAPNTVFSYPFTDTDPFVFPKAPHCFVCCDQPEWETRWEPL

*T. evansi*  ------------------EGGTIFFVSSGSNLNCVNRETRFDSRLDAMSLILQSGCACPTAPNTVFSYPFTDTDPFVFPKAPHCFVCCDQPEWETRWEPL

*T. congolense*  ------------------KGETLFFVSSGSNIHCVSRETNINSRLDAMSFVLRSGCACPTAPNTLFSYPFKDVDPFVFPQAPHCFVCCDQPEWQTRWEPL

710 720 730 740 750 760 770 780 790 800

....|....|....|....|....|....|....|....|....|....|....|....|....|....|....|....|....|....|....|....|

jaculum1 DELRARTGDYYTHNDNNGDADDGDEEQQQQRQQRTRRGASAARREQHKAMRDTSDNDNKDEEGTAVAASSSARHDGVRLVCVPAFHRTGVLVLVDIHSPT

jaculum2 VELSKQTESYFEKSDSTSSNDNITKANNKQE----------GKTEENIEEETNEEEINRK-KHDYVV--NEEDISGIRLICVPAFYRTGTLVLVDVNSAT

*C. fasciculata*  GELYEETHHHYSAQTTEAPAAVLQKASSAT-------------------------------LNEEDR--FESGEAGVRLVCVPAFTRTGALVLMDVNSPT

*L. pyrrhocoris*  EKMYAETHHSYAGAEAAKEAGKQKTESA----------------------------------NEETE--TEGAGAGVRLICVPSFARSGALVLMDVNSPT

*L. seymouri*  SEMDEEMLRSYAS----AEVGRQKTAHASA-------------------------------QSEESE--LRGTEAGVRLICVPSFARSGSLVLMDVNSPT

*L. arabica*  EELHEETHCSFTGPASLPVRSREKLSAAST-------------------------------KDEKKE--AVRAEAGVRLICVPSFARSGALVLVDVNSPT

*L. aethiopica*  EELHEETHRSFTGPASLSVRSKEKLSAAST-------------------------------KDEENK--AVRAEAGVRLICVPSFARSGALVLVDVNSPT

*L. donovani*  EELHEETHRSFTGPASLSVQSKEKLSAAS--------------------------------KDEENK--AVPAEAGVRLICVPSFARSGALVLVDVNSPT

*L. gerbilli*  EELHEETHRSFTGPASLSVRSKEKLSAAST-------------------------------KDEEKK--AVLAEAGVRLICVPSFARSGALVLVDVNSPT

*L. enriettii*  EELHEETHHCFTGRGASSVQLKSMPSATSA-------------------------------KDEESK--AVRAEAGVRLICVPSFARSGSLVLVDVNSPT

*L. infantum*  EELHEETHRSFTGPASLSVRSKEKLSAAST-------------------------------KDEENK--AVPAEAGVRLICVPSFARSGALVLVDVNSPT

*L. panamensis*  DELHEETHHNFTESASSSPRLKATASAVSA-------------------------------KGDENK--AVLPEAGVRLICVPSFARSGALVLVDVNSPT

*L. turanica*  EELHEETHRSFTGPASLSVRSKEKLSAAST-------------------------------KDEENK--AVRAEAGVRLICVPSFARSGALVLVDVNSPT

*L. tropica*  EELHEETHRSFTGPASLSVRSKEKLSAAST-------------------------------KDEENK--AVRAEAGVRLICVPSFARSGALVLVDVNSPT

*L. major*  EELHEETHHSFTGPASLSARSKEKLSAAST-------------------------------KDEENK--AARAEAGVRLICVPSFARSGALVLVDVNSPT

*L. mexicana*  EELHEETHRSFTAPASLSVRSKEKPSAAFA-------------------------------KDEENK--AVRAEAGVRLICVPSFARSGALVLVDVNSST

*T. cruzi*  EGFNPAGDDTTNEEA------------------------------------------------------PVEDSAGVRLICVPPFSQTGALVLVDVNSPQ

*T. brucei brucei*  ATFDPTRDTNGCGILLNEEM-----------------------------------------EGQSQE--IESDGAGVRLVCVPTFAQTGKLVLVDVNSPT

*T. brucei gambiense* ATFDPTRDTNGCGILLNEEM-----------------------------------------EGQSQE--IESDGAGVRLVCVPTFAQTGKLVLVDVNSPT

*T. evansi*  ATFDPTRDTNGCGILLNEEM-----------------------------------------EGQSQE--IESDGAGVRLVCVPTFAQTGKLVLVDVNSPT

*T. congolense*  ESFSPTYDANTNEIHVNE-------------------------------------------EGGETG--KDETAAGVRLVCVPPFVQTGILVLVDVNSPK

810

....|....|....|.

jaculum1 LETTAIDFSLLH----

jaculum2 LETTMIDFSIL-----

*C. fasciculata*  LETTVVNFAVQ-----

*L. pyrrhocoris*  LETTAVNFTVP-----

*L. seymouri*  LESTVVTFSVQ-----

*L. arabica*  LETSVVTFSVP-----

*L. aethiopica*  LETSVLTFSVP-----

*L. donovani*  LETSVVTFSVP-----

*L. gerbilli*  LETSVVTFSVP-----

*L. enriettii*  LETSVVTFSVP-----

*L. infantum*  LETSVVTFSVP-----

*L. panamensis*  LETSVVSFLVP-----

*L. turanica*  LETSVVTFSVP-----

*L. tropica*  LETSVLTFSVP-----

*L. major*  LETSVVIFSVP-----

*L. mexicana*  LETSVVTFSVP-----

*T. cruzi*  LDTVVVNFTSASHEMP

*T. brucei brucei*  LETTYVDFASGLRNPR

*T. brucei gambiense* LETTYVDFASGLCNPR

*T. evansi*  LETTYVDFASGLCNPR

*T. congolense*  LDTTTVSFMPKL----

**C) Rad50 DNA repair protein**

10 20 30 40 50 60 70 80 90 100

....|....|....|....|....|....|....|....|....|....|....|....|....|....|....|....|....|....|....|....|

jaculum1 MTSIEQLQVCGIRSFNPDPARRQTVTFHKPLTVILGKNGAGKTTIIEALLNACTGAMPPGSGTEKSAFVYDPKVLGEADVKAQIRLIFTGKGGNVMQVIR

jaculum2 MTSIEQLQLCGIRSFDPNPARRQTVTFHKPLTVILGKNGAGKTTIIEALLNACTGAMPPGSGTEKSAFVYDPKVLGESEVKAQIRLIFTGKGGKVMQVIR

*C. fasciculata*  MTSIEKIQLCGVRSFDPNPANQQFIQFQKPLTVILGKNGAGKTTIIEALLNACTGAMPPGSGTEKGSFVYDPKVVGETEVKAQIRLIFTGKGGKLMQVIR

*L. pyrrhocoris*  MTSIEKIQICGVRSFDPNPANQQFIQFQKPLTVILGKNGAGKTTIIEALLNACTGAMPPGSGTEKGSFVYDPKVVGETEVKAQIRLIFTGKGGKLMQVIR

*L. seymouri*  MTSVEKIQLCGVRSFDPNPANQQFIQFQKPLTVILGKNGAGKTTIIEALLNACTGAMPPGSGTEKGSFVYDPKVVGETEVKAQIRLIFTGKGGKLMQVIR

*L. arabica*  MTSIEKLQLCGVRSFDPNPANQQFIQFQKPLTVILGKNGAGKTTIIEALLNACTGAMPPGSGTERGSFVYDPKVVGETEVKAQIRLIFTGKGGKLMQVIR

*L. aethiopica*  MTSIEKLQLCGVRSFDPNPANQQFIQFQKPLTVILGKNGAGKTTIIEALLNACTGAMPPGSGTERGSFVYDPKVVGETEVKAQIRLIFTGKGGKLMQVIR

*L. donovani*  MTSIEKLQLCGVRSFDPNPANQQFIQFQKPLTVILGKNGAGKTTIIEALLNACTGTMPPGTGTEKGSFVYDPKVVGETEVKAQIRLIFTGKGGKLMQVIR

*L. gerbilli*  MTSIEKLQLCGVRSFDPNPANQQFIQFQKPLTVILGKNGAGKTTIIEALLNACTGAMPPGSGTERGSFVYDPKVVGETEVKAQIRLIFTGKGGKLMQVIR

*L. enriettii*  MTSIEKLQLCGVRSFDPNPTNQQFIQFQKPLTVILGKNGAGKTTIIEALLNACTGAMPPGSGTEKGSFVYDPKIVGETEVKAQIRLIFVGKGGKLMQVIR

*L. infantum*  MTSIEKLQLCGVRSFDPNPANQQFIQFQKPLTVILGKNGAGKTTIIEALLNACTGTMPPGTGTEKGSFVYDPKVVGETEVKAQIRLIFTGKGGKLMQVIR

*L. panamensis*  MTSIEKLQLCGVRSFDPNPANQQFIQFQKPLTVILGKNGAGKTTIIEALLNACTGAMPPGSGAEKGSFVYDPKVVGETEVKAQIRLIFTGKGSKVMQVIR

*L. turanica*  MTSIEKLQLCGVRSFDPNPANQQFIQFQKPLTVILGKNGAGKTTIIEALLNACTGAMPPGSGTERGSFVYDPKVVGETEVKAQIRLIFTGKGGKLMQVIR

*L. tropica*  MTSIEKLQLCGVRSFDPNPANQQFIQFQKPLTVILGKNGAGKTTIIEALLNACTGAMPPGSGAERGSFVYDPKVVGETEVKAQIRLIFTGKGGKLMQVIR

*L. major*  MTSIEKLQLCGVRSFDPNPTNQQFIQFQKPLTVILGKNGAGKTTIIEALLNACTGAMPPGSGTERGSFVYDPKVVGETEVKAQIRLIFTGKGGKLMQVIR

*L. mexicana*  MTSIEKLQLCGVRSFDPNPANQQFIQFQKPLTVILGKNGAGKTTIIEALLNACTGAMPPGSGTEKGSFVYDPKVVGETEVKAQIRLIFTGKGGKLMQVIR

*T. cruzi*  MTSIEQIQISGVRSFDPNPAHRQTIVFQKPLTVILGKNGAGKTTIIEALLNACTGQMPPGSGSEKSSFVYDPKVMGETDVKAQIRLLFTGRGGKVMQVIR

*T. brucei brucei*  MTSIEQIEISGVRSFDPNPNNRQRIVFKKPLTVILGKNGAGKTTIIEALLNACTGQMPPGGGTEKSSFVYDPKVVGENDVKAQIRLLFTGRGGKVMQVIR

*T. brucei gambiense* MTSIEQIEISGVRSFDPNPNNRQRIVFKKPLTVILGKNGAGKTTIIEALLNACTGQMPPGGGTEKSSFVYDPKVVGENDVKAQIRLLFTGRGGKVMQVIR

*T. evansi*  MTSIEQIEISGVRSFDPNPNNRQRIVFKKPLTVILGKNGAGKTTIIEALLNACTGQMPPGGGTEKSSFVYDPKVVGENDVKAQIRLLFTGRGGKVMQVIR

*T. congolense*  MTSIEQIELSGVRSFDPNPNNRQRITFKKPLTVILGKNGAGKTTIIEALLNACTGQMPPGSGIEKSSFVYDPKVAGEGDVKAQIRLLFTGRGGKVMQVIR

110 120 130 140 150 160 170 180 190 200

....|....|....|....|....|....|....|....|....|....|....|....|....|....|....|....|....|....|....|....|

jaculum1 SFQALRSRSKVTFTTLDNTVAFLDTASGKVVSNTYRASDVDRVVPEMLGVSPAVLEHVIFCHQEDCNWPLGAPKDVKRIFDDIFAATRYVMALDRLRENS

jaculum2 SFQASRSRNKLTFTTLDNTIAFQDAETHKVVSNTYRASDVDRVIPEMLGVSSAVLEHVIFCHQEDCNWPLAPPKDVKRIFDDIFAATRYVLALERLRENS

*C. fasciculata*  SFQATRTQQRVTFATLDNTVAFQDAATGEVISSTYRASDVDRVVPEMLGVSAAVLAHVIFCHQEDCNWPLGPPKDVKRIFDDIFAATRYVLALDRLRDNS

*L. pyrrhocoris*  SFQATRSSNRVTFTTLDNTVAFQDAATGEVISSTYRASDVDRVVPEMLGVSSAVLAHVIFCHQEDCNWPLGPPKDVKRIFDDIFAATRYVLALDRLRDNS

*L. seymouri*  SFQATRTSQRVTFATLDNTVAFQDSATGEVVSSTYRASDVDRVVPEMLGVSAAVLAHVIFCHQEDCNWPLGPPKDVKRIFDDIFAATRYVLALDRLRDNS

*L. arabica*  SFQATRSAHRVTFTTLDNTVAFQDLSTGEVISSTYRSSDVDRVVPEMLGVSPAVLEHVIFCHQEECNWPLGPPKDVKRIFDDIFAATRYVLALDRLRDNS

*L. aethiopica*  SFQATRSAHRVTFTTLDNTVAFQDLATGEVISSTYRSSDVDRVVPEMLGVSPAVLEHVIFCHQEECNWPLGPPKDVKRIFDDIFAATRYVLALDRLRDNS

*L. donovani*  SFQATRSAHRVTFTTLDNTVAFQDLATGEVISSTYRSSDVDRVVPEMLGVSPAVLEHVIFCHQEECNWPLGPPKDVKRIFDDIFAATRYVLALDRLRDNS

*L. gerbilli*  SFQATRSAHRVTFTTLDNTVAFQDLSTGEVISSTYRSSDVDRVVPEMLGVSPAVLEHVIFCHQEECNWPLGPPKDVKRIFDDIFAATRYVLALDRLRDNS

*L. enriettii*  SFQATRSAHRVTFTTLDNTIAFQDSATGEVISSTYRASDVDRVVPEMLGVSPAVLEHVIFCHQEECNWPLGPPKDVKRIFDDIFAATRYVLALDRLRENS

*L. infantum*  SFQATRSAHRVTFTTLDNTVAFQDLATGEVISSTYRSSDVDRVVPEMLGVSPAVLEHVIFCHQEESNWPLGPPKDVKRIFDDIFAATRYVLALDRLRDNS

*L. panamensis*  SFQATRSAHRVTFTTLDNTVAFQDSATGEVISSTYRSSDVDRVVPEMLGVSPAVLTHVIFCHQEECNWPLGPPKDVKRIFDDIFAATRYVLALDRLRDNS

*L. turanica*  SFQATRSARRVTFTTLDNTVAFQDLSTGEVISSTYRSSDVDRVVPEMLGVSPAVLEHVIFCHQEECNWPLGPPKDVKRIFDDIFAATRYVLALDRLRDNS

*L. tropica*  SFQATRSAHRVTFTTLDNTVAFQDVATGEVISSTYRSSDVDRVVPEMLGVSPAVLEHVIFCHQEECNWPLGPPKDVKRIFDDIFAATRYVLALDRLRDNS

*L. major*  SFQATRSAHRVTFTTLDNTIAFQDLSTGEVISSTYRSSDVDRVVPEMLGVSPAVLEHVIFCHQEECNWPLGPPKDVKRIFDDIFAATRYVLALDRLRDNS

*L. mexicana*  SFQATRSAHRVTFTTLDNTVAFQDLATGEVVSSTYRASDVDRVVPEMLGVSPAVLEHVIFCHQEECNWPLGPPKDVKRIFDDIFAATRYVLALDRLRDNS

*T. cruzi*  SFQALRTRTKTTFTTLDSTVAFQDTATGKVLSSTYRANDVDRAVPEMLGVSPAVLEHVIFCHQEDANWPLLPPKEVKKIFDEIFAATRYVLALDRLRENS

*T. brucei brucei*  SFQATRTRNKTTFATLDNIVAFQDSATGKIISSTYRANDVDRAIPDMLGVSPAVLEHVIFCHQEDGNWPLSPPKEVKKIFDDIFAATRYVLALDRLRENN

*T. brucei gambiense* SFQATRTRNKTTFATLDNIVAFQDSATGKIISSTYRANDVDRAIPDMLGVSPAVLEHVIFCHQEDGNWPLSPPKEVKKIFDDIFAATRYVLALDRLRENN

*T. evansi*  SFQATRTRNKTTFATLDNIVAFQDSATGKIISSTYRANDVDRAIPDMLGVSPAVLEHVIFCHQEDGNWPLSPPKEVKKIFDDIFAATRYVLALDRLRENN

*T. congolense*  SFQAVRARNKTSFTTLDNIVAFQDAATGKVVSSTYRSNDVDRAIPDMLGVTPAVLEHVIFCHQEEGNWPLSTPKEVKKIFDDIFAATRYVLALDRLRENN

210 220 230 240 250 260 270 280 290 300

....|....|....|....|....|....|....|....|....|....|....|....|....|....|....|....|....|....|....|....|

jaculum1 KELRRQMRDHETTLMALREHRDQARQLQEELARKEAAVREMAERNAHDEPELTQLRATAAALDALERDAEALATEATLLHGRMAEKRDAHARALVAARTQ

jaculum2 KELKRQQKEHETTLMALREHREQAAQLEDEIARKQAAIEEVRKQNKSVEPELRELYSSSAQIDSVEREMEGLMKEAMLLEGRIEEKRQAITRTRHARSS-

*C. fasciculata*  KEFRRQLKEHEASLMALREHREQAKQLSQQIEQKESTIKALQGRSTGIEPELQELRTALEQLRAVEDQIESLQRAVAVTKARLEERREAVRRADLPVST-

*L. pyrrhocoris*  KEFRRQLKEHEASLMALREHREQAKQLQQQIDQKDSVIRAIQGRSTGIEPELQELRQALTQLRSVEDQIESMQREVAVTQARLEERREAVRRADLPLST-

*L. seymouri*  KEFRRQLKEHETSLMALREHREQAKLLQQQVDQKENVIRTIQGRSTGIEPELQELRQALEQLRGVEDQIESMQREVAVTKARLEERREAVRRADLPSTT-

*L. arabica*  KEFRRQLKEHEASLMALREHREQAKQLEQQIAQKESVVKAIQGRSVGIEPELRGLRQAREALRTVEEQIETLQREVAVTNGRLEERREAVRRMGVPATS-

*L. aethiopica*  KEFRRQLKEHEASLMALREHREQAKQLEQQIAQKESVVKAIQGRSIGIEPELRGLRQAREALRTVEEQIEALQREVAVTNGRLEERREAVRRMGGPATS-

*L. donovani*  KEFRRQLKEHEASLMALREHREQAKQLEQQIAQKESVVKAIQGRSIGIEPELRGLRQAREELRTVEEQIETLQREVAVTNGRLEERREAVRRMGVPATS-

*L. gerbilli*  KEFRRQLKEHEASLMALREHREQAKQLEQQIAQKESVVKAIQGRSIGIEPELRGLRQAREALRTVEEQIETLQREVAVTNGRLEERREAVRRMGVPATS-

*L. enriettii*  KEFRRQLKEHEASLMALREHREQAKQLEQQIAQKESVIKAIQSRSTGIEPELRGLRQAREELRTVEEQIETMQREVAVTNGRLEERREAVRRMGAPVAS-

*L. infantum*  KEFRRQLKEHEASLMALREHREQAKQLEQQIAQKESVVKAIQGRSIGIEPELRGLRQAREELRTVEEQIETLQREVAVTNGRLEERREAVRRMGVPATS-

*L. panamensis*  KEFRRQLKEHEASLMALREHREQAKQLEQQIAQKESLVKAIQSRSIGIEPELRGLCQAREELRAVEEQIETLQREVAVTSGRLEERREAIRRMGAPTTS-

*L. turanica*  KEFRRQLKEHEASLMALREHREQAKQLEQQIAQKESVVKAIQGRSIGIEPELRGLRQAREALRTVEEQIETLQREVAVTNGRLEERREAVRRMGVPATS-

*L. tropica*  KEFRRQLKEHEASLMALREHREQAKQLEQQIAQKESVVKAIQGRSIGIEPELRGLRQAREALRTVEEQIEALQREVAVTSGRLEERREAVRRMGGPATS-

*L. major*  KEFRRQLKEHEASLMALREHREQAKQLEQQIAEKESVVKAIQGRSIGIEPELRGLRQAREALRTVEEQIEALQREVAVTNGRLEERREAVRRMGVPATS-

*L. mexicana*  KEFRRQLKEHEASLMALREHREQAKQLEQQIAQKESVVKAIQDRSIGIEPELRGLRQAREALRTVEEQIETLQREVAVTNGRLEERREAVRRMGVPATS-

*T. cruzi*  KEFRRQQKEHEANLMALREHREQAQQLTGDIAAKEELVRTIQQRAKSLEPQLKELHAVTAALSAVEQGAEGLAREAAMIQGRIDEKQESLSRMTLPPTT-

*T. brucei brucei*  KELRRQQKEHEASLMSLSEHREQARQISADITVKEETVAGIKAKTDALAPQLQELQAIATALDNVEHRAEGLSREAAVIEGRIGEKRESLSRLNVSPPG-

*T. brucei gambiense* KELRRQQKEHEASLMSLSEHREQARQISADITVKEETVAGIKAKTDALAPQLQELQAIATALDNVEHRAEGLSREAAVIEGRIGEKRESLSRLNVSPPG-

*T. evansi*  KELRRQQKEHEASLMSLSEHREQARQISADITVKEETVAGIKAKTDALAPQLQELQAIATALDNVEHRAEGLSREAAVIEGRIGEKRESLSRLNVSPPG-

*T. congolense*  KELRRQQKEHEATLAALSEHREQARQLLADIAAKESTLEANVERRNELVPKLEEIQAVIRDLNEVEQKAESLSREAAIIEGRIGEKKESLVRLDVGVIT-

310 320 330 340 350 360 370 380 390 400

....|....|....|....|....|....|....|....|....|....|....|....|....|....|....|....|....|....|....|....|

jaculum1 QQQQQQRPVGMHRDKVDVDVDDDNNDVDDVIVDDDDDVGENGVRDG--GDATPLAELQAAHEAAAAQRRDGDAAVAAAGQRCEVAQAAARGCETRLYELR

jaculum2 -----SLP-NLTSENIDSSDNYNNNRNNNNNEGNRSDADSNKYNSGTVFEHMPLADLINKEKQIKVEIAETEVELSRASQELNASEEGTRFCEGDVHSLN

*C. fasciculata*  ---------------------------------------------------QTLEAMREMRANFGAQMARVEEELATNAKRHDTAAQARRTQEEEVFRAR

*L. pyrrhocoris*  ---------------------------------------------------QTLETMQEMRSNFGAQIQHVEEELAQNTKRHDAVAQTRRTQEEELYRTR

*L. seymouri*  ---------------------------------------------------HSLETMQEMRSNFGAQMKQVEAELAQNTKRHDAVAQTRRTQEEELYRTR

*L. arabica*  ---------------------------------------------------QTLEDLLEMRSSFTAQMQQLEEGLVQDTKRYDAAAARRRAQEEDMYKCR

*L. aethiopica*  ---------------------------------------------------QTLEDLLEMRGSFTAQMQQLEEGLVQDTKRYDAAAARRRAQEEAMYKCR

*L. donovani*  ---------------------------------------------------QTLDDLLEMRGSFTAQMQQLEEGLVHDTKRYDAAAARRRAQEEEMYKCR

*L. gerbilli*  ---------------------------------------------------QTLEDLLEMRGSFTAQMQQLEEGLVQDTKRYDAAAARRRAQEEDMYKCR

*L. enriettii*  ---------------------------------------------------QTLEELLELRSSFATQMQQLEEGLVHDTKRHEAAAARRRAHEEDMYKCR

*L. infantum*  ---------------------------------------------------QTLDDLLEMRGSFTAQMQQLEEGLVHDTKRYDAATARRRAQEEEMYKCR

*L. panamensis*  ---------------------------------------------------QTLEDLLEIRRCFGAQMQQLEESLVRDTKRHDAAAARRRIQEEEMYKCR

*L. turanica*  ---------------------------------------------------QTLEDLLEMRGSFTAQMQQLEEGLVQDTKRYDAAAARRRAQEEDMYKCR

*L. tropica*  ---------------------------------------------------QTLEDLLEMRGSFTAQMQQLEEGLVQDTKRYDAAAARRRAQEEEMYKCR

*L. major*  ---------------------------------------------------QTLEDLLEMRGSFTAQMQQLEEGLVQDTKRYDAAAARRRAQEEDMYKCR

*L. mexicana*  ---------------------------------------------------QTLEDLLEMRGSFTAQMQQLEEGLVHDTKRHDTAAARRRAQEEEMYKCR

*T. cruzi*  ---------------------------------------------------LTIEEMLEFKQGFVERIKGVEADASDKVNLLEKAEAKKQQCEETALRLR

*T. brucei brucei*  ---------------------------------------------------YSLGELLQRRQNFGEKLRELESTVSTKKELMERAVAELRRCEESVVSLR

*T. brucei gambiense* ---------------------------------------------------YSLGELLQRRQNFGEKLRELESTVSTKKELMERAVAELRRCEESVVSLR

*T. evansi*  ---------------------------------------------------YSLGELLQRRQNFGEKLRELESTVSTKKELMERAVAELRRCEESVVSLR

*T. congolense*  ---------------------------------------------------HTKEELLQRQENFDNKLKKLEEELVSKKDLLSQAEASVRKHEENVVSLR

410 420 430 440 450 460 470 480 490 500

....|....|....|....|....|....|....|....|....|....|....|....|....|....|....|....|....|....|....|....|

jaculum1 TRLQLCEHAAHEQQRHRDALQQALASLYAKYGG-DDDDDGTQNSVNVVVDDDVGTDKDAARVHAVVARARACVQRRRDAAHATVAAHAAHERDAAAAVQA

jaculum2 VTVRLLQQEMEQHVRNQDELRHLLQGVQPYLTL-PNSYLDSHETLNDYIDHIV---HDEVELTNTLAKLQETVSERQSEICKVEQHNASTIHDAEERRQS

*C. fasciculata*  SNLQLLEREDQQHRQSVARLRDLLHGMSEEVAIGEADV-------------------DERSVQRVLRKAEADVDTARRQLSSDTVELRQRLEAEEEELQR

*L. pyrrhocoris*  SNIQLLERDDQQHKLSVARLRELLSELSSEYVLGENDV-------------------DERSLQRLLRKAESDAAAARQQLRSATAGLQQSIRAEESEVQS

*L. seymouri*  SNIQLLERENQQHKHNVVRLRELLHELSSEFVLSENNV-------------------DERGLQRLLKKAESDVTTARQQLQSAAADLQHSIREMENEVQG

*L. arabica*  SNVQLLEREAQLHKQNVAQLRERIQHLSHEYVMSEGTI-------------------DERSLQRLLQKAEDAVAAERQRARAASESVQQRIRDAEDGVQC

*L. aethiopica*  SNVQLLEREAQLHKQNVAQLRELIQHLSHEYVLSEGNI-------------------DERSLQRLLQKAEDAVAAERQRARAASEGVQQRIRDAEGGVQG

*L. donovani*  SNVQLLEREAQLHKQNVAQLRELIQHLSNEYVLSEGNI-------------------DERSLHRLLQKAEDAVAAERQRARAASESVQQHIRDAEDGVQG

*L. gerbilli*  SNVQLLEREAQLHKQNVAQLRELIQHLSHEYVLSEGNI-------------------DERSLQRLLQKAEDAVAAERQRARAASESVQQRIRDAEDGVQC

*L. enriettii*  SNVQLLEREAQVHKQNVAQLRELIQHLSSECVLSEDNI-------------------DESSLRRVLQRAEEAVAAARHRVHAVAESFQERIQDAEGGAQG

*L. infantum*  SNVQLLEREAQLHKQNVAQLRELIQHLSNEYVLSEGNI-------------------DERSLHRLLQKAEDAVAAERQRARAASESVQQHIRDAEDGVQG

*L. panamensis*  SNVQLLEREALVHKGNVVQLRELIQHLSSEYVLSEDSI-------------------DELSLQRVLQKAEVAVSAARQRICAATECVQQRIQDAEDGVQG

*L. turanica*  SNVQLLEREAQLHKQNVAQLRELIQHLSHEYVLSEGNI-------------------DERSLQRLLQKAEDAVAAERQRARAASESVQQRIRDAEDGVQC

*L. tropica*  SNVQLLEREAQLHKQNVAQLRELIQHLSHEYVLSEGNI-------------------DERSLQRLLQKAEDAVAAERQRARAASEGVQQRIRDAEGGVQD

*L. major*  SNVQLLEREAQLHKQNVAQLRELIQHLSHDHILSESNI-------------------DERSLQRLLQKAEDAVAAERQRARTASESVQQRIRDAEDGAQC

*L. mexicana*  SNVQLLEREAQLHKQNVARLRELIQHLSNEYVLSEGNI-------------------DERSLQRLLQKAEEAVAVERRRARTTSEGIQQRIRDAEDGVQG

*T. cruzi*  STTEFLEQQERQYKENCMELQGIVKNLSTGLVLGDDDM-------------------CEEGLQRVSDHLNAELQMLKAERDKTLKEFDDEKKLLEDQRNM

*T. brucei brucei*  SGIEFREKQEQQHKRECEELKVIMSDLSMKFVI-DGEI-------------------NEHCLTKITEYVNEELHNEEQKRSGELNSIDAGIRAVEDRRST

*T. brucei gambiense* SGIEFREKQEQQHKRECEELKVIMSDLSMKFVI-DGEI-------------------NEHCLTKITEYVNEELHNEEQKRSGELNSIDAGIRAVEDRRST

*T. evansi*  SGIEFREKQEQQHKRECEELKVIMSDLSMKFVI-DGEI-------------------NEHCLTKITEYVNEELHNEEQKRSGELNSIDAGIRAVEDRRST

*T. congolense*  SVIAFREQQEQQHMKDCEQLAQVLKGVTSKFTV-DENM-------------------NEESLSRIMDYVSDELREAEKEYNDLVHSIEGKVSAIEKEQSA

510 520 530 540 550 560 570 580 590 600

....|....|....|....|....|....|....|....|....|....|....|....|....|....|....|....|....|....|....|....|

jaculum1 QQRATDVTSAERALLTQRCSELEARVQRTAAEAAALDS-GGGGGDGCAVRLARARDAAAALTQRVQAADAVR-RGGARAQQRQTLLRDADALSVRVAQLT

jaculum2 LLRSIEGKRQEYTMLQSLIGDAKEKLRHSRAEHAQLGS-RES----LEKEIANANEQLSTLTARVHVAESVRLQDNDEHSQRVKLLHELDEQNRRVAELR

*C. fasciculata*  AVRELDANTNEARMRQQSLDAVRRRIADNAAALAALGGADAH-----RTQLRHLQAEVEELRQRVAAAEELR-GKGEAAQRSGAILRELEEHNVTVAALR

*L. pyrrhocoris*  GLRLLDGNLTETQMRQQSLDAVRQRVKDNARAIAGLGG-DSR-----RVQLRQLQAEIEELQQRVAAAEELR-RKDTTEQRSQAILKELEEQNIAVATLR

*L. seymouri*  CLRLLDVNTNETQMRQHSLDTARRRMSDNARAIADLGG-ASR-----RVQLQQLQDEVEELQQRVAAAEKLR-EKGSGEQRSQSILKELEEQNITVAQLR

*L. arabica*  GSRAVDGCNKEIEVREHAVENVRRRIKEHAAAIAALGG-EGC-----RTQLRQVQVDVEELQQRVAAAEELR-KKGGGRHQSQRILMELEEQNSTVAQLR

*L. aethiopica*  GSRAVDGCNKEIEVREHAVENVRRRIRENAAAIAALGG-DGC-----RTQLRQVQVEVEELQQRVAAAEELR-KKGGGRHQSQRILMELEEQNSTVAQLR

*L. donovani*  GSRAVDGCKKEIEVREHAVENVRRRIKENAAAIAALGG-DGC-----RTKLRQVQVEVEELQQRVAAAEELR-KKGDGEHQSQRILMELEEQNSTVAQLR

*L. gerbilli*  GSRAVDGCQKEIEVREHAVENVRRRIKEHAAAIAALGG-EGC-----RTQLRQVQVEVEELQQRVAAAEELR-KKGGGRHQSQCILMELEEQNSTVAQLR

*L. enriettii*  GSRALDGCNKEMEMREQALESLRRRIKENAAAVSALGG-DGC-----RLQLRQIQAEVEELQQRVAAAEELR-KKGSKGQCSQRILTELEEQNRIVAQLR

*L. infantum*  GSRVVDGCKKEIEVREHAVENVRRRIKENAAAIAALGG-DGC-----RTKLRQVQVEVEELQQRVAAAEELR-KKGDGEHQSQRILMELEEQNSTVAQLR

*L. panamensis*  NSRAVDGCNREIEMREHALEGVCRRIKENAAAISALGG-DGC-----RTQLLQVQTEVEELQQRVAAAEVLR-KKGGEAKRSQHILMELEEQNSAVAQLR

*L. turanica*  GSRAVDGCKKEIEVREHAVENVRRRIKEHAAAIAALGG-EGC-----RTQLRQVQVEVEELQQRVAAAEELR-KKGGGRHQSQRILMELEEQNSTVAQLR

*L. tropica*  GSRAVDGCNKEIEVREHAVENVRRRIRENAAAIAALGG-DGC-----RAQLRQVQAEVEELQQRVAAAEELR-KKGGGRHQSQRILMELEEQNSTVAQLR

*L. major*  ASRAVDGCKKEIEVREHAVENVRRRIKEHAAAIAALGG-EGC-----RTQLRQAQVEVEELQQRVAAAEELR-KKGGGLHQSQRILMELEEQNSTVAQLR

*L. mexicana*  SSRAVDGCNKEIEMREHAVENVRRRMKENAAAIAALGG-DGC-----RGQLRQVQVEVEELQQRVAATEELR-KKGSGGHQSQRIIMELEEQNSTVAELR

*T. cruzi*  LLRSMDADNKEKDMKEDQLKHLHQRVVSTEEALGKLKP-YVG-----ATHIESLKKTISDLEQRLEVMEELK-KKGESYKQRQDILQRIDAQNRIVAGLR

*T. brucei brucei*  TLRAMDTEGKEKEMKEEQLRHLMKRCGDAKEALGKLAP-YVT-----PTRLKNVQDTITELEKRVEAMELLQ-KGDARYRQRQDILQSVEAQNKVVAQLR

*T. brucei gambiense* TLRAMDTEGKEKEMKEEQLRHLMKRCGDAKEALGKLAP-YVT-----PTRVKNVQDTITELEKRVEAMELLQ-KGDARYRQRQDILQSVEAQNKVVAQLR

*T. evansi*  TLRAMDTEGKEKEMKEEQLRHLMKRCGDAKEALGKLAP-YVT-----PTRVKNVQDTITELEKRVEAMELLQ-KGDARYRQRQDILQSVEAQNKVVAQLR

*T. congolense*  AFRSIDADNKEKEMKEEQLRLVLKRRTEAEGALKQLEP-HVT-----QAQLAGLQEKITELEQRAEAMEQLR-KSNSDYEERRNILQKFEAQNKIVAELR

610 620 630 640 650 660 670 680 690 700

....|....|....|....|....|....|....|....|....|....|....|....|....|....|....|....|....|....|....|....|

jaculum1 RHMVALQQRESARAETTVLREQLRSREAHVRATLTQQLLPQLAALGVHVQQNNSSHSNYNSDDGGATSTALLARVTVCVADARRQRDEAAARAQSELSAL

jaculum2 QRLAEQTEQQRSRTEITILQTQISKSESNLTNELVNSVWPRLVELAL-VDSTS-------------TESVNVNTANSVLTHAIMRCQRELAECQSEQSSL

*C. fasciculata*  TEMMRQKQRERVQQEAEHLRSQVAESDASVAAALQDEVLPRLREIGVQAP-VVTADA---------FTPSVLLSLRSQATQVHQAKAAELQTLQSRVFEL

*L. pyrrhocoris*  EEMMRQKQRERSEQEAEHLRRQVEESNVSVAAALQNDVLPLLRAVGVQAP-VVATDA---------FSPSVLLSLRSQATQVHQAKANELQALQSRVFEL

*L. seymouri*  EEMMRQKQQERIQQEAAQLRQQVEESNASVTAALQNEVLPLLRAVGVQAP-DVAMDT---------FSSSILLSLRSQATKLHQAKANELHSLQNRMFEL

*L. arabica*  EEMMQQKLLERQQQDLVLMRQQIDEARAAVAAALQRDVLPILHEVGVQVPQEAAADS---------FSLTTLTPLRSQAAQVRQLKAEELRALQTRVLEL

*L. aethiopica*  EEMMQQKLLERQQQDLAMMRQQIDEARAAVVAALQRDVLPILHEVGVQVPQEAAADS---------FSVTTLTPLRSQAAQVRQLKAEELRALQTRVLEL

*L. donovani*  EEMMQQKLLERQQQDLVLMRQQIDEARAAVAAALQRDVLPILHEVGVQVPQDAAADS---------FSLTTLTSLRSQAAQIRQLKAEELRALQARVLEL

*L. gerbilli*  EEMMQQKLLERQQQDLVLMRQQIDEARAAVAAALQRDVLPILHEVGVQVPQEAAADS---------FSLTTLTPLRSQAAQVRQLKAEELRALQTRVLEL

*L. enriettii*  EEMMQQKMLERQHQEIMLLRQQVDESSGAVATALQRDVLPVLLEIGVEAPREVADDC---------LSPQTLTSLRNQASQIQKLKMEELRSLQTRLLEL

*L. infantum*  EEMMQQKLLERQQQDLVLMRQQIDEARAAVAAALQRDVLPILNEVGVQVPQDAAADS---------FSLTTLTSLRSQAAQIRQLKAEELRALQARVLEL

*L. panamensis*  EEMMQQKLMERQGHDLVLLRQQVDESNAAVATALQRDVLPILQEIGMQVPQDVGADT---------FSLQTLTSLRTQTAQVQQLKAEDLRALQTRVLEL

*L. turanica*  EEMMQQKLLERQQQDLVLMRQQIDEARAAVAAALQRDVLPILHEVGVQVPQEAAADS---------FSLTTLTPLRSQAAQVRQLKAEELRALQTRVLEL

*L. tropica*  EEMMQQKLLERQQQDLVMMRQQIDEARVAVAAALQRDVLPILHEVGVQVPQEAAADS---------FSLTTLTPLRSQAAQVRQLKAEELRALQTRVLEL

*L. major*  EEMMQQKLLERQQQDLALMRQQIDEARAAVAAALQRDVLPILHEVGVQVPQEAAADS---------FSLPALTSLRSQASQVRQLKAEELRALQTRALEL

*L. mexicana*  EEMMQQKLLERQQQDLVLLRQQIDESSAAVAAALQRDVLPILHEVGVQVPQEAAADS---------LSLTTLTSLRSQAAQVRQLKAEELSALQTRALEL

*T. cruzi*  QELARHKECLGGEAEMNLLRTQIAEKEGFLEAEMKETLVPELSNFGHEM-----------------TEGRSLSQISLLIEQLREQKLGVLRVIQTEHGEL

*T. brucei brucei*  QELSRHKQRSGREAEMNLLRTQIAEKEEIINNRLQEELIAGLNDLGCNT-----------------GGSQTLTTVTMQIDKLRRKMADTLYSVEAEVNDL

*T. brucei gambiense* QELSRHKQRSGREAEMNLLRTQIAEKEEIINNRLQEELIAGLNDLGCNT-----------------GGSQTLTAVTMQIDKLRRKMADTLYSVEAEVNDL

*T. evansi*  QELSRHKQRSGREAEMNLLRTQIAEKEEIINNRLQEELIAGLNDLGCNT-----------------GGSQTLTAVTMQIDKLRRKMADTLYSVEAEVNDL

*T. congolense*  QQLARHNERSCREAEMNLLRTQISEKQERVDKGLRETLIAGLEAFGHDV-----------------VESSSLTHVTMQVEKLRDKKADALRNIEAEANEL

710 720 730 740 750 760 770 780 790 800

....|....|....|....|....|....|....|....|....|....|....|....|....|....|....|....|....|....|....|....|

jaculum1 ERHGAVTEQRRLQLLDELTHTEAQARACATRCARALAADDTAS---------------------------SSADAAAAADAVVVVDDD----------DK

jaculum2 ERNRALVEQRQASHMQEKMTKEMEMHSWSEDCRRVLMPLLSSHVYHVELGSEQTIEPETIQRNQTEVKQESVPESKYVSSAIRTNSDDYSSNTGINRGDP

*C. fasciculata*  EKARAAQEQKTAYLSTELVACQREVVREKEQCVCLI----------------------------------------------------------------

*L. pyrrhocoris*  QKARATQEQKSAYLSEELVQCQREVSDGLAQCVNSV----------------------------------------------------------------

*L. seymouri*  QKTRATQEQKTAYLSEELVQCQREVTNGITECAGLV----------------------------------------------------------------

*L. arabica*  EKACALQEQKMSYSTDELGQCRRAVDAGTRQCAGFF----------------------------------------------------------------

*L. aethiopica*  EKACALQEQKMSYSTDELGQCRRAVDAGTRQCAGLF----------------------------------------------------------------

*L. donovani*  EKVCALQEQKMSYSTDELGQCRRTVDAGTRQCAGLV----------------------------------------------------------------

*L. gerbilli*  EKACALQEQKMSYSTDELGQCRRAVDAGTRQCAGFF----------------------------------------------------------------

*L. enriettii*  EKEYAIGEQKITYCADELAQCRQAVDAGTRQCAGLV----------------------------------------------------------------

*L. infantum*  EKVCALHEQKMSYSTDELGQCRRTVDAGTRQCAGLV----------------------------------------------------------------

*L. panamensis*  ENMCAIEEQQMSYNTNELDQCRRSVDAGTRQCSGLV----------------------------------------------------------------

*L. turanica*  EKACALQEQKMSYSTDELGQCRRAVDAGTKQCAGFF----------------------------------------------------------------

*L. tropica*  EKACALQEQTMSYSTDELGRCRRAVDAGTRQCADLF----------------------------------------------------------------

*L. major*  EKACALQEQKMSYSTDELGQCRRAVDAGTRQCAGLF----------------------------------------------------------------

*L. mexicana*  EKACALQEQKMSYSADELAQCRRAVDAGTRQCAGLF----------------------------------------------------------------

*T. cruzi*  DRQIAVLQQNQSQRMEEIMRENSELQRKRTSCVKAL----------------------------------------------------------------

*T. brucei brucei*  DRQLIALKQNRSQLEGKIVSENIELQRKRVQCISKL----------------------------------------------------------------

*T. brucei gambiense* DRQLIALKQNRSQLEGKIVSENIELQRKRVQCISKL----------------------------------------------------------------

*T. evansi*  DRQLIALKQNRSQLEGKIVSENIELQRKRVQCISKL----------------------------------------------------------------

*T. congolense*  DRQLVVLQQRQSQVADELKRESIELKRKRSHCISRL----------------------------------------------------------------

810 820 830 840 850 860 870 880 890 900

....|....|....|....|....|....|....|....|....|....|....|....|....|....|....|....|....|....|....|....|

jaculum1 GDSVTTTTTAAVGA----------------AEEDVCVDDYEVVLQRARDAAAAHTRTQHAHETMTTCYADFVALARSTGACAVCERPFASDEAVAQFVAA

jaculum2 SSSSLSSSTLSQRARQSHQTFDESKEVYDLTSPRHILAAYTKILQSIRHRVAELSEKCNNAQAREMCYHEFVRAAETRNLCMVCNRPFNTEEERQRFIEI

*C. fasciculata*  ----------------------------------SDVDAYEVQLQEARDAAESSTQKWHALEALATCYHEFMNVAAADNMCAVCERPFADHAAQQRFMDI

*L. pyrrhocoris*  ----------------------------------TDLDTYEEYLRQAREAAESSTQKWHAMEALVTCYHDFMSVAMSENKCAVCERPFADHDVRQKFLEI

*L. seymouri*  ----------------------------------KDLDTYEEQVQRAKVAVESATQEWHALEALATCYHGFVKVAVSENRCAVCERPFADRDAMQSFVDA

*L. arabica*  ----------------------------------PDMDAYEEVLLQAKEAAETAARKHHALEALASCYHDFMVVAKEERTCAVCERPFDSDDSLESFLTR

*L. aethiopica*  ----------------------------------PDMDAYEEVLLQAREAAEAAAQKRHAVEALASCYHDFMVVAKEERTCAVCERPFDSDDSLESFLTR

*L. donovani*  ----------------------------------PDMDVYEEVLLQAREAAETAAQKRHALEALASCYHDFMVVAKEERTCAVCERPFDSEDALESFLTR

*L. gerbilli*  ----------------------------------PDMDAYEEVLLQAKEAAETAARKRHALEALASCYHDFMVVAKEERTCAVCERPFGSDDSLESFLTR

*L. enriettii*  ----------------------------------PNMDVYEEVLLKAREAAEAAAQRRHSMEVLATCYRDFAVMAKKEKTCAVCERPFNTHESLENFLSR

*L. infantum*  ----------------------------------PDMDVYEEVLLQAREAAETAAQKRHALEALASCYHDFMVVAKEERTCAVCERPFDSEDALESFLTR

*L. panamensis*  ----------------------------------SNMEDYEEMLLQAREAAETASQRRHALETLATCYHDFMVVAREKGMCAVCERSFESEDALERFLTR

*L. turanica*  ----------------------------------PDMDAYEEVLLQAKEEAETAARKRHALEALASCYHDFMVVAKEERMCAVCERPFDSDDSLESFLTR

*L. tropica*  ----------------------------------PDMDAYEEVLLQAREAAEAAAQKRHALEALASCYHDFMVVAKEERTCAVCERPFDSDDSLESFLTR

*L. major*  ----------------------------------PDMDAYEEVLLQAKEAAETAAQKRHALEALASCYHDFMVVAKGERMCAVCERPFDSDDSLESFLTR

*L. mexicana*  ----------------------------------PDMDAYEGVLLQAREAAETAVQKRHALEALASCYHDFMVVAKEEKTCAVCERPFDSDDSLESFLTR

*T. cruzi*  -------------------------------DGLGEIEHFEAVLEKARDLLQAARNRHHALEAMSTCYANFVQVAREEGKCPVCDRVFTDELALANFVDL

*T. brucei brucei*  -------------------------------GNDDALTNFEALLVEARDRYHKLNEKLSGSKALAACHAHFVEQAKVEDKCPLCGRAFGSENELNDFLAS

*T. brucei gambiense* -------------------------------GNDDALTNFEALLVEARDRYHKLNEKLSGSKALAACHAHFVEQAKVEDKCPLCGRAFGSENELNDFLAS

*T. evansi*  -------------------------------GNDDALTNFEALLVEARDRYHKLNEKLSGSKALAACHAHFVEQAKVEDKCPLCGRAFGSENELNDFLAS

*T. congolense*  -------------------------------GSAEALDSFEAAFIKAREAHHSMSHEQSASRAMAACYSHFVEKAKADGKCPLCDHNFTDDAALNNFLDT

910 920 930 940 950 960 970 980 990 1000

....|....|....|....|....|....|....|....|....|....|....|....|....|....|....|....|....|....|....|....|

jaculum1 TEERQR----------------RAPAAMATARAAAQTALARLRRLEAAQEDVLQLRQLRAAHAHKEQLVRQLTSELQSNAAQRAECAARSDAAARAAAAM

jaculum2 NSELTRSDSNTNNNILSESTYKCNQSVNSTLHNDLHIAQLQLASLESLESSMTRLQQHCNTLPQLNASITELASELATIVEEERTVCEKCNELQLELHTL

*C. fasciculata*  NADKQR----------------TTPEALAAVRGAADTAQDAFQQLERLQSVVASLRQHQQRVPVLEADLERLAAQSAETTSALADASAACDAAQQAVQQL

*L. pyrrhocoris*  NADKQR----------------ATPQALAAVRGAADTAQEAYQRLEKLQGVVVSVRQHRSRIPVLEEELKSLVVQMEQNTAAQGEARAARDAAQTAVQQL

*L. seymouri*  NADKQR----------------TTPEALAAARGAADAAQETCQRLEKLQGVVLSVRRHQCRVPLLEDDLKSLAEQMELNTAAQKEAQAARDAAQEAVLRL

*L. arabica*  KVDRQR----------------TSPEALAAVQAAVNTAQEAYQSLEKLQSVVVTVRQNRHRIPVLEAELDALEEKMKQNRAEHREVCAARDAAQHVVHQL

*L. aethiopica*  KADRQR----------------TSPEALAAVQAAVNTAQEAFQSLEKLQSVVVTVRQNRHRIPVLEAELDALEDKMRQNRAEQREVCAARDAAQHVVHQL

*L. donovani*  KADKQR----------------ASPEALAAVQAAVNTAQEAYQSLEKLQSVVVTVRQNRHRIPVLEAELDALGDKMKQNRAEQRGVCAARDAAQHVVHQL

*L. gerbilli*  KVDRQR----------------TSPEALAAVQAAVNTAQEAYQSLEKLQSVVVTVRQNRHRIPVLEAELDALEDKMKQNRAEHREVCAARDAAQHVVHQL

*L. enriettii*  NADKQR----------------IRPETLAAAQEAVTAAQDAYQSLEKLQSVVTTVRQHRHRIPILEADLGALEDKMKENLAEQRKACGARDAAQQVVHQL

*L. infantum*  KADKQR----------------ASPEALAAVQAAVNTAQEAYQSLEKLQSVVVTVRQNRHRIPVLEAELDALGDKMKQNRAEQRGVCAARDAAQHVVHQL

*L. panamensis*  NAAKQR----------------TSPDQLAAVEAAVTKAQATYEGFEKLQNVVATVRRNLPRIPDLEAELEALEERAKKNRAEQRKVSAARDASQHVVHQL

*L. turanica*  KVDRQR----------------TSPEALAAVQAAVNTAQEAYQSLEKLQSVVVTVRQNRHRIPVLEAELDALEDKMKQNRAEHREVCAARDAAQHVVHQL

*L. tropica*  KADRQR----------------TSPEALAAVQAAVNTAQEAYQSLEKLQSVVVTVRQNRHRIPVLEAELDALEDKMKQNRAEQREVCAARDAAQHVVHQL

*L. major*  KADRQR----------------TSPETLAAVQAAVNTTREAYQSLEKLQSVVVTVRQNRHRIPVLEAELDALEDKMKQNRAEQREVCAARDAAQHEVHQL

*L. mexicana*  KADRQR----------------TSPEALAAVQATVTAAQEAYQSLEKLQSVVVTVRQNRHRIPVLEVELNALEDKMKQSCAEQREVCATRDAAQHVVHQL

*T. cruzi*  NERHHG----------------ASPEMIEKAHLEVTEAEERVRILESLEADVHDVRRLASSVPHLELLVTSINEELANKSVLLEDAERKREDVEHQLKRV

*T. brucei brucei*  FKVGQQT---------------SGKDSIKEG--DVEKALQRVRGLEQLESDVMDVRRLADNAPQLEESLKSTIKQIRDKEILLEDVHNKRDKVKDEMQRA

*T. brucei gambiense* FKVGQQT---------------SGKDSIKEG--DVEKALQRVRGLEQLESDVMDVRRLADNAPQLEESLKSTIKQIRDKEILLEDVHNKRDKVKDEMQRA

*T. evansi*  FKVGQQT---------------SGKDSIKEG--DVEKALQRVRGLEQLESDVMDVRRLADNAPQLEESLKSTIKQIRDKEILLEDVHNKRDKVKDEMQRA

*T. congolense*  IKVKQQ----------------TQTNTTEK---DVEDAQGRVRMFEELEADVVSIRRLTARIPQIEESLSTVTEEINIKRALLEDVNGRRSGVEDQLKRV

1010 1020 1030 1040 1050 1060 1070 1080 1090 1100

....|....|....|....|....|....|....|....|....|....|....|....|....|....|....|....|....|....|....|....|

jaculum1 ADVAGQLQALHTHANEAAALHTQLQ-------------------------------RYEQQQQ-----------------------QQLQ------HEED

jaculum2 QSISQKVCTLTGMAQEIANMRNQLSACVRRVHTSHEVNNIINNDERSNNNLNSVDMRNEHQHKRIRDETNYTLSENNNRNEQQLEEQEQQ------FSEE

*C. fasciculata*  EGVCRHLAGLCMVEEKAAVMRVTLA-------------------------------QREREAE-----------------------VTLQEAV------E

*L. pyrrhocoris*  EGVCHHLAGLCMMEEKATALKATLT-------------------------------RREKEVE-----------------------AASPQEE---VIGA

*L. seymouri*  EGVCRHLAGLCMMDEKAASLKATLT-------------------------------RRESDAA-----------------------AARQQQR---MIET

*L. arabica*  DTMYQHLCMVCTMGERTVALRDTLA-------------------------------RKEKDFE-----------------------AAQAEQQPH-ETQR

*L. aethiopica*  DTMYQHLCMVCTMGEKTVSLRDTLA-------------------------------RKEKDFE-----------------------AAQAEQQPH-ETQR

*L. donovani*  DTMYQHLCMACTMGEKTVALRDTLA-------------------------------RKEKDFE-----------------------AAQAEQQPH-ETQR

*L. gerbilli*  DTMYQHLCMVCTMGERTVALRDTLA-------------------------------RKEKDFE-----------------------AAQAEQQPH-ETQR

*L. enriettii*  DTVYEQLCIVCAMGEKTAALRDTLA-------------------------------RREKDAE-----------------------AAQAEQRAH-ETGC

*L. infantum*  DTMYQHLCMACTMGEKTVALRDTLA-------------------------------RKEKDFE-----------------------AAQAEQQPH-ETQR

*L. panamensis*  DIVFQHLCRVCTMGEKTVALRDTLR-------------------------------RKEKDAE-----------------------AAQAEQQRQPQIRR

*L. turanica*  DTMYQHLCMVCTMGERTVALRDTLA-------------------------------RKEKDFE-----------------------AAQAEQQPH-ETQR

*L. tropica*  DTMYQHLCMVCTMGEKTVALRDTLA-------------------------------RKEKDFE-----------------------AAQAEQQPH-ETQR

*L. major*  DTMYQHLCMVCTMGERTVALRDTLA-------------------------------RKEKDFE-----------------------AAQAEQQPH-ETQR

*L. mexicana*  DTMYQHLCMVCTMGEKTVALRDTLA-------------------------------RKEKDFE-----------------------AAQAEQQQQ-ETQR

*T. cruzi*  QDLMRTAIDLNAVACDIRALRQQLS-------------------------------RREAAIK-----------------------EIQA------EVVT

*T. brucei brucei*  QKLVQAAMEVNAMTSEKQALLQQLN-------------------------------RREAACA-----------------------EAST------SSAN

*T. brucei gambiense* QKLVQAAMEVNAMTSEKQALLQQLN-------------------------------RREAACA-----------------------EAST------PSAN

*T. evansi*  QKLVQAAMEVNAMTSEKQALLQQLN-------------------------------RREAACA-----------------------EAST------PSAN

*T. congolense*  QNLMQAATDINGLASEKHFLKQQLQ-------------------------------RREAANV-----------------------EMDA------ASAK

1110 1120 1130 1140 1150 1160 1170 1180 1190 1200

....|....|....|....|....|....|....|....|....|....|....|....|....|....|....|....|....|....|....|....|

jaculum1 -----D-AMGYKDEESGSFEAVSRAYEEATAQLQRMNAQLRELERGDAASSSSGNGGEDDVDAQLRRAQ--AAVHALELTASKLADAQAALQRERLELRA

jaculum2 HTNNND-ENTKEDGEEETYDELNAKYNEATTHLHLLNVQLSELERLHAGSAEDI------TVAQREMHRMESSVQELEFKLHKVNDCIAREEREAENAQT

*C. fasciculata*  AGQAAD-TAANSAAPLRSFDELSEAYAAATDRLHQLNKLFTEAQRVERGQDEHA------AARQLQERQ--AAMYHVEVAVAKVDGLEKTARELQNEAGQ

*L. pyrrhocoris*  VGEDGD----DQVSSRRTFDELSEAYAAATDRLHQLNKLFTEAQRMERGQDEHA------AQRQLQERK--AAMYQAEVSVAKLEDLEKTSAELQQEAAQ

*L. seymouri*  VRDAKE-GQVEAVASLRTFDELSEAYAAATDRLHQLNKLFTEAQRIERGQDEHA------AERQLSERK--AAMYQAEVSVAKLEDLENASAELQQEAAQ

*L. arabica*  KWGSGDGATARTEPAPRSYEELCEAYAAATERLHKLNRQFTEAQRMERGQAENA------AERELHERK--EAMLQAEVTVAKLEDLESAARELQEEAAQ

*L. aethiopica*  KWGSGDGATARAEPAPRSYEELCEAYAAATERLHKLNRQFTEAQRMERGQAENA------AERELQERK--AAMLQAEVAVAKLEDLEAAARELQEEAAQ

*L. donovani*  KWGSGDGATARAEPPQRSYEELCEAYAAATERLHKLNRQFTEAQRMERGQAENA------AERELQDRK--TAMFQAEVAVSKLEDLEAAARELQEEAAQ

*L. gerbilli*  KWGSGDGATARAEPAPRSYEELCEAYAAATERLHKLNRQFTEAQRMERGQAENA------AERELQERK--AAMLQAEVAVAKLEDLESAARELQEEAAQ

*L. enriettii*  QRASGDGAAARIEPQQRSFDDLSEAYTAATERLHHLNRQFSEAQRMERGQDENA------AERQLQERK--AAMFQAQVAVSKLEDLALAAREQQEEALR

*L. infantum*  KWGSGDGATARAEPPQRSYEELCEAYAAATERLHKLNRQFTEAQRMERGQAENA------AERELQDRK--TAMFQAEVAVSKLEDLEAAARELQEEAAQ

*L. panamensis*  MQGSGGGTAERVQLPQRSFEELSEAYAASTERLHQLNRQFAEAQRMECGQDENA------VHRQLQERK--AALFQAEVAVAKLEDLETVARELQEEAVH

*L. turanica*  KWSSGDGATARAEPEPRSYEELCEAYAAATERLHKLNRQFTEAQRMERGQAENA------AERELQERK--AAMLQAEVAVAKLEDLESSARELQEEAAQ

*L. tropica*  KWGSGDGATARAEPAPRSYEELCEAYAAATERLHKLNRQFTEAQRMERGQAENA------AERELQERK--AAMLQAEVAVAKLEDLEAAARELQEEAAQ

*L. major*  KWGSGDGATGRAEPAPCSYEELCEAYAAATERLHKLNRQFTEAQRMERGQAENA------AERELQERK--AAMLQAEVAVAKLDDLESAARELQEEAAQ

*L. mexicana*  KGGSGDGATARAEPPQRSYEELCEAYAAATERLHKLNRQFTEAQRMERGQDENA------AERELQERK--AAMLQAEVAVAKLEDLEAAVRELQEEAAQ

*T. cruzi*  AAG--G-GGGAGGGA-RTYEEVSTEYESANTELHRLNVMLNEAQRREDGESDQV------AASELNARR--AEYYKLEMKWMRQGELEEVLARYKGEEKG

*T. brucei brucei*  IGGAGE-PCCAGELASLSYEELSDKYESANAELHRLNVLLSEMQRGEEGGSAQA------LVKELTMKR--SELCELKMKLTRQAEVEATIAQYAEQEEG

*T. brucei gambiense* IGGAGE-PCCAGELASLSYEELSDKYESANAELHRLNVLLSEMQRGEEGGSAQA------LVKELTMKR--SELCELKMKLTRQAEVEATISQYAEQEEG

*T. evansi*  IGGAGE-PCCAGELASLSYEELSDKYESANAELHRLNVLLSEMQRGEEGGSAQA------LVKELTMKR--SELCELKMKLTRQAEVEATIAQYAEQEEG

*T. congolense*  HAEMGE-NGGSGVCA-PTYEQLSAQCEEANAELHRLNVLLNELHRRQNGESDQV------LIGELTKRR--SELCELKMKFTRQSELEWAIAQCIEEENT

1210 1220 1230 1240 1250 1260 1270 1280 1290 1300

....|....|....|....|....|....|....|....|....|....|....|....|....|....|....|....|....|....|....|....|

jaculum1 LHAHRDELTTRLTQQQHALDAHATALAAATAQRMQCEAAA-QSSCGALDAALQRVEACAARVLESVRDDNNNNNNSSSTTNDDDGSGGSAVARLQCARAA

jaculum2 LERRLESVQMEIEKIETEELSHASSVLIKLREEAQLQEETMRNALRALESALHQIESATPRVLSFLNSNR--------------------TAELHSAESE

*C. fasciculata*  HRQRMQELSTLADELRPTADAHRARVQELRSQCQAAEASA-RQALEEAEARLRRLESAVSPVMAYVQGNG--------------------AQRLDTLRVH

*L. pyrrhocoris*  HRGRLEELTAQVATLRTSVDARQAKVQELRAECQRAEEAE-RHAVEQAEARLRQLQLAVPPVLSYVQSGG--------------------AQRLEELRVH

*L. seymouri*  HRDRIDELAAQAAMLRTSVDAHQARLQELRAQCHKAEDAE-RRTVEQAEARLRQLQLDVPPVLGYVQSGG--------------------TMRLEEVRVH

*L. arabica*  HRTRVEELKRQAAEAQHSVEAHQQRVLQLRAELQKTEAAE-RGALDIAEKQLRELQLSLPPVLSYLQNGC--------------------ARQLEELRMH

*L. aethiopica*  HRTRVEELKRQAAEAQHSVEAHQQRVLQLRAELQKTDAAE-RGALDTAEKQLRELQLSLPPVLTYVQNGC--------------------ARQLEELRVH

*L. donovani*  HRTRVEELKRQAAEAQHSVEAHQQRVLQLRAELQKTDAAE-RGVLDTAEKQLRELQLSLPPVLSYVQNGC--------------------ARQLEELRVH

*L. gerbilli*  HRTRVEELKRQAAEAQHSVEAHQQRVLQLRAELQKTEAAE-RGALVTAEKQLRELQLSLPPVLSYLQHGC--------------------ARQLEELRMH

*L. enriettii*  HRTRVEELKRQAAEAQRTAEAHQQRLLQLRTELQKAEASE-RGALETVEEQLRQLQQSLPPVLTYMQNGC--------------------IQRLEEVRGH

*L. infantum*  HRTRVEELKRQAAEAQHSVEAHQQRVLQLRAELQKTDAAE-RGVLDTAEKQLRELQLSLPPVLSYVQNGC--------------------ARQLEELRVH

*L. panamensis*  HRTRLEELKVQATEAQRSVEVHQWRVLQLRTELQKVEAAE-RGALDTAEQQLRQLQLSLPAVLSYVQNGC--------------------NQRLEGLRGH

*L. turanica*  HRTRVEELKRQASEAQHSVEAHQQRVLQLRAELQKTEAAA-RGALDTAEKQLRELQLSLPPVLSYLQNGC--------------------ARQLEELRMH

*L. tropica*  HRTRVEELKRQAAEAQHSVEAHQQRVLQLRAELQKTDAAE-RGALDTAEKQLRELQLSLPPVLSYVQNGC--------------------ARQLEELRVH

*L. major*  HRTRVEELKRQAAEAQHSVEAHQQRVLQLRAERQKTEAAE-RGALDTAEKQLRELQLSLPPVLSYLHNGC--------------------ARQLEELRMH

*L. mexicana*  HRTRVEELKRQAAEAQRSVEAHQQRVLQLRAEFQKTEAAE-RGALDTAETQLRELQLSLPPVLRYVQNGC--------------------ARQLEELRVH

*T. cruzi*  YRERIAVINAEQEGLRERLQRLQGRLDTLQQARQDAECAAQQGRIGKIEESMRMLAGIVPKMRDYFASKH--------------------GEQLFSAREQ

*T. brucei brucei*  YVSRIAEIDDQREKLVAQLESYNKELEALHAKRKELELASQQGYIGQLKRTLGLLSAVLPRLRDYITSRV--------------------GEELSRDRES

*T. brucei gambiense* YVSRIAEIDDQREKLVTQLESYNKELEALHAKRKELELASQQGYIGQLKRTLGLLSAVLPRLRDYITSRV--------------------GEELSRDRES

*T. evansi*  YVSRIAEIDDQREKLVTQLESYNKELEALHAKRKELELASQQGYIGQLKRTLGLLSAVLPRLRDYITSRV--------------------GEELSRDRES

*T. congolense*  YVGRVADIDAKNEKATEVLKGLSAELEVLHKKREELEMSLNQGYVGQLKATAQLVSNIVPKLRDYFACRA--------------------AEQLSEARAS

1310 1320 1330 1340 1350 1360 1370 1380 1390 1400

....|....|....|....|....|....|....|....|....|....|....|....|....|....|....|....|....|....|....|....|

jaculum1 VAEGEAALARASEAAAVAARERDAAAASRDAASRACVQLSRRIALATLRDALAADAAQLAQVTAQLRELRRGRVPQLEQLLGADVVARETVPQLRERVRV

jaculum2 LEQAHERLVQVKRDVCRLMQSVVELRSRLKSHEQTLEVLRVQLEHVQLQEALAADTTRLTEVRNSMDEMKAKQIPNIHHLVG-DAAQVSSLVQLRDIVRN

*C. fasciculata*  LRDAESTCQATAQEMEGLSRRVEEARRTLGDQHRRSADMDRHIDLLQQQETIKADEARLVELEQSLVSLKTERLQGVEQLLG-DAATHATLSSLRERITA

*L. pyrrhocoris*  LREAEKACTATVQELDGLMTHMEDARRTLSDQHRRSADMDRHIEALQQQQSIALDETHLAETAQILVSLKTDRLQGVEQLVG-EEARQASLSTLRELITA

*L. seymouri*  LREAENSYKATMQELDGLTKSMEEARHTLSDQHRLSADMDRHIEALQQQQSIAVDEKHLAEIEGVLGSLKTDQLQGVEQLLG-EEAKQASLSTLREMITA

*L. arabica*  LCETESAYKASAQEEEGLASRMKEARKTLSDQHRRSTDMDRHIDVLQQEASIAADEAHLAEMERTLASLKSDRLHDVEQLLG-EEARQASLATLREMITA

*L. aethiopica*  LRETESAYKAAAQEEEGLASRMKEARKTLSDQHRRSADMDRHIDVLQQEASIAADEAHLAETERTLASLKSDRLQDVEQLLG-EEARQASLATLREMITA

*L. donovani*  LRETESAYKAAAQEEEGLASRMKEARKTLSDQHRRSAEIDRHIDILQQEASIAADEAHLAETERTLASLKSDRLQDVEQLLG-EEARHASLATLRELITA

*L. gerbilli*  LRETESAYKASAQEEEGLASRMKEARKTLSDQHRRSADMDRHIDVLQQEASIAADEAHLAEMERTLASLKSDRLHDVEQLLG-EEARQASLATLREMITA

*L. enriettii*  LRDTESAYKAAAQEEEELARRMREARQTLSDQHRRSVEMDRHIEALQQEASIAADEAHLAEAERTLASLKSDRLHGVEQLLG-EEAKQASLVTLREMITA

*L. infantum*  LRETESAYKAAAQEEEGLASRMKEARKTLSDQHRRSAEIDRHIDILQQEASIAADEAHLAETERTLASLKSDRLQDVEQLLG-EEARHASLATLRELITA

*L. panamensis*  LRETESAYEAATQEEEELASRINEARTTLSDQHRRSADMDRQIDVLQQAVSIAADEARLTETERTLASLKSDRLHDVEQLLG-EGAGQASLAALREMITA

*L. turanica*  LRETESAYKASAQEEEGLASRMKEARKTLSDQHRRSADMDRHIDVLQQEASIAADEAHLAEMERTLASLKSDRLHDVEQLLG-EEARQASLATLREMITA

*L. tropica*  LRETESAYKAAAQEEEGLASRMKEARKTLSDQHRRSADMDRHIDVLQQEASIAADEAHLAETERTLASLKSDRLHDVEQLLG-EEARQASLATLREMITV

*L. major*  LRETESAYKASAQEEEGLASRMKEARKTLSDQHRRSADMDRHIDVLQQEASIAADEAHLAEMERTLASLKSDRLHDVEQLLG-KEARQASLATLREMITA

*L. mexicana*  LHETESAYKAAAQEEEGLASRMKEARETLSDQHRRSADMDRHIDVLQQEASIAADEAHLAETERTLASLKSDRLQEVEQLLG-KEARQASLATLREMITA

*T. cruzi*  LQLAETSRLRAVDEVRQLREAIQESRRVVDEQHRQAAEVGKHIEAFEKRRSIDEDQARLQEVERSLTEMKSREIRGVAAILGADVIAGETVSRIRELIRE

*T. brucei brucei*  LCVNEKRRDTQAEEVKLLRLSIDDTLRIINEEQRLRVEVDKYIEYLEKKGSIEEDEKRLSDVRCTLSELKVNAVPAAEAVLGKDVVERESVGRIRELIRG

*T. brucei gambiense* LRVNEKRRDTQAEEVKLLRLSIDDTLRIINEEQRLRVEVDKYIEYLEKKGSIEEDEKRLSDVRCTLSELKVNAVPAAEAVLGKDVVERESVGRIRELIRG

*T. evansi*  LRVNEKRRDTQAEEVKLLRLSIDDTLRIINEEQRLRVEVDKYIEYLEKKGSIEEDEKRLSDVRCTLSELKVNAVPAAEAVLGKDVVERESVGRIRELIRG

*T. congolense*  LHDGEASCAAAVNEITSLRSAIDELLRGMEEERRMQSQLSKQIEYLEKQKAIEEDEARLSELVQVITKLKLKGVPGAEAILGRDVVEREGIARIRELIRA

1410 1420 1430 1440 1450 1460 1470 1480 1490 1500

....|....|....|....|....|....|....|....|....|....|....|....|....|....|....|....|....|....|....|....|

jaculum1 RCAELERSRALREGNVEAMLQDVASVRAQLARDKYNDVERRYRAVFVKLHTTEMAAKDIDKYYGALEKAVQSYHQEKIAQINELIAELWRQTYRGSDIDV

jaculum2 KINELERTKAQRDGNIEAMLQDMGHLKSQLAKEKYQNIEKRYRSVFLKVETTEIAVKDIDKYYVALEKAVQSYHQEKIAQINQIIAELWRQTYRGSDIDT

*C. fasciculata*  KMTALEKVRAQQDGNMEAMLLDVGQLKQQLRGDKYQSIEKRYRSTFLKVQTTEISIQDIEKYYSALEKAVQSYHQEKITQINQIIAELWRQTYRGSDIDT

*L. pyrrhocoris*  KMTSLEKIRAQQDGNMEAMLLDVAQLKQQLRSDKYQSIEKRYRSTFLKVQTTEISIQDIEKYYSALEKAVQSYHQEKIAQINQIIAELWRQTYRGSDIDT

*L. seymouri*  KVTSLEKIRAQHDGSMEAILVDVAQLKQQLRSDKYRSIEKRYRSTFLKVQTTEISIQDIEKYYGALEKAVQSYHQEKIAQINQIIAELWRQTYRGSDIDT

*L. arabica*  KITSLEKIRAQQDGNTEAMLLDVNQLKQQLRGDKYQNIEKRYRSTFLKVQTTEISIQDIEKYYSALEKAVQSYHQEKIAQINQIIAELWRRTYRGSDIDT

*L. aethiopica*  KITSLEKIRAQQDGNTEAMLLDVNQLKQQLRGDKYQNIEKRYRSTFLKVQTTEISIQDIEKYYSALEKAVQSYHQEKIAQINQIIAELWRRTYRGSDIDT

*L. donovani*  KITSLEKIRAQQDGNTEAMLLDVNQLKQQLRGDKYQNIEKRYRSTFLKVQTTEISIQDIEKYYSALEKAVQSYHQEKIAQINQIIAELWRQTYRGSDIDT

*L. gerbilli*  KITSLEKIRAQQDGNTEAMLLDVNQLKQQLRGDKYQNIEKRYRSTFLKVQTTEISIQDIEKYYSALEKAVQSYHQEKIAQINQIIAELWRRTYRGSDIDT

*L. enriettii*  KMTSLERIRAQQDGNMEAMLLDVNQLKQQLRGDKYRNIEKRYRSTFLKVQTTEISIQDIEKYYGALEKAVQSYHQEKIAQINQIIAELWRQTYRGSDIDT

*L. infantum*  KITSLEKIRAQQDGNTEAMLLDVNQLKQQLRGDKYQNIEKRYRSTFLKVQTTEISIQDIEKYYSALEKAVQSYHQEKIAQINQIIAELWRQTYRGSDIDT

*L. panamensis*  KITSLEKIRAQQDGNTEAMLLDVSQLKQQLRGDKYQNIEKRYRSTFLKVQTTEISIQDIEKYYSALEKAVQSYHQEKITQINQIIAELWRLTYRGSDIDT

*L. turanica*  KITSLEKIRAQQDGNTEAMLLDVNQLKQQLRGDKYQNIEKRYRSTFLKVQTTEISIQDIEKYYSALEKAVQSYHQEKIAQINQIIAELWRRTYRGSDIDT

*L. tropica*  KITSLEKIRAQQDGNTEAMLLDVNQLKQQLRGDKYQNIEKRYRSTFLKVQTTEISVQDIEKYYSALEKAVQSYHQEKIAQINQIIAELWRRTYRGSDIDT

*L. major*  KITSLEKIRAQQDGNTEAMLLDVNQLKQQLRGDKYQNIEKRYRSTFLKVQTTEISIQDIEKYYSALEKAVQSYHQEKIAQINQIIAELWRRTYRGSDIDT

*L. mexicana*  KITSLEKIRAQQDGNTEAMLLDVNQLKQQLRGDKYQNIEKRYRSTFLKVQTTEISIQDIEKYYSALEKAVQSYHQEKIAQINQIIAELWRQTYRGSDVDT

*T. cruzi*  KVSELERSRAQQEGNVEAMMQDITNLRSQLAREKYHDIEKRYRSTFLKVQTTEIAVADIEKYYRALEKAVQSYHQEKIAQINQILADLWRQTYKGSDIDT

*T. brucei brucei*  KISALECLRAQQDGVAEAMRQDIESLKGQLTRDKYKDIEKRYRTTFLKVQTTEIAVSDVEKYYRALEKAVQTYHQEKIAQINQILADLWRHTYKGSDIDT

*T. brucei gambiense* KISALECLRAQQDGVAEAMRQDIESLKGQLTRDKYKDIEKRYRTTFLKVQTTEIAVSDVEKYYRALEKAVQTYHQEKIAQINQILADLWRHTYKGSDIDT

*T. evansi*  KISALECLRAQQDGVAEAMRQDIESLKGQLTRDKYKDIEKRYRTTFLKVQTTEIAVSDVEKYYRALEKAVQTYHQEKIAQINQILADLWRHTYKGSDIDT

*T. congolense*  RGGELERLGAQQDGVMEAMRQEITGLKAQLAREKFKDVEKRYRTTFLKAQTTDIAVADVEKYYRALEKAVQTYHQEKIAQINQILADLWRQTYKGSDIDT

1510 1520 1530 1540 1550 1560 1570 1580 1590 1600

....|....|....|....|....|....|....|....|....|....|....|....|....|....|....|....|....|....|....|....|

jaculum1 IEICSEADTTTTTTSSASGRRSYNYRVIMKRGGTAIDMRGRCSAGQRVLASVIIRLALSEAFCCDCGILALDEPTTNLDEDNARSLAEALHALIEARRAV

jaculum2 VEICSETEANTST----SARRSYNYRVIMRRGSSVIDMRGRCSAGQRVLACVIIRLALSEAFCCDCGILALDEPTTNLDEDNARSLAEALRALIETRRSI

*C. fasciculata*  VEIRSETESTTTT----AARRSYNYRVVMKRGNNEMDMRGRCSAGQKVLASIIIRLALSEAFCYDCGILALDEPTTNLDDDNSRSLADALRTLIEARRAV

*L. pyrrhocoris*  VEIRSETEGTTTT----AARRSFNYRVVMKRGNNEMDMRGRCSAGQKVLASIVIRLALSEAFCCDCGILALDEPTTNLDDDNARSLAAALRTLIQARRAV

*L. seymouri*  VEIRSETEGTTTT----AARRSYNYRVVMKRGSNEMDMRGRCSAGQKVLASIIIRLALSEAFCCDCGILALDEPTTNLDDDNARSLADALRTLIQARRAV

*L. arabica*  VEIRSETEGTTTT----TARRSYNYRVVMKRGNNEMDMRGRCSAGQKVLASIIIRLALSEAFCCDCGILALDEPTTNLDGDNARSLADALRTLIQARRAV

*L. aethiopica*  VEIRSETEGTTTT----TARRSYNYRVVMKRGNNEMDMRGRCSAGQKVLASIIIRLALSEAFCCDCGILALDEPTTNLDGDNSRSLADALRTLIQARRAV

*L. donovani*  VEIRSETEGTTTT----TARRSYNYRVVMKRGNNEMDMRGRCSAGQKVLASIIIRLALSEAFCCDCGILALDEPTTNLDDDNARSLADALRTLIQARRAV

*L. gerbilli*  VEIRSETEGTTTT----TARRSYNYRVVMKRGNNEMDMRGRCSAGQKVLASIIIRLALSEAFCCDCGILALDEPTTNLDGDNARSLADALRTLIQARRAV

*L. enriettii*  VEIRSETEGTTST----AARRSYNYRVVMKRANNEMDMRGRCSAGQKVLASIIIRLALSEAFCCDCGILALDEPTTNLDDDNARSLADALRTLIQARRAV

*L. infantum*  VEIRSETEGTTTT----TARRSYNYRVVMKRGNNEMDMRGRCSAGQKVLASIIIRLALSEAFCCDCGILALDEPTTNLDDDNARSLADALRTLIQARRAV

*L. panamensis*  VEIRSETEGTTTT----AARRSYNYRVVMKRGNNEMDMRGRCSAGQKALASIIIRLALSEAFCCDCGILALDEPTTNLDDDNARSLADALRTLIEVRRAV

*L. turanica*  VEIRSETEGTTTT----TARRSYNYRVVMKRGNNEMDMRGRCSAGQKVLASIIIRLALSEAFCCDCGILALDEPTTNLDGDNARSLADALRTLIQARRAV

*L. tropica*  VEIRSETEGTTTT----TARRSYNYRVVMKRGNNEMDMRGRCSAGQKVLASIIIRLALSEAFCCDCGILALDEPTTNLDGDNARSLADALRTLIEARRAV

*L. major*  VEIRSETEGTTTT----TARRSYNYRVVMKRGNNEMDMRGRCSAGQKVLACIIIRLALSEAFCCDCGILALDEPTTNLDGDNARSLADALRTLIQARRAV

*L. mexicana*  VEIRSETEGTTTT----AARRSYNYRVVMKRGNNEMDMRGRCSAGQKVLASIIIRLALSEAFCCDCGILALDEPTTNLDDDNARSLADALRTLIQARRAV

*T. cruzi*  VELRSEDDVTST-----TARRSYSYRVVMKRGNSEMDMRGRCSAGQKVLASVLIRLALSEAFCCDCGILALDEPTTNLDEDNARSLAESLRLLIENHRAV

*T. brucei brucei*  IELRSEDDVTST-----TARRSYSYRVVMKRGNSEMDMRGRCSAGQKVLASVLIRLALSEAFCCDCGILALDEPTTNLDEDNARSLAESLRMLIDSHRAV

*T. brucei gambiense* IELRSEDDVTST-----TARRSYSYRVVMKRGNSEMDMRGRCSAGQKVLASVLIRLALSEAFCCDCGILALDEPTTNLDEDNARSLAESLRMLIDSHRAV

*T. evansi*  IELRSEDDVTST-----TARRSYSYRVVMKRGNSEMDMRGRCSAGQKVLASVLIRLALSEAFCCDCGILALDEPTTNLDEDNARSLAESLRMLIDSHRAV

*T. congolense*  IELRSEDDVTST-----TVRRSYSYRVVMKRGNGEMDMRGRCSAGQRVLASVLIRLALSEAFCCDCGILALDEPTTNLDEDNARSLAESLRMLIDNHRTV

1610 1620 1630 1640 1650

....|....|....|....|....|....|....|....|....|....|.

jaculum1 KHFQLIVITHDEQFVRALGGHSLDRVYYVQKDREGAFSVIEERAFDQLFA-

jaculum2 KHFQLVIITHDEQFVRALGGQTLDRFYYVHKDREGAFSVIDERTFDQLFA-

*C. fasciculata*  KHFQLIVITHDEQFVRALGGQSLDKFFYVHKDREGAFSVIEERTFDQLFA-

*L. pyrrhocoris*  KHFQLIVITHDEQFVRALGGQSLDKFFYIHKDREGAFSVIEERTFDQLFA-

*L. seymouri*  KHFQLIVITHDEQFVRALGGQSLDKFFYIHKDREGAFSVIEERTFDQLFA-

*L. arabica*  KHFQLVVITHDEQFVRALGGQSLEKFYYIHKDREGAFSVIDERTFDQLFA-

*L. aethiopica*  KHFQLVVITHDEQFVRALGGQSLEKFYYVHKDREGAFSVIDERTFDQLFA-

*L. donovani*  KHFQLVVITHDEQFVRALGGQSLEKFYYVHKDREGAFSVIDERTFDQLFA-

*L. gerbilli*  KHFQLVVITHDEQFVRALGGQSLEKFYYVHKDREGAFSVIDERTFDQLFA-

*L. enriettii*  KHFQLVVITHDEQFVRALGGQSLEKFYYVHKDREGAFSVIDERTFDQLFA-

*L. infantum*  KHFQLVVITHDEQFVRALGGQSLEKFYYVHKDREGAFSVIDERTFDQLFA-

*L. panamensis*  KHFQLVVITHDEQFVRALGGQSLEKFYYVHKDREGAFSVIDERTFDQLFA-

*L. turanica*  KHFQLVVITHDEQFVRALGGQSLEKFYYVHKDREGAFSVIDERTFDQLFA-

*L. tropica*  KHFQLVVITHDEQFVRALGGQSLEKFYYVHKDREGAFSVIDERTFDQLFA-

*L. major*  KHFQLVVITHDEQFVRALGGQSLEKFYYVHKDREGAFSVIDERTFDQLFA-

*L. mexicana*  KHFQLVVITHDEQFVRALGGQSLEKFYYVLKDREGAFSVIDERTFDQLFA-

*T. cruzi*  KHFQLIVITHDEQFVRALGGQALDTFYYIHKDREGAFSVIEERTFDQLFAA

*T. brucei brucei*  KHFQLIVITHDEHFVRALGGQALDTFYYIHKDREGAFSVIEERTFDQLFAS

*T. brucei gambiense* KHFQLIVITHDEHFVRALGGQALDTFYYIHKDREGAFSVIEERTFDQLFAS

*T. evansi*  KHFQLIVITHDEHFVRALGGQALDTFYYIHKDREGAFSVIEERTFDQLFAS

*T. congolense*  KHFQLIVITHDEHFVRALGGQALDTFYYIHKDREGAFSVIEERTFDQLFAS

**D) DNA topoisomerase III**

10 20 30 40 50 60 70 80 90 100

....|....|....|....|....|....|....|....|....|....|....|....|....|....|....|....|....|....|....|....|

jaculum1 MVAQQQRQVWLNVAEKPSVAKTLAATLSRGQCRSERTASPYNPLFRFAYEGVDMLVTSVTGHLMEYKFADETRDWVHYPVHELFRAPLRKTLRESCAALG

jaculum2 ------MTVWLNVAEKPSVAKEIAHTLSRGSARTISSKSRYNPVFEFTFDNHTMLVTSVAGHLLNIDFVPSARSWHSFPFKELFGVEIVKSVKSDLKPVQ

*C. fasciculata*  ------MPTWLNVAEKPSVAKEMAQSLSGGSCRTIASLSRFNPVFEFAFEGKTMLVTSVAGHLMEDQFPPHTKSWTGFPFQGLFTAQITKYVRADLEPVK

*L. pyrrhocoris*  ------MPTWLSVAEKPSVAKELAHSLCGGNCHTIASLSRFNPVFEFSFEGKTMLVTSVSGHLMEDQFPPNTKNWASYPFQGLFSAKITKYVRADLEPVK

*L. seymouri*  ------MPTWLNVAEKPSVAKEMAHCLSGGSCRTIASMSRFNPVFEFNFEGKSMLVTSVAGHLMEDQFPPNTKSWASFPFQGLFSATITKYVRSDLEPVK

*L. arabica*  ------MPTWLNVAEKPSVAKEIAQSLSGGNCRTVPSQSRFNPVYEFKFEGKTMLVTSVAGHLMEDQFPPNTKNWSTYPFQGLFSAPITKYVRADLEPVK

*L. aethiopica*  ------MPTWLNVAEKPSVAKEMAQSLSGGSYRTVPSQSRFNPVYEFKFEGKTMLVTSVAGHLMEDQFPPNTKNWSTYPFQGLFSAPITKYVRADLEPVK

*L. donovani*  ------MPTWLNVAEKPSVAKEMAQSLSGGNCRTVPSQSRFNPVYEFKFEGKTMLVTSVAGHLMEDQFPPNTKNWSTYPFQGLFLAPITKYVRADLEPVK

*L. gerbilli*  ------MPTWLNVAEKPSVAKEMAQSLSGGNCRTVPSQSRFNPVYEFKFEGKTMLVTSVAGHLMEDQFPPNTKNWSTYPFQGLFSAPITKYVRADLEPVK

*l. enriettii*  ------MPTWLNVAEKPSVAKEMAQSLSGGNCRTVQSQSRFNPVFEFSFEGKTMLVTSVAGHLMEDQFPPHTKNWSTYPFQGLFSAPITKYVRADLEPVK

*L. infantum*  ------MPTWLNVAEKPSVAKEMAQSLSGGNCRTVPSQSRFNPVYEFKFEGKTMLVTSVAGHLMEDQFPPNTKNWSTYPFQGLFSAPITKYVRADLEPVK

*L. panamensis*  ------MPTWLNVAEKPSVAKEMAQSLSGGHCRTVQSQSRFNPVFEFNFEGKTMLVTSVVGHLMEDQFPPNTKNWSTYPFQGLFSAPISKYVRTDLEPVK

*L. tropica*  ------MPTWLNVAEKPSVAKEMAQSLSGGSYRTVPSQSRFNPVYEFKFEGKTMLVTSVAGHLMEDQFPPNTKNWSTYPFQGLFSAPITKYVRADLEPVK

*L. turanica*  ------MPTWLNVAEKPSVAKEMAQSLSGGNCRTVPSQSRFNPVYEFKFEGKTMLVTSVAGHLMEDQFPPNTKNWSTYPFQGLFSAPITKYVRADLEPVK

*L. major*  ------MPTWLNVAEKPSVAKEMAQSLSGGNCRTVPSQSRFNPVYEFKFEGKTMLVTSVAGHLMEDQFPPNTKNWSTYPFQGLFSAPITKYVRADLEPVK

*L. mexicana*  ------MPTWLNVAEKPSVAKEMAQSLSGGNCRTAQSQSRFNPVYEFKFEGKTMLVTSVAGHLMEDQFPPNTKNWSTYPFQGLFSAPITKYVRADLEPVK

*T. cruzi*  ------MPTWLNVAEKPSVAKELSFVLSNGTCRMGNSLSRFNPVFEFDFEGKQMLCTSVAGHLMNDEFPPETRSWQSYPLVNLFTASITKHVKPEMEPVK

*T. brucei brucei*  ------MTIWLNVAEKPSVAREIATILSGGNQRTIRSHSIYNPVFEFNFEGKTMICTSVSGHLMESDFPPQAAGWASFPIIDLFTLGTVKRVRSDLSEIQ

*T. brucei gambiense* ------MTIWLNVAEKPSVAREIATILSGGNQRTIRSHSIYNPVFEFNFEGKTMICTSVSGHLMESDFPPQAAGWASFPIIDLFTLGTVKRVRSDLSEIQ

*T. evansi*  ------MTIWLNVAEKPSVAREIATILSGGNQRTIRSHSIYNPVFEFNFEGKTMICTSVSGHLMESDFPPQAAGWASFPIIDLFTLGTVKRVRSDLSEIQ

*T. congolense*  ------MCTWLNVAEKPSVAKEISFILSNGTYRSTATHSRYNPVFEFDMNGKHMLCTSVKGHLMTDEFPPQAKNWTNYPIQELFTATLTRHVKPEMSAVK

110 120 130 140 150 160 170 180 190 200

....|....|....|....|....|....|....|....|....|....|....|....|....|....|....|....|....|....|....|....|

jaculum1 DNVRRYAREADTLVLWLDCDREGENICFEVVELACQAN----RRLRVLRAHFSTLTERELVHAVQHLRPPDQRLSEAVDARQEMDLRVGAAFTRYQTVRF

jaculum2 DNLKSLAKRATTLVLWLDCDREGENIAFEVMEIVLSSSSTMTNRMEIKRAHFSALTERDLFFAVRHLKSPDRWLSDAVEARQEMDLRIGATFTRFLTIKF

*C. fasciculata*  RNLEALAGRANTLVLWLDCDREGENICFEVLQVVQGKR----PRVQVKRAHFSALTSRDLLHAVHNLQAPNKRLSDAVEARQEMDLRVGAAFTRYQTVKF

*L. pyrrhocoris*  KNLEALASRADTLVLWLDCDREGENICFEVMQVVQSKR----PRVQVKRAHFSALTSRDLLHAVHNLQAPNKRLSEAVEARQEMDLRIGAAFTRYQTVKF

*L. seymouri*  KNLEALACRADTLVLWLDCDREGENICFEVIQVVQSKR----PRVQIKRARFSALTARDLLHAVHNLQAPNKRLSDAVEARQEMDLRIGAAFTRYQTVKF

*L. arabica*  KNLEALAGRADTLVLWLDCDREGENICFEVMQVVQSKR----PQVQVKRAHFSALTARDLLNAVHNLKLPDKRLSDAVEARQEMDLRIGAAFTRYQTVKF

*L. aethiopica*  KNLEALAGRADTLVLWLDCDREGENICFEVMQVVQSKR----PQVQVKRAHFSALTARDLLNAVHNLKLPDKRLSDAVEARQEMDLRIGAAFTRYQTVKF

*L. donovani*  KNLEALAGRADTLVLWLDCDREGENICFEVMQVVQSKR----PQVQVKRAHFSALTARDLLNAVHNLKLPDKRLSDAVEARQEMDLRIGAAFTRYQTVKF

*L. gerbilli*  KNLEALAGRADTLVLWLDCDREGENICFEVIQVVQSKR----PQVQVKRAHFSALTARDLLNAVHNLKLPDKRLSDAVEARQEMDLRIGAAFTRYQTVKF

*l. enriettii*  KNLEALASRADALILWLDCDREGENICFEVMQVVQSKR----PQVTVKRARFSALTARDLLNAVHSLKLPDKRLSDAVEARQEMDLRIGAAFTRFQTVNF

*L. infantum*  KNLEALAGRADTLVLWLDCDREGENICFEVMQVVQSKR----PQVQVKRAHFSALTARDLLNAVHNLKLPDKRLSDAVEARQEMDLRIGAAFTRYQTVKF

*L. panamensis*  KNLEALATRADALILWLDCDREGENICFEVMQVVQSKR----PQLQVKRAHFSALTARDLLNAVHNLKLPDKRLSDAVEARQEMDLRIGATFTRYQTVKF

*L. tropica*  KNLEALAGRADTLVLWLDCDREGENICFEVMQVVQSKR----PQVQVKRAHFSALTARDLLNAVHNLKLPDKRLSDAVEARQEMDLRIGAAFTRYQTVKF

*L. turanica*  KNLEALAGRADTLVLWLDCDREGENICFEVMQVVQSKR----PQVQVKRAHFSALTARDLLNAVHNLKLPDKRLSDAVEARQEMDLRIGAAFTRYQTVKF

*L. major*  KNLEALAGRADTLVLWLDCDREGENICFEVMQVVQSKR----PQVQVKRAHFSALTARDLLNAVHNLKLPDKRLSDAVEARQEMDLRIGAAFTRYQTVKF

*L. mexicana*  KNLEALASRAETLVLWLDCDREGENICFEVMQVVQSKR----PQVQVKRAHFSALTARDLLNAVHNLKLPDKRLSDAVEARQEMDLRIGAAFTRYQTVKF

*T. cruzi*  KNLETLGRRASNLVLWMDCDREGENICFEVMQVVQGVN----RSINICRAHFSALTRRDLLSAVRSLKAPNKALSDAVEARQEIDLRIGAVFSRFQTIKF

*T. brucei brucei*  KNLRELGRRATTLALWMDCDREGENICFEVIDVVQKEH----SSISICRAHFSALTKRDILGAMRNLKAPNRALSEAVEARQELDLRLGAVFTRFQTLKF

*T. brucei gambiense* KNLRELGRRATTLALWMDCDREGENICFEVIDVVQKEH----SSISICRAHFSALTKRDILGAMRNLKAPNRALSEAVEARQELDLRLGAVFTRFQTLKF

*T. evansi*  KNLRELGRRATTLALWMDCDREGENICFEVIDVVQKEH----SSISICRAHFSALTKRDILGAMRNLKAPNRALSEAVEARQELDLRLGAVFTRFQTLKF

*T. congolense*  RNLETLGRRATTLGLWMDCDSEGENICFEVINVVSRVN----SRIKICRAVFSALTKRDIFRAMENLRQPNRAVADAVDARQEIDLRVGAVFSRFQTLRF

210 220 230 240 250 260 270 280 290 300

....|....|....|....|....|....|....|....|....|....|....|....|....|....|....|....|....|....|....|....|

jaculum1 RSAFAG-LSEVLSFGPCQFPTLGFVVRRYWQQRGFVAEDYYTLRLQHEGCVFSSTRGAMYDAVAATLLFDDMCASASAASAATTQGGTSTAAHCSSGGGG

jaculum2 RRIFGESSKEVLSFGPCQFPTLGFVVRRYWEQRGFVAEDFYNLMLEHRVTKFKSSRGRIFDQITATLIYHDMCELAQCDD-------------------N

*C. fasciculata*  RHLFDE-IPGVLSFGPCQFPTLGFVVRRYWQQQGFVPEDFFTLHLQHGETKFHSSRGSMYDQVAATLLYEDMLRSAAEEG-------------------H

*L. pyrrhocoris*  RHLFEE-IPGVLSFGPCQFPTLGFIVRRHWQQQGFVPEEFFTLSVQHGDTKFHSSRGSMYDQVAATLVYESMLQTAAEEG-------------------H

*L. seymouri*  RHLFEE-IPGVLSFGPCQFPTLGFVVRRYWQQQGFVPEEFYTLNLQHGDTKLHSSRGSMYDQVAATLVYEDMLQVAAEED-------------------H

*L. arabica*  RHLFAA-IPGVLSFGPCQFPTLGFVVRRYWQQQGFVPEDFFTLQLQHGDTKFHSSRGSMYDQVAATLIYEDMLLAAAAEG-------------------H

*L. aethiopica*  RHLFAA-IPGVLSFGPCQFPTLGFVVRRYWQQQGFIPEDFFTLQLQHGDTKFHSSRGSMYDQVAATLIYEDMLQAAAAEG-------------------H

*L. donovani*  RHLFAA-IPGVLSFGPCQFPTLGFVVRRYWQQQGFVPEDFFTLQLQHGDTKFHSSRGSMYDQVAATLIYEDMLQAAAAEG-------------------H

*L. gerbilli*  RHLFAA-IPGVLSFGPCQFPTLGFVVRRYWQQQGFVPEDFFTLQLQHGDTKFHSSRGSMYDQVAATLIYEDMLLAAAAEG-------------------H

*l. enriettii*  RHLFAE-IPGVLSFGPCQFPTLGFVVRRHWQQQGFVPEDFFTLHVQHGDITFYSSRGSLYDQVAATLIYEDMLQMAAAEG-------------------H

*L. infantum*  RHLFAA-IPGVLSFGPCQFPTLGFVVRRYWQQQGFVPEDFFTLQLQHGDTKFHSSRGSMYDQVAATLIYEDMLQAAAAEG-------------------H

*L. panamensis*  RHLFAE-VPGVLSFGPCQFPTLGFVVRRYWKQQGFVPEDFFTLQVQHGDTKFYSSRGSLYDQVAATLIYEDMLQAAAAEE-------------------H

*L. tropica*  RHLFAA-IPGVLSFGPCQFPTLGFVVRRYWQQQGFVPEDFFTLQLQHGDTKFHSSRGSMYDQVAATLIYEDMLQAAAAEG-------------------H

*L. turanica*  RHLFAA-IPGVLSFGPCQFPTLGFVVRRYWQQQGFVPEDFFTLQLQHGDTKFHSSRGSMYDQVAATLIYEDMLLAAAAEG-------------------H

*L. major*  RHLFAA-IPGVLSFGPCQFPTLGFVVRRYWQQQGFVPEDFFTLQLQHGDTKFHSSRGSMYDQVAATLIYEDMLLAAAAEG-------------------H

*L. mexicana*  RHLFAA-IPGVLSFGPCQFPTLGFVVRRYWQQQGFVPEDFFTLQLQHGDTKFHSSRGSMYDQVAATLIYEDMLQAAAADG-------------------H

*T. cruzi*  RDAFSG-MPRVLSFGPCQIPTLGFVVRRHREREGFVQEDFFTLVMRHGQTVFHGCRGSVFDQIAATLVLEMMLESAGPNA--------------------

*T. brucei brucei*  CSQFKN-FPKVLSFGPCQFPTLGFLVQRGWEQKGFVPEDYFTLTLQHGDTSFNSVRGPIYDKIAAAVVLQQMFDEARTRP--------------------

*T. brucei gambiense* CSQFKN-FPKVLSFGPCQFPTLGFLVQRGWEQKGFVPEDYFTLTLQHGDTSFNSVRGPIYDKIAAAVVLQQMFDEARTRP--------------------

*T. evansi*  CSQFKN-FPKVLSFGPCQFPTLGFLVQRGWEQKGFVPEDYFTLTLQHGDTSFNSVRGPIYDKIAAAVVLQQMFDEARTRP--------------------

*T. congolense*  RDMFSG-MPRVLSFGPCQIPTLGFVVRRSWERKGFVPEDYFTLVLRHNNTTFNSLRGSMYDLIAATLVFETMLEEARETP--------------------

310 320 330 340 350 360 370 380 390 400

....|....|....|....|....|....|....|....|....|....|....|....|....|....|....|....|....|....|....|....|

jaculum1 VARVVRVHKQPRTRRPPEPLATVALQKLAATHLAMSSEQCMSLAEALYQEGYISYPRTETDAFSFDDATLLELVRVQLDNPDVADYARALLASTQGTATS

jaculum2 KAMVLHVTQHENKRHRPVPLNTVMLQKLAASHLHLPAAECMSIAEKLYQEGFISYPRTETDSFTFTQSELLNLVRLQTSNPVVGEYASTML---------

*C. fasciculata*  KGRVTSVQQRPSHRRPPVPLATVVMQKLAATHLRIPSERCMTLAESLYQEGLISYPRTETDSYTFQANELLDLVRVQTANADVADYAAAMV---------

*L. pyrrhocoris*  TGRITDVQQRPSHRRPPVPLATVVMQKLAATHLRISSERCMTLAESLYQEGLISYPRTETDSYTFQQNELLDLVRAQTSNADVADFVHAML---------

*L. seymouri*  KGRITNVQQRPSHRRPPVPLATVVMQKLAATHLRIPSERCMTLAESLYQEGLISYPRTETDSYTFQQGELLDLIRVQTSNADVADYANAML---------

*L. arabica*  KGRITNVQQRPSHRRPPVPLATVVMQKLAATHLRIPSERCMTLAESLYQEGLISYPRTETDSYTFQESELLELVRVQISNADVAEYATSML---------

*L. aethiopica*  KGRITNVQQRPSHRRPPVPLATVVMQKLAATHLRITSERCMTLAESLYQEGLISYPRTETDSYTFQESELLELVRVQIANADVAEYATAML---------

*L. donovani*  KGRITNVQQRPSHRRPPVPLATVVMQKLAATHLRIPSERCMTLAESLYQEGLISYPRTETDSYTFQESELLELVRVQIANADVAEYVAAML---------

*L. gerbilli*  KGRITNVQQRPSRRRPPVPLATVVMQKLAATHLRIPSERCMTLAESLYQEGLISYPRTETDSYTFQESELLELVRVQISNADVAEYATAML---------

*l. enriettii*  KGRITNVQQRPMHRRPPVPLATVVMQKLAATHLRIPSERCMTLAESLYQEGLISYPRTETDSYTFKEGELLELVRVQVANADVAEYVNAML---------

*L. infantum*  KGRITNVQQRPSHRRPPVPLATVVMQKLAATHLRIPSERCMTLAESLYQEGLISYPRTETDSYTFQESELLELVRVQIANADVAEYVAAML---------

*L. panamensis*  KGHITNVQQRPSRRRPPVPLATVIMQKLAATHLRISSERCMTLAESLYQEGLISYPRTETDSYTFQESELLELIRLQIANPDVAEYATAML---------

*L. tropica*  KGRITNVQQRPSHRRPPVPLATVVMQKLAATHLRIPSERCMTLAESLYQEGLISYPRTETDSYTFQESELLELVRVQTANADVAEYATAML---------

*L. turanica*  KGRITNVQQRPSHRRPPVPLATVVMQKLAATHLRIPSERCMTLAESLYQEGLISYPRTETDSYTFQESELLELVRVQISNADVAEYATAML---------

*L. major*  KGRITNVQQRPSHRRPPVPLATVVMQKLAATHLRIPSERCMALAESLYQEGLISYPRTETDSYTFQESELLELVRVQISNADVAEYATAML---------

*L. mexicana*  KGRITNVQQRPSHRRPPVPLATVVMQKLAATHLRITSERCMTLAESLYQEGLISYPRTETDSYTFQESELLELVRVQTANADVAEYATAML---------

*T. cruzi*  EALVVDVVRRPTRRHPPVPLATVALQKLAATHLHISSEQCMTWAESLYQEGFISYPRTETDSFSFTDDELREIAGVQRDNPEVADYVGAML---------

*T. brucei brucei*  EAEVVEVMKRPNRRRPPFPLSTVMLQKLCTAHLRISSDQCMTWAESLYQEGYISYPRTETDSFSFTDNELLDIVGSQRRNPEVSGFVEAML---------

*T. brucei gambiense* EAEVVEVMKRPNRRRPPFPLSTVMLQKLCTAHLRISSDQCMTWAESLYQEGYISYPRTETDSFSFTDNELLDIVGSQRRNPEVSGFVEAML---------

*T. evansi*  EAEVVEVMKRPNRRRPPFPLSTVMLQKLCTAHLRISSDQCMTWAESLYQEGYISYPRTETDSFSFTDNELLDIVGSQRRNPEVSGFVEAML---------

*T. congolense*  EAEIVEVIEKREVRRRHVPLATVELQKLCASHLRITSEQCMMWAESLYQEGYLSYPRTETDSFTMTDEELLEIAALQSTNPEVSGFVDAML---------

410 420 430 440 450 460 470 480 490 500

....|....|....|....|....|....|....|....|....|....|....|....|....|....|....|....|....|....|....|....|

jaculum1 VSNNSSSSSAGATTAAAAEEQPRFRRPLRGCHDDKAHPPIHPTKAWAAAAAAT---------AGSNNNNNS-------------GTRDAAKARLYRLIVR

jaculum2 -----------------SDTTNKFRMPLRGGHDDHAHPPIHPTKLMMSLSINTSTGQEKNSESSASNNKKSNKRYKFNSDNKKNNEGNSSKNALYLLIVR

*C. fasciculata*  -----------------SDPATRVRQPLRGGHDDKAHPPIHPTKAWNA--------------------------------------SQDERGKLYNLIVR

*L. pyrrhocoris*  -----------------ADTATRVRPPLRGGHNDKAHPPIHPTKAWNA--------------------------------------TQEDRGKLYNLIVR

*L. seymouri*  -----------------SDTATRVRPPLRGGHDDKAHPPIHPTKAWNA--------------------------------------TQDDRGKLYNLIVR

*L. arabica*  -----------------ADIATRVRPPLRGGHDDKAHPPIHPTKAWNA--------------------------------------TQDERGKLYNLIVR

*L. aethiopica*  -----------------ADIATRVRPPLRGGHDDKAHPPIHPTKAWNA--------------------------------------TQDERGRLYNLIVR

*L. donovani*  -----------------ADIATRVRPPLRGGHDDKAHPPIHPTKAWNA--------------------------------------TQDERGRLYNLIVR

*L. gerbilli*  -----------------ADIATRVRPPLRGGHDDKAHPPIHPTKAWNA--------------------------------------TQDERGRLYNLIVR

*l. enriettii*  -----------------ADVPTRVRPPLRGGHDDKAHPPIHPTKAWNA--------------------------------------TPDERGRLYNLIVR

*L. infantum*  -----------------ADIATRVRPPLRGGHDDKAHPPIHPTKAWNA--------------------------------------TQDERGRLYNLIVR

*L. panamensis*  -----------------ADIAARVRPPLRGGHDDKAHPPIHPTKAWNA--------------------------------------TRDERGSLYNLIVR

*L. tropica*  -----------------ADIATRVRPPLRGGHDDKAHPPIHPTKAWNA--------------------------------------TQDERGRLYNLIVR

*L. turanica*  -----------------ADIPTRVRPPLRGGHDDKAHPPIHPTKAWNA--------------------------------------TQDERGRLYNLIVR

*L. major*  -----------------ADIATRVRPPLRGGRDDKAHPPIHPTKAWNA--------------------------------------TQDERGRLYNLIVR

*L. mexicana*  -----------------ADIATHVRPPLRGGHDDKAHPPIHPTKAWNA--------------------------------------TQDERGRLYNLIVR

*T. cruzi*  -----------------DDPSNKFRRPLKGGHDDKAHPPIYPTKLMRA--------------------------------------KQDSRGALYNLIVR

*T. brucei brucei*  -----------------ADPNRKFRRPLNGGHDDKAHPPIYPTKPMPA--------------------------------------ANDGRAKLYNLIVR

*T. brucei gambiense* -----------------ADPNRKFRRPLNGGHDDKAHPPIYPTKPMPA--------------------------------------ANDGRAKLYNLIVR

*T. evansi*  -----------------ADPNRKFRRPLNGGHDDKAHPPIYPTKPMPA--------------------------------------ANDGRAKLYNLIVR

*T. congolense*  -----------------RDPQNKYCRPLDGGHSDNAHPPIYPTKPLHV--------------------------------------KDDRRAPLYNFIVR

510 520 530 540 550 560 570 580 590 600

....|....|....|....|....|....|....|....|....|....|....|....|....|....|....|....|....|....|....|....|

jaculum1 HFLACVSSDAVAATTRVEVVYGDEPFSARGTAVLWRGWLAIFPYASWRDSATMPNYNDGDVFTPSSVTMHKSTTTPPPHLTETNLIALMDENGIGTDATI

jaculum2 HFLACLSIDAIGARTSVEVCFGGELFTTSGVTIVEEGWLAVYPYVNWNSSANIPNYKPGETFTPSKVHLEHGRTCPPQDLSETDLIALMDYNGIGTDATI

*C. fasciculata*  HFLASLSPDAVAATTRVTAEFGGELFSTGGTTIVQRGWLDIFPYERWNST-MIPNYQPNEAFEPTAVQLHKGSTTAPPHLTETNLITLMDSNGIGTDATI

*L. pyrrhocoris*  HFLASLSPDAVAATTRVSAEFGGEVFSAGGTTIVQRGWLDVFPYERWNNT-LIPNYQAGEAFEPSAVHLSKGCTAAPPHLSETNLISLMDSNGIGTDATI

*L. seymouri*  HFLASMSPDAVAATTRVSAEFGGELFTTGGTTIVQRGWLDIFPYERWNNT-LIPNYQVGEAFEPSAVHLSRGCTTAPPHLTETNLISLMDSNGIGTDATI

*L. arabica*  HYLASLSPDAVAATTQVSAEFGGELFSTGGTTILQRGWLDIFPYERWNST-CIPNYQIGDTFQPTAVPLKKGCTSAPPHLTETHLISLMDSNGIGTDATI

*L. aethiopica*  HFLASLSPDAVAATTRVSAEFGGELFSTGGTTILQRGWLDIFPYERWNST-CIPNYQIGDTFQPTAVPLKKGCTSAPPHLTETHLISLMDSNGIGTDATI

*L. donovani*  HFLASLSPDAVAATTQVSAEFGGELFSTGGTTILQRGWLDIFPYERWNNT-CIPNYQIGDTFQPTAVPLKKGCTSAPPHLTETHLISLMDSNGIGTDATI

*L. gerbilli*  HYLASLSPDAVAATTQVSAEFGGELFSTGGTTILQRGWLDIFPYERWNST-CIPNYQIGDTFQPTAVPLKKGCTSAPPHLTETHLISLMDSNGIGTDATI

*l. enriettii*  HFLASLSPDAVAATTRVSADLGGESFTTGGTTIVQRGWLDIFPYERWNNT-CIPNYQIGDTFEPTAVQLKKGCTTAPPHLTETHLISLMDSNGIGTDATI

*L. infantum*  HFLASLSPDAVAATTQVSAEFGGELFSTGGTTILQRGWLDIFPYERWNNT-CIPNYQIGDTFQPTAVPLKKGCTSAPPHLTETHLISLMDSNGIGTDATI

*L. panamensis*  HFLASLSPDAVAATTRVSAEFGGESFSTGGTTIVQRGWLDIFPYERWDST-CIPNYQIGDTFEPTTVLLKKGRTSAPPHLTESRLISLMDSNGIGTDATI

*L. tropica*  HFLASLSPDAVAATTQVSAEFGGELFSTGGTTILQRGWLDIFPYERWNST-CIPNYQIGDTFQPTAVPLNKGCTSAPPHLTETHLISLMDSNGIGTDATI

*L. turanica*  HYLASLSPDAVAATTQVSAEFGGELFSTGGTTILQRGWLDIFPYERWNST-CIPNYQIGDTFQPTAVPLKKGCTSAPPHLTETHLISLMDSNGIGTDATI

*L. major*  HFLASLSPDAVAATTQVSAEFGGELFSTGGTTILQRGWLDIFPYERWNST-CIPNYQIGDAFQPTAVPLKKGCTSAPPHLTETHLISLMDSNGIGTDATI

*L. mexicana*  HFLASLSPDAVAATTQVSAEFGGELFSAGGTTILQRGWLDIFPYERWNNT-CIPNYQIGDMFQPTAVPLKKGCTSAPPHLTETHLISLMDSNGIGTDATI

*T. cruzi*  HFLACLSPDAVAATTAVTAVFGEEKFTTSGTAILEKGWLEVYPYERWNST-FIPAYQQGERFVPKDISLEKHRTTPPPHLTETALIALMDEHGIGTDATI

*T. brucei brucei*  HFLACTSPDAVASTTSVAVVYGGEKFTTSGTTIDEKGWMEVYIYERWKST-TLPTYKQGERFRPTHADLEQHRTSPPPNLTEADLITLMDKHGIGTDATI

*T. brucei gambiense* HFLACTSPDAVASTTSVAVVYGGEKFTTSGTTIDEKGWMEVYIYERWKST-TLPTYKQGERFRPTHADLEQHRTSPPPNLTEADLITLMDKHGIGTDATI

*T. evansi*  HFLACTSPDAVASTTSVAVVYGGEKFTTSGTTIDEKGWMEVYIYERWKST-TLPTYKQGERFRPTHADLEQHRTSPPPNLTEADLITLMDKHGIGTDATI

*T. congolense*  YFLACISPDAVGATTSVTAVFGGEKFTTSGTVVLENGWMEVYPYKKWYSS-TIPLYKRGERFVPTDANLQKHRTSPPPNLTERDLIHLMNENGIGTDATI

610 620 630 640 650 660 670 680 690 700

....|....|....|....|....|....|....|....|....|....|....|....|....|....|....|....|....|....|....|....|

jaculum1 AQHIKTIQERHYVERTGGGGSGGGGMCFVPTRLGIALASAYEMVGLGSLLQPELRAQMELAMGDVVSGRATRAQVVEAAVRQYAAIFDMLVSHTQSFYAQ

jaculum2 AQHIDTILTRKYVERRGR--------SLIPTPLGLALASAYEVVGLGSLLQPFLRAQMELAMNDITSGNATREQVVYAAVRQYKDIFIQLEERQEFFYKE

*C. fasciculata*  AQHIKTVLDREYVKREGQ--------ALVPTRLGIALASAYEALGLGSLLQPQLRAQMELAMGDIANGGATKDQVVKAAVQLYEEIFSRLFASTGAFYEE

*L. pyrrhocoris*  AQHIKTVLDREYVRREGQ--------ALVPTRLGIALASAYEALGLGSLLQPQLRAQMELAMGDIANGGATKEQVVAAAVQLYEEIFSRLSASTAAFYEE

*L. seymouri*  AQHIKTVLDREYVKREGQ--------TLVPTQLGIALASAYEALGLGSLLQPQLRAQMELAMGDIANGSATKEQVVKAAVQLYEEIFSRLYASTSAFYEE

*L. arabica*  AQHIKTVLDREYVKREGQ--------SLVPTTLGIALASAYESLGLASLLQPHLRAQMELAMGDIANGAATKEQVVAAAVQLYEEIFSRLMANTNAFYEE

*L. aethiopica*  AQHIKTVLDREYVKREGQ--------SLVPTTLGIALASAYESLGLASLLQPQLRAQMELAMGDIANGAATKEQVVAAAVQLYEEIFSRLMANTNAFYEE

*L. donovani*  AQHIKTVLDREYVKREGQ--------SLVPTTLGIALASAYESLGLASLLQPQLRAQMELAMGDIANGAATKEQVVTAAVQLYEEIFSRLMANTNAFYEE

*L. gerbilli*  AQHIKTVLDREYVKREGQ--------SLVPTTLGIALASAYESLGLASLLQPQLRAQMELAMGDIANGAATKEQVVAAAVQLYEEIFSRLMANTNAFYEE

*l. enriettii*  AQHIKTVLDREYVKREGQ--------SLVPTTLGIALASAYEVLGLASLLQPQLRAQMELAMGDIANGAATKEQVVAAAVRLYEEIFKRLMASTNAFYEE

*L. infantum*  AQHIKTVLDREYVKREGQ--------SLVPTTLGIALASAYESLGLASLLQPQLRAQMELAMGDIANGAATKEQVVAAAVQLYEEIFSRLMANTNAFYEE

*L. panamensis*  AQHIKTVLDREYVRREGQ--------SLVPTTLGIALASAYESLGLASLLQPQLRAQMELAMGDIANGAATKAQVVAAAVQLYEEIFSRLMASTGAFYEE

*L. tropica*  AQHIKTVLDREYVKREGQ--------SLVPTTLGIALASAYESLGLASLLQPQLRAQMELAMGDIANGAATKEQVVAAAVQLYEEIFSRLMANTNAFYEE

*L. turanica*  AQHIKTVLDREYVKREGQ--------SLVPTTLGIALASAYESLGLASLLQPQLRAQMELAMGDIANGAATKEQVVAAAVQLYEEIFSRLMANTNAFYEE

*L. major*  AQHIKTVLDREYVKREGQ--------SLVPTTLGIALASAYESLGLASLLQPQLRAQMELAMGDIANGAATKEQVVAAAVQLYEEIFSRLMANTNAFYEE

*L. mexicana*  AQHIKTVLDREYVRREGQ--------SLVPTTLGIALASAYESLGLASLLQPQLRAQMELAMGDIANGAATKEQVVAAAVQLYEEIFSRLMANTNAFYEE

*T. cruzi*  AQHIKTVLDRGYVKREGS--------FLVPTALGIALASAYEVIGLLSLLQPQLRAQMELAMSDIVSGKASKAQVVDAAVKLYTEVFQKLSLGSKEMYEE

*T. brucei brucei*  SHHIKTVVEREYVKREGS--------SLVPTHVGNALASAYEVNGLVSLLQPQMRAQMELAMADIAAGKATRRDVVDAAVRLYREIFQKMLSLTDAMNRV

*T. brucei gambiense* SHHIKTVVEREYVKREGS--------SLVPTHVGNALASAYEVNGLVSLLQPQMRAQMELAMADIAAGKATRRDVVDAAVRLYREIFQKMLSLTDAMNRV

*T. evansi*  SHHIKTVVEREYVKREGS--------SLVPTHVGNALASAYEVNGLVSLLQPQMRAQMELAMADIAAGKATRRDVVDAAVRLYREIFQKMLSLTDAMNRV

*T. congolense*  PQHIKTILDREYVRREGS--------DLVPTPLGIALASAYEVIGLVSLLQPQLRAQTELAMDDIAKGRATKQDVVDASVRLYREIFQKLSNNSQEVYRE

710 720 730 740 750 760 770 780 790 800

....|....|....|....|....|....|....|....|....|....|....|....|....|....|....|....|....|....|....|....|

jaculum1 LAQHLQQSE----------SDNS----EGGGGAAVLPSTTTAAANNAANNSVHE-VM--RRAALTV-------DATVLHAAYTPCPTCG--PQHAMDMVD

jaculum2 LIKYIANEN----------ARN----------------------------------------------------YNVMNTNTTQYT--------------

*C. fasciculata*  LKRHLRPAE---GLAPAAATHDAAAASLGPATVVAAGLIACGRCGGPMDLVERQGER--DREVWGLRCGPCNTLHRVPNGRLNALEPLTPPHRCPLCGFA

*L. pyrrhocoris*  LKRHLGPAAAAGGDYRDATLADATAAVTGPATVVAAGLSGCGSCDRRMDLVECHGE----REVWAVRCDGCSKSYRVPNGRLNTLEPVTPPHRCPLCGFA

*L. seymouri*  LKRHLRPAA-AGGDRRDVASVAATAKVAGPATVVKPGLIACGMCGSLMDLVEHQGER--DREVWAVRCGRCSKSYRVPNCRLNKLEPLTPPQRCPLCGFE

*L. arabica*  LKRYLQPEV----------ETAACAV---QATVVKANFIPCGTCGRLMDLVERAGER--DREVWSVRCHACNKMHRVPNGRLNTLEPVSPPHTCPLCGFL

*L. aethiopica*  LKRYLQPEV----------ETDACAV---PATVVKANFIPCGTCGRLMDLVERAGER--DREVWSVRCHACDKMHRVPNGRLNTLEPVSPPHTCPLCGFL

*L. donovani*  LKRYLQPAV----------ETDACAV---QATVVKANFIPCGTCGRLMDLVERAGER--DREVWSVRCHACNKMYRVPNGRLNTLEPVSPPHTCPLCGFL

*L. gerbilli*  LKRYLQPEV----------ETDACAV---QATVVKANFIPCGTCGRLMDLVERAGER--DREVWSVRCHACNKMHRVPNGRLNTLEPVSPPHTCPLCGFL

*l. enriettii*  LKRHLRPAA----------EPDARAV---QVTVVKANFIPCGTCGQLMDLVERVGER--DREVWTVRCLACNKMHRVPNGRLNALEPVAPPLTCPICGFV

*L. infantum*  LKRYLQPAV----------ETDACAV---QATVVKANFIPCGTCGRLMDLVERAGER--DREVWSVRCHACNKMYRVPNGRLNTLEPVSPPHTCPLCGFL

*L. panamensis*  LKRHLRLAA----------ETDAHAV---QVTVVKDNFIQCGTCGRHMDLVERAGERDHDREVWSVRCRGCNKMHRLPNGRLNTLEPVNPPHTCPLCGFV

*L. tropica*  LKRYLQPEV----------ETDACAV---QATVVKANFIPCGTCGRLMDLVERAGER--DREVWSVRCHACNKMHRVPNGRLNTLEPVSPPHTCPLCGFL

*L. turanica*  LKRYLQPEV----------ETDACAV---QATVVKANFIPCGTCGRLMDLVERAGER--DREVWSVRCHACNKMHRVPNGRLNTLEPVNPPHTCPLCGFL

*L. major*  LKRYLQPEV----------ETDACAV---QATVVKANFIPCGTCGRLMDLVERAGER--DREVWSVRCHACNKMHRVPNGRLNTLEPVSPPHTCPLCGFL

*L. mexicana*  LKRYLQPAV----------ETDACAV---EATVVKANFIPCGTCGGPMDLVERAGER--DREVWSVRCHACNKMHRVPNGRLNTLEPVSPPHTCPLCGFL

*T. cruzi*  LCCHLSPAA----------PQTSGTHAYGTGRIDQRRLVHCGVCKNPMDLIEQA-EG--ERDAWCVRCHTCLKDYRVPNGRLNQLSSCG--QSCILCGFG

*T. brucei brucei*  LHLHLDPVY----------QTAVEEVGTSRAHISASGLVECGTCGNPMNLMEHV-QK--ERNPWFVRCDTCQKEYRVPNGRHNRIERSG--HRCVICKFG

*T. brucei gambiense* LHLHLDPVY----------QTAVEEVGTSRAHISASGLVECGTCGNPMNLMEHV-QK--ERNPWFVRCDTCQKEYRVPNGRHNRIERSG--HRCVICKFG

*T. evansi*  LHLHLDPVY----------QTAVEEVGTSRAHISASGLVECGTCGNPMNLMEHV-QK--ERNPWFVRCDTCQKEYRVPNGRHNRIERSG--HRCVICKFG

*T. congolense*  LYTRLSPVF----------GAGSLVIEGGPAKTSRRGLVHCGSCQNLMDLMEYT-DR--DHGSWVVRCATCAKEYRVPNARRNHLEPLS--QRCVICGFG

810 820 830 840 850 860 870 880 890 900

....|....|....|....|....|....|....|....|....|....|....|....|....|....|....|....|....|....|....|....|

jaculum1 VVD--TIVPNNSSSNSTHTATTAT---------------TAAAAASVVRHRRYVRCLHCRTAQAVDALPITRCTVCGVGDLCLRSTNTTATATTATATTN

jaculum2 -----NEENQNNSVL------LPP---------------P--LLLSSAGGRG-------ANSWDAESIAISRCMNCTDGELCFVQ---------------

*C. fasciculata*  VLRVTNKEKHTSYTVCPYCFTHPPDAATANA--------ALPDMETIGEFRCFQCLADCPLAKGLESLGITTCIACRQNELRLR----------------

*L. pyrrhocoris*  VLRVTNRDKQTSYTVCPYCFTHPPTAGSTSG--------VVPDMEATAEFRCFQCMADCPLAKGLESIGITPCIACRQNELRLR----------------

*L. seymouri*  VLRVTNREKQTSHTICPYCFSHPPASASVNG--------MLPDMETAGEFRCFQCTADCPLAKGLESIGITPCIACRQNELRLR----------------

*L. arabica*  ALRVANREKQTFYHVCPHCFGSPPKAWSVTAYGCAGAAASLPDVEAAAEFRCFQCTADCPLAKGLEAIGITTCIACRQHELRLR----------------

*L. aethiopica*  ALRVANREKQTSYHVCPHCFGSPPKTWSVTAYGSAGAAASLPDVEAAAEFRCFQCTADCPLAKGLEAIGITTCIACRQHELRLR----------------

*L. donovani*  ALRVANREKQTSYHVCPHCFGSPPKAWSVTAYGSAGAAAPLPDVEAAAEFRCFQCTADCPLAKGLEAIGITTCIACRQHELRLR----------------

*L. gerbilli*  ALRVANREKQTSYHVCPHCFGSPPKAWSVTAYGCAGAAASLPDVEAAAEFRCFQCTADCPLAKGLEAIGITTCIACRQHELRLR----------------

*l. enriettii*  ALRVVNREKQTSYHVCPHCFGSPPRAWTASGCSATCSAALLPDIEAVAEFRCFQCTADCPLAKGLETIGITTCIACHRHELRLR----------------

*L. infantum*  ALRVANREKKTSYHVCPHCFGSPPKAWSVTAYGSAGAAAPLPDVEAAAEFRCFQCTADCPLAKGLEAIGITTCIACRQHELRLR----------------

*L. panamensis*  ALRVTNREKQTSYHVCPHCFGSPPRAWNVTGCGTAGADALLPDIEAAAEFRCFQCTADCPLAKGLEAIGITTCIACHQHELRLR----------------

*L. tropica*  ALRVANREKQTSYHVCPHCFGSPPKAWSVTAYGSAGAAASLPDVEAAAEFRCFQCTADCPLAKGLEAVGITTCIACRQHELRLR----------------

*L. turanica*  ALRVANREKQTSYHVCPHCFGSPPKAWSVTAYGCAGAAASLPDVEAAAEFRCFQCTADCPLAKGLEAIGITTCIACRQHELRLR----------------

*L. major*  ALRVANREKQTSYHVCPHCFGSPPKAWSVTTYGCVGVAASLPDVEAAAEFRCFQCTADCPLAKGLEAIGITTCIACRQHELRLR----------------

*L. mexicana*  ALRVANREKQTSYHVCPHCFGSPPKAWSVSAKGAASAAAPFPDVEAAAEFRCFQCTADCPLAKGLEAIGITTCIACCQHELRLR----------------

*T. cruzi*  VLDIRNTEKQTSYTVCPYCFTSPP---------------PGTDMESFTGFRCFQCLADCPLAKGLESVAIARCSACKEHDIRLC----------------

*T. brucei brucei*  VLEITNIDKGTSHTVCPYCFTSPP---------------PGAEMESLAEFRCFHCGADCPLAKGHETVTITNCMSCKKNGLRIR----------------

*T. brucei gambiense* VLEITNIDKGTSHTVCPYCFTSPP---------------PGAEMESLAEFRCFHCGADCPLAKGHETVTITNCMSCKKNGLRIR----------------

*T. evansi*  VLEITNIDKGTSHTVCPYCFTSPP---------------PGAEMESLAEFRCFHCGADCPLAKGHETVTITNCMSCKKNGLRIR----------------

*T. congolense*  VLDVKNIEKQTSHTICVHCFSVPP---------------PGADIESSTEFRCFHCVANCPLAKGFDNVSITRCTACNEHDMRLR----------------

910 920 930 940 950 960 970 980 990 1000

....|....|....|....|....|....|....|....|....|....|....|....|....|....|....|....|....|....|....|....|

jaculum1 NNSNNTTFFLSCRQYPACRARVSLPRAATVLPC--PSQHCAHCGGVLLLFDFAG-VYGVPGVDRDDALCVLCDARMTEYIAFKTGV---VGNNSNNSSNS

jaculum2 --TNNSNVFLKCKRYPVCSTRVFFPRASRIEPCVNPVERCEICYAVKLVFEFAGSVQPVPGLERVEKLCVFCHPVMREYITIKRETKPAINNTSSNSEES

*C. fasciculata*  --SSAGGFFLSCKGYPGCALTVSLPTASSVKPC--PTQRCPTCNAVLLTFDFRG-VTGVPGLDVVDTICVSCDARIKDYITVKGLP-AAGAAAP------

*L. pyrrhocoris*  --ASSNGYFLSCKGYPSCSLLVSLPAATAVKPC--PTQRCPSCNAVLLTFDFRG-VQGVPGLNTVDTICVSCDARIKDYISVKGFP-AAGSNSAGAGGNG

*L. seymouri*  --ASSCGYFLSCKGYPTCNLIVSLPTATSVKPC--PTQRCPSCNAVLLIFDFRG-VQGVPGLSPVDTICVSCDARIKDYICVKGFP-AAGVSSADTDGND

*L. arabica*  --SGANGFFLSCRGYPSCHLRVSLPAAASVKPS--PSQRCPACSAVLLTFDFSG-RQGVPGLNMLDTICVRCDARIKDYIAVKGMP-TSGANSSAVPST-

*L. aethiopica*  --SGANGFFLSCRGYPSCHLRVSLPAAASVKPS--PSQRCPACSAVLLTFDFSG-RQGVPGLNMLDTICVRCDARIKDYIAVKGMP-TSGANSSAVPST-

*L. donovani*  --SGANGFFLSCRGYPSCHLRVSLPAAASVKPS--PSQRCPACSAVLLTFDFSG-RQGVPGLDMLDTICVRCDARIKDYIAVKGMP-TGGANSSAVPSS-

*L. gerbilli*  --SGANGFFLSCRGYPSCHLRVSLPAAASVKPS--PSQRCPACSAVLLTFDFSG-RQGVPGLNMLDTICVRCDARIKDYIAVKGMP-TSGANSSAVPST-

*l. enriettii*  --SGPNGFFLSCKGYPSCNLSVSLPAAASVKPS--PLQRCPACNAVLLTFDFSG-RQGVPGLNMLDTICIRCDARIKDYITVKGIP-MGNASSSAVPSS-

*L. infantum*  --SGANGFFLSCRGYPSCHLRVSLPAAASVKPS--PSQRCPACSAVLLTFDFSG-RQGVPGLDMLDTICVRCDARIKDYIAVKGMP-TGGANSSAVPSS-

*L. panamensis*  --SGPNGFFLSCRGYPSCHLSVSLPAAASVKPS--PSQRCPACNAVLLTFDFSG-RQGVPGLNMLDTICIRCDARIKDYITVKGFL-TGGASSSAMPSS-

*L. tropica*  --SGANGFFLSCRGYPSCHLRVSLPAAASVKPS--PSQRCPACSAVLLRFDFSG-RQGVPGLNMLDTICVRCDARIKDYIAVKGMP-TSGANSPAVPST-

*L. turanica*  --SGANGFFLSCRGYPSCHLRVSLPAAASVKPS--PSQRCPACSAVLLTFDFSG-RQGVPGLNMLDTICVRCDARIKDYIAVKGMP-TSGANSSAVPST-

*L. major*  --SGANGFFLSCRGYPSCQLRVSLPAAASVKPS--PSQRCPACSAVLLTFDFSG-RQGVPGLNMLDTICVRCDARIKDYIAVKGMP-TSGANSSAVPSA-

*L. mexicana*  --SGAKGFFLSCRGYPSCHLRVSLPAAASVKPS--PSQRCPACSAVLLTFDFSG-RQGVPGLNMLDAICVRCDARIKDYITVKGMP-TGDPNASAAPNS-

*T. cruzi*  --TGSRGAFLSCRGFPGCTFSVTLPWAKGVRPT--PTMRCASCQAVMLQFNFGG-MPSVPGLEEGDCVCVFCDGRLQDYISVKGGV-TRGHDHTTDREHA

*T. brucei brucei*  --SNNSGFFLACRGFPVCNFTIKLPPAERVSLA--YDARCPSCSAIMLKFDFGG-SPAVPGVQEGEKVCVFCDARMKEHIRTKGNN-VLRGSRSAPQER-

*T. brucei gambiense* --SNNSGFFLACRGFPVCNFTIKLPPAERVSLA--YDARCPSCSAIMLKFDFGG-SPAVPGVQEGEKVCVFCDARMKEHIRTKGNN-VLRGSRSAPQER-

*T. evansi*  --SNNSGFFLACRGFPVCNFTIKLPPAERVSLA--YDARCPSCSAIMLKFDFGG-SPAVPGVQEGEKVCVFCDARMKEHIRTKGNN-VLRGSRSAPQER-

*T. congolense*  --GGGIGNFLACRGFPHCTFCINLPRAKRVIPV--QGDRCGSCNAVLLQFEFGG-IQTVPGVVEGEKACVLCDTRLQEYITMKGGV-QQGGVRPA-----

1010 1020 1030 1040 1050 1060 1070 1080 1090 1100

....|....|....|....|....|....|....|....|....|....|....|....|....|....|....|....|....|....|....|....|

jaculum1 NSTSA----LTSGRADAVAATAAYTLPSVM-DADVAG--RGRVGHNSSSNSRRVSTRAGDVGSGTHTSDA-A----AAA------------AAQ------

jaculum2 NNTGSNADNISGNGGMEVATGEQYTLPSIS-LRAVNNIHQGAKPQNMRNNAQGKSNNNNN--NNNNNNNN-N----VMP------------TGT------

*C. fasciculata*  ----------------SGRETAAYTLPSVG-AT-----------------------------AAASGRRGRGGAGGGRG-GRGGG------GGGSSASDA

*L. pyrrhocoris*  ------------NGSNGGPSNAAYTLPSVV-A------------------------------AAASGRRG-G----GGG-KKSSA------GAAASVNKS

*L. seymouri*  ------------NNRKNASPSAAYTLPSVA-A------------------------------AAASGRRG-GGSGGGRGRGRSGGGYHDHAGTGASTFGS

*L. arabica*  ------------AASVASTASGTYTLPSVK-PS-----------------------------ARHSVRGG-G----QRG-GRGGD------AAA--ANTS

*L. aethiopica*  ------------AASVASTASGTYTLPSVK-PS-----------------------------ARRSVRGG-G----QRG-GRGGD------AAA--ANTS

*L. donovani*  ------------AASVASTASRTYTLPSVK-PS-----------------------------ARRSVRGG-G----QRG-GSGGD------AAA--ANTS

*L. gerbilli*  ------------AASVASTASGTYTLPSVK-PS-----------------------------ARRSIRGG-G----QRG-GRGGD------AAA--ANTS

*l. enriettii*  ------------AASVAITASETYTLPSVT-SS-----------------------------ARRNGRGR-G----QRG----GG------AAA--ANMA

*L. infantum*  ------------AASVASTASRAYTLPSVK-PS-----------------------------ARRSVRGG-G----QRG-GSSGD------AAA--ANTS

*L. panamensis*  ------------AASVASTVTGTYTLPSVT-SS-----------------------------SRRSGRGG-G----QRG-GRSGG------AAAGEANMS

*L. tropica*  ------------AASVASTASGTYTLPSVK-PS-----------------------------ARRSVRGG-G----QRG-GRGGD------AAA--ANTS

*L. turanica*  ------------AASVASTASGTYTLPSVK-PS-----------------------------ARRSVRGG-G----QRG-GRGGD------AAA--ANTS

*L. major*  ------------AASVASTASGTYTLPSVK-PS-----------------------------ARRSVRGG-G----QRG-GRGGD------AAA--ANTP

*L. mexicana*  ------------AASVASTASGTYTLPSVKPSS-----------------------------ARRSVRGG-G----QRG-GHGAD------AAA--TNTS

*T. cruzi*  N-------------QPQQQHPAPYSLPSLR-GK-----------------------------GTNNRSKP------AKG------------SAT------

*T. brucei brucei*  ------------QLAQQQQPRPAYTLPSID-VN-----------------------------SGLFQIPP-G----LAD------------GAA------

*T. brucei gambiense* ------------QLAQQQQPRPAYTLPSID-VN-----------------------------SGLFQIPP-G----LAD------------GAA------

*T. evansi*  ------------QLAQQQQPRPAYTLPSID-VN-----------------------------SGLFQIPP-G----LAD------------GAA------

*T. congolense*  -------------GEATAQPRGCYTLPIVK-DA-----------------------------KKVKRRGL-A----AVD------------SNE------

1110 1120 1130 1140

....|....|....|....|....|....|....|....|....|

jaculum1 ----PPPCACARPARRLISHKPASHGRAFYTCATKQCSFFQWAD-

jaculum2 ----KPLCHCKETCIERLSNTDRTRGRRFFKCATNTCNYFQWADS

*C. fasciculata*  GVTSDTVCGCGAPAKQLVSRKEASKGKRFLTCASRQCGFFQWLD-

*L. pyrrhocoris*  GDGAGAMCGCGVPAKQLVSRKEASKGKRFLTCANRQCGFFQWLD-

*L. seymouri*  GDGANVMCGCGMPAKQLVSRKEASKGKRFLTCANRQCGFFQWLD-

*L. arabica*  SGGVDPVCGCGAPAKQLVSRKEASRGKRFLTCANRQCSFFQWLD-

*L. aethiopica*  GGGVDPVCGCGAPAKQLVSRKEASRGKRFLTCANRQCSFFQWLD-

*L. donovani*  SGGVDPVCGCGAPAKQLVSRKEASRGKRFLTCANRQCSFFQWLD-

*L. gerbilli*  SGGVDPVCGCGAPAKQLVSRKEASRGKRFLTCANRQCSFFQWLD-

*l. enriettii*  PAGVDTVCGCGVPAKQLVSRKEASRGKHFLTCANRQCSFFQWLE-

*L. infantum*  SGGVDPVCGCGAPAKQLVSRKEASRGKRFLTCAHRQCSFFQWLD-

*L. panamensis*  TGRVDTVCGCGMPVKQLVSRKEASRGKRFLTCASRKCAFFQWLD-

*L. tropica*  GGGVDPVCGCGAPAKQLVSRKEASRGKRFLTCANRQCSFFQWLD-

*L. turanica*  SGGVDPVCGCGAPAKQLVSRKEASRGKRFLTCANRQCSFFQWLD-

*L. major*  SGGVDPVCGCGTPAKQLVSRKEASRGKRFLTCANRQCSFFQWLD-

*L. mexicana*  SGGVDTVCGCGAPAKQFVSRKEASRGKRFLTCANRQCSFFQWLD-

*T. cruzi*  ----GTLCECGVPAKQLVSRKEVSKGKRFLTCADRKCSFFQWLD-

*T. brucei brucei*  ----TPLCHCGLPAVHLVSGQAASRGRRFVKCDGSKCQFFQWLD-

*T. brucei gambiense* ----TPLCHCGLPAVHLVSGQAASRGRRFVKCDGSKCQFFQWLD-

*T. evansi*  ----TPLCHCGLPAVHLVSGQAASRGRRFVKCDGSKCQFFQWLD-

*T. congolense*  ----VSLCDCGVPAVQLVSRKQGSKGRHFLKCDKRKCSFFQWLD-

**E) eukaryotic translation initiation factor 3 subunit g**

10 20 30 40 50 60 70 80 90 100

....|....|....|....|....|....|....|....|....|....|....|....|....|....|....|....|....|....|....|....|

jaculum1 MATTTTTTTTAMAPAPATTTWVDDVNDDVMYSDGGEHYGDDHDEKALYSSS-AA-AALA--DERRWGDAKVVVEHEVDMDGNRYEVTKKVLQRHEARETA

jaculum2 MRYMENEAKGAGEFGVHAGNAIDEDEWMLMTEEQQQEHQLLQQQLEQEEYD-EQ-AMMD--DEALWDSAKVVVDHEVDVDGNKFEIVKKVRQHHEDRPTT

*C. fasciculata*  -----------------MSNWADQAQE----GEEEFYD----EVVSNDSYN-AD--YED--SKALWANAKTETTHEADAEGNTYIVVRKVRQYHVDRPIV

*L. pyrrhocoris*  -----------------MSNWADQAQE-----EEEFYN----DVASHGSYN-SE--FED--PKMSWANAKTETTQEADAEGNTYVVTRKVRKYHVDRPIS

*L. seymouri*  -----------------MSNWADQAQE-----EEEFYG----DVASNNSFA-SE--YED--PKASWANAKTETSHEADADGNTYIVTRKVRQYHVDRPIV

*L. arabica*  -----------------MSNWADAAQD-----EEQYQA----AYGSDDNDS-YS-EHEE--PKPSWDTAKTEITFEVDADGNRFEVMRKVRSYHVDRPVT

*L. aethiopica*  -----------------MSNWADAAQD-----EEQYQA----AYGSDDNDS-YS-EYEE--PKPSWDTAKTEITFEVDADGNRFEVMRKVRSYHVDRPVT

*L. donovani*  -----------------MSNWADAAQD-----EEQYQA----AYGSDDNDS-YS-EYQE--PKPSWDTAKTEITFEVDADGNRFEVMRKVRSYHVDRPVT

*L. gerbilli*  -----------------MSNWADAAQD-----EEQYQA----AYGSDDNDS-YS-EHEE--PKPSWDTAKTEITFEVDADGNRFEVMRKVRSYHVDRPVT

*L. enriettii*  -----------------MSNWADAAQD-----EDQYQA----AYGSDDNGSYSS-EFEA--PKPSWDTAKTETVLEVDADGNRFEVLRKVRSYHVDRPAT

*L. infantum*  -----------------MSNWADAAQD-----EEQYQA----AYGSDDNDS-LS-EYQE--PKPSWDTAKTEITFEVDADGNRFEVMRKVRSYHVDRPVT

*L. panamensis*  -----------------MSNWADAAQD-----EEQYQA----AYGSDDNGS-SS-EYEE--PKPSWDTAKTEVTYEVDADGNRFEVMRKVRTYHVDRPVT

*L. tropica*  -----------------MSNWADAAQD-----EEQYQA----AYGSDDNDS-YS-EYEE--PKPSWDTAKTEITFEVDADGNRFEVMRKVRSYHVDRPVT

*L. turanica*  -----------------MSNWADAAQD-----EEQYQA----AYGSDDNDS-YS-EHEE--PKPSWDTAKTEVTFEVDADGNRFEVMRKVRSYHVDRPVT

*L. major*  -----------------MSNWADAAQD-----EEQYQA----AYGSDDNDS-YS-EHEE--PKPSWDTAKTEITFEVDADGNRFEVMRKVRSYHVDRPIT

*L. mexicana*  -----------------MSNWADAAQD-----EEQYQA----AYDSDDNDS-SS-EYEE--LKPSWDTAKTEITFEVDADGNRFEVMRKVRSYHVDRPVT

*T. cruzi*  -----------------MAAWADDFDPTLEGGFADEI-----SASFEGGAD-AAVAAKD--DKLAWENAKTVIETMTDSENKRYEIVKKVRTYHVDRPVT

*T. brucei brucei*  -----------------MATWADDMEPI---ALGED------FGGNQLTPQ-EAKALAD--KEAAWKNAKVVTETITDAENKQYEIVKRVLQYRVDREAT

*T. brucei gambiense* -----------------MATWADDMEPI---ALGED------FGGNQLTPQ-EAKALAD--KEAAWKNAKVVTETITDAENKQYEIVKRVLQYRVDREAT

*T. evansi*  -----------------MATWADDMEPI---ALGED------FGGNQLTPQ-EAKALAD--KEAAWKNAKVVTETITDAENKQYEIVKRVLQYRVDREAT

*T. congolense*  -----------------MSTWADDMDPM---SLEEEI-----MNSKSLPPQ-KEPTPAERKKKAAWDNAETVTETITDGENRRYEIVKKVLKYSVDRAAT

110 120 130 140 150 160 170 180 190 200

....|....|....|....|....|....|....|....|....|....|....|....|....|....|....|....|....|....|....|....|

jaculum1 PADLRARFAPFGKAKG--DQSALVSHEPPLALEMGTADKFEREGRAEVKRLLHEVSGVDVKVHDADLALLDKFAAEVAAATAAVAAGGGVGAGGGLLSSS

jaculum2 AIDIRAKYTPYGKAKE--DQSMLISHEAPMALEMGDADQFERESRNEVKRMLNEAMTMEVKVKDPHLQIVVRKEEAKKAAAAAASA-------FGMEADG

*C. fasciculata*  AADLRAKLPHFGKGLG--DQSTLVQAEAPVAMEMGSVDQFERETRQEVRRMINEAAGMEVIVKDEHLRVIRELEKK------------------------

*L. pyrrhocoris*  MADLRARLKHFGKGLG--DQTTLVSTEPPLALEMGSVDQWERETRTEVKRMIHEASSMEVVVKDEHLRVIRELEEKD-----------------------

*L. seymouri*  AADLRSRLKHFGKGLG--DQSTLVSTEPPLALEMGSVDQWERETRTEVKRMIHEASNMEIVVKDEHLRVIRELEERD-----------------------

*L. arabica*  MADIRAKLAHFGKGKG--DQSTLVSAEPPLALEMGSVDQFERESRAEVKRMIHEASGIDVAVKDEHLRVVREMEEKAK----------------------

*L. aethiopica*  MADIRAKLAHFGKGKG--DQSTLVSAEPPLALEMGSVDQFERESRAEVKRMIHEASGIDVVVKDEHLRVVREMEEKAK----------------------

*L. donovani*  MADIRAKLAHFGKGKG--DQSTLVSAEPPLALEMGSVDQFERESRAEVKRMIHEASGIDVAVKDEHLRIVREMEEKAK----------------------

*L. gerbilli*  MADIRAKLAHFGKGKG--DQSTLVSAEPPLALEMGSVDQFERESRAEVKRMIHEASGIDVAVKDEHLRVVREMEEKAK----------------------

*L. enriettii*  MADIRAKLAHFGKGKG--DQSTLVSAEPPLALEMGSVDQFERESRAEVKRMIHEASGIDVVVKDEHLRVVREMEEKAK----------------------

*L. infantum*  MADIRAKLAHFGKGKG--DQSTLVSAEPPLALEMGSVDQFERESRAEVKRMIHEASGIDVAVKDEHLRIVREMEEKAK----------------------

*L. panamensis*  MADIRAKLAYFGKGKG--DQSTLVSAEPPLALEMGAVDQFERESRAEVKRMIHEASGIDVVVKDEHLRVVREMEEKAK----------------------

*L. tropica*  MADIRAKLGHFGKGKG--DQSTLVSAEPPLALEMGSVDQFERESRAEVKRMIHEASGIDVAVKDEHLRVVREMEEKAK----------------------

*L. turanica*  MADIRAKLAHFGKGKG--DQSTLVSAEPPLALEMGSVDQFERESRAEVKRMIHEASGIDVAVKDEHLRVVREMEEKAK----------------------

*L. major*  MADIRAKLAHFGKGKG--DQSTLVSAEPPLALEMGSVDQFERESRAEVKRMIHEASGIDVAVKDEHLRVVREMEEKAK----------------------

*L. mexicana*  MADIRAKLAHFGKGKG--DQSTLVSAEPPLALEMGAVDQFERESRAEVKRMIHEASGIDVVVKDEHLRVVREMEEKAK----------------------

*T. cruzi*  VVDIRAKWKRFGKSGD--NNQELVSRDPPIVLELGEIDPFERVARDEIVRLMNEVERMKIEVTDPRLARFAKIKEEQEKAAL------------------

*T. brucei brucei*  PVDVRAKWKRFGRATNPADQKDLVSRDPPIVLELGEVDPFERMAREEVMRLMNEVERYTVEVKDVHLARYAKVKEEQERAAK------------------

*T. brucei gambiense* PVDVRAKWKRFGRATNPADQKDLVSRDPPIVLELGEVDPFERMAREEVMRLMNEVERYTVEVKDVHLARYAKVKEEQERAAK------------------

*T. evansi*  PVDVRAKWKRFGRATNPADQKDLVSRDPPIVLELGEVDPFERMAREEVMRLMNEVERYTVEVKDVHLARYAKVKEEQERAAK------------------

*T. congolense*  PVDVRARWKRFGKDTDLTNLQDLVSRDPPIVLELGEIDPFERAARDEVMRLMNDMERATVECKDPTLARYAKVKEDRDRAAM------------------

210 220 230 240 250 260 270 280 290 300

....|....|....|....|....|....|....|....|....|....|....|....|....|....|....|....|....|....|....|....|

jaculum1 SGAAAASPTTAGAPAAAATWGAARGTRA--SNDHDVDVKRRIRVTNVSDDITEDNLRNIFSVNDCVIERLYMPRDADTKRLRGFAFITFREPWMVDEALK

jaculum2 SGAEGANVTSSSTNAGAATWGANRTLRSKPTTDRSQVYKRRIRLTNVSDDITEQNLETIFNINDCIVSRVKISKDDLTGKNRGFAFIEFREEWMVDDTLK

*C. fasciculata*  ------KKLAPRTATGGSTWGSVTPK----PTESREQVKAGIRIRNLSDDITQNNLADIFAGFNWKANVVRIPRGDN-NQSRGFAFVVFEESWMADEAIK

*L. pyrrhocoris*  ------KKAAPRTAAAGSTWGSVSK-----PTDSREQMSTGIRIRNLSDDVTQENLSRIFEGFGWSAKSVRIPRGDN-NQTRGFAFVIFDESWMADAAIQ

*L. seymouri*  ------KKSAPRTAASGSTWGSVSK-----QTDTREQMSTGIRIRNLSDDVTQENLSRIFEGLGWSAKSVRIPRGDN-NQTRGFAFVIFDEPWMADAAIK

*L. arabica*  ------KDPRVVRSGAGSTWGAIASK----STDNREQSTTGIRIRNLSDDITQENLSRIFEGRGWITKNVRIPRGDN-NQTRGFAFVIFEEAWMADAAIK

*L. aethiopica*  ------KDPRVVRSGAGSTWGAIASK----STDNREQSTTGIRIRNLSDDITQENLSRIFEGRGWITKNVRIPRGDN-NQTRGFAFVIFEEAWMADAAIK

*L. donovani*  ------KDPRVVRSGAGSTWGAIASK----STDNREQSTTGIRIRNLSDDITQENLSRIFEGRGWITKNVRIPRGDN-NQTRGFAFVIFEEAWMADAAIK

*L. gerbilli*  ------KDPRVVRSGAGSTWGAIASK----STDNREQSTTGIRIRNLSDDITQENLSRIFEGRGWITKNVRIPRGDN-NQTRGFAFVIFEEAWMADAAIK

*L. enriettii*  ------KDPRVVRSGGGSTWGAIASK----STDTREQSATGIRIRNLSDDITQENLSRIFEGRGWITKSVRIPRGDN-NQTRGFAFVIFEAAWMADAAIK

*L. infantum*  ------KDPRVVRSGAGSTWGAIASK----STDNREQSTTGIRIRNLSDDITQENLSRIFEGRGWITKNVRIPRGDN-NQTRGFAFVIFEEAWMADAAIK

*L. panamensis*  ------KDPRSVRSGAGSTWGAVASK----STDTREQSTTGIRIRNLSDDITQENLSRIFEGRGWITKNVRIPRGDN-NQTRGFAFVIFEEAWMADAAIK

*L. tropica*  ------KDPRVVRSGAGSTWGAIASK----STDNREQSTTGIRIRNLSDDITQENLSRIFEGRGWITKNVRIPRGDN-NQTRGFAFVIFEEAWMADAAIK

*L. turanica*  ------KDPRVVRSGAGSTWGAIASK----STDNREQSTTGIRIRNLSDDITQENLSRIFEGRGWITKNVRIPRGDN-NQTRGFAFVIFEEAWMADAAIK

*L. major*  ------KDPRVVRSGAGSTWGAIASK----STDNREQSTTGIRIRNLSDDITQENLSRIFEGRGWITKNVRIPRGDN-NQTRGFAFVIFEEAWMADAAIK

*L. mexicana*  ------KDPRVVRSGAGSTWGAIASK----STDNREQSTTGIRIRNLSDDITQENLSRIFEGRGWITKNVRIPRGDN-NQTRGFAFVIFEEAWMADAAIK

*T. cruzi*  ------EETAPTDQPKERTWAAARGEKG--SAQRKEDTDRRLRITNISDDISREELYNIFDTNEYRIEKLFLPRDNATGNYRGFAFITFEDHEQAERCLK

*T. brucei brucei*  ------EAAAPDDQGKERTWAAARGDKT--SVQHKEDTDRRLRITNISDDISREELYNIFNTDEYRIDKLFLPTDGKTSNYRGFAFITFETPEQAERCLS

*T. brucei gambiense* ------EAAAPDDQGKERTWAAARGDKT--SVQHKEDTDRRLRITNISDDISREELYNIFNTDEYRIDKLFLPTDGKTSNYRGFAFITFETPEQAERCLS

*T. evansi*  ------EAAAPDDQGKERTWAAARGDKT--SVQHKEDTDRRLRITNISDDISREELYNIFNTDEYRIDKLFLPTDGKTSNYRGFAFITFETPEQAERCLS

*T. congolense*  ------EENASPDQAKERTWASARVEKT--STQRREDSDRRLRITNISDDISREEMFNIFNTDEYRIEKLFLPTDSVTSNYRGFAFITFETPEQAERCLQ

310 320

....|....|....|....|....|....

jaculum1 -RTR--YTFKNVVMHVSRA--IDNKK---

jaculum2 RRQR--YMFKNVIMQAARA--TEDKWKKH

*C. fasciculata*  -MGR--FHFKNVVLDVSRA--ESRN----

*L. pyrrhocoris*  -KGK--FHFKNVVLDVSRA--ETRL----

*L. seymouri*  -KGK--FRFKNVVLDVSRA--ETRL----

*L. arabica*  -EGK--FHFKNVVLDVSRA--ETRT----

*L. aethiopica*  -EGK--FHFKNVVLDVSRA--ETRT----

*L. donovani*  -EGK--FHFKNVVLDVSRA--ETRT----

*L. gerbilli*  -EGK--FHFKNVVLDVSRA--ETRT----

*L. enriettii*  -EGK--FHFKNVVLDVSRA--ETRT----

*L. infantum*  -EGK--FHFKNVVLDVSRA--ETRT----

*L. panamensis*  -EGK--FHFKNVVLDVSRA--ETRT----

*L. tropica*  -EGK--FHFKNVVLDVSRA--ETRT----

*L. turanica*  -EGK--FHFKNVVLDVSRA--ETRT----

*L. major*  -EGK--FHFKNVVLDVSRA--ETRT----

*L. mexicana*  -EGK--FHFKNVVLDVSRA--ETRT----

*T. cruzi*  -KTKGVARFKNTVMRIVRALPEAEQRRR-

*T. brucei brucei*  -RTKGVARFKNTVMHIVRALPEGAKQRN-

*T. brucei gambiense* -RTKGVARFKNTVMHIVRALPEGAKQRS-

*T. evansi*  -RTKGVARFKNTVMHIVRALPEGAKQRS-

*T. congolense*  -RTKGVARFKNTVMRIVRALPEGESRRS-

**F) eukaryotic translation initiation factor 3 subunit l**

10 20 30 40 50 60 70 80 90 100

....|....|....|....|....|....|....|....|....|....|....|....|....|....|....|....|....|....|....|....|

jaculum1 MTTNPDIETDIPNAIYGFFRDLNRAVDNRDVGALYELYESRFDALTQEYYVVGMGQFRSWPSPKLPQVASELR-NRTADLLYTFLYNKHLFTG-RAVRHA

jaculum2 MTTNPDIESDIPQEIYQFFRQLNKAVEQRDTSSLHNLYENKFETLTKDFYTIAPGQVRNWPSLRLKQVSGEFK-NRCAELIYSFLYHKHLFTD-RSVKQA

*C. fasciculata*  MATNPDIETDIPRDVYGFFRDLSRAVERRDAGAMHELYESQFDALTKNYYVVGHGQFRSWPALRLEQVSSCFRGNKMAELVYSFLFYKHLFTGNSSIKLS

*L. pyrrhocoris*  MATNPDIETDIPRDVYGFFRDLGRAVERRDAGAMHELYEVQFDSLTKNYYIVGHGQFRSWPALRLEQVSSCFRGNKMAELVYSFLFYKHLFTGNSSIKLS

*L. seymouri*  MATNPDIETDIPRDVYSFFRDLSRAVERRDAGAMYDLYESQFDTLTKNYYIVGHGQFRSWPPLRLEQVSSCFRGNKMAELVYSFLFYKHLFTGNSFIKPS

*L. arabica*  MATNPDIETDIPRDVYQFFRGLNRAVERRDAAAMHDLYESQFDALTKNYYMAGHGQFRSWPALRLEQVSSCFRGNRMAELVYSFLFYKHLFMDNRSVRAP

*L. aethiopica*  MATNPDIETDIPRDVYQFFRGLNRAVERRDAAAMHDLYESQFDALTKNYYMAGHGQFRSWPALRLEQVSSCFRGNRMAELVYSFLFYKHLFMDNRSVRAP

*L. donovani*  MATNPDIETDIPRDVYQFFRGLNRAVERRDAAAMHDLYESQFDALTKNYYMAGHGQFRSWPALRLEQVSSCFRGNRMAELVYSFLFYKHLFMDNRSVRAP

*L. gerbilli*  MATNPDIETDIPRDVYQFFRGLNRAVERRDAAAMHDLYESQFDALTKNYYMAGHGQFRSWPALRLEQVSSCFRGNRMAELVYSFLFYKHLFMDNRSVRAP

*L. enriettii*  MATNPDIETDIPRDVYQFFRGLNRAVERRDAAAIHDLYESQFDALTKNYYMAGHGQYRSWPALRLEQVSSCFRGNRMAELVYSFLFYKHLFMDNRSVRAP

*L. infantum*  MATNPDIETDIPRDVYQFFRGLNRAVERRDAAAMHDLYESQFDALTKNYYMAGHGQFRSWPALRLEQVSSCFRGNRMAELVYSFLFYKHLFMDNRSVRAP

*L. panamensis*  MATNPDIETDIPRDVYQFFRGLNRAVERRDAAAMHELYESQFDALTKNYYMAGHGQFRSWPALRLEQVSSCFRGNRMAELVYSFLFYKHLFMDNRSVRAP

*L. tropica*  MATNPDIETDIPRDVYQFFRGLNRAVERRDAAAMHDLYESQFDALTKNYYMAGHGQFRSWPALRLEQVSSCFRGNRMAELVYSFLFYKHLFMDNRSVRAP

*L. turanica*  MATNPDIETDIPRDVYQFFRGLNRAVERRDAAAMHDLYESQFDALTKNYYMAGHGQFRSWPALRLEQVSSCFRGNRMAELVYSFLFYKHLFMDNRSVRAP

*L. major*  MATNPDIETDIPRDVYQFFRGLNRAVERRDAAAMHDLYESQFDALTKNYYMAGHGQFRSWPALRLEQVSSCFRGNHMAELVYSFLFYKHLFMDNRSVRAP

*L. mexicana*  MATNPDIETDIPRDVYQFFRGLNRAVERRDAAAMHDLYESQFDALTKNYYMAGHGQFRSWPALRLEQVSSCFRGNRMAELVYSFLFYKHLFMDNRSVRAP

*T. cruzi*  MTTNPDIESDIPHEVYSFFRALNKAVDSWDTSLLHELYENQFPGLTQNFYMAGPGQFRPWPSLRIKAVADCFK-NRTAEQLYSFLRLKHLFTD-RAVSPN

*T. brucei brucei*  MASNPDSKLHIPDEMFNFFNSLNTAVDTGDISSFHNLYENIFPSHLSKYYAAEQGEFRPLPVLQRMEVAECFG-NDTAGKLYSFLCFKHLFTD-RDVTAE

*T. brucei gambiense* MASNPDSKLHIPDEMFNFFNSLNTAVDTGDISSFHNLYENIFPSHLSKYYAAEQGEFRPLPVLQRMEVAECFG-NDTAGKLYSFLCFKHLFTD-RDVTAE

*T. evansi*  MASNPDSKLHIPDEMFNFFNSLNTAVDTGDISSFHNLYENIFPSHLSKYYAAEQGEFRPLPVLQRMEVAECFG-NDTAGKLYSFLCFKHLFTD-RDVTAE

*T. congolense*  MTTNPDIESDIPREVYSFFGGLDDAVDSWDTNILHELYENQFPSLTQAFYVGGQGQFRSWPSLRIKKVSDCFK-NRTAEQLYSFLRFKHLFTD-RGVKQA

110 120 130 140 150 160 170 180 190 200

....|....|....|....|....|....|....|....|....|....|....|....|....|....|....|....|....|....|....|....|

jaculum1 DATGAWATFNELFDLLTRGMCELPSGMLWDVFDEFVFQMTVVYQRRFSGAVEWAVPQAFRLLDRVIAESNVREVLRPSEASDEPRKRAHNTRSLSGYFAT

jaculum2 DAKMSWATFTELFEKLPQGQCDLPSWMLWDIFDEFVFQMTVAYQKRFSGVTEWSVSETLKVLDRVIAESNIIAALQQPPVVEEMTKG-NYTRVLCGFFAA

*C. fasciculata*  DAEGSWKMYTELFTLL--NSFELPSWMLWDMFDEFLYQMTVVYQKAYTSEGVWSMPEITRLLNDVIDRSHVRETMEQPERLEEILKG-HSTNALSGFYGI

*L. pyrrhocoris*  DAEGSWKMYSELFPLL--NSYELPSWMLWDMFDEFLYQMTVVYQKVFTSEGVWSMPEVTRLLNDVADRSHLRETMEQPERLEEILKG-QSTGALSGFYAI

*L. seymouri*  DAEGSWKMYSELFPLL--NSYELPSWMLWDMFDEFLYQMSVVYQKVFTSEGVWSMPEVTRLLNDVASRSHLREAMEQPERLEEILKG-QSTDALSGFYAT

*L. arabica*  DAEGAWKTYSELLPML--KSYELPNWMLWDIFDEFLYQMTVVYQKIFVTEGIWAVPEVSRLLNEVIDNSRLLELVKQPDFLEDIHRG-PNTGALSGFYAI

*L. aethiopica*  DAEGAWKTYGELLPML--KSYELPNWMLWDIFDEFLYQMTVVYQKVFATEGIWAVPEVSRLLNEVVDNSRLLELVKEPDFLDDIHRG-PNTGALSGFYAI

*L. donovani*  DAEGAWKTYSELLPML--KSYELPNWMLWDIFDEFLYQMTVVYQKVFATEGIWAVPEVSRLLNEVVDNSRLLELVKEPDFLDDIHRG-PNTGALSGFYAI

*L. gerbilli*  DAEGAWKTYSELLPML--KSYELPNWMLWDIFDEFLYQMTVVYQKIFVTEGIWAVPEVSRLLNEVIDNSRLLELVKQPDFLEDIHRG-PNTGALSGFYAI

*L. enriettii*  DAEGAWRTYSELLPML--KSYELPSWMLWDIFDEFLYQMTVVYQKVFATEGIWAVLEVSRLLNEVVDNSRLLELVKEPDFLDDIHRG-PNTGALSGFYAI

*L. infantum*  DAEGAWKTYSELLPML--KSYELPNWMLWDIFDEFLYQMTVVYQKVFATEGIWAVPEVSRLLNEVVDNSRLLELVKEPDFLDDIHRG-PNTGALSGFYAI

*L. panamensis*  DAEGAWRTYSDLFPLL--KSYELPNWMLWDIFDEFLYQMTVVYQKVFASEGTWAVPEVSRLLNEVVDNSRLFELMKEPDFLDDIHRG-PNTGALSGFYAI

*L. tropica*  DAEGAWKTYSELLPML--KSYELPNWMLWDIFDEFLYQMTVVYQKVFATEGIWAVPEMSRLLNEVVDNSRLLELVKEPDFLDDIHRG-PNTGALSGFYAI

*L. turanica*  DAEGAWKTYSELLPML--KSYELPNWMLWDIFDEFLYQMTVVYQKIFVTEGIWAVPEVSRLLNEVIDNSRLLELVKQPEFLEDIHRG-PNTGALSGFYAI

*L. major*  DAEGAWKTYSELLPML--KSYELPNWMLWDIFDEFLYQMTVVYQKVFVTEGIWAVPEVSRLLNEVIDNSRLLELVKQPDFLEDIHRG-PNTGALSGFYAI

*L. mexicana*  DAEGAWKTYSELLPML--KSYELPNWMLWDIFDEFLYQMTVVYQKVFVTEGIWAVPEVSRLLNEVVDNSRLLELVKEPDFLDDIHRG-PNTGALSGFYAI

*T. cruzi*  DARSSWDTFNNLFNALPSGSCDVPNWLLWDIFDEFLFQMTVVYQKRFTGRVGWSVNEGMQLMERVVAESGIEEAIQSENLDEITKPGARHAQWMCGFFGI

*T. brucei brucei*  DAKVSWRTFCDLFVSLP-GSCDIPNWFLWDIFDEFLFQMTVVYQKRFAEGAEWSVTEAVQMMEKVISESGIEEVMESDKADDITKSGENHVRWMSGFFGI

*T. brucei gambiense* DAKVSWRTFCDLFVSLP-GSCDIPNWFLWDIFDEFLFQMTVVYQKRFAEGAEWSVTEAVQMMEKVISESGIEEVMESDKADDITKSGENHVRWMSGFFGI

*T. evansi*  DAKVSWRTFCDLFVSLP-GSCDIPNWFLWDIFDEFLFQMTVVYQKRFAEGAEWSVTEAVQMMEKVISESGIEEVMESDKADDITKSGENHVRWMSGFFGI

*T. congolense*  DAKSSWETFVILFSALPSGSCDIPNWFLWDIFDEFLFQMTVVYQKRFAGRAEWSVTETVQIMERVVADSGIMEVMETESVEEIVKPGPRHAQWMCGFFGI

210 220 230 240 250 260 270 280 290 300

....|....|....|....|....|....|....|....|....|....|....|....|....|....|....|....|....|....|....|....|

jaculum1 VAKAKLNVLLGDYYGALSKLEVLDIYGSGRGVLQSVVPAHISLFYHIGFSYLMLHRYEDASNCFRRCLSVKAKGRRFSERVQLDAAYLFVCARVLGGMVV

jaculum2 LTKTKIHVLLGDYYAALAELEPYDIFNKSRSLLQKVAPANISLLYHVGFSYLMLRRYEDASNNFRRCFTTGVKGRKFSERVQLDSAYLFVCARVLGGMVL

*C. fasciculata*  ATMSKLDVLVGDYYSALKNLEPLDVYNKGRTVLNRVQPAAVSIYYHIGFSYLMIHRFEDASNAFRRCVQTKVNGRRFSERVQQDAAYMFVCARVLGGMPI

*L. pyrrhocoris*  AAKSKLDVLLGDYYSALKDLEPLDVYNRGRAVLNRVQPAAVSINYHIGFSYLMIHRFEDASNAFRRCVQTKLNGRRFSERVQQDAAYMFICARVLGGMPI

*L. seymouri*  ATKSKLNVLLGDYYSALKDLEPLDVYNKGRTVLNRVPPAAVSIYYHIGFSYLMIHRFEDASNAFRRCAQTKLNGRRFSERVQQDATYMFVCARVLGGMPI

*L. arabica*  VTKSKLNVLLGDYYSALTDLEPLDVYNKGRAVLTRVSPCAVSVFYHIGFSYLMIHRFEDASNAFRRCVTVKLNGRRFSERVQQDAAYMYVCARVLGGMPI

*L. aethiopica*  VTKSKLNVLLGDYYSALTDLEPLDVYNKGRAVLTRVSPCAVSVFYHIGFSYLMIHRFEDASNAFRRCVTVKLNGRRFSERVQQDAAYMYVCARVLGGMPI

*L. donovani*  VTKSKLNVLLGDYYSALTDLEPLDVYNKGRAVLTRVSPCAVSVFYHIGFSYLMIHRFEDASNAFRRCVTVKLNGRRFSERVQQDAAYMYVCARVLGGMPI

*L. gerbilli*  VTKSKLNVLLGDYYSALTDLEPLDVYNKGRAVLTRVSPCAVSVFYHIGFSYLMIHRFEDASNAFRRCVTVKLNGRRFSERVQQDAAYMYVCARVLGGMPI

*L. enriettii*  VTKSKLNVLLGDYYSALTDLEPLDVYNKGRAVLTRVSPCAVSVFYHIGFSYLMIHRFEDASNAFRRCVTVKLNGRRFSERVQQDAAYMYVCARVLGGMPI

*L. infantum*  VTKSKLNVLLGDYYSALTDLEPLDVYNKGRAVLTRVSPCAVSVFYHIGFSYLMIHRFEDASNAFRRCVTVKLNGRRFSERVQQDAAYMYVCARVLGGMPI

*L. panamensis*  VTKSKLNVLLGDYYSALTDLEPLDVYNKGRAVLSRVSPCAVSVFYHIGFSYLMIHRFEDASNAFRRCVTVKLNGRRFSERVQQDAAYMYVCARVLGGMPI

*L. tropica*  VTKSKLNVLLGDYYSALTDLEPLDVYNKGRAVLTRVSPCAVSVFYHIGFSYLMIHRFEDASNAFRRCVTVKLNGRRFSERVQQDAAYMYVCARVLGGMPI

*L. turanica*  VTKSKLNVLLGDYYSALTDLESLDVYNKGRAVLTRVSPCAVSVFYHIGFSYLMIHRFEDASNAFRRCVTVKLNGRRFSERVQQDAAYMYVCARVLGGMPI

*L. major*  VTKSKLNVLLGDYYSALTDLEPLDVYNKGRAVLTRVSPCAVSVFYHIGFSYLMIHRFEDASNAFRRCVTVKLNGRRFSERVQQDAAYMYVCARVLGGMPI

*L. mexicana*  VTKSKLSVLLGDYYSALTELEPLDVYNKGRAVLTRVSPCAVSVFYHIGFSYLMIHRFEDASNAFRRCVTVKLNGRRFSERVQQDAAYMYVCARVLGGMPI

*T. cruzi*  VTIAKVNVLLGDYMGALSALKPLDVYGRGRQILLVVAPAYVSLLYHMGFSYLMLRRYADASRVFRLSLTTKVSSRKFSEKMQFDCAYMHVISCILGGMQP

*T. brucei brucei*  ITVAKINVLLGDYNSALSVLKPLDIYGRGKKILAEVAPANVSLMYYVGFSYLMLRRYADASRVFRQSLSAKVSSRKFSERVRLDCAFMHVVSCILCGTQP

*T. brucei gambiense* ITVAKINVLLGDYNSALSVLKPLDIYGRGKKILAEVAPANVSLMYYVGFSYLMLRRYADASRVFRQSLSAKVSSRKFSERVRLDCAFMHVVSCILCGTQP

*T. evansi*  ITVAKINVLLGDYNSALSVLKPLDIYGRGKKILAEVAPANVSLMYYVGFSYLMLRRYADASRVFRQSLSAKVSSRKFSERVRLDCAFMHVVSCILCGTQP

*T. congolense*  ITLAKINVLLGDYTSALSVLKPLDIYGRGRQILLQVAPANVSLLYHVGFSYLMLRRYADASRVFRLSLSTKVNSRKFSEKVQLDCAYMHVVSCILGGTQP

310 320 330 340 350 360 370 380 390 400

....|....|....|....|....|....|....|....|....|....|....|....|....|....|....|....|....|....|....|....|

jaculum1 SNISYYLDSNKLPAFEDDKETLRSGDEDRFREVFERCSPKFLAVPPASSGTASGGHAFAANHGGLRGSEGRELQARMFRRAVQQQQQIIKLRGYFGVYQN

jaculum2 SNIRSYIDTRKASTFEDDKELLRSGDEDSFREVFDRCSPKFFTIPPLSTNV--------------KGTEGKELQARMFRRAVQQQQPIIKLRGYFGVYQN

*C. fasciculata*  TNVVSYFDHRRLATFEDDRESLRTGDEERFRDVFDRCSPKFITVPPSDGSP-------------VKGTEGRELQARMFRRAVQQQQDIIKLRGYFKVYQN

*L. pyrrhocoris*  TNMSSYFDHRRQATFEDDRESLRSGDEERFRDVFDRCSPKFLTVPPSDGSP-------------VKGTEGRELQARMFRRAVQQQQDIIKLRGYFKVYQN

*L. seymouri*  TNMSSHFDHRRQATFEDDRESLRTGDEERFRDVFDRCSPKFLTVPPSDGSP-------------VKGSEGRELQARMFRRAVQQQQDIIKLRGYFKVYQN

*L. arabica*  NNLTSYLDSRKVAAFEDDRESLRTGDEERFRDVFDRCSPKFLTVPPSDGSP-------------VKGSEGRELQARMFRRAVQQQQDIIKLRGYFKVYQN

*L. aethiopica*  NNLTSYLDSRKVAAFEDDRESLRTGDEERFRDVFDRCSPKFLTVPPSDGSP-------------VKGSEGRELQARMFRRAVQQQQDIIKLRGYFKVYQN

*L. donovani*  NNLTSYLDSRKVAAFEDDRESLRTGDEERFRDVFDRCSPKFLTVPPSDGSP-------------VKGSEGRELQARMFRRAVQQQQDIIKLRGYFKVYQN

*L. gerbilli*  NNLTSYLDSRKVAAFEDDRESLRTGDEERFRDVFDRCSPKFLTVPPSDGSP-------------VKGSEGRELQARMFRRAVQQQQDIIKLRGYFKVYQN

*L. enriettii*  NNLTSYLDSRKVAAFEDDRESLRIGDEERFRDVFDRCSPKFLTVPPSDGSP-------------VKGLEGRELQARMFRRAVQQQQDIIKLRGYFKVYQN

*L. infantum*  NNLTSYLDSRKVAAFEDDRESLRTGDEERFRDVFDRCSPKFLTVPPSDGSP-------------VKGSEGRELQARMFRRAVQQQQDIIKLRGYFKVYQN

*L. panamensis*  SNLTSYLDSRKVAAFEDDRESLRTGDEERFRDVFDRCSPKFLTVPPSDGLP-------------VKGSEGRELQARMFRRAVQQQQDIIKLRGYFKVYQN

*L. tropica*  NNLTSYLDSRKVAAFEDDRESLRTGDEERFRDVFDRCSPKFLTVPPSDGSP-------------VKGSEGRELQARMFRRAVQQQQDIIKLRGYFKVYQN

*L. turanica*  NNLTSYLDSRKVAAFEDDRESLRTGDEERFRDVFDRCSPKFLTVPPSDGSP-------------VKGSEGRELQARMFRRAVQQQQDIIKLRGYFKVYQN

*L. major*  NNLTSYLDSRKVAAFEDDRESLRTGDEERFRDVFDRCSPKFLTVPPSDGSP-------------VKGSEGRELQARMFRRAVQQQQDIIKLRGYFKVYQN

*L. mexicana*  NNLTSYLDSRKVAAFEDDRESLRTGDEERFRDVFDRCSPKFLTVPPSDGSP-------------VKGSEGRELQARMFRRAVQQQQDIIKLRGYFKVYQN

*T. cruzi*  DNLSWLVEPRKLSGFEDEKELLSAGDEERFREVFDRCSPKFLAIPPITTIM-------------YKGTDGKELQARLFRRAVKQQEDIIKLRGFFGVYQT

*T. brucei brucei*  DNLSWLMDSRKLQVLEDDKELLATGDEERFRDVFDRCSPKFLAVPPVPPTV-------------CKGMEGKELQARLFLRAVKQQQDTIKLRVYLGVYQT

*T. brucei gambiense* DNLSWLMDSRKLQVLEDDKELLATGDEERFRDVFDRCSPKFLAVPPVPPTV-------------CKGMEGKELQARLFLRAVKQQQDTIKLRVYLGVYQT

*T. evansi*  DNLSWLMDSRKLQVLEDDKELLATGDEERFRDVFDRCSPKFLAVPPVPPTV-------------CKGMEGKELQARLFLRAVKQQQDTIKLRVYLGVYQT

*T. congolense*  DNLSWLMEPRKHSVFEDDKELLAAGDEERFRDVFDRCSPKFLAVPPMTTSV-------------YKGTEGKELQARLFRRAVKQQEDTIKLRGYFGVYQT

410 420 430 440 450 460 470 480 490 500

....|....|....|....|....|....|....|....|....|....|....|....|....|....|....|....|....|....|....|....|

jaculum1 TKMSLLETLLETDDGYAPLFALKMRSRQLVHDGESANLLSGQYRVSSQYDCIVSDDSVEVVSCATFGGTEAKLYNKIKGLQRDQRQTLKPGSGPRQARRD

jaculum2 TNMQLLKQLLETEDGYAPLFALKMRSKQLVHNGESANLLSGEYKVSSQYDCVVSDDNVDVVRCTTFGGAETKLFNKIKSIYRDQAEQQKQQEQRQRREQN

*C. fasciculata*  TKMNLLETLLEVEDGYAPLFALKMRSRQLVHDGEAANLLTGEYRVSSEFDCIVQDDNVEVVPSMTVGGEEQKLISRIKATQREIEMAERKFNQVRKREDG

*L. pyrrhocoris*  TKMKLLETLLEVDDGFAPLFALKMRSRQLVHDGEAANLLTGEYRVSSEFDCIVQDDNVEVVPSMTFGGTEQKFVSKIKSTQRDIEMAERKFNQARKREEG

*L. seymouri*  TKMKLLETLLEVDDGFAPLFALKMRSRQLVHDGESADLLTGEYRVSSEFDCIVQDDNVEVVPSMTFGGTEQKLVNKIRSTQRDIEMTERKFNQARKREEG

*L. arabica*  TKMNLLETLLEVDDGYAPLFALKMRSRQLVHDGVTADLLQGVFTSSSEFDCIVQDDNVEVVPSMTFGGIEQKLLNKIKNTQRDIEMAKRKFNQTRNRDEG

*L. aethiopica*  TKMNLLETLLEVDDGYAPLFALKMRSRQLVHDGVTADLLQGVFTSSSEFDCIVQDDNVEVVPSMTFGGIEQKLLNKIKNTQRDIEMAKRKFNQTRNRDEG

*L. donovani*  TKMNLLETLLEVDDGYAPLFALKMRSRQLVHDGVTADLLQGVFTSSSEFDCIVQDDNVEVVPSMTFGGIEQKLLNKIKNTQRDIEMAKRKFNQTRNRDEG

*L. gerbilli*  TKMNLLETLLEVDDGYAPLFALKMRSRQLVHDGVTADLLQGVFTSSSEFDCIVQDDNVEVVPSMTFGGIEQKLLNKIKNTQRDIEMAKRKFNQTRNRDEG

*L. enriettii*  TKMNLLETLLEVDDGYAPLFALKMRSRQLVHDGVSADLLQGVFTSSSEFDCIVQDDNVEVVPSMTFGGIEQKLLNKIRNTQRDIEMAKRKFNQARNREEG

*L. infantum*  TKMNLLETLLEVDDGYAPLFALKMRSRQLVHDGVTADLLQGVFTSSSEFDCIVQDDNVEVVPSMTFGGIEQKLLNKIKNTQRDIEMAKRKFNQTRNRDEG

*L. panamensis*  TKMNLLETLLEVDDGYAPLFALKMRSRQLVHDGVTADLLQGVFTSSSEFDCIVQDDNVEVVPSMTFGGIEQKLLNKIKNTQRDIEMAKRKFNQARNREEG

*L. tropica*  TKMNLLETLLEVDDGYAPLFALKMRSRQLVHDGVTADLLQGVFTSSSEFDCIVQDDNVEVVPSMTFGGIEQKLLNKIKNTQRDIEMAKRKFNQTRNRDEG

*L. turanica*  TKMNLLETLLEVDDGYAPLFALKMRSRQLVHDGVTADLLQGVFTSSSEFDCIVQDDNVEVVPSMTFGGIEQKLLNKIKNTQRDIEMAKRKFNQTRNRDEG

*L. major*  TKMNLLETLLEVDDGYAPLFALKMRSRQLVHDGVTADLLQGVFTSSSEFDCIVQDDNVEVVPSMTFGGIEQKLLNKIKNTQRDIEVAKRKFNQTRNRDEG

*L. mexicana*  TKMNLLETLLEVDDGYAPLFALKMRSRQLVHDGVTADLLQGVFTSSSEFDCIVQDDNVEVVPSMTFGGIEQKLLNKIKNTQRDIEMAKRKFNQTRNRDEG

*T. cruzi*  TTTELVKTVLDVDDGHVPLFAMRLRSRQLVHDGSSADLLSGSYAVRSAIDYTVKGENIDVVQKSSYRTTESKYFMRINNLRRRQRHFENQRQERRQVQPI

*T. brucei brucei*  TTTELVKTVLDVNDGLVPLFAMKLTSRQLVHDGVSADLHSGTYVVRAALDCTVEGDNICVVQKSSYRTIESKYFKRMTQRRPRHRHFDNPRQGRRPAPDA

*T. brucei gambiense* TTTELVKTVLDVNDGLVPLFAMKLTSRQLVHDGVSADLHSGTYVVRAALDCTVEGDNICVVQKSSYRTIESKYFKRMTQRRPRHRHFDNPRQGRRPAPDA

*T. evansi*  TTTELVKTVLDVNDGLVPLFAMKLTSRQLVHDGVSADLHSGTYVVRAALDCTVEGDNICVVQKSSYRTIESKYFKRMTQRRPRHRHFDNPRQGRRPAPDA

*T. congolense*  TTTELVKTILDVDDGLIPLFAMKLSSRQLIHDGVSADLHSGTYAVRAALDYTVKGGNISVVQKSSYRTTESKYHTRIINLRRRYRYLETHRPERRQAQTP

510 520 530 540 550 560 570 580 590 600

....|....|....|....|....|....|....|....|....|....|....|....|....|....|....|....|....|....|....|....|

jaculum1 YGAAAQGISNNAA-----RRQL-----------YGRGGAAARGGGVG----GGRGGARQH-GGRMPAAVVQ----PGNNLFNRNEH--------------

jaculum2 E-QRRQNMTNGAGQGQGPRRQMRHNANYHNNNNNNNGGMNAIGGGGG----NNRNQMR---GPRVNWAQQTFANNNNNNNNNPNSNPMQGQGQGQGQGEG

*C. fasciculata*  G-KK-----------KDPREQNRKPHPKQ--------------SAPN----QA-GGEAPRRRPNPKAPAQP----MMNNLFNRNDN--------------

*L. pyrrhocoris*  G-KKT----------KDQREQKRKPNLQQ------------GGGAPN----AA-GGDAPRRRPNVKVAAQP----MTSNLFNRNDN--------------

*L. seymouri*  G-KKT----------KDQREQKRRPNQQQ------------GVGAAN----AT-GTDAPRRRPNAKVAAQP----MTNNLFNRNDN--------------

*L. arabica*  G-NKR----------KDQRDQKRRPNQQQ-----------LHGGVGNNAGSANRSSNNANRHPRQKMAAQP----MTSNLFNRNDN--------------

*L. aethiopica*  G-NKR----------KDPRDQKRRPNQQQ-----------LHGGVGNNAGSANRNSNNANRHPRQKMAAQP----MASNLFNRNDN--------------

*L. donovani*  G-NKR----------RDQRDQKRRPNQQQ-----------LHGGVGNNAGSANRNNNNANRYPRQKMAAQP----MTSNLFNRNDN--------------

*L. gerbilli*  G-NKR----------KDQRDQKRRPNQQQ-----------LHGGVGNNAGSANRSSNNANRHPRQKMAAQP----MTSNLFNRNDN--------------

*L. enriettii*  G-NKR----------KDQREQKRRPNQQQ-----------YHGGIGG----ANRNSNNLNQNQRTKMAAQP----MASNLFNRNDN--------------

*L. infantum*  G-NKR----------RDQRDQKRRPNQQQ-----------LHGGVGNNAGSANRNNNNANRYPRQKMAAQP----MTSNLFNRNDN--------------

*L. panamensis*  G-NRR----------KENREQKRRPNQQL-----------LHGGAGN----ANRNSNNTN-HPRQRVTAQP----MTSNLFNRNDN--------------

*L. tropica*  G-NKR----------KDQRDQKRRPNQQQ-----------LHGGVGNNAGSANRNSNNANRHPRQKMAAQP----MTSNLFNRNDN--------------

*L. turanica*  G-NKR----------KDQRDQKRRPNQQQ-----------LHGGVGNNAGSANRSSNNANRHPRQKMAAQP----MTSNLFNRNDN--------------

*L. major*  G-NKR----------KDQRDQKRRPNQQQ-----------LHGGVGNNAGSANRSSNNVNRHPRQKMAAQP----MTSNLFNRNDN--------------

*L. mexicana*  A-NKR----------KDPRDQKRRPNQQL-----------LHGGVGNNTGSANRNSNNANRHPRQKMAAQP----MTNNLFNRNDN--------------

*T. cruzi*  S-VAK-----------------------------------------------------------------------------------------------

*T. brucei brucei*  H-NTN-----------------------------------------------------------------------------------------------

*T. brucei gambiense* H-NTN-----------------------------------------------------------------------------------------------

*T. evansi*  H-NTN-----------------------------------------------------------------------------------------------

*T. congolense*  Y-TTN-----------------------------------------------------------------------------------------------

610 620 630

....|....|....|....|....|....|.

jaculum1 -------------------------------

jaculum2 QRMQRQRPMPRRVTAPVVKVQPTNDFFSRNE

*C. fasciculata*  -------------------------------

*L. pyrrhocoris*  -------------------------------

*L. seymouri*  -------------------------------

*L. arabica*  -------------------------------

*L. aethiopica*  -------------------------------

*L. donovani*  -------------------------------

*L. gerbilli*  -------------------------------

*L. enriettii*  -------------------------------

*L. infantum*  -------------------------------

*L. panamensis*  -------------------------------

*L. tropica*  -------------------------------

*L. turanica*  -------------------------------

*L. major*  -------------------------------

*L. mexicana*  -------------------------------

*T. cruzi*  -------------------------------

*T. brucei brucei*  -------------------------------

*T. brucei gambiense* -------------------------------

*T. evansi*  -------------------------------

*T. congolense*  -------------------------------

**G) DNA J-binding protein**

10 20 30 40 50 60 70 80 90 100

....|....|....|....|....|....|....|....|....|....|....|....|....|....|....|....|....|....|....|....|

jaculum1 MLVQHPSVS----ADGVAPSSVWG----KKMRSELHD-----VSSASTRARSPEELMRAYAEAVKLHPFRHNAHSLCDIYADTVLRDGNGTVVAVLIRGG

jaculum2 MLKTETKR-----IKLEACSVNEE--ESNKLDSETITPLT--PHSALSLRRTSEVAERAYYEAIKQHPFYHNAHSVADVYSSSVIRDGAGQVVAVLIKNG

*C. fasciculata*  M---------------------EP--KSKKVKQDIFN------FPDGKDVPTTKEKAEAYVDALKAHPFYDNVHSVVDVYDSATLRDGKGRVIGVMLRKA

*L. pyrrhocoris*  ---------------------MKP--DLKKAKLDIFS------FPSGKDVRTPADVTESYTDAMKSHPFFDNAHTIAELYDSATVKDGKGRIIGVVLRKA

*L. seymouri*  ---------------------MEP--GLKRAKLDIFS------LPRGKSGHTPEEAAASYAEAVRSHPFYDNAHSIVDLYDSATLKDGKGRIIGVVLRKA

*L. arabica*  ---------------------MEP--DPKKIKLDIFN------FPTARETRTPEEVAESYAEAVKSHPFYDNVHSVIDFYDSGTIKDGRGQIIGVVLREA

*L. aethiopica*  ---------------------MEP--DPKKVKLDIFN------FPTARETRTPEEVAESYAEAVKSHPFYDNVHSVIDFYDSGTIKDGRGQIIGVVLREA

*L. donovani*  ---------------------MEP--DPKKVKLDIFD------FPTARETRTPEEVAESYAEAVKSHPFYDNVHSAIDFYDSGTIKDGRGQIIGVVLREA

*L. gerbilli*  ---------------------MEP--DPKKIKLDIFN------FPTAKETRTPEEVAESYAEAVRSHPFYDNVHSVIDFYDSGTIKDGRGQIIGVVLREA

*L. enriettii*  ---------------------MES--DAKKVRLDIFN------FPSGKDKRTPEDVAESYTEAVKSHPFHDNVHSVIDFYDSGTIRDGRGQVVGVVLRKA

*L. infantum*  ---------------------MEP--DPKKVKLDIFD------FPTARETRTPEEVAESYAEAVKSHPFYDNVHSAIDFYDSGTIKDGRGQIIGVVLREA

*L. panamensis*  ---------------------MES--STKRIKMDIFN------FPTIKETRTPEEVAESYAEAVKLHPFYDNAHCVIDFYDSGTIKDGRGEIIGVVLRKA

*L. tropica*  ---------------------MEP--DPKKVKLDIFN------FPTARETRTPEEVAESYAEAVKSHPFYDNVHSVIDFYDSGTIKDGRGQIVGVVLREA

*L. turanica*  ---------------------MEP--DPKKIKLDIFN------FPTARETRTPEEVAESYAEAVKSHPFYDNVHSVIDFYDSGTIKDGRGQIIGVVLREA

*L. major*  ---------------------MEP--DPKKIKLDIFN------FPTARETRTPEEVAESYAEAVKSHPFYDNVHSVIDFYDSGTIKDGRGQIIGVVLREA

*L. mexicana*  ---------------------MEP--DSKKVKLDIFS------FPTARETRTPEEVAESYAEAVKSHPFYDNVHSVIDFYDSGTIKDGRGKIIGVVLREA

*T. cruzi*  M-KQ-KRGKQDVKMLESAPPQLLP----KKGRLEISE-----LAPQQRTIRTAEEIETAYNEAVKKHPFYDNADHTIDFHDATVFRDARGVVGGVFLPGA

*T. brucei brucei*  MRRQVKK-----VLREKADDSMKPGWDVYQPSNDVVYAFNHYMQGSQIDAEAREKAEKAFQEAVKKHPFHNNADHTVDFHGTTVFRNAKGKVCGVLIPKA

*T. brucei gambiense* MRRQVKK-----VLREKADDSMKPGWDVYQPSNDVVYAFNHYMQGSQIDAEAREKAEKAFQEAVKKHPFHNNADHAVDFHGTTVFRNAKGKVCGVLIPKA

*T. evansi*  MRRQVKK-----VLREKADDSMKPGWDVYQPSNDVVYAFNHYMQGSQIDAEAREKAEKAFQEAVKKHPFHNNADHAVDFHGTTVFRNAKGKVCGVLIPKA

*T. congolense*  ----------------------------------------------------------------------------------------------------

110 120 130 140 150 160 170 180 190 200

....|....|....|....|....|....|....|....|....|....|....|....|....|....|....|....|....|....|....|....|

jaculum1 LPPHAAAAAATVLRGAATRTTLRMNIYGGEAPHSGIAGYFDYRGTPIEHKYRKTAFTYENADAWPGVFAMVDYVSALYKRALPQCWHAQDAAIPDVVRIH

jaculum2 LPRFAAEQAAHVLRPAATRTSLRSRIYGGEAPHSGIAGYFDYRGSPIEHKYRKTAFTYEHIENWHEVYPMIDYVSTLYKLTLPSCWEAQSNAIPDVVRIH

*C. fasciculata*  LPEHATTAASGLLSAAAVRTSLRSSMFGGESPLSGIAGYFDYRGSPVELKARKTAFTYEHEKKWPAVFPLVDYVSEIYKSVMPEHWAAQDSAIPDIVRIH

*L. pyrrhocoris*  LPEFAASTAADLLISAAVRSSLRSPMFGGESPLSGIAGYFDYRGSPVELKARKTSFTYEHEKDWPAVFPLVDYVSEIYKCVMPEHWAAQDSAIPDVVRIH

*L. seymouri*  LPEFAASAAADLLISAAVRTSLRSPMFGGEAPLSGIAGYFDYRGSPVELKSRKTSFTYEHEKEWPAVFPLVDYVSAIYKCVMPEHWAAQNSAIPDIVRIH

*L. arabica*  LPKYAASMASELLASAAVRTSLRSMMFGGESPLSGIAGYFDYRGSPVELKSRKTSFTYEHEAAWPAVFPVVDYVSELYRHVAPERWKAQNDAIPDVVRIH

*L. aethiopica*  LPKYAASMASELLASAAVRTSLRSMMFGGESPLSGIAGYFDYRGSPVELKSRKTSFTYEHEAAWPAVFPVVDYVSELYRHVAPERWKAQNDAIPDVVRIH

*L. donovani*  LPKYAASMASELLASAAVRTSLRSMMFGGESPLSGIAGYFDYRGSPVELKSRKTSFTYEHEAAWPAVFPVVDYVSELYRHVAPERWKAQNDAIPDVVRIH

*L. gerbilli*  LPKYAASMASELLASAAVRTSLRSMMFGGESPLSGIAGYFDYRGSPVELKSRKTSFTYEHEAAWPAVFPVVDYVSELYRHVAPERWKAQNDAIPDVVRIH

*L. enriettii*  IPEYAASMAAELLISAAVRTSLRSTMFGGESPLSGIAGYFDYRGSPVELKSRKTSFTYEREAEWPAVFPVVDYVSEIYRHVAPEQWKAQNDAIPDVVRIH

*L. infantum*  LPKYAASMASELLASAAVRTSLRSMMFGGESPLSGIAGYFDYRGSPVELKSRKTSFTYEHEAAWPAVFPVVDYVSELYRHVAPERWKAQNDAIPDVVRIH

*L. panamensis*  LPKYATSMASVLLISAAVRTSLRSMIFGGESPLSGIAGYFDYRGSPVELKSRKTSFTYEHEEAWSAVFPVVDYVSEIYRHVAPERWKAQNNAIPDLVRIH

*L. tropica*  LPKYAASMASELLASAAVRTSLRSMMFGGESPLSGIAGYFDYRGSPVELKSRKTSFTYEHEAAWPAVFPVVDYVSELYRHVAPERWKAQNDAIPDVVRIH

*L. turanica*  LPKYAASMASELLASAAVRTSLRSMMFGGESPLSGIAGYFDYRGSPVELKSRKTSFTYEHEAAWPAVFPVVDYVSELYRHVAPERWKAQNDAIPDVVRIH

*L. major*  LPKYAVSMASELLASAAVRTSLRSMMFGGESPLSGIAGYFDYRGSPVELKSRKTSFTYEHEAAWPAVFPVVDYVSELYRHVAPERWKAQNDAIPDVVRIH

*L. mexicana*  LPKYATSMASELLTSAAVRTSLRSMMFGGESPLSGIAGYFDYRGSPVELKSRKTSFTYEHEAEWPAVFPVIDYVSELYRHVAPKQWKAQNDAIPDLVRIH

*T. cruzi*  LPAFAATMAADVLRPAAVRTSLRSNMFGGFAPLSGIAGYFDYRGSPVELKCRKTSFTYENVHSWPNVFPMIDYVSAIYKAVFPEQWAAQDAAVPDIVRIH

*T. brucei brucei*  LPSFATSMAADVLECAVARTSLRSALFGGVSPNSGIAGYFDYRGTPVELKCRKTSFTYEHTKEWRSVFPMIDYTSAIYKAALPDHWKAQDAAVPDVVRIH

*T. brucei gambiense* LPSFATSMAADVLECAVARTSLRSALFGGVSPNSGIAGYFDYRGTPVELKCRKTSFTYEHTKEWRSVFPMIDYTSAIYKAALPDHWKAQDAAVPDVVRIH

*T. evansi*  LPSFATSMAADVLECAVARTSLRSALFGGVSPNSGIAGYFDYRGTPVELKCRKTSFTYEHTKEWRSVFPMIDYTSAIYKAALPDHWKAQDAAVPDVVRIH

*T. congolense*  ----------------------------------------------------------------------------------------------------

210 220 230 240 250 260 270 280 290 300

....|....|....|....|....|....|....|....|....|....|....|....|....|....|....|....|....|....|....|....|

jaculum1 GSPFSTLTINSRFRTASHTDAGDFDAGYGCLACLEGNFTGMCLTLDDFRVNVTLQPCDVLLFDTHHFHSNTEVELDDV-----TAPWSRLTCVFYYRAAL

jaculum2 NSPFSTLTINSRFRTASHTDAGDFDSGFGCLACLEGEFTGLCLTIDKLKINVRVRPCDVLIFNTHHWHSNTELECESVHHNMNSTSESRLTCVFYYRAAL

*C. fasciculata*  GTPFSTLTINSRFRTASHTDAGDFDGGYSCIACIDGDFKGLALGFDDFHVNVPMQPRDVLVFDSHYFHSNSELEISCP-----TEDWRRLTCVFYYRSAL

*L. pyrrhocoris*  GAPFSTLTINSRFRTATHTDAGDFDGGYSCIACIDGNFKGLALTFDDFHMSVLLQPRDVFVFDSHHFHSNTEVEESCP-----SEDWRRLTCVFYYRSPL

*L. seymouri*  GTPFSTLTINSRFRTASHTDAGDFDGGYSCIACIDGNFKGLALTFDDFRVNVLLQPRDVLVFDSHHYHSNTEVEISCQ-----SEDWKRLTCVFYYRSAL

*L. arabica*  GTPFSTLTINSRFRTASHTDVGDFDGGYSCIACLDGHFKGLALAFDDFGINVLMQPRDVMIFDSHHFHSNTEVELSFS-----GEDWKRLTCVFYYRAAL

*L. aethiopica*  GTPFSTLTINSRFRTASHTDVGDFDGGYSCIACLDGHFKGLALAFDDFGINVLMQPRDVMIFDSHHFHSNTEVELSFS-----GEDWKRLTCVFYYRAAL

*L. donovani*  GTPFSTLTINSRFRTASHTDVGDFDGGYSCIACLDGQFKGLALAFDDFGINVLMQPRDVMIFDSHHFHSNTEVELSFS-----GEDWKRLTCVFYYRAAL

*L. gerbilli*  GTPFSTLTINSRFRTASHTDVGDFDGGYSCIACLDGHFKGLALAFDDFGINVLMQPRDVMIFDSHHFHSNTEVELSFS-----GEDWKRLTCVFYYRAAL

*L. enriettii*  GTPFSTLTINSRFRTASHTDVGDFDGGYSCIACIDGQFKGLALSFDDFRINVLLQPRDVLIFDSHHFHSNTEVEASCS-----GEDWKRLTCVFYYRAAL

*L. infantum*  GTPFSTLTINSRFRTASHTDVGDFDGGYSCIACLDGQFKGLALAFDDFGINVLMQPRDVMIFDSHHFHSNTEVELSFS-----GEDWKRLTCVFYYRAAL

*L. panamensis*  GTPFSTLTINSRFRTASHTDVGDFDAGYSCIACIDGQFKGLALTFDDFRINVLMQPRDVMVFDSHHFHSNTEVEVSCS-----EEDWKRLTCVFYYRTAL

*L. tropica*  GTPFSTLTINSRFRTASHTDVGDFDGGYSCIACLDGNFKGLALAFDDFGINVLMQPRDVMIFDSHHFHSNTEVELSFS-----GEDWKRLTCVFYYRAAL

*L. turanica*  GTPFSTLTINSRFRTASHTDVGDFDGGYSCIACLDGHFKGLALAFDDFGINVLMQPRDVMIFDSHHFHSNTEVELSFS-----GEDWKRLTCVFYYRAAL

*L. major*  GTPFSTLTINSRFRTASHTDVGDFDGGYSCIACLDGHFKGLALAFDDFGINVLMQPRDVMIFDSHHFHSNTEVELSFS-----GEDWKRLTCVFYYRAAL

*L. mexicana*  GTPFSTLTINSRFRTASHTDVGDFDGGYSCIACLDGQFKGLALALDSFGINVLMQPRDVMIFDSHHFHSNTEVELSFS-----GEDWKRLTCVFYYRAAL

*T. cruzi*  GSPFSTLTVNQQFRTASHTDAGDFDMGYGLLAVLEGKFEGLSLALDDFGVCFRMQPRDILIFNTHFFHSNTELELDHP-----GDEWSRLTCVCYYRAAL

*T. brucei brucei*  GSPFSTLTVNERFRTASHTDNGDFDNGYGVLAVLKGEYSGLSLALDDYGVCFNMQPTDVLLFDTHLFHSNTELEAKEA-----NATWNRLSCVFYYRAAL

*T. brucei gambiense* GSPFSTLTVNERFRTASHTDNGDFDNGYGVLAVLKGEYSGLSLALDDYGVCFNMQPTDVLLFDTHLFHSNTELEAKEA-----NATWNRLSCVFYYRAAL

*T. evansi*  GSPFSTLTVNERFRTASHTDNGDFDNGYGVLAVLKGEYSGLSLALDDYGVCFNMQPTDVLLFDTHLFHSNTELEAKEA-----NATWNRLSCVFYYRAAL

*T. congolense*  -----------------------------------------------------MRPTDVLLFNTHFFHSNTELETIDP----NDGNWSRLSCVFYFRTML

310 320 330 340 350 360 370 380 390 400

....|....|....|....|....|....|....|....|....|....|....|....|....|....|....|....|....|....|....|....|

jaculum1 GEAAAYATYERRLLAAQRALTEAATAAAVAATVVAATRTRKGARARLTIRQRRTQPARATTARQTADTSARDGNDNGGSRGGARMSSKRAAATAVAKIQT

jaculum2 GEAAAYAEYNRRLQNALQT---PNFAPSLRF---------------------------------------------------------------------

*C. fasciculata*  GEPSSYAEYRRRLAAAQQ----DSTAQPVV----------------------------------------------------------------------

*L. pyrrhocoris*  GEPGSYAEYQRRLAAALQD---KE-ANLAV----------------------------------------------------------------------

*L. seymouri*  GEPGSYAEYQRRLEAALQD---KE-AHPVV----------------------------------------------------------------------

*L. arabica*  GEPASYAEYRRRLEKSKQD---TS-FTPVM----------------------------------------------------------------------

*L. aethiopica*  GEPASYAEYRRRLEKSKQD---TS-FTPVL----------------------------------------------------------------------

*L. donovani*  GEPASYAEYRRRLEKSKQD---TS-FTPVV----------------------------------------------------------------------

*L. gerbilli*  GEPASYAEYRRRLEKSKQD---TS-FTPVV----------------------------------------------------------------------

*L. enriettii*  GEPSSYAEYQRRLEKSRQD---AS-FTPVV----------------------------------------------------------------------

*L. infantum*  GEPASYAEYRRRLEKSKQD---TS-FTPVV----------------------------------------------------------------------

*L. panamensis*  GEPSSYAEYRRRLEKSKQD---PS-FTPVV----------------------------------------------------------------------

*L. tropica*  GEPASYAEYRRRLEKSKQD---TS-FTPVV----------------------------------------------------------------------

*L. turanica*  GEPASYAEYRRRLEKSKQD---TS-FTPVV----------------------------------------------------------------------

*L. major*  GEPASYAEYQRRLEKSKQD---NS-FTPVV----------------------------------------------------------------------

*L. mexicana*  GEPASYAEYRRRLEKSKQD---TS-FTPAV----------------------------------------------------------------------

*T. cruzi*  GEPACVAEYERRLARAKEI---GASPPPAV----------------------------------------------------------------------

*T. brucei brucei*  GEQPCVEEYRRRLKKAKEE---KS-TSLSF----------------------------------------------------------------------

*T. brucei gambiense* GEQPCVEEYRRRLKKAKEE---KS-TSLSF----------------------------------------------------------------------

*T. evansi*  GEQPCVEEYRRRLKKAKEE---KS-TSLSF----------------------------------------------------------------------

*T. congolense*  GRPSCLAEYRRRLGINTHA---AITAEHLD----------------------------------------------------------------------

410 420 430 440 450 460 470 480 490 500

....|....|....|....|....|....|....|....|....|....|....|....|....|....|....|....|....|....|....|....|

jaculum1 CATRATTTAAAATMTTTTTTMATLDVKDGVLVDAAVHAAVRRCRAIVVKPNGENANKPSVVHPLELTPFAVLATLRCCAHCAG-----------------

jaculum2 -------------------------------------------TSIEVKPNGENLNKPATLYEVSLTPFLVYSSLRCASKRVNMICNWKNNEQEVIINTS

*C. fasciculata*  -------------------------------------------SSVVEKPNGKNLYKPSTVFPIDPTPFAVVAQLHRLHHCAA-----------------

*L. pyrrhocoris*  -------------------------------------------KEVLVKPNGENLNRPSPVFPVQPSPFAVVSALHRMHHCAA-----------------

*L. seymouri*  -------------------------------------------KEVAVKPNGENLNKPSPVFPIHLSPFAVITTLHRMRHCAA-----------------

*L. arabica*  -------------------------------------------SKVKVKANGTNLNRPSPVYPICPSPFWVPMVAHCLQHCAS-----------------

*L. aethiopica*  -------------------------------------------SNVRVKENGTNLNRPSPVYPICPSPFWVPMVAHCLQHCAS-----------------

*L. donovani*  -------------------------------------------SNVRVKENGTNLNRPSPVYPIFLSPFWVPMVAHCLQHCAS-----------------

*L. gerbilli*  -------------------------------------------SNVKVKANGTNLNRPSPVYPICPSPFWVPMVAQCLQHCAS-----------------

*L. enriettii*  -------------------------------------------SKVRVKENGTNLNRRSPVYPITPSPFSVPMLAHRLQHCAS-----------------

*L. infantum*  -------------------------------------------SNVRVKENGTNLNRPSPVYPIFLSPFWVPMVAHCLQHCAS-----------------

*L. panamensis*  -------------------------------------------SNVMIKENGTNLNRPSPVHPVPPSPFWLPMLTHCLQHCAS-----------------

*L. tropica*  -------------------------------------------SNVRVKENGTNLNRPSPVYPICPSPFWVPMVAHCLQHCAS-----------------

*L. turanica*  -------------------------------------------SNVKVKANGTNLNRPSPVYPICPSPFWVPMVAHCLQHCAS-----------------

*L. major*  -------------------------------------------SNVRVKENGTNLNRPSPVYPICPSPFWVPMVAHCLQHCAS-----------------

*L. mexicana*  -------------------------------------------SNVRVKENDTNLNRPSPVYPISHSPFWVPMVAHCLQHCAS-----------------

*T. cruzi*  -------------------------------------------DAIIQKDNGNNFNKPAPTFPYLLTPFGGAASVCSLHCCTA-----------------

*T. brucei brucei*  -------------------------------------------NHIEQKDNGENTNKPAPVYPVSLTPFSCAASAWALRGCAA-----------------

*T. brucei gambiense* -------------------------------------------NHIEQKDNGENTNKPAPVYPVPLTPFSCAASAWALRGCAA-----------------

*T. evansi*  -------------------------------------------NHIEQKDNGENTNKPAPVYPVPLTPFSCAASAWALRGCAA-----------------

*T. congolense*  -------------------------------------------KLMEQTHPAEVTNKLAPVHPVPFTPFVCAAYACSFRGAAT-----------------

510 520 530 540 550 560 570 580 590 600

....|....|....|....|....|....|....|....|....|....|....|....|....|....|....|....|....|....|....|....|

jaculum1 ---TSLRVHAWL-LHRAH---------------------AAAALFGEELQTTDGIVARREAELILANTTPDMH-TTPIRGFAQGEATLRAAAAKQQYLEL

jaculum2 GRELALCMHHWL-LQGIDNNNFVEEANNNKIGTCSLAVSRAVQLFGESLEICDGIAERNDEALVMATSAAILK-TPAMGGFRECDAELQEAIAQQTLLEL

*C. fasciculata*  ---KGLCVHELLAVPSSP---------------------LAVLLFGERLSCSDGIPLRAAEQKLKANADGASRGVTSSGGFSESDAVLTTAVEKSKYLER

*L. pyrrhocoris*  ---KALRVHELLLAPSSL---------------------LATTLFGEELTCPDGFPLRSLDVKLKANADATQRTAGRFGGFSETGTVLTTAAEKKKYLER

*L. seymouri*  ---KALRVHELLIALYST---------------------LSTTLFGEDLVCTDGIPLRGIDEKLKANADSTARPVSRLGGFSEAGAVLTTAAERRKYLER

*L. arabica*  ---EAQCVHDAMTADGSR---------------------LAEVMFGEPLYTSDGIPLRGEDKKLKANSDSAPRPLSRLGGFSETNLMVSTAVEKKKYLNS

*L. aethiopica*  ---EAQCVHEAMTADGSR---------------------LAEVMFGEPLSTSDGIPLRGEENKLKANNDSAPRPLSRLGGFSETNLMVSTAVEKKKYLNS

*L. donovani*  ---EAQCVHDAMTADGSR---------------------LAEVMFGEPLSTSDGIPLRGEEEKLKANSDSASRPLSRLGGFSETNLMVSTAVEKKKYLNS

*L. gerbilli*  ---EAQCVHDAMTADGSR---------------------LAEVMFGEPLSTSDGIPLRGEDKKLKANSDSAPRPLSRLGGFSETNRMVSTAVEKKKYLNS

*L. enriettii*  ---AAQIVHNAMTTVGSR---------------------LAEVVFGEPLSISDGIPLREEDEKLKANADSAAKPPSHLGGFSETNLMVSTAVEKKKYLNS

*L. infantum*  ---EAQCVHDAMTADGSR---------------------LAEVMFGEPLSTSDGIPLRGEEEKLKANSDSASRPLSRLGGFSETNLMVSTAVEKKKYLNS

*L. panamensis*  ---AAQSVHEAMTADGSQ---------------------LAEIIFGEPLSISDGIPLRGDDEKLKANGDTGAKPLSRLGGFSETDLMVSTAAEKRKYLDS

*L. tropica*  ---EAQCVHDAMTADGSR---------------------LAEVMFGEPLSTSDGIPLRGEDNKLKANSDSAPRPLSRLGGFSETNLMVSTAVEKKKYLNS

*L. turanica*  ---EAQCVHDAMTADGSR---------------------LAEVMFGEPLSTSDGIPLRGEDKKLKANSDSAPRPLSRLGGFSETNLMVSTAVEKKKYLNS

*L. major*  ---EAQCVHDAMTADGSR---------------------LAEVMFGEPLSTSDGIPLRGEDKKLKANSDSASRPLSRLGGFSETNLMVSTAVEKKKYLNS

*L. mexicana*  ---AAQCVHDAMTADGSR---------------------LAEVMFGEPLSTLDGIPLRREDEKLKANGDSASRPLSRLGGFSETNLMVTTAVEKKKYLNS

*T. cruzi*  ---KLLRLHELL-LENPK---------------------LEVILFGESLRTDDGLPRREKEQLISVHLPVVVK-MSPSGGFSELGGALKAAEEKQYFFEE

*T. brucei brucei*  --AMLTRLHGLV-RENAS---------------------LMTELFGEPVEVADGLPRRAPEEIIPVHKHTNVQ-MHYLGGFSEKGDILNEAMNKRHYLDK

*T. brucei gambiense* --AMLTRLHGLV-RENAS---------------------LMTELFGEPVEVADGLPRRAPEEIIPVHKHTNVQ-MHYLGGFSEKGDILNEAMNKRHYLDK

*T. evansi*  --AMLTRLHGLV-RENAS---------------------LMTELFGEPVEVADGLPRRAPEEIIPVHKHTNVQ-MHYLGGFSEKGDILNEAMNKRHYLDK

*T. congolense*  --SKLLQLHRLS-CENVA---------------------LQEELFGEPLAVSDGLLPRKPEELTISLDSASLP-VTFAGGFNVSESSIKFFQKRRQCFDK

610 620 630 640 650 660 670 680 690 700

....|....|....|....|....|....|....|....|....|....|....|....|....|....|....|....|....|....|....|....|

jaculum1 GHLSRCLGDELCDIWRLARERWLSLLREDWARLRARAPGRSNFAWNNRSAMNAAFFDLCEVARQVMLVLLSKESATASEEQAFWAIFAAHLCRACSEEVG

jaculum2 NYLSQCIGDELLEVWRVAREKWLQLVQETWNHLHTRAPNRTDFVWNNKSEMNAAFFDLCEVGKQLMLSLLATESATPQEEQAFWTLYATHLHKACEEELH

*C. fasciculata*  DHLSQCISAELLAMWVEARKHWLRLVATEWARMIATAPERTDFLWKNKSPMNTAFFDLCEVAKQVMLGLLDKETATPTEERHFWSVYAAHLHRACAERLM

*L. pyrrhocoris*  AYLSEFIAAELLEMWEKARAKWLDLVAKEWKRLIAIMPERTDFLWKNTSDMNAAFFDLCEVAKQVMLGLLEKETAQRAEEQAFWSMYAVHLNAACTEELG

*L. seymouri*  EYLSEFIAVELLEMWEQARAKWLDLVAKEWKHLVAISPKRTDFLWKNTSDMNAAFFDLCEVGKQIMLGLLEKETALPAEEETFWSMYAMHLYTACAEELG

*L. arabica*  EFLSHFISAQLLDMWKQARGKWLELVGREWTHMLTINPERKDFLWKNQSDMNSAFFDLCEVGKQVMLGLLGKEVALPKEEQAFWTMYAVHLNAACAEELH

*L. aethiopica*  EFLSHFISAQLLDMWKQARGKWLELVGREWTHMLALNPERKDFLWKNQSEMNSAFFDLCEVGKQVMLGLLGKEVALPKEEQAFWTMYAVHLNAACAEELH

*L. donovani*  EFLSHFISAQLLDMWKQARGKWLELVGREWTHMLALNPERKDFLWKNQSEMNSAFFDLCEVGKQVMLGLLGKEAALPKEEQAFWTMYAVHLNAACAEELN

*L. gerbilli*  EFLSHFISVQLLDMWKQARGKWLELVGREWTHMLTLNPERKDFLWKNQSEMNSAFFDLCEVGKQVMLGLLGKEVALPKEEQAFWTMYAVHLNAACAEELH

*L. enriettii*  EFLSSCISAQLLSMWKQARAKWLELVDKEWEHMLTLNPERKDYVWKNQSEMNAAFFDLCEVGKQVMFGLLGKEAALPKEEQAFWTMYAVHLSAACVEELH

*L. infantum*  EFLSHFISAQLLDMWKQARGKWLELVGREWTHMLALNPERKDFLWRNQSEMNSAFFDLCEVGKQVMLGLLGKEAALPKEEQAFWTMYAVHLNAACAEELN

*L. panamensis*  EFLAHCISVQLLDMWKQARARWLELVGKEWMHMLTLNPERKDFLWKNRSEMNSAFFDLCEVGKQVMLGLLDKEAALPKEEQAFWTLYAVHLSAACAEELH

*L. tropica*  EFLSHFISAQLLDMWKQARGKWLELVGREWTHMLALNPERKDFLWKNQSEMNSAFFDLCEVGKQVMLGLLGKEVALPKEEQAFWTMYAVHLNAACTEELH

*L. turanica*  EFLSHFISAQLLDMWKQARGKWLELVGREWTHMLTLNPERKDFLWKNQSEMNSAFFDLCEVGKQVMLGLLGKEVALPKEEQAFWTMYAVHLNAACAEELH

*L. major*  EFLSHFISAQLLDMWKQARGKWLELVGREWTHMLALNPERKDFLWKNQSEMNSAFFDLCEVGKQVMLGLLGKEVALPKEEQAFWTMYAVHLNAACAEELH

*L. mexicana*  EFLSHGISAQLLNMWKQARAKWLELVSREWTHMIALNPERKDFLWKNQSEMNSAFFDLCEVGKQVMLGLLGKEAALPKEEQAFWTMYAVHLNAACAEELH

*T. cruzi*  KYLADELGPDLMSMWTQSRAHWLRLVKEDWERLCRRDPERTKFTWNNSSAMNAAFFDLCDVAKQMMIGLLNKETPSSAENHSFWILFAAHLNYACATENG

*T. brucei brucei*  ENLQKMFGEEFVNIWTQSRTHWLQLVKKEWEHQKETNPTRTRFSWNNTSAMNFAFFDLCDVAKQLMCGAFGDREVNKKEEQSFWGMFAAHLDNACINEIG

*T. brucei gambiense* ENLQKMFGEEFVNIWTQSRTHWLQLVKKEWEHQKETNPTRTRFSWNNTSAMNFAFFDLCDVAKQLMCGAFGDREVNKKEEQSFWGMFAAHLDNACINEIG

*T. evansi*  ENLQKMFGEEFVNIWTQSRTHWLQLVKKEWEHQKETNPTRTRFSWNNTSAMNFAFFDLCDVAKQLMCGAFGDREVNKKEEQSFWGMFAAHLDNACINEIG

*T. congolense*  KNLKKKFTKELVEMWDQCLKLWLILVKEDWNRQNGRLPGRKRFTWNNTSLMSSAFFDLCDVASQMVDDMLVDEG-DMNDEQNFWGIFAAFLDDACVKKIG

710 720 730 740 750 760 770 780 790 800

....|....|....|....|....|....|....|....|....|....|....|....|....|....|....|....|....|....|....|....|

jaculum1 MPAEAMPMHKLNVKVKDYQFGGTRYFKDMPAEEQQRRRERRARVEQARRRQC---QRPSATATRRGHDGDDDDDKPADAGEALASDAHDIHARQWLTSDV

jaculum2 MPADAMSMRKLNVKIKDYQFGGTRYFKDLPPEEQQRRLERKARIEEARRNGE---STL-------------------------HSNDNDLRAHAWLHNDT

*C. fasciculata*  MPEEAMSLRKLNVKLKDFSFGGTRYFKDMPVEEQERRVARKASIEEARRRST---AAK-----------------------------DGEQRSNWLTNDA

*L. pyrrhocoris*  MPPEAMSLRKLNVKLKDFNFGGTRYFKDMPDDEQQRRVERKRRIEEARRRNAASSAGS------------------------------GERHSTWLTNDS

*L. seymouri*  MPQDAMSLRKLNVKLKDFNFGGTRYFKDMPIKEQQRRMERKGRIEEARRRSSPTSSAS------------------------------SERRSSWLTNDS

*L. arabica*  MPHVAMSLRKLNVKLKDFNFGGTRYFKDMPPEEQKRRMERKQRIEEARRHGMS--SGA------------------------------HEKRANWLTNDS

*L. aethiopica*  MPHVAMSLRKLNVKLKDFNFGGTRYFKDMPPEEQKRRMERKQRIEEARRHGMS--SGA------------------------------HEKRANWLTNDS

*L. donovani*  MPHVAMSLRKLNVKLKDFNFGGTRYFKDMPPEEQKRRMERKQRIEEARRHGMS--SGA------------------------------HEKRANWLTNDS

*L. gerbilli*  MPHVAMSLRKLNVKLKDFNFGGTRYFKDMPPEEQKRRMERKQRIEEARRHGMS--SGA------------------------------HEKRANWLTNDS

*L. enriettii*  MPQEAMSLRKLNVKLKDFNFGGTRYFKDMPPEEQQRRMERKQRIEDARRHGML--SGA------------------------------HEKRESWLTNDS

*L. infantum*  MPHVAMSLRKLNVKLKDFNFGGTRYFKDMPPEEQKRRMERKQRIEEARRHGMS--SGA------------------------------HEKRANWLTNDS

*L. panamensis*  MPHDAMSLRKLNVKLKDFNFGGTRYFKDMPPEEQQRRMERKQRIEEARRHGM---TGA------------------------------HEKRANWLTNDS

*L. tropica*  MPHVAMSLRKLNVKLKDFNFGGTRYFKDMPPEEQKRRMERKQRIEEARRHGMS--SGA------------------------------HEKRANWLTNDS

*L. turanica*  MPHVAMSLRKLNVKLKDFNFGGTRYFKDMPPEEQKRRMERKQRIEEARRHGMS--SGA------------------------------HEKRANWLTNDS

*L. major*  MPHVAMSLRKLNVKLKDFNFGGTRYFKDMPPEEQKRRMERKQRIEEARRHGMS--SGA------------------------------HEKRANWLTNDS

*L. mexicana*  MPHAAMSLHKLNVKLKDFNFGGTRYFKDMPPEEQKRRVERKQRIEEARRHGMS--SGS------------------------------HEKRANWLTNDS

*T. cruzi*  MPRDAVGMHKLNVKLKDFHFGGTRYLKDMPPEEQERRLERKKRIEEARRRGS---SAH------------------------------ETHTDNWLLNDK

*T. brucei brucei*  MLQGSMGMHKLNVKLKDYNFGGTRYLKDMPPEEQERRRRRRLEIEQARRRAP---IC-------------------------------DSESGDWLRNEA

*T. brucei gambiense* MLQGSMGMHKLNVKLKDYNFGGTRYLKDMPPEEQERRRRRRLEIEQARRRAP---IC-------------------------------NSESGDWLRNEA

*T. evansi*  MLQGSMGMHKLNVKLKDYNFGGTRYLKDMPPEEQERRRRRRLEIEQARRRAP---IC-------------------------------NSESGDWLRNEA

*T. congolense*  MPPHAMGMQKLNVKLKDYTFGGTRYLKDLPPEDQMRRLLRRQKIEDARRYGT---KLS------------------------------SAQSSEWLHNDT

810 820 830 840 850 860 870 880 890 900

....|....|....|....|....|....|....|....|....|....|....|....|....|....|....|....|....|....|....|....|

jaculum1 FDYQSEDAVVDYASHGWLTPDEHARRCVDEVPVT-VPSASAVATAHNALYATHAGERALVEVLVVLPAPPQTNAASSTSSEDV-GDMGVT----------

jaculum2 FDYQTEECYVDYIANEWILPEENALKRTEFTIKQEIYESSEDKTTTNHSPFIKKE----DEQLE-------------EDEEKE-AKEKIAAKLHLYQFTF

*C. fasciculata*  FDYQTEDCEVDYAGHGWAVPTQHAKTVTANVH-QEAVAAT--TEA--------------VRVLIVLPRP--------PSGDRGDAAVDLP----------

*L. pyrrhocoris*  FDYQSEDREVDYAANNWPLPQRHAEDVTKCVH---KEDVPASTEL--------------VRVLVVHSHPFG------KGHGGD-GDCKEEVDE-------

*L. seymouri*  FDYQCEDCVVDYAKNEWPLPQCYAESVTMHVC---KEDSTACWEP--------------VGLLVVFPRPDG------KGCGGV-GDCKEEVKE-------

*L. arabica*  FDYQTEDCVVDYAQHKWPPPALHAKEITKNVR---AGELPTREGV--------------VRVLVVLPDP--------QSKLEC-VDCKMEVPE-------

*L. aethiopica*  FDYQTEDCVVDYAQHKWPPPALHAREITKNVR---TGELPTREGV--------------VRVLVVLPDP--------QSTVEC-VDCKLEVPE-------

*L. donovani*  FDYQTEDCVVDYAQHKWVPPALHAKEITKNVR---SGELPTREGV--------------VRVLVVLPDP--------QSKVDC-VDCKLEVSE-------

*L. gerbilli*  FDYQTEDCVVDYAQHKWPPPALHAKEITKNVR---TGELPTREGV--------------VRVLVVLPDP--------QSKLEC-VDCKLEVPE-------

*L. enriettii*  FDYQTEDCAIDYAGHNWVLPARHAEEITKNAR---TGELPTREGV--------------VRVLVVLPNS--------KSPADS-VNCKLEMPD-------

*L. infantum*  FDYQTEDCVFDYAQHKWVPPALHAKEITKNVR---SGELPTREGV--------------VRVLVVLPDP--------QSKVDC-VDCKLEVSE-------

*L. panamensis*  FDYQTEDCVVDYAKHKWVLPERHAKAVTKDVH---TAWLPTREEV--------------VRVLVVLPDL--------QIRVEG-VDCKLEKPD-------

*L. tropica*  FDYQTEDCVVDYAQHKWPPPALHAREITKNVR---TGALPTREGV--------------VRVLVVLPDS--------QSTVEC-VDCKLEVPE-------

*L. turanica*  FDYQTEDCVVDYAQHKWPPPALHAKEITKNVR---TGELPSREGV--------------VRVLVVLPDP--------QSKLEC-VDCKLEVPE-------

*L. major*  FDYQTEDCVVDYAQHKWPPPALHAKEITKNVR---TGELPTREGV--------------VRVLVVLPDP--------QSKLEC-VDCKLEVPE-------

*L. mexicana*  FDYQTEDCIVDYAQHKWVPPAVHAKEITKNVR---TGELPTREGL--------------VRVLVVLPDP--------QSKVKC-VDCKLEVPE-------

*T. cruzi*  FDYQQEDRKVEFEENGWMTPEAYVKHLGLKPCGD-VTATASPTEP--------------IHVLVVLPRPVV------AAAAKD-AKRDV-----------

*T. brucei brucei*  FDYQTEDVAVNYEREQWITPENNAKRFGFPERGV-YGAEGAATGT--------------ISVLIVLPKP--------TNHRQK----TC-----------

*T. brucei gambiense* FDYQTEDVAVNYEREQWITPENNAKRFGFPERGV-YGAEGAATGT--------------ISVLIVLPKP--------TNHRQK----TC-----------

*T. evansi*  FDYQTEDVAVNYEREQWITPENNAKRFGFPERGV-YGAEGAATGT--------------ISVLIVLPKP--------TNHRQK----TC-----------

*T. congolense*  FDYQSEDAPVEYKKNGWIEPEENSRQFRSFVLEQ-LTDVCKDTDS--------------TDVLVVLPRP--------AASKKP-VS--------------

910 920 930 940 950 960 970 980 990 1000

....|....|....|....|....|....|....|....|....|....|....|....|....|....|....|....|....|....|....|....|

jaculum1 ---------KT----HRDSDESRRLLCSRAAQRLRQRGCCNHALP-AT-----RSEDGLVVTFAYAGNEPGR-TFDFVVAQHVLAGMSGDAQARAYVQQL

jaculum2 LLYFLVRLWKD----KQNLLEARSERVSERQQKVKIIIIRKIKIV-RS-----MKMNEIDECVILLRCRYKKVNQHMNIFCHLLQIIIVKKVKLIAISHK

*C. fasciculata*  ---------KE----ATASAEWVRLMSSPAVRRVLAAKQRNLTLL-PN-----CNVEAVSLNFAYHDSLPQKATFDFVVLQHVLSAMPEDAIATEYVSRM

*L. pyrrhocoris*  ----------N----VATSAEWLRLMSSPAVHRVLSSTQRNTSLP-PE-----CSLDNVKVAFAYHDDLPEE-KFDFVVLQHVLSVMPDDAVAATYLRGV

*L. seymouri*  ----------EVKECVATSAEWMRLMSSSAVHRVLSSAQRNTSLP-PD-----RSFDNVRVRFAYHDDLPDE-KFDFVVLQHVLSVMPDDAVAATYVQGV

*L. arabica*  ----------T----VRCSREWERLMSSLAVRRVLAAAQRNLQLP-GS-----VTHGNIEIRFAFHSRLPTD-MCDFVVLQHVLSCIPDDVLASSYIRRA

*L. aethiopica*  ----------T----VRCSCEWERLMSSPAVHRVLAAAQRNLQLP-DS-----VTHGNIEIRFAFHSRLPTG-MCDFVVLQHVLSCIPDDVLASAYIRRA

*L. donovani*  ----------T----VRCSCEWERLMSSPAVHRVLAAAQRNLQLP-DS-----VTHDNIEIRFAFHSRLPTD-MCDFVVLQHVLSCIPDDVLASAYIRRS

*L. gerbilli*  ----------T----VRCSCEWERLMSSLAVHRVLAAAQRNLQLP-GS-----VTHGNIEIRFAFHSRLPTD-MCDFVVLQHVLSCIPDDVLASAYIRRA

*L. enriettii*  ----------N----VRCSSEWMRLMSSPGVRRVLDAEQRNLQLP-DS-----FTQDSIHIVFAFHSTLPTD-VYDFVVLQHVLSCIPDDARASSYIKRA

*L. infantum*  ----------T----VRCSCEWERLMSSPAVHRVLAAAQRNLQLP-DS-----VTHDNIEIRFAFHSRLPTD-MCDFVVLQHVLSCIPDDVLASAYIRRS

*L. panamensis*  ----------T----VEDSSEWVRLVSSPAVHRLLAAAQRNLQLP-DD-----VLHGNIHIRFVFHSTLPTD-MYDFVVLQHVLSRIPDDVLASSYITRA

*L. tropica*  ----------T----VRCSCEWERLMSSPAVHRVLAAAQRNLQLP-DS-----VTHGNIEIRFAFHSRLPTG-MCDFVVLQHVLSCIPDDVLASAYIRRA

*L. turanica*  ----------T----VRCSGEWERLMSSLAVHRVLAAAQRNLQLP-GS-----VTHGNIEIRFAFHSRLPTD-MCDFVVLQHVLSCIPDDVLASAYIRRA

*L. major*  ----------T----VRCSCEWERLMSSLAVRRVLAAAQRNLQLP-GS-----VTHGNIEIRFAFHSRLPTD-MCDFVVLQHVLSCIPDDVLASAYIRRA

*L. mexicana*  ----------T----LRCSSEWERLMSSLAVHRVLAAVQRNLQLP-DS-----VTQGNIQIHFAFHSTLPTA-VYDFVVLQHVLSCIPEDVLASEYIRRA

*T. cruzi*  ----------P----LATSEESIRLLMNPAAQRVLTGKARNVTLP-SP-----LSFGGVKITVLFDGDDIDCIHPDFVILQHLLAAIEEDEAAKARVKYW

*T. brucei brucei*  ----------E----LPTSREADRIMKNPAAQRLLCAKPCNIGLSTSSNKSRTVLCGNIRIDKVFDGGSVGGKMYDFVIMRHLLAATTGEREPLECLVRW

*T. brucei gambiense* ----------E----LPTSREADRIMKNPAAQRLLCAKPCNIGLSTSSNKSRTVLCGNIRIDKVFDGGSVGGKMYDFVIMRHLLAATTGEREPLECLVRW

*T. evansi*  ----------E----LPTSREADRIMKNPAAQRLLCAKPCNIGLSTSSNKSRTVLCGNIRIDKVFDGGSVGGKMYDFVIMRHLLAATTGEREPLECLVRW

*T. congolense*  ----------T----IPQSQEADRLMKNPAAQRLLLFKKDATSERVRNKTPHVESHGRVRIVTIYSDDDVGESVYDFVIMRHVLASV-GVCDAVRSLKQW

1010 1020 1030 1040 1050 1060 1070 1080 1090 1100

....|....|....|....|....|....|....|....|....|....|....|....|....|....|....|....|....|....|....|....|

jaculum1 RRRTRGCVLIAETDLQCRYHYMLKDSVREAYLAVATDCYQQLLRAKYGYANAMVRTKATLEAMAA---PHCLARYKFDGSALNTMILLIAGDGAAA----

jaculum2 EHVS----FIKLSKKNNNNNNSSSKNINAIKLKAQKTMFALTICFSMRTELKRIFAEHTRINLLE---------YNMSSAPLP-----MTNEHPR-----

*C. fasciculata*  RSICTGCLFVVETDVQCRQYFTLHYPLRVQYDAVAPAFFQLLHRRSYGTPLARTRTKAEVEALFP---FVCCARYKLQGSPMNTVVHLLALE--------

*L. pyrrhocoris*  GAICSGCIFVAETDVQFRQYYTLQYPIRAAYDAVASTFFQLLHRVAYGTKLARTRTKAEVESLYPL---LCCARYKLQGSPMNTVVHILAPE--------

*L. seymouri*  GAVCSGCIFVVETDVQCRQYYTLKCPIRAEYDAVAPAFFQQLHRTSYGTERARMRTKAEIESLFPL---PCVARYKLEGSPMNTVVHLLALEGEHTM---

*L. arabica*  AALCSGCVYVVETDVQCRQYYTLKCAARCDYDAVAPLFFQQLHRASYGTEAARVRTKGELESLIPT---VCCARYKLQGSPLNTTVHVVSPAPSR-----

*L. aethiopica*  AALCSGCVYVVETDVQCRQYYTLKCSARCDYDAVAPLFFQQLHRVSYGTKAARMRTKGELESLIPT---VCCARYKLQGSPLNTTVHVVSPAPPAE----

*L. donovani*  AALCSGCVFVVETDVQCRQYYTLKCSVRCDYDAVAPLFFQQLHRVSYGTKAARVRTKGELESLIPT---VCCARYKLQGSPLNTTVHVVAPAPPR-----

*L. gerbilli*  AALCSGCVYVVETDVQCRQYYTLKCAARCDYDAVAPLFFQQLHRASYGTKAARVRTKGELESLIPT---VCCARYKLQGSPLNTTVHVVSPAPSR-----

*L. enriettii*  LALCSGCLFVAETDVQCRQYYTLKPSIRCDYDAVAPLFFQQLHRVSYGTKMARVRTKGELEALITT---VCHARCKLQGSPLNTTVHVVSPDVPQ-----

*L. infantum*  AALCSGCVFVVETDVQCRQYYTLKCSVRCDYDAVAPLFFQQLHRVSYGTKAARVRTKGELESLIPT---VCCARYKLQGSPLNTTVHVVAPAPPR-----

*L. panamensis*  AALCSGCLFVEETDVQCRQYYTLKYSIRRNYDAVAPHFFQQLHQASYGTKMARVRTKGELEALIPM---VCCARYKLQGSPLNTTIHVVSPTAPH-----

*L. tropica*  AALCSGCVYVVETDVQCRQYYTLKCSARCDYDAVAPLFFQQLHRVSYGTKAARVRTKGELESLIPM---VCCARYKLQGSPLNTTVHVVSPAPPR-----

*L. turanica*  AALCSGCVYVVETDVQCRQYYTLKCAARCDYDAVAPLFFQQLHRASYGTEAARVRTKGELESLIPT---VCCARYKLQGSPLNTTVHVVSPALSR-----

*L. major*  AALCTGCVYVVETDVQCRQYYTLKCAARCDYDAVASLFFQQLHRVSYGTKAARVRTKGELESLIPT---VCCARYKLQGSPLNTTVHVVSPAPSR-----

*L. mexicana*  AALCSGCLFVAETDVQCRQYYTLKCAVRCDYDTVAPLFFQQLHQASYGTKAARVRTKGELESLIPT---VCCARYKLKGSPLNTTVHVVSPAPPS-----

*T. cruzi*  AHVARYCVFVVETDVRDRRHFLLREEVRVAYEDVAEDCFRSLHAAAYSTKYNRLRTTPSLIALCNRK--NIGLRFKFRGSPLNTIALIVVGERLD-----

*T. brucei brucei*  TSLARYCTFVVEVDLLDRHHYILKSEIGEEYSAVSEICFSALYSATYARDKVNLRTTPCLLSFIDKSGNMLESRFKFNGSPLNTVAFVVRRREK------

*T. brucei gambiense* TSLARYCTFVVEVDLLDRHHYILKSEIGEEYSAVSEICFSALYSATYARDKVNLRTTPCLLSFIDKSGNMLESRFKFNGSPLNTVAFVVRRREK------

*T. evansi*  TSLARYCTFVVEVDLLDRHHYILKSEIGEEYSAVSEICFSALYSATYARDKVNLRTTPCLLSFIDKSGNMLESRFKFNGSPLNTVAFVVRRREK------

*T. congolense*  TSRARYCTFVVEVDVLDRRTYVLRKAVRDEYNRVASDCLKELYSAANCRSAIDLLTTPIIISSIKESGNVFESRFKFSGSPLNTVAFVVRRPEPKLAGDE

1110

....|....|..

jaculum1 ------------

jaculum2 ------------

*C. fasciculata*  ------------

*L. pyrrhocoris*  ------------

*L. seymouri*  ------------

*L. arabica*  ------------

*L. aethiopica*  ------------

*L. donovani*  ------------

*L. gerbilli*  ------------

*L. enriettii*  ------------

*L. infantum*  ------------

*L. panamensis*  ------------

*L. tropica*  ------------

*L. turanica*  ------------

*L. major*  ------------

*L. mexicana*  ------------

*T. cruzi*  ------------

*T. brucei brucei*  ------------

*T. brucei gambiense* ------------

*T. evansi*  ------------

*T. congolense*  HEIPLEPEALHV

**H) Tat binding protein 1-interacting protein**

10 20 30 40 50 60 70 80 90 100

....|....|....|....|....|....|....|....|....|....|....|....|....|....|....|....|....|....|....|....|

jaculum1 MTT-------GK------------AKKACVTQGKVKDSNEAARRVLAWFVRVNSPATAQSLTDALGSRVSKPLVQRTLDALHAQQRLLHKDLKRVRFYYY

jaculum2 MPT----------VSYISNKVSQLSSCNSGTSTKIKNTNDAHEMISEWFEQNNKPVTVQSLTDALGSRVAKSLVQKILEQLTEVEFLQTKDLKKIRYYYY

*C. fasciculata*  MSG------------------------CKTVIKKVKDVAEATTLVADWFERENKPATPQSLTDALGSRVAKPLVQKILDQLHTDNKLCVKDLKKIRFYYL

*L. pyrrhocoris*  MSG------------------------CKTSIKKVKDVTEATALVHDWFERENRPATPQSLTDALGSRVSKTCIQKILDQLHADSKLCVKDLKKIRFYYL

*L. seymouri*  MSG------------------------CKTSIKKLRDVKEATALVDDWFERENRPATPQSLTDALGSRVAKSLIQKILDQLHAEGRLCVKDLKKIRFYYL

*L. arabica*  MKT------------------------CKTGIQKVRDVSEATVLVLDWFERHNKPATPQSLADALGSRVAKPLLQKILEQLHAEEKLHVKDMKKIRFYYL

*L. aethiopica*  MKT------------------------CKTGINKVRDVSEATVLVLDWFERHNKPATPQSLADALGSRVAKPLLQKILEQLHAEEKLHVKDMKKIRFYYL

*L. donovani*  MKT------------------------CKTGIKKVRDVSEATTLVLDWFERHNKPATPQSLADALGSRVAKPLLQKILEQLHAEEKLHVKDMKKMRFYYL

*L. gerbilli*  MKT------------------------CKTGIQKVRDVSEATVLVLDWFERHNKPATPQSLADALGSRVAKPLLQKILEQLHAEEKLHVKDMKKIRFYYL

*L. enriettii*  MGT------------------------CKTDIKKVKDVAEATALVLDWFQRHNKPVTPQSLTDALGSRVAKSLVQKILEQLHAEETLYAKDMKKIRFYYL

*L. infantum*  MKT------------------------CKTGIKKVRDVSEATTLVLDWFERHNKPATPQSLADALGSRVAKPLLQKILEQLHAEEKLHVKDMKKMRFYYL

*L. panamensis*  MGT------------------------CKIDIKRVKDVSEATALVLDWFERHNKPATPQSLTDALGSRVAKPLLQRILEQLHTEEKLHVKDMKKIRFYYL

*L. tropica*  MKT------------------------CKTGIKKVRDVSEATVLVLDWFERHNKPVTPQSLADALGSRVAKPILQKILEQLHAEEKLHVKDMKKIRFYYL

*L. turanica*  MKT------------------------CKTGIQKVRDVSEATVLVLDWFERHNKPATPQSLADALGSRVAKPLLQKILEQLHAEEKLHVKDMKKIRFYYL

*L. major*  MKT------------------------CKTGIQKVRDVSEATVLVLDWFERRNKPATPQSLADALGSRVAKSLLQKILEQLHAEEKLHVKDMKKIRFYYR

*L. mexicana*  MGT------------------------CKTGIKKVKDVSEATALVLDWFERHNKPATPQSLADALGSRVSKPLLQKILEQLHAEEKLHVKDMKKMRFYYL

*T. cruzi*  MAL--------------------------KKAVKNSDGKDAETAILQWFECEGEPATVQSLTDALGSKFGKVLVQSVLEQCVSAKKLQAKDIKKARLYFL

*T. brucei brucei*  MAS--------------------------KKGGKKANGEDLEAAILRWFECEGEPATVQSLTDALGSKFGKQLVQNTLEQCLKEQKLLAKDIKKARFYFL

*T. brucei gambiense* MAS--------------------------KKGGKKANGEDLEAAILRWFECEGEPATVQSLTDALGSKFGKQLVQNTLEQCLKEQKLLAKDIKKARFYFL

*T. evansi*  MAS--------------------------KKGGKKANGEDLEAAILRWFECEGEPATVQSLTDALGSKFGKQLVQNTLEQCLKEQKLLAKDIKKARFYFL

*T. congolense*  MKSPSSLYSPSKTPTPARKKTPVVDTRMVTKNAKKPGEEDVEAAIINWFECEGEPATAQSLTDALGSRFGKQIVQNTLERCLEHKKLQAKDIKKARFYFL

110 120 130 140 150 160 170 180 190 200

....|....|....|....|....|....|....|....|....|....|....|....|....|....|....|....|....|....|....|....|

jaculum1 NHARDD-DDDDDDAD------------------GGGEEGGVVAATAATTTPRNDDTSDSKSS----------NDDKDNARAAQA----TCVHEALALRAR

jaculum2 CSNKEYRGVVDASSDIKEELCKEPSQIAVNKFCEPEFAESICSEELEVKSDENNVLNDNKTEC----FGINGQCENCNLQNSNTNDLPSLIDKIMCIREL

*C. fasciculata*  NMQGG--LASEKSSE-------------------GEATASPLSCDDAAQTGKPLVVD-----------------NPEEAVADDA----TALHDVSLAAAS

*L. pyrrhocoris*  RIDEP---TAHRDGE-------------------GNAICDAPNVTTA--------TGDEK--------------ADASAERDDS----AALCDVSLAASS

*L. seymouri*  RAAEP---TPHNDNE-------------------GSAFSGVPAVTAAPTTHHPVQENEEA--------------ESAPAEVDSS----TALSDVSLAASS

*L. arabica*  RVLPL--SDADGATT--------------------------TKQHSEPEDDPAATTGERGDAVSAPEVCGEAETSAELPADAHA----ALLHAVAISAVQ

*L. aethiopica*  RVLPL--SDAKGATA--------------------------TEQHSEPEDDPAATTGERGDAVSAPEVCGGAETCAESPADAHA----ALLHAVAISAVQ

*L. donovani*  RVLPL--SDANGATT--------------------------TEQHSELEDDAAATTGERGDAVGAPEVCGGAETCAESPADTHA----ALLHAVAISAVQ

*L. gerbilli*  RVLPL--SDANGATT--------------------------TKQHSEPEDDPAATTGERGDAVSAPEVCGGAETCAESPADAHA----ALLHAVAISAVQ

*L. enriettii*  RVMPL--PDPRGATT--------------------------TEPNGETEEKAVATADKGGDVTNAPEVGGGSEACGESPADAHA----ALLHAVATTAVQ

*L. infantum*  RVLPL--SDANGATT--------------------------TEQHSELEDDAAATTGERGDAVGAPEVCGGAETCAESPADTHA----ALLHAVAISAVQ

*L. panamensis*  RVLPL--LHPDGATT--------------------------IQQHGEPEDEAAIAIGE-GDMASAPEVGAGTEVCEGLPADARA----ALLRAVGISAVQ

*L. tropica*  RVLPL--SDANGATT--------------------------TEQHSQPEDDPAATTGERGDAVSAPEVCGGAETCAESPADAHA----ALLHAVAISAVQ

*L. turanica*  RVLPL--SDANGATT--------------------------TKQHSEPEGDPAATTGERGDAVSAPEVCGGAETSAESPADAHA----ALLHAVAISAVQ

*L. major*  RVLPL--SDADGATT--------------------------TKQHSEPEDDPAATTGERGNAVNVPEVCGGAETCAESPADAHA----ALLHAVAISAVQ

*L. mexicana*  RVPPL--SDANGATT--------------------------TKQHSEPEDKPAATTGERGDAASAPEVGGEAETCAESPADAHA----ALLCAVAISAVQ

*T. cruzi*  NLAEA--AEKHGG--------------------------------------------------------------VCPVDPARI----ELMQQIQQQTKL

*T. brucei brucei*  ASPAP--GSNDEGSD---------------------------------------------------------------VGSHKI----DIVKQLRQLRNG

*T. brucei gambiense* ASPAP--GSNDEGSD---------------------------------------------------------------VGSHKI----DLVKQLRQLRNG

*T. evansi*  ASPAP--GSNDEGSD---------------------------------------------------------------VGSHKI----DLVKQLRQLRNG

*T. congolense*  VSPTC--GSGEE-------------------------------------------------------------------DSAKA----ALVQQLQRQRRE

210 220 230 240 250 260 270 280 290 300

....|....|....|....|....|....|....|....|....|....|....|....|....|....|....|....|....|....|....|....|

jaculum1 VRDKQAALAVLQSRATRAERAAQLRRVADDVAALRARVAELQAHANATRTATA--TTTTTMTTTTGTRTCDAVVAAARAYVDARRRWREYKAYTMQLVHT

jaculum2 IATSQTLKMKLSKLQTSTERKVKVEEVQRNVETLLHQVEILKQYAERSRTNEI--NQGLEI-------KKRQLEELIDVSSKIRLLWHDRKYLANTLLEV

*C. fasciculata*  LAENHRRLARWRQWPSRADRVAQRAALEEEVRQLQADINTLRQRRGAEEEERSSPHSSTES-------RVARVRRVVRRYRRARQLWTERKRWAMRLLEV

*L. pyrrhocoris*  LAEKYCSLARWRGWPSQEERAAQQDALTREVRQLEADVEVLQGGRKADEKGV----ADAET-------RASRVRRAVCRYRRARQFWTERKGWAMRLLEA

*L. seymouri*  LAEKYCCLARWRGWPSQGDRAAHQDTLAREVRQLEVGAGALQRGYTADEEKV----SNAET-------CALRARRAVCRYRRARHLWTERKGWTVRLLEA

*L. arabica*  LSERSRRLTRWRGWPSSTERAEKYADLAREVCELRNSLECLQSRHAGESAEA---HGGGSW--------TSRVRRAVCRYRRARRHWVQRKDWAMCLIEA

*L. aethiopica*  LSERSRRLTRWRGWPSPTERADKYADLAREVCELRNSLERLQSRHAGESAEA---HGGGSW--------TSRVRRAVCRYRRARRHWVQRKDWAMCLLEA

*L. donovani*  LSERSRRLTRWRGWPSSTERAAKYADLAREVCELRNSLERLQSRHAGESAEA---HSGGSW--------TSRVRRAVCRYRRARRHWVQRKDWAMCLLEA

*L. gerbilli*  LSERSRRLTRWRGWPSSTERADKYADLAREVCELRNSLERLQSRHAGESAEA---HGGGSW--------TSRVRRAVCRYRRARRHWVQRKDWAMCLLEA

*L. enriettii*  LSERSRRLARWRGCPSSAERAAKFADLARDVGELRESLERHQSRYAGEPTEA---DGASSW--------AWRARRAVCRYRRARRHWLQRKEWAMCLLEA

*L. infantum*  LSERSRRLTRWRGWPSSTERAAKYADLAREVCELRNSLERLQSRHAGESAEA---HSGGSW--------TSRVRRAVCRYRRARRHWVQRKDWAMCLLEA

*L. panamensis*  LSERSRRLARWCEWPSSTERAAKCTDLARVVCELRDGLERLQSRHAEESAEA---HSNGSW--------AWCAHRAVCRYRRARRHWVQRKDWAMRLLEA

*L. tropica*  LSERSRRLTRWRGWPSPTERADKYADLAREVCELRNSLERLQSRHAGESAEA---HGGGSW--------TSRVRRAVCRYRRARRHWVQRKDWAMCLLEA

*L. turanica*  LSERSRRLTRWRGWPSSTERADKYADLAREVCELRNSLERLQSRHAGESAEA---HGGGSW--------TSRVRRAVCRYRRARRHWVQRKDWAMCLLEA

*L. major*  LSERSLRLTRWRGWPSSTERADKYADLAREVCELRNSLERLQSRHAGESAEA---HGGGSW--------TSRVRRAVCRYRRARRHWVQRKDWAMCLLEA

*L. mexicana*  LSERSRRLTRWRGWPSSMERAAKYADLAREVSELRNSLERLQFRHAGESAEA---HGGSSW--------ASRVRRAVCRYRRARRHWVQRKDWAMCLLEA

*T. cruzi*  ISDLSKELNTLLQKPSSLQRASNIALLSTECAALKQRVENVKREINQGHDVK----------------KVEDFSLLIRRYNRARELWRERKHMAEHVIDA

*T. brucei brucei*  VSMLSSELSVLLQAQTSSKRATTIASLVSECAALRSRLQALHVIAGKESSCV-----------------SDDVSLLIHRYKRARELWRDRKRITVCVIDA

*T. brucei gambiense* VSMLSSELSVLLQAQTSSKRATTIASLVSECAALRSRLQALHVIASKESSCV-----------------SDDVSLLIHRYKRARELWRDRKRITVCVIDA

*T. evansi*  VSMLSSELSVLLQAQTSSKRATTIASLVSECAALRSRLQALHVIAGKESSCV-----------------SDDVSLLIHRYKRARELWRDRKRITVCVIDA

*T. congolense*  VSELASELDALQQLPAATARATTITSLMAECKMLIARQESLRQAASNKRERD-----------------CESVSLLIHRYHRARELWKDRKHMAVRIIDA

310 320 330 340 350 360

....|....|....|....|....|....|....|....|....|....|....|....|

jaculum1 AATAAAADDNDDGNNNNTSVPDVMARMRVCTDEEAGVLYSDTTVVLPRALRV--------

jaculum2 VTTELTG------------NADIAEKLNLHPGD---------------------------

*C. fasciculata*  TGGDMHS------------PQQIAVLLGCTTDADAGVSFESAALAIPPQLLRDVGLT---

*L. pyrrhocoris*  TGGDAHS------------PQHMAALLGCTTDADVGVSFESTAVALPRQMLREIGLN---

*L. seymouri*  TGGDMHS------------HQHMAALLGCTTDADAGVSFESTAVTLTPQILRDLKLT---

*L. arabica*  TAGDAHT------------PVQAAALLGCTTDAEAGVSFGDTAVALPAPLLRELSLSQR-

*L. aethiopica*  TAGDAHT------------PVQAAALLGCTTDAEAGVSFEDTAVALPAPLLRELSLSQR-

*L. donovani*  TAGDAHT------------PVQAAALLGCTTDAEAGVSFEDTAVALPAPLLRELSLSQR-

*L. gerbilli*  TAGDAHT------------PVQAAALLGCATDAEAGVSFEDTAVALPAPLLRELSLSQR-

*L. enriettii*  TAGDALT------------PVQAASLLGCTTDVEAGVSFEETAVALPAPLLRELRLSPR-

*L. infantum*  TAGDAHT------------PVQAAALLGCTTDAEAGVSFEDTAVALPAPLLRELSLSQR-

*L. panamensis*  TAGDAHT------------PVEAAALLGCTTDEDAGVSFEDTAVVLPVSLLREPGLSQRW

*L. tropica*  TAGDAHT------------PVQAAALLGCTTDAEAGVSFEDTAVALPAPLLRELSLSQR-

*L. turanica*  TAGDVHT------------PVQAAALLGCTTDAEAGVSFEDTAVALPAPLLRELSLSQR-

*L. major*  TAGDAHT------------PLQAAALLGCTTDAEAGVSFEDTAVALPAPLMRELNLSQR-

*L. mexicana*  TAGDAHT------------PVQAAALLGCTTDAEAGVSFEDTAVALPAPLLRELSLSQRG

*T. cruzi*  ILGDSCD------------SKDLAEHFGLISDQEANVSLNETAITFPRVEGLA---NV--

*T. brucei brucei*  VLGDSCG------------PQELEDIFGLSTDEQMNLCLSGTALTLPATVSS--------

*T. brucei gambiense* VLGDSCG------------PQELEDIFGLSTDEQMNLCLSGTALTLPATVSS--------

*T. evansi*  VLGDSCG------------PQELEDIFGLSTDEQMNLCLSGTALTLPATVSS--------

*T. congolense*  VLGDNCG------------TKDLTDVFGLSTDEQMNVSLAGTALRLPEVERT--------

**I) Nucleoporin NUP96**

10 20 30 40 50 60 70 80 90 100

....|....|....|....|....|....|....|....|....|....|....|....|....|....|....|....|....|....|....|....|

jaculum1 MM--QT--------------------------------TNDPAEEVRSALAQLTARARQLAAGG-DTAATNATMATTTTAAAPPAVVAPLLHVKRNSHSL

jaculum2 ML--SRFSENATGRVASNPFGGSDINKVNYNKQI-RHGGLDPSDEMRNTLSQLTARARQLVEHETNTSHTTRSDWRHCNTASS--------NVSGSGLSH

*C. fasciculata*  -----------------------------------MPTTGDPTDEVRASLATLTARAQRLVSEKDSE------------------------PLLRNGYGL

*L. pyrrhocoris*  MF--STLSASGPRRLDGSSAMR---SNDDASNLRVLPTTGDPTDEVRASLASLTARAQRLVSEKDAE------------------------PLLRNGYGL

*L. seymouri*  MF--SSSSASGPRRLDDSSPMR---SNDDTSNLRVMPTMGDPTDEVRASLASLTARAQRLVSEKDAE------------------------PLLRNGYGL

*L. arabica*  MF--SSTSNIAPRRLGSAGP-----GGDDASALRVMPSAGDPTDEVRASLASLTARAQRLVSERDAE------------------------QLLRNGFGL

*L. aethiopica*  MF--SSTSNIAPRQLGSAGP-----GGDDASALRVMPSAGDPTDEVRASLASLTARAQRLVSERDAE------------------------QLLRNGFGL

*L. donovani*  MF--SSTSNIAPRRLGSAGP-----GGDDASALRVMPSAGDPADEVRVSLASLTARAQRLVSERDAE------------------------QLLRNGFGL

*L. gerbilli*  ML--SSTSNIAPRRLGSAGP-----GGDDASALRVMPSAGDPTDEVRASLASLTARAQRLVSERDAE------------------------QLLRNGFGL

*L. enriettii*  MF--SSPSNVTPRRL----------GGDDASTLRVMPSTGDPTDEVRASLASLTARAQRLVTERDAE------------------------PLLRNGFGL

*L. infantum*  MF--SSTSNIAPRRLGSAGP-----GGDDASALRVMPSAGDPADEVRVSLASLTARAQRLVSERDAE------------------------QLLRNGFGL

*L. panamensis*  MF--SSTSTIAPRRLGSAGP-----GGDDASALRVMPSTGDPTDEVRASLASLTARAQRLVAERNAE------------------------QLLRNGFGL

*L. tropica*  MF--SSTSNIAPRQLGSAGP-----GGDDASALRVMPSAGDPTDEVRASLASLTARAQRLVSERDAE------------------------QLLRNGFGL

*L. turanica*  ML--SSTSNIAPRRLGSAGP-----GGDDASALRVMPSTGDPTDEVRASLASLTARAQRLVSERDAE------------------------QLLRNGFGL

*L. major*  MF--SSTSDLAPRRLGSAGP-----GGDDASALRVMPSAGDPTDEVRVSLASLTARAQRLVSERGAE------------------------QLLRNGFGL

*L. mexicana*  MF--SSTSNIAPRRLGSAGP-----GGDDASALRVMPSTGDPTDEVRASLASLTARAQRLVSERDAE------------------------QLLRNGFGL

*T. cruzi*  M---SMLHDVSGGALTSKN------HLKTAVGV-----TTDPGGDVRGNIARITARARQLLADDTDT------------------------RVVRNGLSA

*T. brucei brucei*  MMSIPNLTDAPGGILIAHS------HNQSTSAVP-SGGSVDPNNDVRGNITRLTARARQLLVSDIDT------------------------RVVRNGLST

*T. brucei gambiense* MMSIPNLTDAPGGILIAHS------HNQSTSAVP-SGGSVDPNNDVRGNITRLTARARQLLVSDIDT------------------------RVVRNGLST

*T. evansi*  MMSIPNLTDAPGGILIAHS------HNQSTSAVP-SGGSVDPNNDVRGNITRLTARARQLLVSDIDT------------------------RVVRNGLST

*T. congolense*  MMSFSNFTGAPGGTLIAHN------HTQSTSAIP-QAGSSDSNNDIRGSITRLTARARQLLVDDKDM------------------------RVVRNGVST

110 120 130 140 150 160 170 180 190 200

....|....|....|....|....|....|....|....|....|....|....|....|....|....|....|....|....|....|....|....|

jaculum1 YAASRRLHDTVGLPPAAAAAAPAATMPATTR-----ASQQASQTSYAHAATLPPAAADATAFASGPSVELFMARQLGFDAQEQQRLLQQLQRRQTLVAAD

jaculum2 ------------IPPI------AQNAHALRA-----MASKTSSTL--TDLTTPSTMSCIPNVLPSSSVEVFMARQLGVDVAEQQHLLHTLEQHQPREATI

*C. fasciculata*  YEASRKA---------------AEEARQQATKCQSTSEGYAPATH--------------------ASVELFMTQHLGFDAQAQQRLLQQLQHRHAQLGAD

*L. pyrrhocoris*  YEASRKA---------------AEEARRQASKCQSASEGYAPATH--------------------ASVELFMTQHLGFDAQAQQRLLQQLQHRHAQLGAD

*L. seymouri*  YEASRKA---------------AEEARHQANKCQSTGEGYAPATH--------------------ASVELFMTQHLGFDAQAQQRLLQQLQHRHAQLGAD

*L. arabica*  FEASRKA---------------AEEARHQANKCQSTSEGYAPATH--------------------ASVELFMTQHLGFDAQAQQRLLRQLQHRHAQLGVD

*L. aethiopica*  FEASRKA---------------AEEARHQANKCQSTSEGYAPATH--------------------ASVELFMTQHLGFDAQAQQRLLRQLQHRHAQLGVD

*L. donovani*  FEASRKA---------------AEEARHQANKCQSTSEGYAPATH--------------------ASVELFMTQHLGFDAQAQQRLLRQLQRRHAQLGVD

*L. gerbilli*  FEASRKA---------------AEEARHQAHKCQSTSEGYAPATH--------------------ASVELFMTQHLGFDAQAQQRLLRQLQHRHAQLGVD

*L. enriettii*  FEASRKA---------------AEEARHQASKCQFTSEGYAPATH--------------------ASVELFMTQHLGFDAQAQQRLLQQLQHRNAQLGVD

*L. infantum*  FEASRKA---------------AEEARHQANKCQSTSEGYAPATH--------------------ASVELFMTQHLGFDAQAQQRLLRQLQRRHAQLGVD

*L. panamensis*  FEASRKA---------------AEKARHQASKCQSTSEGYAPATH--------------------ASVELFMTQHLGFDAQAQQRLLLQLQHRHAQLGVD

*L. tropica*  FEASRKA---------------AEEARHQANKCQSTSEGYAPATH--------------------ASVELFMTQHLGFDAQAQQRLLRQLQHRHAQLGVD

*L. turanica*  FEASRKA---------------AEEARHQANKCQSTSEGYAPATH--------------------ASVELFMTQHLGFDAQAQQRLLRQLQHRHAQLGVD

*L. major*  FEASRKA---------------AEEARHQANKCQSTSEGYAPATH--------------------ASVELFMTQHLGFDAQAQQRLLRQLQHRHAQLGVD

*L. mexicana*  FEASRKA---------------AEEARHQANKCQSTSEGYAPATH--------------------ASVELFMTQHLGFDAQAQQRLLQQLQRRHAQLGAD

*T. cruzi*  YETSRKV---------------AADISTRAR-----TPGQTGPTQ--------------------ASVELFMA-SLGFNLNEQQRLLQQLQANQLPLTVT

*T. brucei brucei*  YEMSRKT---------------ATDARLWAQ-----VAGQATSSQ--------------------ASVELFMA-SLGFNLQEQLRLLHQLHTNQLPIVGR

*T. brucei gambiense* YEMSRKT---------------ATDARLWAQ-----VAGQATSSQ--------------------ASVELFMA-SLGFNLQEQLRLLHQLHTNQLPIVGR

*T. evansi*  YEMSRKT---------------ATDARLWAQ-----VAGQATSSQ--------------------ASVELFMA-SLGFNLQEQLRLLHQLHTNQLPIVGR

*T. congolense*  YEMSRRV---------------AADWHARAQ-----VTGYPSTSQ--------------------ASVELFMA-SHGFNLQEQLRLLHQLQSSQLPLVGV

210 220 230 240 250 260 270 280 290 300

....|....|....|....|....|....|....|....|....|....|....|....|....|....|....|....|....|....|....|....|

jaculum1 VAQSTTTATNSSSGGAAAAAVQRAVDMTSAAATGATAGGACAAGEDDVAVMVARAKAAMLQRGRAMVHAQLEQQLRARGEALCRLAWEPYVASLQGDLVT

jaculum2 IN-------NATSPGVYKNA---NCTIATEEGVGQE-----------LNEYIAHITHKYEEEQRCALHERLHDAMAHRQQTILKASWEPYFLNAWQHDSN

*C. fasciculata*  L----------GPTDPMTAA-------EASGFTGVT----------DVDAVVAEIKNDILLDVQHAVHRRQTTLVRQQVDTLFKAAWHPYYVRVADDFET

*L. pyrrhocoris*  L----------GPVDPMAAA-------ESSGFMGIT----------DVDAVVAEIKNDILLDVQHAVHRRQTTLVRDQVDALFKAAWHSYYVQVADDYEA

*L. seymouri*  L----------GPIDPMATA-------EGSGFMGIT----------AVDAVVAEIKNDILLDVQHAVHRRQTTLVRDQVDALFKAAWHPYYVRMADDYEA

*L. arabica*  L----------GPIDTLPGA-------EASGFMGIA----------DVDAVVAELKNDILLDVQHAVHRRQTSIVRDQVDTLFKAAWHPFYVRVADDYEA

*L. aethiopica*  L----------GPIDTLPGA-------EASGFTGIA----------DVDAVVAELKNDILLDVQHAVHRRQTSIVRDQVDTLFKAAWHPFYVRVADDYEA

*L. donovani*  L----------GPIDTLPGA-------EASGFTGIA----------DVDAVVAELKNDILLDVQHAVHRRQTSIVRDQVDTLFKAAWHPFYVRVADDYET

*L. gerbilli*  L----------GPIDTLPGA-------EASGFMGIA----------DVDAVVAELKNDILLDVQHAVHRRQTSIVRDQVDTLFKAAWHPFYVRVADDYEA

*L. enriettii*  L----------GPIDALPGA-------EASGLMGTA----------DVDAVVAELKNDILLDVQHAVHRRQTSIVRDQVDTLFKAAWHPFYVRVADDYEA

*L. infantum*  L----------GPIDTLPGA-------EASGFTGIA----------DVDAVVAELKNDILLDVQHAVHRRQTSIVRDQVDTLFKAAWHPFYVRVADDYET

*L. panamensis*  L----------GPIDALPDT-------EASGFMGIA----------DVDAVVAELKNDILLDVQHAVHRRQTSIVRDQVDTLFKAAWHPFYVRIADDYEA

*L. tropica*  L----------GPIDTLPGA-------EASGFTGIA----------DVDAVVAELKNDILLDVQHAVHRRQTSIVRDQVDTLFKAAWHPFYVRVADDYEA

*L. turanica*  L----------GPIDTLPGA-------EASGFMGIA----------DVDAVVAELKNDILLDVQHAVHRRQTSIVRDQVDTLFKAAWHPFYVRVADDYEA

*L. major*  L----------GPIDTLPGA-------EASGFTGIA----------DVDAVVAELKNDILLDVQHAVHQRQTSIVRDQVDTLFKAAWHPFYVRVADDYEA

*L. mexicana*  L----------GPIDTVPSA-------EASGFTGIA----------DVDAVVAELKNDILLDVQHAVHRRQTSIVRDQVDTLFKAAWHPFYVRVADDYEA

*T. cruzi*  GDVGT----AAKTPGAFFQE-------QIDDELGST-----------LESFVANRRNETMRRSMEEMHHRVENLVQERSATIIRSSWEKCCNEVADAFES

*T. brucei brucei*  NQL--------DSLDAPWDT-------QASDELGTA-----------LEVFVANRRHEIMQRSVDEMHQRVGEMMQERSEAIIRSSWEKYSVEIADVFET

*T. brucei gambiense* NQL--------DSLDAPWDT-------QASDELGTA-----------LEVFVANRRHEIMQRSVDEMHQRVGEMMQERSEAIIRSSWEKYSVEIADVFET

*T. evansi*  NQL--------DSLDAPWDT-------QASDELGTA-----------LEVFVANRRHEIMQRSVDEMHQRVGEMMQERSEAIIRSSWEKYSVEIADVFET

*T. congolense*  THT--------DSHDTPWNA-------EASEELGSA-----------LEAFVARRRQEIIQKSVDETHQRVNELMQERSEAIIRASWEKYGVEIADALET

310 320 330 340 350 360 370 380 390 400

....|....|....|....|....|....|....|....|....|....|....|....|....|....|....|....|....|....|....|....|

jaculum1 AHVRS-------CCAD--GVAGAAASATATQASLSHVQQQQLV----HSMLDG--SQGVPLSSA---AGVSSFVYDGSVAAAALRARAVAFARIVDDCAP

jaculum2 LLAQH-------EKND--MMAMMAKNNNNNNSLLLSSSSPHMAPYNVHGATTSNANPIDNMNESSLVTNVNELMMGHSSWSASVRHKAITFARYVNEYAP

*C. fasciculata*  LSMGAAELAVRTAGGG--DRVNAG--------------------------------------TA---RSLLDVLRSDSALAAELNTRISAFAATVEEAAP

*L. pyrrhocoris*  LSVGAAELAMNKSGSSGVGRISAGAT----------------------TQRPA---ASFGASTA---HSLLDLLNGDSVLAAELNKRIAAFAAIVEEYAP

*L. seymouri*  LSMGTAELAVNKGGGSGVGRTSAGAA----------------------SHQSA---TSFGASTS---RSLLDLLNSGSALAAELNRRIAAFAAVVEEYAP

*L. arabica*  LSMGANELAAGKAGAS--GRLSSTGT----------------------AAPSTNAFGGVGSGAS---SSLVDILNSGSSLATELNRRVAAFAAIVEEYAP

*L. aethiopica*  LSMGANELAAGKAGAS--GRLSSTGT----------------------AAPSANAFGGVGSGTS---SSLVDILNSGSSLATELNRRVAAFAAIVEEYAP

*L. donovani*  LSMGANELAAGKAGAS--GRLRSMGT----------------------AAPSANALGGVGSDAS---SSLVDILNSGSSLAMELSRRVAAFAAIVEEYAP

*L. gerbilli*  LSMGANELAAGKAGAS--GRLSSTST----------------------AAPSANAFGGVGSGAS---SSLVDILNSGSSLATELNRRVAAFAAIVEEYAP

*L. enriettii*  LAMGANELAAEKGGVG--GRLSSTAA----------------------AATSARAFAGVGNGVG---NSLVDILNSGSLLATELNRRVAAFAAIVEECAP

*L. infantum*  LSMGANELAAGKAGAS--GRLRSMGT----------------------AAPSANALGGVGSDAS---SSLVDILNSGSSLAMELSRRVAAFAAIVEEYAP

*L. panamensis*  LAMGASELACGKAGPS--GRLSATDT----------------------AASSAHTFAGAGSGAS---KSLVDILNSGSALATELNRRIAAFAAIVEEYAP

*L. tropica*  LSMGANELAAGKAGAS--GRLSSTGT----------------------AAPSANAFGGVGSGAS---SSLVDILNSGSSLATELNRRVAAFAAIVEEYAP

*L. turanica*  LSMGANELAAGKAGAS--GRLSSTGT----------------------AAPSANAFGGVGSGAS---SSLVDILNSGSSLATELNRRVAAFAAIVEEYAP

*L. major*  LSMGANELAAGKAGAS--GRLSSTGT----------------------AAPSANAFGGVGSGAS---SSLVDILNSGSSLATELNRRVAAFAAIVEEYAP

*L. mexicana*  LSMGANELAAGKAGAT--GRLSSTGT----------------------AAPSANAFGGASSSAS---SSLVDILNSGSSLAAELNSRIAAFAAIVEEYAP

*T. cruzi*  FSLKA-------CSGG--GDVSRI------------------------KMSTSSLIPGGSMRPR---AGVRALLNGRDATSMRILNKIAGFANIVDTQPP

*T. brucei brucei*  ASLKA-------GSGG--GRGGNSRN------SISNGQLMHAS--TLGGAVNGVAAAGTALQSH---AGVVALLNGRDTASTKALAKVSAFAHIVHTQPP

*T. brucei gambiense* ASLKA-------GSGG--GRGGNSRN------SISNGQLMHAS--TLGGAVNGVAAAGTALQSH---AGVVALLNGRDTASTKALAKVSAFAHIVHTQPP

*T. evansi*  ASLKA-------GSGG--GRGGNSRN------SISNGQLMHAS--TLGGAVNGVAAAGTALQSH---AGVVALLNGRDTASTKALAKVSAFAHIVHTQPP

*T. congolense*  ASLRA-------KGSG--SRVSNL------------------------GKNDSTAGTAASLQRH---SGVIALLNGRDTVSVKVLNKISAFAHIVHTQPP

410 420 430 440 450 460 470 480 490 500

....|....|....|....|....|....|....|....|....|....|....|....|....|....|....|....|....|....|....|....|

jaculum1 SQWAGQCAAYVHEQHEMLLLQQEQQRQQQNDGQAHVTGSTPSVQRDSGAVTVLWRGVASLLSSVTRA---------AGTSASALTFAHAARVHLESRALR

jaculum2 REWISYFTTYVRDTPLPEINMNTSTTNT----------D-CNA---SNALTQLWCDVSDIFDELRRSEVKINDVRNTSIVDTEMRYLSASRSIIEQRAWR

*C. fasciculata*  LQWVSHFAAFVTE-------------------------ASRDA---ADELTVLWATVAQLLEGLQR----------RGSSADTLAYVAASRRVLERKSLS

*L. pyrrhocoris*  LQWVSYFTSYVTE-------------------------TSLDV---ADELTVLWATVAQILEAMQR----------RGAEADMLAYVAMSRRVMERKSLS

*L. seymouri*  LQWVSYFTSYVTE-------------------------ASPDV---ADELTVLWATVAQILEPLQR----------RGADVDMLSYVSMSRRVMERKSLS

*L. arabica*  AQWITHFTAYVTE-------------------------TSLDG---ANDLTVLWASIVQIIEPMQR----------RGSEADILSYVAGSRRVMERKSLS

*L. aethiopica*  AQWITHFTAYVTE-------------------------TSLDG---ANDLTVLWASIVQIIEPMQR----------RGSEADILSYVASSRRVMERKSLS

*L. donovani*  AQWITHFTAYVTE-------------------------TSLDG---ANDLTVLWASIVQIIEPMQR----------RGSEADILSYVASSRRVMERKSLS

*L. gerbilli*  AQWITHFTAYVTE-------------------------TSLDG---ANDLTVLWASIVQIIEPMQR----------RGSEADILSYVASSRRVMERKSLS

*L. enriettii*  AQWAAHFTAYVTE-------------------------TSLNG---ADDLTVLWASIVQIIEPMQR----------RGSEADILSYVASSRRVMERKSLS

*L. infantum*  AQWITHFTAYVTE-------------------------TSLDG---ANDLTVLWASIVQIIEPMQR----------RGSEADILSYVASSRRVMERKSLS

*L. panamensis*  AQWVTHFTAYVTE-------------------------TSLNG---RDDLTVLWASIVQIIEPMQR----------RGSEVDILSYVASSRRVMERKSLS

*L. tropica*  AQWITHFTAYVTE-------------------------TSLDG---ANDLTVLWASIVQIIEPMQR----------RGSEADILSYVASSRRVMERKSLS

*L. turanica*  AQWITHFTAYVTE-------------------------TSLDG---ANDLTVLWASIVQIIEPMQR----------RGSEADILSYVASSRRVMERKSLS

*L. major*  AQWITHFTAYVTE-------------------------TSLDG---ANDLTVLWASIVQIIEPMQR----------RGSEADILSYVASSRRVMERKSLS

*L. mexicana*  AQWIKHFTAYVTE-------------------------TSLDG---ANDLTVLWTSIVQIIEPMQR----------RGSEADIMSYVASSRRVMERKSLS

*T. cruzi*  LQWIRCFAAHVAD-------------------------DAPSV---TDDIALLWTTVEQIVQPILK----------LGSASTTLTFVASSRHMIERKALI

*T. brucei brucei*  QKWSSHFTNHVVD-------------------------DTPNS---VEEVAVLWTTVGHILQPIIE----------RGSAATSITYVSSSRRVMERKALA

*T. brucei gambiense* QKWSSHFTNHVVD-------------------------DTPNS---VEEVAVLWTTVGHILQPIIE----------RGSAATSITYVSSSRRVMERKALA

*T. evansi*  QKWSSHFTNHVVD-------------------------DTPNS---VEEVAVLWTTVGHILQPIIE----------RGSAATSITYVSSSRRVMERKALA

*T. congolense*  QKWMSYFAAHVVD-------------------------EAPRV---TDEVAVMWSTVEQILRPIME----------RGSASTIMTYVHSSRRVMERKALA

510 520 530 540 550 560 570 580 590 600

....|....|....|....|....|....|....|....|....|....|....|....|....|....|....|....|....|....|....|....|

jaculum1 ALQSRMRRLRGAVGAYDTASRWSAAQVLDALACVDDDDDGDKGEGAHDTRARTRAGRRAE----GSVGGGGGSAWQRVFAAMRVGRYDAAALAAQSVGIA

jaculum2 QIITHVQRERRLQHQHN----------------PDKDQGWHMDELFSASQAVDLIADYCAIHNNNNNNGSNSNPWEIIFHAMRAGRYDAALLAAESIGSV

*C. fasciculata*  RLLDRVLR-------------------------MDPMRFEEV-ENMNAAHVLDVVARYCG----------TANRWMHTFVAMRAGRYDVAKLAAQAIGVS

*L. pyrrhocoris*  RLLQRVLR-------------------------LDPMRFEEV-ENMNAAHVLDVVTRYCG----------TANHWMHTFVAMRAGRYDVAKLAAQTIGIT

*L. seymouri*  RLLERVLR-------------------------LDPLRAAEV-ENMNAAHVLDVVTRYCG----------TANHWMHAFVAMRAGRYDVAKLAAQTIGIA

*L. arabica*  RLLERVLR-------------------------MDPMRFEEV-ENMSASHLMDVIARSCG----------ATNHWMHAFVAMRVGRYDVAQLAMDAIGIQ

*L. aethiopica*  RLLERVLR-------------------------MDPMRFEEV-ENMSASHLMDVIARSCG----------ATNHWMHAFVSMRVGRYDVAQLAMDAIGIQ

*L. donovani*  RLLERVLR-------------------------MDPMRFEEV-ENMSASHLMDVIARSCG----------VTNHWMHTFVAMRVGRYDVAQLAMDAIGIQ

*L. gerbilli*  RLLERVLR-------------------------MDPMRFEEV-ENMSASHLMDVIARSCG----------ATNHWMHAFVAMRVGRYDVAQLAMDAIGIQ

*L. enriettii*  RLLERMLR-------------------------MDPVRFEEV-ENMSASRLMDVIARSCS----------SANHWMHAFVAMRVGRYDVAQLAMDAIGIQ

*L. infantum*  RLLERVLR-------------------------MDPMRFEEV-ENMSASHLMDVIARSCG----------VTNHWMHTFVAMRVGRYDVAQLAMDAIGIQ

*L. panamensis*  RLLERMLR-------------------------MDPIRFEEV-ENLSASHLLDIIARSCG----------ATNHWMHAFVAMRVGRYDVAQLATDAIGIQ

*L. tropica*  RLLERVLR-------------------------MDPMRFEEV-ENMSASHLMDVIARTCG----------ATNHWMHAFVAMRVGRYDVAQLAMDAIGIQ

*L. turanica*  RLLERVLR-------------------------MDPMRFEEV-ENMSASHLMDVIARSCG----------ATNHWMHAFVAMRVGRYDVAQLATDAIGIQ

*L. major*  RLLERVLR-------------------------MDPIRFEEV-ENMSASHLMDVIARSCG----------ATNHWMHAFVAMRVGRYDVAQLVMDAIGIQ

*L. mexicana*  RLLERVLR-------------------------MDPMRFEEV-ENMSALHLMDVIARSCG----------ATNPWMHAFVAMRVGRYDVAQLAMDAIGIQ

*T. cruzi*  TVLSRILK-------------------------VEPERFGEL-ENMHATRVVSILERYTS----------SSNPWAHIYAAMRCGRYDVAAMVANNVGLR

*T. brucei brucei*  AVFSIMLK-------------------------VESDRLSDM-ENMHATRFIGVVERYTS----------SSNPWAHIFTSMRCGRYDAAAAIAAAAGFG

*T. brucei gambiense* AVFSIMLK-------------------------VESDRLSDM-ENMHATRFIGVVERYTS----------SSNPWAHIFTSMRCGRYDAAAAIAAAAGFG

*T. evansi*  AVFSIMLK-------------------------VESDRLSDM-ENMHATRFIGVVERYTS----------SSNPWAHIFTSMRCGRYDAAAAIAAAAGFG

*T. congolense*  AVLSVTLK-------------------------VDPERLSDL-ENMHASRLVSIVERYTS----------SSNPWVHIFTAMRCGRYDAASIAANSAGFT

610 620 630 640 650 660 670 680 690 700

....|....|....|....|....|....|....|....|....|....|....|....|....|....|....|....|....|....|....|....|

jaculum1 AVVHALERAAAA-------------------------------------VSPLARCA-LPPALELQALYKSNGSSGSSDNGNNSKDAYDVYRQAVLFVLL

jaculum2 ALTRALERFTSFDITSVNNYGDGDGDGGLVSYDDSTIDHTGSINNNTKQLHSMKIAL-MPPYMELQILYNEAVTN------------NDPFRKAVLFVLL

*C. fasciculata*  AVTEAVEKMAAA--------------------------------------PPAERNA-LPPAMDLQPIYAEAATR------------EDPYRRAVLLILL

*L. pyrrhocoris*  AVTDAMEKMTAA--------------------------------------APGERNA-LSPAMDLQTVYAEAATK------------EDPYRQAVLMILL

*L. seymouri*  AVADAIDVMAAT--------------------------------------APMQRNT-LSPAVDLQTVYAEANTK------------EDPYRQAVLMILL

*L. arabica*  SVRDAMAKMAAT--------------------------------------PPAERNA-MPPASDLQPVYAEAKTK------------EDPYRQAVLMVLL

*L. aethiopica*  SVRDAMAKMAAT--------------------------------------PPAERNA-IPPASDLQPVYAEAKTK------------EDPYRQAVLMVLL

*L. donovani*  SVRDAMAKMAAT--------------------------------------PPAERNA-MPPASDLQPVYAEAKTK------------EDPYRQAVLMVLL

*L. gerbilli*  SVRDAMVKMAAT--------------------------------------PPAERNA-MPPASDLQPVYAEAKTK------------EDPYRQAVLMVLL

*L. enriettii*  SVRDAVAAVAAA--------------------------------------PPAERNA-MPPASDLQPIYAEAKTK------------EDPYRQAVLMVLL

*L. infantum*  SVRDAMAKMAAT--------------------------------------PPAERNA-MPPASDLQPVYAEAKTK------------EDPYRQAVLMVLL

*L. panamensis*  SVRDAVAKVAAA--------------------------------------PPAERNT-MPPASDLQPVYAEAKTK------------EDPYRQAVLIVLL

*L. tropica*  SVRDAMAKMVAT--------------------------------------PPAERNA-MPPASNLQPVYAEAKTK------------EDPYRQAVLMVLL

*L. turanica*  SVRDAMAKMAAT--------------------------------------PPAERNA-MPPASDLQPVYAEAKTK------------EDPYRQAVLMVLL

*L. major*  SVRDAMAKMAAT--------------------------------------PPAERNA-MPPASDLQPVYAEAKTK------------EDPYRQAVLMVLL

*L. mexicana*  SVRDAMAKMTAT--------------------------------------PPAERNA-MPPASDLQPVYAEAKTK------------EDPYRQAVLLVLL

*T. cruzi*  SVEEKLEEYANA--------------------------------------HMTRRST-LPPAVELRPLYEEEDTR------------LDPYRQIVLFLLL

*T. brucei brucei*  LVEKTLSSYASG--------------------------------------NVMEQYT-PSCPLELRALYSEDCTR------------SDPYRHTVLFLLL

*T. brucei gambiense* LVEKTLSSYASG--------------------------------------NVMEQYT-PSCPLELRALYSEDCTR------------SDPYRHTVLFLLL

*T. evansi*  LVEKTLSSYASG--------------------------------------NVMEQYT-PSCPLELRALYSEDCTR------------SDPYRHTVLFLLL

*T. congolense*  TLEKNLKLYDTD--------------------------------------NITEQCTQASSLLELRTLYSEEATR------------SDPYRHIVLFLLL

710 720 730 740 750 760 770 780 790 800

....|....|....|....|....|....|....|....|....|....|....|....|....|....|....|....|....|....|....|....|

jaculum1 AGD-----TGEANDSTDHN-------------GDAASSRTLLHLCGRVAQSLEQALWLRLACVRRIDDA----------HTRSSNT----SGNVC-VRVQ

jaculum2 VGKITPESTSANAESHVNNSLSSSSITTTKTMNNKSIRETLLELCGKTCSSIEDVLWLRLANIREVSQTPEQTQEMSSCNNNNNNNNDSSNNTST-DSVQ

*C. fasciculata*  AGR-----TGESDA---------------------AVLREMLSLSQSITDSLEDALWLRLSCIRGV------------------------ETSAKVSTIQ

*L. pyrrhocoris*  AGR-----TGESSA---------------------AVLRAMLSLSQRITDSLEDALWLRLSCIRGV------------------------EESAQVSTVQ

*L. seymouri*  AGR-----TGESSA---------------------VVLRTMLNLSQRITDSLEDALWLRLSCIRGV------------------------EEGAQVSTVQ

*L. arabica*  AGR-----TGESPA---------------------AVLRTILSLAQRVTDSLEDALWLRLSCIRGV------------------------DKSAQVTPVQ

*L. aethiopica*  AGR-----TGESPA---------------------AVLRTILSLAQRVTDSLEDALWLRLSCIRGV------------------------DKSAQVTPVQ

*L. donovani*  AGR-----TGESPA---------------------AVLRTILSLAQRVTDSLEDALWLRLSCIRGV------------------------DKSAQVTPVQ

*L. gerbilli*  AGR-----TGESPA---------------------AVLRTILSLAQRVTDSLEDALWLRLSCIRGV------------------------DKSAQVTPVQ

*L. enriettii*  AGH-----TGENQA---------------------AVLRTMLSLEQQVTDSLEDALWLRLSCIRGV------------------------DKSPQVSLVQ

*L. infantum*  AGR-----TGESPA---------------------AVLRTILSLAQRVTDSLEDALWLRLSCIRGV------------------------DKSAQVTPVQ

*L. panamensis*  AGC-----TGENPA---------------------VVLRTLLNLAQRVTDSLEDALWLRLSCIRGV------------------------DKSAQVSPVQ

*L. tropica*  AGR-----TGESPA---------------------AVLRTILSLAQRVTDSLEDALWLRLSCIRGV------------------------DKSAQVTPVQ

*L. turanica*  AGR-----TGESPA---------------------AVLRTILSLAQRVTDSLEDALWLRLSCIRGV------------------------DKSAQVTPVQ

*L. major*  AGR-----TGESPA---------------------AVLRTILSLAQRVTDSLEDALWLRLSCIRGV------------------------DKSAQVTPVQ

*L. mexicana*  AGR-----TGESPA---------------------AVLRTILSLAQRVTDSLEDALWLRLSCIRGV------------------------DKSAQATPVQ

*T. cruzi*  LGN-----TGENSE---------------------VVLSTVASLSSKVARSLEDTLWIRLFCVRSI------------------------EVRGN-NKVQ

*T. brucei brucei*  AGK-----TGESNE---------------------VVQSTVASLSSKVARSLEDTLWIRLFCLHTV------------------------DANNS-EKIQ

*T. brucei gambiense* AGK-----TGESNE---------------------VVQSTVASLSSKVARSLEDTLWIRLFCLHTV------------------------DANNS-EKIQ

*T. evansi*  AGK-----TGESNE---------------------VVQSTVASLSSKVARSLEDTLWIRLFCLHTV------------------------DANNS-EKIQ

*T. congolense*  AGK-----TGESND---------------------VIQSTVAALSSRVARSLEDTLWIRLFCIRSV------------------------DPKDD-NKIQ

810 820 830 840 850 860 870 880 890 900

....|....|....|....|....|....|....|....|....|....|....|....|....|....|....|....|....|....|....|....|

jaculum1 SLTHVQQVILDDMASIVALARGDVLQLAHFLLHAVLPSTALRLLLERDATHIDGLHMALCLTQCNALH---MTTAGATTAAPGDAAAMCNKSAASSNAHA

jaculum2 SLRTLQRTIYNDITNLVQLTNGDVIRLARFLIHALLPCSALRLLLERALTFVDGFHLAMCFDVERLLH---ISP--------------------------

*C. fasciculata*  SLRTLQRAVLDDMQELVTITQGSACRLASFLLHALLPSTGLRLLLENTYTHVDGVHMALCFNAQNLLQGGLNSP--------------------------

*L. pyrrhocoris*  SLRTLQRAVLDDLQELVTLTQGSACRLASFLIHALLPSAGLRLLLENNYTHVDGFHMALCFNAQNLLQEGLSSP--------------------------

*L. seymouri*  SLRTLQSAVLDDLQELVSLTQGSTCRLASFLIHALLPSTGLRLLLENNYTYVDGFHMALCFNAQNLLQEGLNSS--------------------------

*L. arabica*  SLRTLQRAVLDDMQDLVSITQGNACRLASFLIHALLPSTGLRLLLENNITHVDGFHMALCFSAQSLLQGRLHSA--------------------------

*L. aethiopica*  SLRTLQRAVLDDMQDLVSITQGNACRLASFLIHALLPSTGLRLLLENNITHVDGFHMALCFSAQNLLQGRLHSA--------------------------

*L. donovani*  SLRTLQRAVLDDMQDLVSITQGNACRLASFLIHALLPSTGLRLLLENNITHVDGFHMALCFSAQNLLQGRLHSA--------------------------

*L. gerbilli*  SLRTLQRAVLDDMQDLVSITQGNACRLASFLIHALLPSTGLRLLLENNITHVDGFHMALCFSAQSLLQGRLHSA--------------------------

*L. enriettii*  SLRTLQRAVLDDMQELVSITQGNACRLASFLIHALLPSTGLRLLLENSITHVDGFHMALCLSAQNLLQGRLHSA--------------------------

*L. infantum*  SLRTLQRAVLDDMQDLVSITQGNACRLASFLIHALLPSTGLRLLLENNITHVDGFHMALCFSAQNLLQGRLHSA--------------------------

*L. panamensis*  SLRTLQRAVLDDMQELVSITQGNAYRLASFFIHALLPSTGLRLLLENSITHVDGFHMTLCFNAQNLLQGRLHSA--------------------------

*L. tropica*  SLRTLQRAVLDDMQDLVSITQGNACRLASFLIHALLPSTGLRLLLENNITHVDGFHMALCFSAQNLLQGRLHSA--------------------------

*L. turanica*  SLRTLQRAVLDDMQDLVSITQGNACRLASFLIHALLPSTGLRLLLENNITHVDGFHMALCFSAQSLLQGRLHSA--------------------------

*L. major*  SLRTLQRAVLDDMQDLVCITQGNACRLASFLIHALLPSTGLRLLLENNITHVDGFHMALCFSAQSLLQGRLHSA--------------------------

*L. mexicana*  SLRTLQRAVLDDMQDLVSITQGNACRLASFLIHALLPSTGLRLLLENNITHVDGFHMALCFSAQNLLQGRLHSA--------------------------

*T. cruzi*  SLTEMQRLLLDDMQELVELVRGDVVRLASLLIHALLPSSGLRLLIEGDSTYVDGIHLAMCFHNSQLLP---CDD--------------------------

*T. brucei brucei*  SLSDMQRLLLDDMQDLVALTRGNVVRLASLMFHALLPSSGVRLLTENDSTYVDGVHLAMCFHNSKMLQ---CSD--------------------------

*T. brucei gambiense* SLSDMQRLLLDDMQDLVALTRGNVVRLASLMFHALLPSSGVRLLTENDSTYVDGVHLAMCFHNSKMLQ---CSD--------------------------

*T. evansi*  SLSDMQRLLLDDMQDLVALTRGNVVRLASLMFHALLPSSGVRLLTENDSTYVDGVHLAMCFHNSKMLQ---CSD--------------------------

*T. congolense*  SLSEMQRLLLDDMQDLVALVRGNVVRLASLMFHALLPSSGVRLLTENDITYIDGVHLAVCFHNCKVLH---CSE--------------------------

910 920 930 940 950 960 970 980 990 1000

....|....|....|....|....|....|....|....|....|....|....|....|....|....|....|....|....|....|....|....|

jaculum1 STLLDAPLDLSRYVSRYCMQVLLPLSQG----APTMRVPLRAVYAYFAKT---NHVHALVELCMQHDTLCTRLLGARGQQ--------------------

jaculum2 ---IERDIHVAQLLQEYTTMILLPLHRNRFGHHTSPNISVFTILHYFIRATDPQAINALVELC-QSERFFAQLFGQCTLETLSAPSIHSVLHWEPHPSQE

*C. fasciculata*  ---LEAPIDLARHLSRYSSVVLLDVDRR----TRSAAFAGSAMFAYFYKS---GLTEAFVECC-LKDTICARLIGPRT----------------------

*L. pyrrhocoris*  ---LEVPIDLARHMSRYCSVVLLDVDRR----TRAAAFAGTAMFAYFYKS---GLTDAFVECC-LKDTICARLLGSRA----------------------

*L. seymouri*  ---LEVPIDLSRNLSRYSSVVLLDVDRR----TRAAAFAGTAMFAYFHKS---GFTEAFAECC-LKDTICARLLGPRT----------------------

*L. arabica*  ---LEAPIDIARHMNRYCSVVLLGVDRR----IRATVFAGTAIFAYFHKS---GLTEAFVECC-LKDTICTRLLGSRN----------------------

*L. aethiopica*  ---LEAPIDIARHMNRYCSVVLLGVDRR----IRATVFAGTAIFAYFHKS---GLTEAFVECC-LKDTICARLLGSRN----------------------

*L. donovani*  ---LEAPIDIARHMSRYCSVVLLGVDRR----LPATVFAGTAIFAYFHKS---GLTEAFVECC-LKDVICARLLGSRN----------------------

*L. gerbilli*  ---LEAPIDIARHMNRYCSVVLLGVDRR----IRATVFAGTAIFAYFHKS---GLTEAFVECC-LKDTIFARLLGSRN----------------------

*L. enriettii*  ---LEAPIDIARHMSRYCSVVLLDINRR----IRAAVFAGTAMFAYFHKS---GLTEAFVELC-SKDIICARLLGSRN----------------------

*L. infantum*  ---LEAPIDIARHMSRYCSVVLLGVDRR----LPATVFAGTAIFAYFHKS---GLTEAFVECC-LKDVICARLLGSRN----------------------

*L. panamensis*  ---LEAPIDIARHMSRYCSVVLLDVDRR----IRATVFAGTAMFAYFHKS---GLTEAFVECC-LKDLICARLLGPRN----------------------

*L. tropica*  ---LEAPIDIARHMSRYCSVVLLGVDRR----IRATAFAGTAIFAYFHKS---GLTEAFVECC-LKDTICARLLGSRN----------------------

*L. turanica*  ---LEAPIDIARHMNRYCSVVLLGVDRR----IRATVFAGTAIFAYFHKS---GLTEAFVECC-LKDTICARLLGSRN----------------------

*L. major*  ---LEAPIDIARHMNRYCSVVLLGVDRR----IRATVFAGTAIFAYFHKS---GLTEAFVECC-LKDTICARLLGSRN----------------------

*L. mexicana*  ---LEAPIDIARHMNRYCSVVLLGVDRR----IRATVFAGTAIFAYFHKS---GLTEAFVECC-LKDIICARLLGSRN----------------------

*T. cruzi*  ---VEVPIDLGRLLPQYCSLVLLDVDKR----HARTAYAGAAVFRYFLRT---NLVDVFVEYC-GNELVCAKLFGHRG----------------------

*T. brucei brucei*  ---AEVPLDLSRAIQQYCSIALLDADRR----RMNASQVGPAIFWYFFRT---GLIDTFVDYC-SNELVCAKLFGQRA----------------------

*T. brucei gambiense* ---AEVPLDLSRAIQQYCSIALLDADRR----RMNASQVGPAIFWYFFRT---GLIDTFVDYC-SNELVCARLFGHRA----------------------

*T. evansi*  ---AEVPLDLSRAIQQYCSIALLDADRR----RMNASQVGPAIFWYFFRT---GLIDTFVDYC-SNELVCARLFGHRA----------------------

*T. congolense*  ---AEVPLDLSRVTQQYCTIALLDADKR----QLHAPHVGPAIFWYFCHT---GLSDAFVDYC-NNELVCAKLFGQRA----------------------

1010 1020 1030 1040 1050 1060 1070 1080 1090 1100

....|....|....|....|....|....|....|....|....|....|....|....|....|....|....|....|....|....|....|....|

jaculum1 ---LHVAAQEA-NGDDASASAH-DEMTNTALMCMRAVAKHAAARGRALLAAQANIAI--------VAHTRDTPRAPRDVPAALRRAVCVLVPALAHAMHN

jaculum2 SQLLHDP-LAG-EVN-AKNNVS-ESSLRILVDTLTRVAHAIALRGNVVRACFVYLFIVQTLMQNAINHELSQSSL-EYCVGAINSVVELLLPAIAQVFYL

*C. fasciculata*  --------AVG-GQDALLLRAGPSSLSEELLKALLQVAEAAAARSEVGKATHVLLAV-----TYLADQLHRVELQ----QRALLRAVQMISPAIAQVLHL

*L. pyrrhocoris*  --------AIG-GQDALLLRATASPLSEELLKALIQVAEAASARSEVAKATHVLVAV-----MYLAEQLGRAELQ----QQALLRAVQIISPALAQVLHL

*L. seymouri*  --------AIG-GQEALLLRAGANPLSEELLQALIQVAEAASARSEVAKAIHVLLAV-----TYLATQLGRTELQ----QRALLRAVRMISPALAQVLHL

*L. arabica*  --------AVG-GQDALLLRAGTGALSEELLKALMQVAEAASARSEVAKATHVLLAV-----AFLASQLCRPELQ----QRALRRAVQMMSPALAQVLHL

*L. aethiopica*  --------AVG-GQDALLLRAGTGALSEELLKALMQVAEAASARSEVAKATHVLLAV-----AFLASQLCRPELQ----QRALRRAVQMMSPALAQVLHL

*L. donovani*  --------AVG-GQDALLLRAGAGALSEELLKALMQVAEAASARSEVAKATHVLLAV-----AFLASQLCRPELQ----QRALRRAVQMMSPALAQVLHL

*L. gerbilli*  --------AVG-GQDALLLRAGTGALSEELLKALMQVAEAASARSEVAKATHVLLAV-----AFLASQLCRPELQ----QRALRRAVQMMSPALAQVLHL

*L. enriettii*  --------SMS-GQDPLLLRTGTGALSEELLKALIQVAEAASARSEVAKATHVLLAV-----ALLSSQLRRPELQ----QRALRRAVQMMSPALAQVLHL

*L. infantum*  --------AVG-GQDALLLRAGAGALSEELLKALMQVAEAASARSEVAKATHVLLAV-----AFLASQLCRPELQ----QRALRRAVQMMSPALAQVLHL

*L. panamensis*  --------TVG-GYDSLLLRAGTGALSEELLKALIQVAEAASARSEVAKATHVLLAV-----ACLANQLHRPELQ----QRALRRAVQMMSPALAQVLHL

*L. tropica*  --------AVG-GQDALLLRAGTGALSEELLKALMQVAEAASARSEVAKATHVLLAV-----AFLASQLCRPELQ----QRALRRAVQMMSPALAQVLHL

*L. turanica*  --------AVG-GQDALLLRAGTGALSEELLKALMQVAEAASARSEVAKATHVLLAV-----AFLASQLCRPELQ----QRALRRAVQMMSPALAQVLHL

*L. major*  --------AVG-GQDALLLRAGTGALSEELIKALMQVAEAASARSEVAKATHVLLAV-----AFLASQLCRPELQ----QRALRRAVQMMSPALAQVLHL

*L. mexicana*  --------AVG-GQDALLLRAGTRALSEELLKALMQVAEDASARSEVAKATHVLLAV-----AFLASQFCRPELQ----QRALRRAVQMMSPALAQVLHL

*T. cruzi*  --------GSRVGGDGILLQE---TPSTELLEAMERVAEAGAARGQTELAVHVFSVL-----ERAAALVKDEARA----NYALSRALQIICPALSHAFQQ

*T. brucei brucei*  --------GGG-SNDGALFQHG-GVPPNELLDAMVRIAEDAIARGKTELAVHVLTVL-----DHAATLVSDDARS----GYALSRAVQKICPALAQAFHN

*T. brucei gambiense* --------GGG-SNDGALFQHG-GVPPNELLDAMVRIAEDAIARGKTELAVHVLTVL-----DHAATLVSDDARS----GYALSRAVQKICPALAQAFHN

*T. evansi*  --------GGG-SNDGALFQHG-GVPPNELLDAMVRIAEDAIARGKTELAVHVLTVL-----DHAATLVSDDARS----GYALSRAVQKICPALAQAFHN

*T. congolense*  --------GGG-HSDGALFQHG-GAPSNEVLDAMERIAEGAASRGKTELAVHVLTTL-----ESAASLLSDDVRC----GYALSRAVQIICPALAQAFHQ

1110 1120 1130 1140 1150 1160 1170 1180 1190 1200

....|....|....|....|....|....|....|....|....|....|....|....|....|....|....|....|....|....|....|....|

jaculum1 APTSRVVADTFALAADVYELVVQQG------VSMQRTAVHAADAAQRDAYNTTSDNMNNTNDIDDDDDDDDDAQTTQQLRSIFEALCALARVYTAAARGN

jaculum2 AHMSAYTSELFDMLACVQKIINDLS------TCV--------KSSGKNVFECI--------DI-----------------ATTATFARMTEFYTFATRQQ

*C. fasciculata*  PPQSSAVSDVLSQAAQLLQVVRADEAHHLRADAL--------AQQDYQTF---------------------------------DLLCRVADVYAYGAQGN

*L. pyrrhocoris*  PSNSVAVSDVLSQAAKLLQVVNTDE-KHLQGNPL--------VSQDFQTF---------------------------------SLLCRMADVYAYNAQGN

*L. seymouri*  PPHSTVVSDVLSQAAQLRHVVGADE-KHLHSDPL--------VAQEYQNF---------------------------------GLLCRMADVYAYHAQGN

*L. arabica*  PSRSSVVSDVLSRAAQLQQAVNAVP-HHLSEEAI--------NAQDYQTF---------------------------------ALLCRMADVYAFNVSGN

*L. aethiopica*  PSRSSVVSDVLSRAAQLQQAVNAVP-HHLSEEAI--------SAQDYQTF---------------------------------ALLCRMADVYAFNVSGN

*L. donovani*  PSRSSVVSDVLSQAAQLQQAVNAVP-HHLNEEAI--------NAQDYQTF---------------------------------ALLCRMADVYAFNVSGN

*L. gerbilli*  PSRSSVVSDVLSRAAQLQQAVNAVP-HHLSEEAI--------NAQDYQTF---------------------------------ALLCRMADVYAFNVSGN

*L. enriettii*  PARSSVVSDVLSQAAQLQQAVNAVP-HHLQDESI--------GAQDYQTF---------------------------------AQLCRMADVYAFNVNGN

*L. infantum*  PSRSSVVSDVLSQAAQLQQAVNAVP-HHLNEEAI--------NAQDYQTF---------------------------------ALLCRMADVYAFNVSGN

*L. panamensis*  PSRSSVVSDVLSQAAQLQQAVNAVP-HHLHDEAI--------GAQDYQTF---------------------------------AQLCRMADVYAFNVNGN

*L. tropica*  PSRSSVVSDVLSRAAQLQQAVNAAP-HHLSEEAI--------NAQDYQTF---------------------------------ALLCRMADVYAFNVSGN

*L. turanica*  PSRSSVVSDVLSRAAQLQQAVNAVP-HHLSEEAI--------NAQDYQTF---------------------------------ALLCRMADVYAFNVSGN

*L. major*  PSRSSVVSDVLSRAAQLQQAVNAVP-RHLSEEAV--------DAQDYQTF---------------------------------ALLCRMADVYAFNVSGN

*L. mexicana*  PSRSSVVSDVLSQAAQLQQAVNTAP-HHLNEDAI--------NAQDYQTF---------------------------------AQLCRMADVYAFNVNGN

*T. cruzi*  KASRE-STCLFMHAAELQERVACSE------RVI--------PHAYAEAF---------------------------------QLLCTMSEFFVSVARGE

*T. brucei brucei*  EPTSE-SANLFVHAAVLRERLAQTK------CVI--------PSAQTDTF---------------------------------HLLCRMGEVHANAVRGN

*T. brucei gambiense* EPTSE-SANLFVHAAVLRERLAQTK------CVI--------PSAQTDTF---------------------------------HLLCRMGEVHANAVRGN

*T. evansi*  EPTSE-SANLFVHAAVLRERLAQTK------CVI--------PSAQTDTF---------------------------------HLLCRMGEVHANAVRGN

*T. congolense*  DSSSA-GTNLFAHASILQERLARAK------SPI--------SSSHTDAF---------------------------------RLLCKMGDVFSSAARGD

1210 1220 1230 1240 1250 1260 1270 1280 1290 1300

....|....|....|....|....|....|....|....|....|....|....|....|....|....|....|....|....|....|....|....|

jaculum1 QREVLQLFFALPFVPQK-----AEQTRHVTPFSSLPKDIVVALRALVPHVLHAAASRLQELAQYTRTYMHDDNTTVEANARDVSNDMLDVQVNTSALCSW

jaculum2 GESALHSFFALSFIPHS---MVDDLNECVYKYINVHSVIKLATNALLPLAFNAADSVLEQIKQ--NAHVNDNLISSDTRLR-ESEAAMSILQMMKVVCEW

*C. fasciculata*  ASDAVDAFLQLPLVPAPHTATAADVEMYVKAYCSAPACVQLGISAVLPVAVEAAGQVLHHYAR-----SRAN--GAD-----VEEQQAHLLQRMRSVCEW

*L. pyrrhocoris*  FSDAVNAFLQLPFVPASDSAMMGDADTYVDAYCNAPGNVQLGVSAVFPIVVSAAGQLLQLYAR-----SRAN--GGD-----VEERQTHLLQRMRSVCDW

*L. seymouri*  VSDAVSTFLQLPFVPPPNSATAGDAETYVEAYCNASGSTQLGASAVFPIAVSAASQLLQMYAR-----SRAN--GGD-----VEEKQARLLQRMRSVCDW

*L. arabica*  ASDAVDAFLQLPFVPPPNTAVTGGVDTYVEAYCNAPGSVQLGASAALRIAISAAGQLLQHYAR-----NSTG-GGAD-----VDEKRLRLLHRMQTLLEW

*L. aethiopica*  PSDAVDAFLQLPFVPPPKTAVTGGVDDYVEAYCNAPGSVQLGASAALQIAISAAGQLLQHYAR-----NSTG-GGAD-----VDEKRLRLLHRMQTVLEW

*L. donovani*  FSDAVDAFLQLPFVPPPNTAVTGGVDAYVEAYCNAPGSVQLGASAALRIAISAAGKLLQYYAR-----SSTG-GGAD-----VDEKRLRLLHRMQTVLEW

*L. gerbilli*  ASDAVDAFLQLPFVPPPNTAVTGGVDTYVEAYCNAPGSVQLGASAALRIAISAAGQLLQHYAR-----NSTG-GGAD-----VDEKRLRLLHRMQTLLEW

*L. enriettii*  FSDAVDAFLQLPFVPPPNTAVTGGVDTYVEAYCNAPGSVQLGASATLIIAVSAAGQLLQFYAR-----SSSS--GTD-----VDDKRLRLLHRMQTVLEW

*L. infantum*  FSDAVDAFLQLPFVPPPNTAVTGGVDAYVEAYCNAPGSVQLGASAALRIAISAAGKLLQYYAR-----SSTG-GGAD-----VDEKRLRLLHRMQTVLEW

*L. panamensis*  VSDAVDAFLQLPFVPPPNTAVTGGVDAYVEAYCNAPGSVQLGASAALRVAISAAGQLLQYYAR-----NSTG-DSAD-----VDDKRLRLLHRMQTVLEW

*L. tropica*  PSDAVDAFLQLPFVPPPNTAVTGGVDAYVEAYCNAPGSVQLGASAALRIAISAAGQLLQHYAR-----NSTG-GGAD-----VDEKRLRLLHRMQTVLEW

*L. turanica*  ASDAVDAFLQLPFVPPPNTAVTGGVDTYVEAYCNAPGSVQLGASAALRIAISAAGQLLQHYAR-----NSTG-GGAD-----VDEKRLRLLHRMQTLLEW

*L. major*  ASDAVDAFLQLPFVPPPNTAVTGGVDAYVEAYCNAPGSVQLGASAVLRIAISAAGQLLQHYAR-----NITG-GGAD-----VDEKRPRLLHRMQTLLEW

*L. mexicana*  VSDAVDAFLQLPFVPPPNTAVTGGVDAYVEAYCNAPGSVQLGARAALRIAISAAGQLLQHYAR-----NSTS-GGAD-----VDEKRLRLLHRMQTVLEW

*T. cruzi*  VEVALRCFWSLPFVPTA----PDDIERCAEIFDSAPESVATAAPPIVLLAMQNMLKLAEVLKT-------RG----------QTNDATQIKRRAQTVTMW

*T. brucei brucei*  AEMAVRCFCNLPFVPAS----PADVERCAELFDTAPDTVATAAPPIIIHALRQLLRLVKEQRA-----LHDG----------ASDELLLQRNQAQQIIAW

*T. brucei gambiense* AEMAVRCFCNLPFVPAS----PADVERCAELFDTAPDTVATAAPPIIIHALRQLLRLVKEQRA-----LHDG----------ASDELLLQRNQAQQIIAW

*T. evansi*  AEMAVRCFCNLPFVPAS----PADVERCAELFDTAPDTVATAAPPIIIHALRQLLRLVKEQRA-----LHDG----------ASDELLLQRNQAQQIIAW

*T. congolense*  AMATLQCFLSLPFVPTT----PGDVERCAELFDTAPDVVATAVPPIVLLTMRQMLYLAKEQKT-----LHVG----------ASDTLLHVRSQAQQIVAW

1310 1320 1330

....|....|....|....|....|....|....|..

jaculum1 LIEDNQAQYNTANATMMEGIHAFMNQYMHD-----VM

jaculum2 V----RQIRTR--GEVDDVL-PECERVANQFERKYFL

*C. fasciculata*  A----RLCCARTSVAYPTVA-EDFERNYLR-------

*L. pyrrhocoris*  A----RQCCARISVEYPTAA-DDFERQYLG-------

*L. seymouri*  A----RQCCMRISVEYPMAA-EDFERHYLG-------

*L. arabica*  A----RQCCARTSVAYPAVA-EEFERTYLC-------

*L. aethiopica*  A----RQCCARTSVAYPAVA-EEFERAYLC-------

*L. donovani*  A----RQCCALTSVAYPAVA-EEFERVYMC-------

*L. gerbilli*  A----RQCCARTSVAYPAVA-EEFERTYLC-------

*L. enriettii*  A----RQCCVRTSVAYPTVA-EEFERAYLS-------

*L. infantum*  A----RQCCALTSVAYPAVA-EEFERVYMC-------

*L. panamensis*  A----RQCCARTSVAYPAVA-EEFERAYLC-------

*L. tropica*  A----RQCCARTSVAYPAVA-EEFERAYLC-------

*L. turanica*  A----RQCCARTSVAYPAVA-EEFERTYLC-------

*L. major*  A----RQCCARTSVAYPAVA-EEFERTYLC-------

*L. mexicana*  A----RQCCARTSVAYPAVA-EGFERAYLC-------

*T. cruzi*  V----RRWKRHVARSLIDEL-ASLEELFAL-------

*T. brucei brucei*  V----RRWKRHMNRSLLDEL-IVLERLFVQ-------

*T. brucei gambiense* V----RRWKRHMNRSLLDEL-IVLERLFVQ-------

*T. evansi*  V----RRWKRHMNRSLLDEL-IVLERLFVQ-------

*T. congolense*  V----RRWRARASGSLVEEL-LVLERSLMQ-------

**J) mitochondrial ATP synthase subunit**

10 20 30 40 50 60 70 80 90 100

....|....|....|....|....|....|....|....|....|....|....|....|....|....|....|....|....|....|....|....|

jaculum1 MAARTL-----TLVRAGASAALMQTAAALVSKAVGGRSGGSSSIMTRVADAATDDVVPVASARAADKDTSAAAATTTDAAADA-----------------

jaculum2 M-SKSVKILTTTLNAAAFVEALSQVATEIISAVTNG-----------------------------------NVAVSTSSAKSNDNNNTTMNNHTSSKVDR

*C. fasciculata*  M-SKSI-----AVVAGGASAALLQAASAIASKATNG-----------------------------------AVKVSQAASAAS-----------------

*L. pyrrhocoris*  M-SKSI-----SVIAVAAPAALLQAASAIVSKATGG-----------------------------------AVKVTQTAAATA-----------------

*L. seymouri*  M-SKSI-----SVIAANAPAALLQAASAIVSKATGG-----------------------------------AVKVSQTASASA-----------------

*L. arabica*  M-SKSI-----AVIAAGAPAALVQAASAMVSKATGG-----------------------------------AVKAMQATSAAS-----------------

*L. aethiopica*  M-SKSI-----AVIAAGAPAALVQAASAMVSKATGG-----------------------------------AVKAMQATSAAS-----------------

*L. donovani*  M-SKSI-----AVIAAGAPAALVQAASAMVSKATGG-----------------------------------AVKAMQATSAAS-----------------

*L. gerbilli*  M-SKSI-----AVIAAGAPAALVQAASAMVSKATGG-----------------------------------AVKAMQATSAAS-----------------

*L. enriettii*  M-SKSI-----ALIATSAPAALVNAASALVSKATGG-----------------------------------AVKATPTTSAAS-----------------

*L. infantum*  M-SKSI-----AVIAAGAPAALVQAASAMVSKATGG-----------------------------------VVKAMQATSAAS-----------------

*L. panamensis*  M-SKSI-----AIIAAGAPAALVQAASAMVSKATCG-----------------------------------AVKTTQATSAAS-----------------

*L. tropica*  M-SKSI-----AVIAAGAPAALVQAASAMVSKATGG-----------------------------------AVKAMQATSAAS-----------------

*L. turanica*  M-SKSI-----AVIAAGAPAALVQAASAMVSKATGG-----------------------------------AVKAMQATSAAS-----------------

*L. major*  M-SKSI-----AVIAAGAPAALVQAASAMVSKATGG-----------------------------------AVKAMQATSAAS-----------------

*L. mexicana*  M-SKSI-----AVIAAGAPAALVQAASAMVTKATGG-----------------------------------AVQAMQATSAAS-----------------

*T. cruzi*  M-SKQL-----SIISAGASAALLQAATAIVNKATAG-----------------------------------KLSTTMTSMAEI-----------------

*T. brucei brucei*  M-SKQL-----TFISAGATAAVLQSASAIVSKVAGG-----------------------------------RVQTKTAKEAGR-----------------

*T. brucei gambiense* M-PKQL-----TFISAGATAAVLQSASAIVSKVAGG-----------------------------------RVQTKTAKEAGR-----------------

*T. evansi*  M-SKQL-----TFISAGATAAVLQSASAIVSKVAGG-----------------------------------RVQTKTAKEAGR-----------------

*T. congolense*  M-SKQL-----TIVSAGAAAAVLQTASAIVSKVTGG-----------------------------------RVQTTTAKEAGR-----------------

110 120 130 140 150 160 170 180 190 200

....|....|....|....|....|....|....|....|....|....|....|....|....|....|....|....|....|....|....|....|

jaculum1 -------GVVVVGTAAPRGVYACVAEPP-SS--RACAPYDGVRTAVVRAVLPRQPSDQLQLRDVLDVYPAAGIACDEEQQRAREAFARASEVAAAAAAQR

jaculum2 DATSTSIKTMVIGTEPPCNVCAGLVQPS-ASHMKHAGPYAGVQTTIVRAILPRTHASPLPLRDILDVYHKAGINCTNDDAVAERSFAQAAQVAIAAAQQR

*C. fasciculata*  -------NAVVVGTAAPRGVYACVAEPS-S---ATSGPYAGVKTVVVRAVLPRGPSDAMQVRDVIDVYPASGIACEAEVAQAEANYKKAAQVAVEKAKAM

*L. pyrrhocoris*  -------NAVVVGTAAPPGVYACVAEPT-S---ATIGLYAGVQTVVVRAILPRGAPDTMQVRDILDVYPASGIACDAEVAKAEENIKKAAKVAVEKAKAL

*L. seymouri*  -------NAVVVGTAAPRGVYACVVEPS-S---AVSGPYAGVQTVVVRAVLPRSAPDTMQVRDIVDVYPASGIACHEEVTKVEASFKKAAQVAVERAKTL

*L. arabica*  -------NAIVVGMQAPRGVYACVAEPP-S---TASGPYAGVKTVVVRAILPRRAPDTMQVRDILDVYPASGITCEKEAAEAVESFSKAAKVAVEKAKAM

*L. aethiopica*  -------NAIVVGMQAPRGVYACVAEPP-S---TASGPYAGVKTVVVRAILPRRAPDTMQVRDILDVYPASGITCEKETAEAVESFSKAAKVAVEKAKAM

*L. donovani*  -------NAIVVGMQAPLGVYACVAEPP-S---TASGPYAGVKTVVVRAILPRRAPDTMQVRDILDVYPASGITCEKETAEAVENFNRAAKVAVEKAKAM

*L. gerbilli*  -------NAIVVGMQAPLGVYACVAEPP-S---TASGPYAGVKTVVVRAILPRRAPDTMQVRDILDVYPASGITCEKETAEAVESFSKAAKVAVEKAKAM

*L. enriettii*  -------NAIVVGMQAPRGVYACVAEPP-S---ATSGAYAGVKTVMVRAILPRGAPDTMQVRDVVDVYPACGIACEEEAARAVENFTKAAKVAVEKAKGM

*L. infantum*  -------NAIVVGMQAPLGVYACVAEPP-S---TASDPYAGVKTVVVRAILPRRAPDTMQVRDILDVYPASGITCEKETAEAVENFNKAAKVAVEKAKAM

*L. panamensis*  -------NAVVVGMQAPRGVYACVTEPP-S---TPSEPYAGVKTVVVRAILPRGAPDTMQVRDVLDVYPASGIACEAETAKAVENFTKAAKVAVEKAKAM

*L. tropica*  -------NAIVVGMQAPRGVYACVAEPP-S---TASGPYAGVKTVVVRAILPRRAPDTMQVRDILDVYPASGITCEKETAEAVENFSKAAKVAVEKAKAM

*L. turanica*  -------NAIVVGMQAPRGVYACVAEPP-S---TTSGPYAGVKTVVVRAILPRRAPDTMQVRDILDVYPASGITCEKETAEAVESFSKAAKVAVEKAKAM

*L. major*  -------NAIVVGMQAPRGVYACVAEPP-S---TASGPYAGVKTVVVRAILPRRAPDTMQVRDILDVYPASGITCEQETAEAVESFSKAAKVAVEKAKAM

*L. mexicana*  -------NAIVVGMQAPRGVYACVAEPP-S---TTSGPYAGVKTVVVRAILPRSAPDTMQVRDILDVYPASGITCEKEIAEAVENFKKAAKVAVEKAKAM

*T. cruzi*  -------NAVVVGPETPIGVHTSVTAAP-A---SPDPAYAGVKTVLVRAVLPRVSPEKVQLRDALDVFVAAGINMDAEVKAATESFKRSAEVAVANAKAI

*T. brucei brucei*  -------HAVVVGPETPIGVHTAVTEAPKS---AQDPLFSGVSTVVVRAVLPRAAPDSVQLRDALDVYASAGIDTKEEVRSATEAFKKSAEVAVGKAKAK

*T. brucei gambiense* -------HAVVVGPETPIGVHTAVTEVPKS---AQDPLFSGVSTVVVRAVLPRAAPDSVQLRDALDVYASAGIDTKEEVRSATEAFKKSAEVAVGKAKAK

*T. evansi*  -------HAVVVGPETPIGVHTAVTEVPKS---AQDPLFSGVSTVVVRAVLPRAAPDSVQLRDALDVYASAGIDTKEEVRSATEAFKKSAEVAVGKAKAK

*T. congolense*  -------HAVVVGPETPAGVHTAVTEAPKV---LRDAIYSDVSTVLVRAVLPRGAPDTVQLRDALDVYASAGIDIKGEVEAATQSFKKSAEIAVAKAKAK

210 220 230 240 250 260 270 280 290 300

....|....|....|....|....|....|....|....|....|....|....|....|....|....|....|....|....|....|....|....|

jaculum1 AASRVTLVVKPVSKHKQLNELFLRTCSATLEAAGLSVDVLHTAQAANELLLFPERHAVVLVNDEATCERILYAYAGVTGGAATRFCA------DNGATLY

jaculum2 NANRVTLVIKPASKYERLNEFFVRVCGKTIEGAGLSVDTMHTAQASNELILFPEKHGVFLVNDEPTCTRMQLAYAGVIGGSSIKYVTAEANESQVASDMF

*C. fasciculata*  KATRVTLVTKPASKYQRLNALFRESATKVIEAAGLSVDAATTAQASNDLVMFPEKFGVVMVNDDPVCENVQFAYAGLVGGVHTTYYT------DAGSKIH

*L. pyrrhocoris*  KATRVTLVMKPASKYTRLNALFRESAAKVIEGAGLSVEVTTTAQASNDLVMFPEKHGVVMVNDDPVCEKVQFAYAGEVGGVHTTYYT------DAGGKIH

*L. seymouri*  RATRVALVTKPVSKYARLNALFRESATKVIEDAGLSVEVLTTAQASNDLIMFPERHNVVMLNDDPVCENVQFAYAGVVGGAHTTYYT------DAGGKIH

*L. arabica*  KETRVTLVMKPATKYERLNGLFRETCTKTIEAAGLSVETATTAAATNTLIMFPEKMNVVMVSDDPVCENVQYAFAGVVGGAHTTYYT------DAGSTIH

*L. aethiopica*  KATRVTLVMKPATKYERLNGLFRETCTKTIEAAGLSVETATTAAATNTLIMFPEKMSVVMVSDDPVCENVQYAFAGVVGGAHTTYYT------DAGSTIH

*L. donovani*  KATRVTLVMKPATKYERLNGLFRETCTKTIEAAGLSVETSTTAAATNTLIMFPEKMSVVMVSDDPVCENVQYAFAGVVGGVHTTYYT------DAGSKIH

*L. gerbilli*  KATRVTLVMKPATKYERLNGLFRETCTKTIEAAGLSVETATTAAATNTLIMFPEKMSVVMVSDDPVCENVQYAFAGVVGGAHTTYYT------DAGSTIH

*L. enriettii*  KSTRVTLVMKPATKYARLNSLFRETCTKTIEAAGLSVEITTTAAATNTLIMFPEKMSVVMVCDDPVCENVQYAYAGIIGGVHTTYYT------DAGCTIH

*L. infantum*  KATRVTLVMKPATKYERLNGLFRETCTKTIEAAGLSVETSTTAAATNTLIMFPEKMSVVMVSDDPVCENVQYAFAGVVGGVHTTYYT------DAGSKIH

*L. panamensis*  KASRVTLVVKPATKYERLNALFRETCTKTIEASGLSVEASTTAAAANILTMFPERMSVAMVCDDPVCENVQYAYAGIVGGVHTTYYT------DAGSKIH

*L. tropica*  KATRVTLVMKPATKYERLNGLFRETCTKTIEAAGLSVETATTAAATNTLIMFPEKMSVVMVSDDPVCENVQYAFAGVVGGAHTTYYT------DAGSTIH

*L. turanica*  KATRVTLVMKPATKYERLNGLFRETCTKTIEAAGLSVETATTAAATNTLIMFPEKMSVVMVSDDPVCENVQYAFAGVVGGAHTTYYT------DAGSTIH

*L. major*  KATRVTLVMKPATKYERLNSLFRETCTKTIEAAGLSVETATTAAATNTLIMFPEKMSVVMVSDDPVCENVQYAFAGVVGGAHTTYYT------DAGSTIH

*L. mexicana*  KAARVTLVMKPATKYERLNGLFRETCTKTIEAAGLSVETSTTAAATNTLIMFPEKMSVVMVSDDPVCENVQYAFAGVVGGVHTTYYT------DAGSKIH

*T. cruzi*  GVNRVTLVLKQATKYNNVNELFKKVSTETIEAAGMTTEIQNTSVATNQLIMFPESLGVVLLNDVTSTEKIELAYAGVLGGASRTYHT------VSGNKIS

*T. brucei brucei*  GVKRIVLVVKQASKHNCINELFKKISTETIESAGLTTEVVGTASVANQLIVNPESLGVVLLNDVAATEQIELAFAGVVGGVSRVYHT------VEGGKIS

*T. brucei gambiense* GVKRIVLVVKQASKHNCINELFKKISTETIESAGLTTEVVGTAVVANQLIVNPESLGVVLLNDVAATEQIELAFAGVVGGVSRVYHT------VEGGKIS

*T. evansi*  GVKRIVLVVKQASKHNCINELFKKISTETIESAGLTTEVVGTAVVANQLIVNPESLGVVLLNDVAATEQIELAFAGVVGGVSRVYHT------VEGGKIS

*T. congolense*  GVQRVVLVIKPVTKHNYINDLFKKVSTEAIEAAGLTTEVFGTSVAANQLIVNPETLGVVLLNDVVAAEHIELAFAGVVGGVSRVYYT------ADGGKIS

310 320 330 340

....|....|....|....|....|....|....|....|....|..

jaculum1 GGHSTRAVARALAATLHSLGMTAEAKRIAALADAGDVRGVAAAA---

jaculum2 GGYSYTSVAYAVANTLRELGLAKEATRVEEAVGN--ARNYQHITESL

*C. fasciculata*  GGHSYKSVATALAEELKALGMKAEAAKIEAAAQK-DSRNVVAGL---

*L. pyrrhocoris*  GGHSYKSVATALAEELKSLGLKAEAARIEAAAQK-DPRNVVDAI---

*L. seymouri*  GGHSYKSVATALAAELRSLGMKAEAAKIEAAAQK-DPRNVVGAL---

*L. arabica*  GGHSYKSVAMALAEELKSLGMKAEAAKVEAAAQK-SPRNAAAAM---

*L. aethiopica*  GGHSYKSVAMALAEELKSLGMKAEAAKVEAAAQK-SPRNAAAAM---

*L. donovani*  GGHSYKSVAMALAEELRSLGMKAEAAKVEAAAQK-SPRNAAAAM---

*L. gerbilli*  GGHSYKSVAMALAEELKSLGMKAEAAKVEAAAQK-SPRNAAAAM---

*L. enriettii*  GGHSYKSVAMALAEELKSLGMTGEAAKVEAAAQK-SPRNVAAAI---

*L. infantum*  GGHSYKSVAMALAEELRSLGMKAEAAKVEAAAQK-SPRNAAAAM---

*L. panamensis*  GGHSYKSVAMALAEELKSLGMKAEAAKVEAAAQK-SPRNAAAAL---

*L. tropica*  GGHSYKSVAMALAEELKSLGMKAEAAKVEAAAQR-SPRNAAAAM---

*L. turanica*  GGHSYKSVAMALAEELKSLGMKAEAAKVEAAAQK-SPRNAAAAM---

*L. major*  GGHSYKSVAMALAEELKSLGMKAEAAKVEAAAQK-SPRNAAAAM---

*L. mexicana*  GGHSYKSVALALAEELRSLGMKAEAAKVEAAAQK-SPRNAAAAM---

*T. cruzi*  AGHSFKSVALAVAQELRALGMGSEATKVEAAAAK-NPRAVLSSL---

*T. brucei brucei*  AGHSFKSVALAVAQELRELGLSSEADKVEAAASK-NPRAVVSAL---

*T. brucei gambiense* AGHSFKSVALAVAQELRELGLSSEADKVEAAASK-NPRAVVSAL---

*T. evansi*  AGHSFKSVALAVAQELRELGLSSEADKVEAAASK-NPRAVVSAL---

*T. congolense*  AGHSFKSVALAVAQELRELGLTSEAEKVQSAALK-NPRAVLAAL---

**K) cysteinyl-tRNA synthetase**

10 20 30 40 50 60 70 80 90 100

....|....|....|....|....|....|....|....|....|....|....|....|....|....|....|....|....|....|....|....|

jaculum1 MSATAAAPDGSSSSSNDAAPLRRVVDGLQADAPVKRDRHPPWYPPSQCSGMTASRRDGHGNTTSPTSPPLPTTTTATTTATTATATSSDDTDGNNSGGYV

jaculum2 M----------SSTTTPTATQLQFADGLQVNSPVLTDRHPPWYRPVQDDHK-------------------------------------------------

*C. fasciculata*  M-------------STNGNGASKVVSGLNGTQPLKRDRHPVWYPPTQDDGK-------------------------------------------------

*L. pyrrhocoris*  M-----------------ASTAKVVAGLNGEHPVKRDRHPVWYPPTQVDGK-------------------------------------------------

*L. seymouri*  M------------HSTANAAWAKVAGGLDGEHPIKRDRHPIWYPPTQVDGK-------------------------------------------------

*L. arabica*  ---------------MSTNRIATVVAGLDGANPVKRERHPAWYPPLKLDGN-------------------------------------------------

*L. aethiopica*  ---------------MSTNRVATVVAGLDGANPVKRERHPAWYPPLKLDGN-------------------------------------------------

*L. donovani*  ---------------MSANRVPTVVAGLDGANPVKRERHPAWYPPLKLDGN-------------------------------------------------

*L. gerbilli*  ---------------MSTNRVATVVAGLDGANPVKRERHPAWYPPLKLDGN-------------------------------------------------

*L. enriettii*  ---------------MSGDGESKVVAGLDGANPVKRDRHPVWYPPARQDGK-------------------------------------------------

*L. infantum*  ---------------MSANRVPTVVAGLDGANPVKRERHPAWYPPLKLDGN-------------------------------------------------

*L. panamensis*  ---------------MGRNHTSKVAAGLDGANPVKRDRHPVWYPPLKLDGN-------------------------------------------------

*L. tropica*  ---------------MSTNRVATVVAGLDGANPVKRERHPAWYPPLKLDGN-------------------------------------------------

*L. turanica*  ---------------MSTNRVATVVAGLDGANPVKRERHPAWYPPLKLDGN-------------------------------------------------

*L. major*  ---------------MSTNRVATVVAGLDGANPVKRERHPAWYPPLKLDGN-------------------------------------------------

*L. mexicana*  ---------------MSGNRPPTVVAGLDGANPVKRERHPTWYPPVKLDGN-------------------------------------------------

*T. cruzi*  M--------------STSKQQPQVVGGLKVTEPVRRPRHPPWYPPVQNDAK-------------------------------------------------

*T. brucei brucei*  M---------------KESDGLLLADGLNVCEPVKRSRHPPWYPPLNVDGN-------------------------------------------------

*T. brucei gambiense* M---------------KESDVLLLADGLNVCEPVKRSRHPPWYPPLNVDGN-------------------------------------------------

*T. evansi*  M---------------KESDGLLLADGLNVCEPVKRSRHPPWYPPLNVDGN-------------------------------------------------

110 120 130 140 150 160 170 180 190 200

....|....|....|....|....|....|....|....|....|....|....|....|....|....|....|....|....|....|....|....|

jaculum1 GLRVMNSLTETLEPFAPREGRVVRWYTCGPTVYDVAHMGHARAYLTFDILRRIMEDYFHYNVLYQMNITDIDDKIIKRARVNKLLADYKHKLQQLQQQQQ

jaculum2 GLKVLNSLTETIESFSPRSGRVVKWYTCGPTVYDVAHLGHARAYLTFDILRRIMEDYFHYPVIYQMNITDIDDKIIKRARVNELLHNFKEKKLQ------

*C. fasciculata*  GLRVANSLTETIEPFAPREGRLVRWYTCGPTVYDLSHMGHARAYLTFDIIRRIMEDYFGYSVIYQMNITDIDDKIIKRARVNKLLDDFKADELH------

*L. pyrrhocoris*  GLKVANSLTETVEDFAPLDGRLVRWYTCGPTVYDLSHMGHARAYLTFDILRRIMEDYFGYSVIYQMNITDIDDKIIKRARVNKLLDDFKAGELH------

*L. seymouri*  GLKVANSLTETTEAFAPREGRLVRWYTCGPTVYDLSHMGHARAYLTFDILRRIMEDYFGYAVIYQMNITDIDDKIIKRARVNKLLDDFKEVDLH------

*L. arabica*  GLKVMNSLTEALEDFAPRDGRVVRWYTCGPTVYDLSHMGHARAYLTFDIIRRIMEDYFGYSVIYQMNITDIDDKIIKRARVNKLLVDFKEGELR------

*L. aethiopica*  GLKVMNSLTETLEDFAPRDGRVVRWYTCGPTVYDLSHMGHARAYLTFDIIRRIMEDYFGYSVIYQMNITDIDDKIIKRARVNKLLDDFKEAQLR------

*L. donovani*  GLKVMNSLTETLEDFAPRDGRVVRWYTCGPTVYDLSHMGHARAYLTFDIIRRVMEDYFGYSVIYQMNITDIDDKIIKRARVNKLLDDFKEGELR------

*L. gerbilli*  GLKVMNSLTETLEDFAPRDGRVVRWYTCGPTVYDLSHMGHARAYLTFDIIRRIMEDYFGYSVIYQMNITDIDDKIIKRARVNKLLVDFKEGELR------

*L. enriettii*  GLKVMNSLTESLEEFAPRDGRLVRWYTCGPTVYDQSHMGHARAYLTFDIIRRIMEDYFGYSVIYQMNITDIDDKIIKRARVNKLLDDFKEDELH------

*L. infantum*  GLKVMNSLTETLEDFAPRDGRVVRWYTCGPTVYDLSHMGHARAYLTFDIIRRVMEDYFGYSVIYQMNITDIDDKIIKRARVNKLLDDFKEGELR------

*L. panamensis*  GLKVMNSLTETLEDFAPRDGRVVRWYTCGPTVYDLSHMGHARAYLTFDIIRRIMEDYFGYSVLYQMNITDIDDKIIKRARVGKLLDDFKEGELH------

*L. tropica*  GLKVMNSLTETLEDFAPRDGRVVRWYTCGPTVYDLSHMGHARAYLTFDIIRRIMEDYFGYSVIYQMNITDIDDKIIKRARVNKLLDDFKEGELR------

*L. turanica*  GLKVMNSLTETLEDFAPRDGRVVRWYTCGPTVYDLSHMGHARAYLTFDIIRRIMEDYFGYSVIYQMNITDIDDKIIKRARVNKLLVDFKEGELR------

*L. major*  GLKVMNSLTETLEDFAPRDGRVVRWYTCGPTVYDLSHMGHARAYLTFDIIRRIMEDYFGYSVIYQMNITDIDDKIIKRARVNKLLVDFKEEELR------

*L. mexicana*  GLKVMNSLTESLEDFAPRDGRVVRWYTCGPTVYDLSHMGHARAYLTFDIIRRIMEDYFGYSVIYQVNITDIDDKIIKRARVNKLLDDFKEGELR------

*T. cruzi*  GLRVLNSLTESVEPFAPREGRLVRWYACGPTVYELSHMGHARAYLTFDILRRIMEDYFGYQVVYQMNITDIDDKIIKRARISALLKRFREDFLQ------

*T. brucei brucei*  DVCVLNSMTECLEKFAPREGRLVRWYTCGPTVYDVSHMGHARAYLTFDILRRIMEDFFGYKVIYQMNITDIDDKIIKRARVSSLLRHFRDVTLE------

*T. brucei gambiense* DVCVLNSMTECLEKFAPREGRLVRWYTCGPTVYDVSHMGHARAYLTFDILRRIMEDFFGYKVIYQMNITDIDDKIIKRARVSSLLRHFRDVTLE------

*T. evansi*  DVCVLNSMTECLEKFAPREGRLVRWYTCGPTVYDVSHMGHARAYLTFDILRRIMEDFFGYKVIYQMNITDIDDKIIKRARVSSLLRHFRDVTLE------

210 220 230 240 250 260 270 280 290 300

....|....|....|....|....|....|....|....|....|....|....|....|....|....|....|....|....|....|....|....|

jaculum1 QKSATTTAAAAAGAHDKNEGDEAQRGEADGDAAWQQLCAFTDDALRAATTSLSARRSKLAQPLPADA-NSRAKAERDEKLLELELRESQLSETRERVAAA

jaculum2 ------------------------------G-NFEELKSFTQEALNVAENNLARKRKKLSEPLPANVNNSRLRTEREEKLKELQLKEAQLVETDKAIAAA

*C. fasciculata*  ------------------------------GNDLAKLQAFTAKAVAAAEASLARRKAKLAEPIPDGA-NSRAKTDREEKLMEAELKTTQLVETKEKIAAA

*L. pyrrhocoris*  ------------------------------DGDLAKLKAFTTEAVAAAEASLAKRKAKLAEPIPENA-NSRAKTDRDEKLMEAELKTAQLAETREKIAAA

*L. seymouri*  ------------------------------GSDVAKLKTFTAEAVAAAEAGLAKRKAKLAEPIPDNA-NSRAKTDREEKLMEAELKTAQLAETKEKIAAA

*L. arabica*  ------------------------------HSDVAKLRAFTAEAVAAAESGLAKRKAALVEPIAEGA-NSRAKADREEKLLEVQLKESQLVETKVKIAAA

*L. aethiopica*  ------------------------------HSDVAKLMAFTAEAVAAAESGLAKRKAALVEPITDGA-NSRAKADREEKLLEVQLKESQLVETKVKITAA

*L. donovani*  ------------------------------HGDVARLMAFTAEAVTAAESGLAKRKAALFEPITEGA-SSRAKADREEKLLEVQLKESQLVETKVKIAAA

*L. gerbilli*  ------------------------------HSDVAKLMAFTAEAVAAAESGLAKRKAALVEPIAEGA-NSRAKADREEKLLEVQLKESQLVETKVKIAAA

*L. enriettii*  ------------------------------NSDVAKLMAFTAEAVAAAESSLAKRKAKLSEPIPEGA-NSRAKADREEKLLELQLKESQLAETKERIAAA

*L. infantum*  ------------------------------HGDVARLMAFTAEAVTAAESGLAKRKAALFEPITEGA-SSRAKADREEKLLEVQLKESQLVETKVKIAAA

*L. panamensis*  ------------------------------RSNVEKLVAFTAEAVAAAESNLAKRKAKLSEPIPEGA-NSRVKADHEEKMLELQLKESQLAETKEKIAAA

*L. tropica*  ------------------------------HSDVAKLMAFTAEAVAAAESGLAKRKAALVEPITEGP-NSRAKADREEKLLEVQLKESQLVETKVKITAA

*L. turanica*  ------------------------------HSDVAKLMAFTAEAVAAAESGLAKRKAALVEPIAEGA-NSRAKADREEKLLEVQLKESQLEETKVKIAAA

*L. major*  ------------------------------HSDVAKLMAFTAEAVAAAESGLAKRKAALVEPIAKGT-NSRAKADREEKLLEVQLKESQLVETKVKIAAA

*L. mexicana*  ------------------------------HSDVAKLMAFTTEAVAAAESGLEKRKAALLEPITDGA-NSRAKADREEKMLEVQLKESQLAETKVKIAAA

*T. cruzi*  ------------------------------GSDMARLVAFTAEAETAAVEALELARIKLSEPLPDKT-PSRIKAEREEKQMELALKESQLQKTRERIASA

*T. brucei brucei*  ------------------------------GGNMEKLVKFTVEAQRSASRALSETREKLSQALPEGT-SSRVRMEREEKIMELALKETQFSDTSGRIQSA

*T. brucei gambiense* ------------------------------GGNMEKLVKFTVEAQRSASRALSETREKLSQALPEGT-SSRVRMEREEKIMELALKETQFSDTSGRIQSA

*T. evansi*  ------------------------------GGNMEKLVKFTVEAQRSASRALSETREKLSQALPEGT-SSRVRMEREEKIMELALKETQFSDTSGRIQSA

310 320 330 340 350 360 370 380 390 400

....|....|....|....|....|....|....|....|....|....|....|....|....|....|....|....|....|....|....|....|

jaculum1 RATRQLPTLFDAASAVNGELLDALDGDKVTDADIFDAHARTYERAFFDDMQRLGVRPPDVVTRVTEYVPQVVAFITRIMQHGFAYVAQSSVFFDTEAFVK

jaculum2 IANEDFEGTFTAAKDVNGELLDEREGATVTNQSIFEAHARQYERSFFHDMNRLGIREPDIVTRVTEYVPQVVEFVQQIINNGFAYMGTSSIFFDTQAYVH

*C. fasciculata*  --QSDFEALFAAASGVNGDLLDEQKGDSISDQQIFEDHARKYERAFFEDMQRLGIRDPDIVSRVTEYVPQVVTFVQKIIDNGFAYQGESSIFFDTEAYIR

*L. pyrrhocoris*  --KDDFEALFAAASSVNGDLLDEQKGDSICDQQIFEDHARKYERAFFDDMQRLGIREPDIVTRVTEYVPQVVEFVQTIIDNGFAYQGESSVFFDTEAYIK

*L. seymouri*  --KDDFEALFAAASGVNGDLLDELKGHSICDQQIFENHARQYERAFFDDMQRLGIRDPDIVTRVTEYVPEVVTFIQKIMDNGFAYKGESSIFFDTEAYVK

*L. arabica*  --KDDFAALFAAASGVNGDLLDERSGHSVSDQQIFEDHARKYERAFFEDMQRLGIREPDIVTRVTEYVPQVVEFVQKIIDNGFAYAGETSVFFDTEAYIR

*L. aethiopica*  --KDDFAALFAAASGVNGDLLDERSGPSVSDQQIFEDHARKYERAFFEDMQRLGIREPDIVTRVTEYVPQVVEFVEKIIDNGFAYVGETSVFFDTEAYIR

*L. donovani*  --KDDFAALFAAASGVNGDLLDERSGHSVSDQQIFEDHARKYERAFFEDMQRLGIREPDIVTRVTEYVPQVVEFVQKIIDNGFAYVGETSVFFDTEAYIR

*L. gerbilli*  --KDDFAALFAAASGVNGDLLDERSGHSVSDQQIFEDHARKYERAFFEDMQRLGIREPDIVTRVTEYVPQVVEFVQKIIDNGFAYAGETSVFFDTEAYIR

*L. enriettii*  --GDDFAALFAAASGVNGDLLDERNGHRISNQQIFEDHARKYERAFFEDAQRLGVREPDIVTRVTEYVPKVVKFVQKIIDNGFAYVGETSVFFDTEAYVR

*L. infantum*  --KDDFAALFAAASGVNGDLLDERSGHSVSDQQIFEDHARKYERAFFEDMQRLGIREPDIVTRVTEYVPQVVEFVQKIIDNGFAYVGETSVFFDTEAYIR

*L. panamensis*  --KDDFAALFAAASGVNGDLLDERNGHTISDQQIFEDHARKYECAFFEDMQRLGIRDPDIVSRVTEYVPQVVEFVQKIIDNGFAYVGETSVFFDTEAYIR

*L. tropica*  --KDDFAALFAAASGVNGDLLDERSGHSVSDQQIFEDHARKYERAFFEDMQRLGIREPDIVTRVTEYVPQVVEFVQKIIDNGFAYVGETSVFFDTEAYIR

*L. turanica*  --KDDFAALFAAASGVNGDLLDERSGHSVSDQQIFEDHARKYERAFFEDMQRLGIREPDIVTRVTEYVPQVVEFVQKIIDNGFAYAGETSVFFDTEAYIR

*L. major*  --KDDFAALFAAASGVNGDLLDERSGHSVSDQQIFEDHARKYERAFFEDMQRLGIREPDIVTRVTEYVPQVVEFVQKIIDNGFAYAGETSVFFDTEAYIR

*L. mexicana*  --KDDFAALFAAASGVNGDLLDERSGHSVSDQQIFEDHARKYERAFFEDMQRLGIREPDIVTRVTEYVPQVVEFVQKIIDNGFAYVGETSVFFDTEAYIR

*T. cruzi*  TASGNFDELFNAASGINGDLLDEREGQYVTDQSIFEDHARLYERLFFEDMRRLGVRDPDVVSRVTEYVPQVVDFIQKIMDNGFAYRGTSSIFFDTTAFLR

*T. brucei brucei*  IKAGDFDELFDAASGINGDLLDQLEGHTVTDQKIFDDHARRYERLFFEDMKRLGVKDPDVITRVTEYVPQVVNFIQRIMDNGFAYSGETSVFFDTTAFIR

*T. brucei gambiense* IKAGDFDELFDAASGINGDLLDQLEGHTVTDQKIFDDHARRYERLFFEDMKRLGVKDPDVITRVTEYVPQVVNFIQRIMDNGFAYSGETSVFFDTTAFIR

*T. evansi*  IKAGDFDELFDAASGINGDLLDQLEGHTVTDQKIFDDHARRYERLFFEDMKRLGVKDPDVITRVTEYVPQVVNFIQRIMDNGFAYSGETSVFFDTTAFIR

410 420 430 440 450 460 470 480 490 500

....|....|....|....|....|....|....|....|....|....|....|....|....|....|....|....|....|....|....|....|

jaculum1 AGHDYPKLKP----------------------GGERNASEEEMAEGEGALSRAAASEKRSANDFALWKFSKPGEPRWPSPWGAGRPGWHIECSVMASDVL

jaculum2 AGHSLPKLKPISASAREAFTATATNANNGNNDVTEAVTTEEEMAEGEGVLSTALANEKKHPNDFALWKFSKPGEPAWPSPWGNGRPGWHIECSVMASDIL

*C. fasciculata*  AGHDYPKLKP----------------------GGDKNTTEDEMAEGEGALSKGVEGEKRSPNDFALWKFSKPGEPRWPSPWGEGRPGWHIECSVMASDIL

*L. pyrrhocoris*  AGHDYPKLKP----------------------GGDRNTTEDEMAEGEGALSKGVEGEKRSPNDFALWKFSKPGEPRWPSPWGEGRPGWHIECSVMASDIL

*L. seymouri*  AGHDYPKLKP----------------------GGDRNTTDDEMAEGEGALSKGVEGEKRSPNDFALWKFSKPGEPRWPSPWGEGRPGWHIECSVMASDVL

*L. arabica*  AGHDYPKLKP----------------------GGDRNTTEDEMAEGEGVLSKGIEGEKRSPNDFALWKFSKPGEPRWPSPWGEGRPGWHIECSVMASDIL

*L. aethiopica*  AGHDYPKLKP----------------------GGDRNTTEDEMAEGEGVLSKGIEGEKRSPNDFALWKFSKPGEPRWPSPWGEGRPGWHIECSVMASDIL

*L. donovani*  AGHDYPKLKP----------------------GGDRNTTEDEMAEGEGVLSKGIEGEKRSPNDFALWKFSKPGEPRWPSPWGEGRPGWHIECSVMASDIL

*L. gerbilli*  AGHDYPKLKP----------------------GGDRNTTEDEMAEGEGVLSKGIEGEKRSPNDFALWKFSKPGEPRWPSPWGEGRPGWHIECSVMASDIL

*L. enriettii*  AGHDYPKLKP----------------------GGDRSTTEDEMAEGEGALTKGIEGEKRSPNDFALWKFSKPGEPRWPSPWGEGRPGWHIECSVMASDIL

*L. infantum*  AGHDYPKLKP----------------------GGDRNTTEDEMAEGEGVLSKGIEGEKRSPNDFALWKFSKPGEPRWPSPWGEGRPGWHIECSVMASDIL

*L. panamensis*  AGHDYPKLKP----------------------GGDRNTTEEEMAEGEGTLTKAVEGEKRSSNDFALWKFSKPGEPRWPSPWGAGRPGWHIECSVMASDVL

*L. tropica*  AGHDYPKLKP----------------------GGDRNTTEDEMAEGEGVLSKGIEGEKRSPNDFALWKFSKPGEPRWPSPWGEGRPGWHIECSVMASDIL

*L. turanica*  AGHDYPKLKP----------------------GGDRNTTEDEMAEGEGVLSKGIEGEKRSPNDFALWKFSKPGEPRWPSPWGEGRPGWHIECSVMASDIL

*L. major*  AGHDYPKLKP----------------------GGDRNTTEDEMAEGEGVLSKGIEGEKRSPNDFALWKFSKPGEPRWPSPWGEGRPGWHIECSVMASDIL

*L. mexicana*  AGHDYPKLKP----------------------GGDRNTTEDEMAEGEGVLSKGVEGEKRSPNDFALWKFSKPGEPRWSSPWGEGRPGWHIECSVMASDIL

*T. cruzi*  AGHDYPKLKPVS-------------------ESGENDATEAEMAEGEGALTVGVAGEKRNANDFALWKFSKPGEPRWPSPWGEGRPGWHIECSVMASDIL

*T. brucei brucei*  AGHNYPKLKPIS-------------------ERDECNTTEAEMAEGEGALAACVAGEKRSPNDFALWKFSKPGEPHWPSPWGAGRPGWHIECSVMASDIL

*T. brucei gambiense* AGHNYPKLKPIS-------------------ERDECNTTEAEMAEGEGALAACVAGEKRSPNDFALWKFSKPGEPHWPSPWGAGRPGWHIECSVMASDIL

*T. evansi*  AGHNYPKLKPIS-------------------ERDECNTTEAEMAEGEGALAACVAGEKRSPNDFALWKFSKPGEPHWPSPWGAGRPGWHIECSVMASDIL

510 520 530 540 550 560 570 580 590 600

....|....|....|....|....|....|....|....|....|....|....|....|....|....|....|....|....|....|....|....|

jaculum1 GSNMDIHSGGWDLKFPHHDNECAQSEACNLQHQWVNYFLHCGHLHIKGLKMSKSLKNFITIRQALDELGVNARTMRLLFLANAWHKPMNFSDQSLDEARE

jaculum2 GQNMDIHSGGMDLKFPHHDNECAQSEACFNCGQWVNFFFHCGHLHIQGLKMSKSLKNFITIQQALDELKVTPRLMRLLFLANNWYKPMNFSDQSLDEARE

*C. fasciculata*  GENMDIHSGGWDLKFPHHDNECAQSEACNLHNQWVNYFLHCGHLHIKGLKMSKSLKNFITIRQSLDELGVSPRTMRLLFLANPWNKPMNFSDQSLDEAKE

*L. pyrrhocoris*  GANMDIHSGGWDLKFPHHDNECAQSEACNLHSQWVNYFLHCGHLHIKGLKMSKSLKNFITIRQALDDLGVTPRTMRLLFLANPWNKPMNFSDQSLDEAKE

*L. seymouri*  GDNMDIHSGGWDLKFPHHDNECAQSEACNLQSQWVNYFLHCGHLHIKGLKMSKSLKNFITIRQALDDLGVTPRTMRLLFLANPWNKPMNFSDQSLDEAKE

*L. arabica*  GENMDIHSGGWDLKFPHHDNECAQSEACNLHSQWVNYFLHCGHLHIKGLKMSKSLKNFITIRQALDELGVTARTMRLLFLANPWNKPMNFSDQSLDEAKE

*L. aethiopica*  GENMDIHSGGWDLKFPHHDNECAQSEACNLHSQWVNYFLHCGHLHIKGLKMSKSLKNFITIRQALDELGVTARTMRLLFLANPWNKPMNFSDQSLDEAKE

*L. donovani*  GENMDIHSGGWDLKFPHHDNECAQSEACNLHSQWVNYFLHCGHLHIKGLKMSKSLKNFITIRQALDELGVTARTMRLLFLANPWNKPMNFSDQSLDEAKE

*L. gerbilli*  GENMDIHSGGWDLKFPHHDNECAQSEACNLHSQWVNYFLHCGHLHIKGLKMSKSLKNFITIRQALDELGVTARTMRLLFLANPWNKPMNFSDQSLDEAKE

*L. enriettii*  GENMDIHSGGWDLKFPHHDNECAQSEACNLHHQWVNYFLHCGHLHIKGLKMSKSLKNFITIRQALDDLGVTPRTMRLLFLANPWNKPMNFSDQSLDEAKE

*L. infantum*  GENMDIHSGGWDLKFPHHDNECAQSEACNLHSQWVNYFLHCGHLHIKGLKMSKSLKNFITIRQALDELGVTARTMRLLFLANPWNKPMNFSDQSLDEAKE

*L. panamensis*  GENMDIHSGGWDLKFPHHDNECAQSEACNLHNQWVNYFLHCGHLHIKGLKMSKSLKNFITIRQALDDLGVTPRTMRLLFLASPWYKPMNFSDQSLDEAKE

*L. tropica*  GENMDIHSGGWDLKFPHHDNECAQSEACNLHSQWVNYFLHCGHLHIKGLKMSKSLKNFITIRQALDELGVTARTMRLLFLANPWNKPMNFSDQSLDEAKE

*L. turanica*  GENMDIHSGGWDLKFPHHDNECAQSEACNLHSQWVNYFLHCGHLHIKGLKMSKSLKNFITIRQALDELGVTARTMRLLFLANPWNKPMNFSDQSLDEAKE

*L. major*  GENMDIHSGGWDLKFPHHDNECAQSEACNLHSQWVNYFLHCGHLHIKGLKMSKSLKNFITIRQALDELGVTARTMRLLFLANPWNKPMNFSDQSLDEAKE

*L. mexicana*  GENMDIHSGGWDLKFPHHDNECAQSEACNLHSQWVNYFLHCGHLHIKGLKMSKSLKNFITIRQALDELGVTARTMRLLFLANPWNKPMNFSDQSLDEAKE

*T. cruzi*  GTNIDVHSGGWDLKFPHHDNECAQSEACHMQHQWINYFLHCGHLHIKGLKMSKSLKNFITIRQALDELDVTPRVMRLLFLANHWGKPMNFSDQSIEEAKE

*T. brucei brucei*  GTNMDIHSGGCDLKFPHHDNECAQSEAYSMQHQWVNYFLHCGHLHIKGLKMSKSLKNFITIRHALDDLGVTPRTMRLLFLANQWNKAMNFSDQSIDEAKE

*T. brucei gambiense* GTNMDIHSGGCDLKFPHHDNECAQSEAYSMQHQWVNYFLHCGHLHIKGLKMSKSLKNFITIRHALDDLGVTPRTMRLLFLANQWNKAMNFSDQSIDEAKE

*T. evansi*  GTNMDIHSGGCDLKFPHHDNECAQSEAYSMQHQWVNYFLHCGHLHIKGLKMSKSLKNFITIRHALDDLGVTPRTMRLLFLANQWNKAMNFSDQSIDEAKE

610 620 630 640 650 660 670 680 690 700

....|....|....|....|....|....|....|....|....|....|....|....|....|....|....|....|....|....|....|....|

jaculum1 RERVLRAFFGSVEVALRGGNAAAWQQPQGLQPHDRELLERWRACELAVHAALLNNFDTPTALQQLLGLVAATNQYLLC------GE-----RISATLLRK

jaculum2 RERVLRSFFGSLEAILRQDY---WSHTQGATDSDRELLRKWEACEDEVHVALQSNFDTPSALNSLLSLVSSTNQYMVMCNSSSSGDTSDNNRPSATLLRK

*C. fasciculata*  KERVLRAFFGSIDIVLRGDN---WSATQGANSQDRELLNRWVEIEAAVHAALQDNFDTVTALQQLMALVAATNQYLLS------GE-----RPSATLLRK

*L. pyrrhocoris*  KERVLRAFFGSIDIVLRGDA---WALPQGANTQDRELLNRWVEVEGAVHAALQDNFDTVTALQQLMSLVAATNQYLLS------GE-----RPSATLLRK

*L. seymouri*  KERVLRAFFGSIDIVLRGDN---WKSAQGANARDRELLNKWVETEGAVHAALQDNFDTVTALQQLMSLVAATNQYLLT------GE-----RPSATLLRK

*L. arabica*  KERVLRAFFGSVDIVLRADN---WKATQGVNKHDRELLSKWVEAEAAVHAALQDNFDTVVALQQLMSLVAATNQYLLS------GE-----RPSATLVRK

*L. aethiopica*  KERVLRAFFGSVDIVLRADN---WKATQGVNKHDRELLSKWVEAEAAVHAALQDNFDTVVALQQLMSLVSATNQYLLS------GE-----RPSGTLVRK

*L. donovani*  KERVLRAFFGSVDIVLRTDN---WKATQGANKHDRELLSKWVEAEAAVHAALQDNFDTVVALQQLMSLVAATNQYLLS------GE-----RPSATLVRK

*L. gerbilli*  KERVLRAFFGSVDIVLRADN---WKATQGVNKHDRELLSKWVEAEAAVHAALQDNFDTVVALQQLMSLVAATNQYLLS------GE-----RPSATLVRK

*L. enriettii*  KERVLRAFFGSVDIVLRADN---WKATQGANTHDRELLAKWVDVEAAVHAALLDNFDTVVALQQLMSLVAATNQYLLS------GE-----RASATLVRK

*L. infantum*  KERVLRAFFGSVDIVLRTDN---WKATQGANKHDRELLSKWVEAEAAVHAALQDNFDTVVALQQLMSLVAATNQYLLS------GE-----RPSATLVRK

*L. panamensis*  KERVLRAFFGSVDIVLRADN---WKATQGVNTYDRELLGKCIEAEAAVHAALQDNFDTVVALQQLMSLVAATNQYLLS------GE-----RPSATLVRK

*L. tropica*  KERVLRAFFGSVDIVLRADN---WKATQGVNKHDRELLSKWVEAEAAVHAALQDNFDTVVALQQLMSLVSATNQYLLS------GE-----RPSGTLVRK

*L. turanica*  KERVLRAFFGSVDIVLRADN---WKATQGVNKHDRELLSKWVEAEAAVHAALQDNFDTVVALQQLMSLVAATNQYLLS------GE-----RPSATLVRK

*L. major*  KERVLRAFFGSVDIVLRADN---WKATQGVNKHDRELLSKWVEAEAAVHAALQDNFDTVVALQQLMSLVAATNQYLLS------GE-----RPSATLVRK

*L. mexicana*  KERVLRAFFGSVDIVLRADN---WKATQGVNKHDRELLSKWVEAEAAVHAALQDNFDTVVALQQLMSLVAATNQYLLS------GE-----RPSATLVRK

*T. cruzi*  RERVLRAFFGSVDIVLRKDN---WGETQGLNGHDRRLQETWLTAENAVHGALQNNFDTPAAMDSVMGLVGATNHYLLS------GE-----RPSATLVRK

*T. brucei brucei*  RERVLRSFFGSVDMVLRSDT---LKEIQGFNEHDRKLNEAWISTESAVDAALRNNFDTPTAMEAIMGLVSETNRYLVT------GQ-----RPSATLVHK

*T. brucei gambiense* RERVLRSFFGSVDMVLRSDT---LKEIQGFNEHDRKLNEAWISTESAVDAALRNNFDTPTAMEAIMGLVSETNRYLVT------GQ-----RPSATLVHK

*T. evansi*  RERVLRSFFGSVDMVLRSDT---LKEIQGFNEHDRKLNEAWISTESAVDAALRNNFDTPTAMEAIMGLVSETNRYLVT------GQ-----RPSATLVHK

710 720 730 740 750 760 770 780 790 800

....|....|....|....|....|....|....|....|....|....|....|....|....|....|....|....|....|....|....|....|

jaculum1 VARYLTDMFRTFGVIEGNDAIGLSATAGGVGGAARNKKGGSANNTQRQRRTARAAARAAEGEAASSHASGSDKNDATYDDNDDDDGDDSGDAAEANGDNF

jaculum2 IGNYITQMFAVFGVIDGNDTIGFHGAGSRNSEI-------GGNNRDNNNEDDEKITVSVEAEPISPNADKALQTDQMANK------ESTSQIRKVQYNKF

*C. fasciculata*  VGAYITKMFRVFGVVEGSDVVGLQQQASGDGE-----------------------------------------------------------------ARF

*L. pyrrhocoris*  VGRYITKMFRVFGVVEGSDDVGLQQQSTGDGE-----------------------------------------------------------------ARV

*L. seymouri*  VGRYVTKMLHIFGVVEGVDDVGLQQQSSGDGE-----------------------------------------------------------------ARF

*L. arabica*  VGCYVTKMFRIFGVVEGSDDVGLQKQGSGDGEA-----------------------------------------------------------------RF

*L. aethiopica*  VGCYVTKMFRVFGVVEGSDDVGLQKQGSGDGEA-----------------------------------------------------------------RF

*L. donovani*  VGCYVTKMFRIFGVVEGSDDVGLQKQGSGDGEA-----------------------------------------------------------------RF

*L. gerbilli*  VGCYVTKMFRIFGVVEGSDDVGLQKQGSGDGEA-----------------------------------------------------------------RF

*L. enriettii*  VGCYVTKMFRVFGVVEGSDDVGLQKQGSGDGEA-----------------------------------------------------------------RF

*L. infantum*  VGCYVTKMFRIFGVVEGSDDVGLQKQGSGDGEA-----------------------------------------------------------------RF

*L. panamensis*  VGCYVTKMFRVFGVVEGSDDVGLQKQGSGDDET-----------------------------------------------------------------RF

*L. tropica*  VGCYVTKMFRVFGVVEGSDNVGLQKQGSGDGEA-----------------------------------------------------------------RF

*L. turanica*  VGCYVTKMFRIFGVVEGSDDVGLQKQGSGDGEA-----------------------------------------------------------------RF

*L. major*  VGCYVTKMFRIFGVVEGSDDVGLQKQGSDDGEA-----------------------------------------------------------------RF

*L. mexicana*  VGCYVTKMFRIFGVVEGSDDVGLQKQGSGDGEA-----------------------------------------------------------------RF

*T. cruzi*  VGRYVTRILQVFGVVEGNDMVGFATQQGSDD-------------------------------------------------------------------RL

*T. brucei brucei*  VGRYVTRILQVFGVVDGNDMVGFTKTRQTDD-------------------------------------------------------------------QL

*T. brucei gambiense* VGRYVTRILQVFGVVDGNDMVGFTKTRQTDD-------------------------------------------------------------------QL

*T. evansi*  VGRYVTRILQVFGVVDGNDMVGFTKTRQTDD-------------------------------------------------------------------QL

810 820 830 840 850 860 870 880 890 900

....|....|....|....|....|....|....|....|....|....|....|....|....|....|....|....|....|....|....|....|

jaculum1 VRVVDALVAFRDGVREAARQSGTAASQALLPLCDRVRDEWLVDAGVRLEDKPGGATVWKSDDPAILRQELQARKQQQANERLQKLRNVIDKQRKLVDKWQ

jaculum2 DYVVDALVKFRDAIREEAKHNESVR-ASFLPLCDALRDEKLIHAGIRLEDNPAGPTTWKRDDPALLREELAARKAQQESDKLRKLTNQAEKLRKVISKWE

*C. fasciculata*  IEVVNTLVRFRDDVRDAAKEQKVAP--GFLPLCDKVRDEWLVDAGVRLEDSPAGPTTWKNDEPALLRKELQERRAQVEGDRRKKLANQAETKRKLVEKWE

*L. pyrrhocoris*  IAVVDTLVRFRDAVREAAKAEKVVP--VFLPLCDKVRDEWLVDAGVRLEDSPAGPTTWKNDEPALLRKELQERREQQEGDRKKKLANQVETKRKLVEKWA

*L. seymouri*  IEAVNTLVRFRDGVRDTAKAQKAVN--VFLPLCDKVRDEWLIDAGVRLEDNPAGPTTWKNDEPALLRKELQERLEQQDGERKKKLANQIETKRKLVEKWQ

*L. arabica*  IEVVNTLVRFRDEVRDAAKDHKVVA--GFLPLCDKVRDEWLVDAGVRLEDNPAGPTTWKSDEPALLHKELAERRAQQEGDRQRRLANQAETKRKLVEKWR

*L. aethiopica*  IEVVNTLVRFRDEVRDAAKDHKVVA--GFLPLCDKVRDEWLVDAGVRLEDNPAGPTTWKSDEPALLHKELAERRAQQEGDRQRRLANQAETKRKLVEKWR

*L. donovani*  IEVVNTLVRFRDEVRDAAKDHKVVA--GFLPLCDKVRDEWLVDAGVRLEDNPAGPTTWKSDEPALLHKELAERRAQQEGDRQRRLANQAETKRKLVEKWR

*L. gerbilli*  IEVVNTLVRFRDEVRDAAKDHKVVA--GFLPLCDKVRDEWLVDAGVRLEDNPAGPTTWKSDEPALLHKELAERRAQQEGDRQRRLANQAETKRKLVEKWR

*L. enriettii*  IEVVNALVRFRDEVRDAAKEQKVML--SFLPLCDKVRDEWLVDAGVRLEDSPAGRTTWKSDEPTLLRRELAERRSQQEGDRKKKLANQAETKRKLVEKWR

*L. infantum*  IEVVNTLVRFRDEVRDAAKDHKVVA--GFLPLCDKVRDEWLVDAGVRLEDNPAGPTTWKSDEPALLHKELAERRAQQEGDRQRRLANQAETKRKLVEKWR

*L. panamensis*  IEVMNALVRFRDEVRNAAKEQKAVA--AFLPLCDKVRDEWLVDAGVRLEDNPAGPTAWKSDEPALLRKELAERRAQQEGDRKKKLTNQAETKRRLVEKWR

*L. tropica*  IEVVNTLVRFRDEVRDAAKDHKVVA--GFLPLCDKVRDEWLVDAGVRLEDNPAGPTTWKSDEPALLHKELAERRAQQEGDRQRRLANQAETKRKLVEKWR

*L. turanica*  IEVVNTLVRFRDEVRDAAKDHKVVA--GFLPLCDKVRDEWLVDAGVRLEDNPAGPTTWKSDEPALLHKELAERRAQQEGDRQRRLANQAETKRKLVEKWR

*L. major*  IEVVNTLVRFRDEVRNAAKDHKVVA--GFLPLCDKVRDEWLVDAGVRLEDNPAGPTTWKSDEPALLQKELAERRAQQEGDRQRRLANQAETKRKLVEKWR

*L. mexicana*  IEVVNTLVRFRDEVRDAAKDQKVVA--GFLPLCDKVRDEWLVDAGVRLEDNPAGPTTWKNDEPALLHKELAERRAQQEGDRQRKLANQVETKRKLVEKWR

*T. cruzi*  LTVMDALLRFRDGVRDAAKVEKCTG--TFLPLCDAVRDEWLVPAGIRIEDNPAGPTTWKSDDVAVLQREVAERRAQQATERRTRLENQIETKRKLTEKWR

*T. brucei brucei*  VPVMEALLRFRDSVRSEAKASGTTA--NFLPLCDAIRDEWLAQAGIRIEDSPNGPTTWKRDDPAVLLREISERREQQANDRRRKLQNQIETKKKLVEKWR

*T. brucei gambiense* VPVMEALLRFRDSVRSEAKASGTTA--NFLPLCDAIRDEWLAQAGIRIEDSPNGPTTWKRDDPAVLLREISERREQQANDRRRKLQNQIETKKKLVEKWR

*T. evansi*  VPVMEALLRFRDSVRSEAKASGTTA--NFLPLCDAIRDEWLAQAGIRIEDSPNGPTTWKRDDPAVLLREISERREQQANDRRRKLQNQIETKKKLVEKWR

910 920 930 940 950 960 970 980 990 1000

....|....|....|....|....|....|....|....|....|....|....|....|....|....|....|....|....|....|....|....|

jaculum1 RYSCPPAAYLAQQEEARPAG----------------------------------ERKYRHFDPVSGLPHELISGELVSDKDLKKLEKEVAKYSKTYDEYI

jaculum2 KYLIAPKEYFVNKQAQQEQARETANDEEGKVQEVKKSGSDEQIDSADNSSQREEVKKYSAFDESTGLPITLSSGELVSEKEQKKLKKELDKYTKMYNDFD

*C. fasciculata*  QFTYPPSEFLRRQDEERGA-----------------------------------ARKYAAFDDATGLPTKTVDGEEVVEKETKKLAKEVAKYAKSYEEFV

*L. pyrrhocoris*  LFSYPPSEYFRRQDEQRGA-----------------------------------ERKYAVFDPSTGMPTTTVAGEEVGEKEVKKLSKEVAKYAKSYDEYV

*L. seymouri*  QFSCLPSEYLQRQDAQRGA-----------------------------------EKKYAAFDESTGLPTKTVAGEDVGEKEIKKFSKEVAKYAKTYEEFM

*L. arabica*  QLTYPPSEYFRRQDEQRV------------------------------------EKKYAAYDDVTGLPTMTAAGEEVSEKEAKKLSKEQAKYAKSYDEFI

*L. aethiopica*  QFTYPPSEYFRRQDEQRA------------------------------------EKKYAAYDDVTGLPTMTAAGEEVSEKEAKKLSKEQARYAKSYDEFI

*L. donovani*  QFTYPPSEYFRLQDEQRV------------------------------------EKKYAAYDDVTGLPTMTAAGEEVSEKEAKKLSKEQAKYAKSYDEFI

*L. gerbilli*  QFTYPPSEYFRRQDEQRV------------------------------------ERKYAAYDDVTGLPTMTAAGEEVNEKEAKKLSKEQAKYAKSYDEFI

*L. enriettii*  QFIYPPPDFFRRQDELRE------------------------------------QKKYIAYDDATGLPTKTADGEEVSEKELKKLSKEQAKYAKAYDEFL

*L. infantum*  QFTYPPSEYFRLQDEQRV------------------------------------EKKYAAYDDVTGLPTMTAAGEEVSEKEAKKLSKEQAKYAKSYDEFI

*L. panamensis*  QFVHPPSDFFRRQDE----------------------------------------KKYTAYDDVTGLPTTTATGEEVSEKELKKLSKEQAKYAKSYDEFV

*L. tropica*  QFTYPPSEYFRRQDEQRP------------------------------------EKKYAAYDDVTGLPTMTAAGEEVSEKEAKKLSKEQAKYAKSYDEFI

*L. turanica*  QFTYPPSEYFRRQDEQRV------------------------------------EKKYAAYDDVTGLPTMTAAGEEVSEKEAKKLSKEQAKYAKSYDEFI

*L. major*  QFTYPPSEYFRRQDEQRV------------------------------------EKKYTAYDDVTGLPTMTAAGEEVSEKEAKKLSKEQAKYAKSYDEFI

*L. mexicana*  QLTYPPSEYFRRQDEQRE------------------------------------EKKYAAYDDVTGLPTMTAAGEEVSEKDVKKLSKEQAKYAKSYDEFI

*T. cruzi*  QYTCTPIRYFSTRDTE--------------------------------------EKKYAAFDEATGLPTRRATGEALGEKEQKKLAKELARYAKVHEEFV

*T. brucei brucei*  NYTSSPKDYFKMQSG----------------------------------------SVYATFDEETGLPTSNSRGEVVGEKELKKLSKELAKYAKAHEEFN

*T. brucei gambiense* NYTSSPKDYFKMQSG----------------------------------------SVYATFDEETGLPTSNSRGEVVGEKELKKLSKELAKYAKAHEEFN

*T. evansi*  NYTSSPKDYFKMQSG----------------------------------------SVYATFDEETGLPTSNSRGEVVGEKELKKLSKELAKYAKAHEEFN

1010 1020 1030 1040

....|....|....|....|....|....|....|....|.

jaculum1 EKGGSEWLQQQKDALAASEEELRTLEAEANNATNASESNTQ

jaculum2 KLGNIAWYNEQKEELQRLENEIATLTHKK------------

*C. fasciculata*  AKGGEAWLKEQQEELAAMKVELASFS---------------

*L. pyrrhocoris*  SKGGESWLKEQQAELTRMMAELQGPK---------------

*L. seymouri*  SKGGAPWLREQEAELASMVAELQGSK---------------

*L. arabica*  GKGGVSWLQEQEAELASMVAELEGSK---------------

*L. aethiopica*  GKGGVSWLQEREAELASMVAEPEGSE---------------

*L. donovani*  GKGGVSWLQEQEAELASMVAELKGSN---------------

*L. gerbilli*  GKGGVSWLQEQEAELASMVAELEGSK---------------

*L. enriettii*  SKGGESWLLEQEAELASMAAELEGK----------------

*L. infantum*  GKGGVSWLQEQEAELASMVAELKGSN---------------

*L. panamensis*  SKGGVTWLQEQEAELASMVAELEDKK---------------

*L. tropica*  GKGGVSWLQEREAELASMVAEPEGSE---------------

*L. turanica*  GKGGVSWLQEQEAELASMVAELEGSK---------------

*L. major*  GKGGVSWIQEQEAELASMVAELEGSK---------------

*L. mexicana*  GKGGVSWLQEQEAELASMVAELEGSK---------------

*T. cruzi*  AKGGEGWLAEQEKELASLCQALESL----------------

*T. brucei brucei*  SKGGMEWLLEQEQELANMQESLKSTEVS-------------

*T. brucei gambiense* SKGGMEWLLEQEQELANMEESFKSTEVS-------------

*T. evansi*  SKGGMEWLLEQEQELANMEESFKSTEVS-------------

**Figure S1. Multiple insertions are present in “*jaculum*” proteins.** Full length protein alignments of chosen trypanosomatids are shown. Insertions present in “*jaculum*" proteins are highlighted by yellow background. Peptides identified by mass spectrometry are underlined in black. To display borders between neighboring identified peptides, black and red underlining is used.
